# Supplementary figures and images for: Assessing local cultural awareness in university EFL learners: A Delphi and AHP-based index framework
Source: PLoS One. 2025 Oct 8;20(10):e0332233. doi: 10.1371/journal.pone.0332233 (PMC12507305; doi:10.1371/journal.pone.0332233)

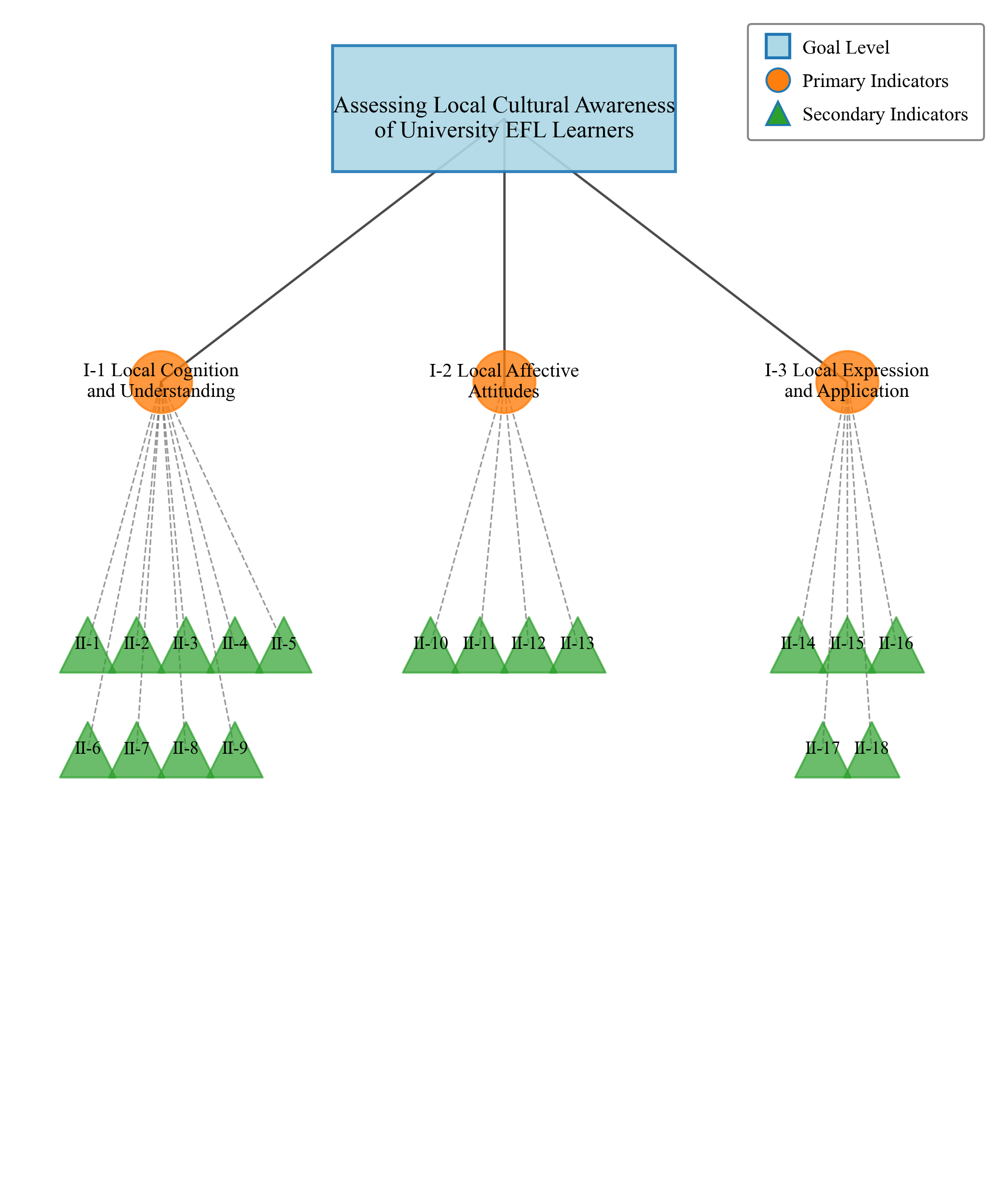

Supplement: S1 Fig — (TIFF) [file pone.0332233.s001.tiff]
