# Supplementary material for: Assessing local cultural awareness in university EFL learners: A Delphi and AHP-based index framework
Source: PLoS One. 2025 Oct 8;20(10):e0332233. doi: 10.1371/journal.pone.0332233 (PMC12507305; doi:10.1371/journal.pone.0332233)
Supplement: S1 Table — (DOCX) [file pone.0332233.s002.docx]

# S1 Table. First Delphi round consultation on secondary indicators for EFL learners' local awareness evaluation system.

| **Primary Indicator** | **Secondary Indicator** | **Description of Secondary Indicator** | **Assessment Methods** | **Importance Level** | | | | | **Comments** |
| --- | --- | --- | --- | --- | --- | --- | --- | --- | --- |
|  |  |  |  | 5 | 4 | 3 | 2 | 1 |  |
| A.Local Cognition and Understanding | A1 Local History | Learners gain an in-depth understanding of local historical events, historical figures, and their impact on modern society. | Oral Expression, Knowledge test, Written Report, etc. |  |  |  |  |  |  |
|  | A2 Regional Geography | Learners demonstrate their ability to use English to describe and connect geographical features, such as landscapes, rivers, mountains, and scenic spots, with local cultural practices, tourism, and community identity, etc. | Interpretation of Geographical Maps, Project Presentation, etc. |  |  |  |  |  |  |
|  | A3 Local Cultural Practices, Art, and Literature | Learners demonstrate their ability to use English to interpret and discuss local art forms and literary works, highlighting their significance as expressions of local culture and creativity, etc. | Analysis of Cultural Works, Discussion of Literary works, Art Exhibitions, etc. |  |  |  |  |  |  |
|  | A4 Local Development Achievements | Learners use English to explore and articulate the significance of local achievements and innovations in various fields, such as science, and technology, and their broader impact on society, etc. | Assessment Methods: Case Analysis, Project Reports, Data Interpretation, etc. |  |  |  |  |  |  |
|  | A5 National Virtues and Qualities | Learners demonstrate their ability to use English to interpret and discuss their nation's core values, such as resilience, hospitality, and social virtues. | Cultural Values Analysis, Discussion, Writing Tasks, etc. |  |  |  |  |  |  |
|  | A6 Daily Life Experiences | Learners demonstrate their ability to use English to describe and discuss aspects of local daily life, such as diet, family structure, education, and work environments, emphasizing their relevance to personal and community experiences, etc. | Oral narratives, Sharing of Life Experiences, Writing tasks, etc. |  |  |  |  |  |  |
|  | A7 Local Political System and Governance | Learners demonstrate their ability to use English to discuss and reflect on local governance structures and policies, emphasizing their impact on everyday life and the functioning of the community. | Political System Analysis, Written Reports, Debate Tasks, etc. |  |  |  |  |  |  |
|  | A8 Local Economic System | Learners use English to describe and discuss the role of local industries, agricultural practices, and businesses, emphasizing their contributions to community development. | Economic Data Analysis, Project Presentations, etc. |  |  |  |  |  |  |
|  | A9 Local Language and Dialect Varieties | Learners demonstrate their ability to use English to discuss local language varieties, including dialects or regional expressions, and their connections to Standard English in terms of usage, meaning, and cultural significance. | Oral Presentation, Comparative Essay,Role-Playing in Cross-Cultural Scenarios, Multimedia Projects, etc. |  |  |  |  |  |  |
|  | A10 Local Social Issues | Learners demonstrate their ability to use English to discuss current local social issues, including their causes, impacts, and potential solutions, while connecting these discussions to real-life examples and personal experiences. | Case Analysis of Social issues, Classroom Discussions, Project Reports, etc. |  |  |  |  |  |  |
|  | A11 Local Educational System | Learners employ English to describe and analyze key features of the local education system, focusing on its cultural relevance, strengths, and challenges in shaping community growth. | Comparative Analysis of Education Systems, Report Writing, etc. |  |  |  |  |  |  |
|  | A12 Local Environment and Sustainable Development | Learners use English to identify and discuss local environmental challenges, sustainable practices, and community responses, emphasizing their implications for local and global sustainability. | Environmental Policy Analysis, Sustainable Development Projects, Interpretation of Ecological data, etc. |  |  |  |  |  |  |
|  | A13 Local Ethical and Legal Systems | Learners'awareness of local ethical standards and legal systems and their implications for social behavior and cultural practices. | Case Study Analysis,Written Reports,Legal Text Analysis, etc. |  |  |  |  |  |  |
|  | A14 International relations and global issues relevant to their country | Learners' understanding of the international relations and global issues relevant to their country, including its engagement with the international community, regional partnerships, and the impact of global dynamics on local development. | Case Study and Analysis, Debate and Discussion, Research Paper or Essay, etc. |  |  |  |  |  |  |
|  | **Are there any indicators that need to be added in this dimension?** | | | | | | | |  |
|  |  | | | | | | | |  |
|  |  |  |  |  |  |  |  |  |  |
|  |  |  |  |  |  |  |  |  |  |
| B. Local Affective Attitudes | B1 Motivation to Express and Communicate Local Identity | Learners' motivation and willingness to express and disseminate local culture, identity, and values through English learning. | Surveys, Interviews and Reflective Journals, Task Selection and Completion, etc. |  |  |  |  |  |  |
|  | B2 Emotional Attachment to Local Culture | Learners' emotional attachment and identification with local culture, and willingness to convey these emotions in cross-cultural communication. | Reflective Journals, Peer and Teacher Feedback, Emotional Expression Tasks, etc. |  |  |  |  |  |  |
|  | B3 Cultural Pride | Learners' high identification and pride in local culture, and confidence demonstrated in cross-cultural communication. | Surveys, Oral Narratives, Classroom Participation Observation, etc. |  |  |  |  |  |  |
|  | B4 Openness in Cross-Cultural Understanding | Learners' interest in understanding and discussing other cultures and the similarities and differences with their local culture through English, coupled with the ability to maintain a critical and open-minded approach during foreign language learning and cross-cultural communication, avoiding blind adoration or rejection of other cultures. | Interviews and Journals, Cross-Cultural Discussion Tasks, Classroom Participation, etc. |  |  |  |  |  |  |
|  | **Are there any indicators that need to be added in this dimension?** | | | | | | | |  |
|  |  | | | | | | | |  |
|  |  |  |  |  |  |  |  |  |  |
| C. Local Expression and Application | C1 Cultural Comparison | Learners' ability to compare local culture with target culture in cross-cultural communication and reflect cultural differences in expression. | Cross-Cultural Discussions, Role-playing, etc. |  |  |  |  |  |  |
|  | C2 Interdisciplinary Knowledge Application | Learners' ability to integrate interdisciplinary knowledge of local culture (such as history, art, geography, etc.) in English expression. | Interdisciplinary Projects, Knowledge Integration tasks, etc. |  |  |  |  |  |  |
|  | C3 Adaptation of Language to Reflect Local Norms | Learners' ability to adjust language expression according to local cultural and social norms in cross-cultural communication. | Role-Playing, Cultural Adaptation Discussions, etc. |  |  |  |  |  |  |
|  | C4 Use of English for Local Storytelling | Learners' ability to freely tell stories related to local culture in English | Oral Narratives, Writing Tasks, etc. |  |  |  |  |  |  |
|  | C5 Participation in Culturally Relevant Discussions | Learners' active participation in English discussions related to local culture in classrooms or other cross-cultural communication settings. | Classroom Observation, Peer Feedback, etc. |  |  |  |  |  |  |
|  | C6 Promotion and Preservation of Local Image | Learners' initiative to promote and preserve local image in cross-cultural communication, showcasing local cultural values through English. | Contextualized Language Tasks, Public Speaking, etc. |  |  |  |  |  |  |
|  | **Are there any indicators that need to be added in this dimension?** | | | | | | | |  |
|  |  | | | | | | | |  |
|  |  |  |  |  |  |  |  |  |  |
|  |  |  |  |  |  |  |  |  |  |
| Other advice: |  | | | | | | | |  |
|  |  |  |  |  |  |  |  |  |  |

**Note**

**Importance Level Key:**

5 - Very Important

4 - Important

3 - Moderately Important

2 - Not Important

1 - Not at All Important
