# Supplementary material for: Assessing local cultural awareness in university EFL learners: A Delphi and AHP-based index framework
Source: PLoS One. 2025 Oct 8;20(10):e0332233. doi: 10.1371/journal.pone.0332233 (PMC12507305; doi:10.1371/journal.pone.0332233)
Supplement: S2 Table — (DOCX) [file pone.0332233.s003.docx]

**S2 Table. Second Delphi round consultation on secondary indicators for EFL learners' local awareness evaluation system.**

| **Primary Indicator** | **Secondary Indicator** | **Description of Secondary Indicator** | **Importance Level** | | | | | **Comments** |
| --- | --- | --- | --- | --- | --- | --- | --- | --- |
|  |  |  | 5 | 4 | 3 | 2 | 1 |  |
|  |  |  |  |  |  |  |  |  |
| A.Local Cognition and Understanding | A1 Local History | Learners engage with and critically analyze key aspects of local history, including significant events, figures, and cultural/societal contexts, through English. This involves understanding and reflecting on historical narratives that are relevant to their own cultural and social experiences, demonstrating both knowledge acquisition and critical thinking in the process. |  |  |  |  |  |  |
|  | A2 Local Scenic Beauty | Learners identify and interpret geographical features (e.g., landscapes, rivers,scenic spots) through English, linking them to local cultural practices, tourism, and community development. |  |  |  |  |  |  |
|  | A3 Local Cultural Practices, Art, and Literature, | Learners' understanding of local cultural customs, traditional festivals, religious beliefs, arts, and literature. |  |  |  |  |  |  |
|  | A4 Local Development Achievements | Learners use English to explore and articulate the significance of local achievements and innovations in various fields, such as science, and technology, and their broader impact on society, etc. |  |  |  |  |  |  |
|  | A5 National Virtues and Qualities | Learners understand and interpret their nation's core values through English, such as resilience, hospitality, and social virtues. |  |  |  |  |  |  |
|  | A6 Daily Life Experiences | Learners use English to describe and discuss aspects of local daily life, such as diet, family structure, education, and work environments, emphasizing their relevance to personal and community experiences, etc. |  |  |  |  |  |  |
|  | A7 Local and Global Issues in Everyday Contexts | Learners demonstrate their ability to use English to understand, discuss, and analyze broader societal issues that influence their daily lives, such as community governance, economic development, environmental challenges, and international relations, with a focus on their practical impact and global relevance. |  |  |  |  |  |  |
|  | A8 Local Language and Dialect Varieties | Learners demonstrate their ability to use English to discuss local language varieties, including dialects or regional expressions, and their connections to Standard English in terms of usage, meaning, and cultural significance. |  |  |  |  |  |  |
|  | A9 Local Ethical and Legal Systems | Learners demonstrate their ability to use English to discuss local ethical principles and legal frameworks, emphasizing their practical role in shaping societal order, resolving conflicts, and guiding everyday behavior within the community. |  |  |  |  |  |  |
|  |  |  |  |  |  |  |  |  |
|  | Are there any indicators that need to be added in this dimension? | | | | | | | |
|  |  | | | | | | | |
|  |  |  |  |  |  |  |  |  |
| B.Local Affective Attitudes | B1 Motivation to Express and Communicate Local Identity | Learners' motivation and willingness to express and disseminate local culture, identity, and values through English learning. |  |  |  |  |  |  |
|  | B2 Emotional Attachment to Local Culture | Learners' emotional attachment and identification with local culture. |  |  |  |  |  |  |
|  | B3 Cultural Pride | Learners' high identification and pride in local culture, and confidence demonstrated in cross-cultural communication. |  |  |  |  |  |  |
|  | B4 Openness in Cross-Cultural Engagement | Learners' ability to engage with other cultures through English while maintaining a balanced perspective—demonstrating openness to cultural diversity and a critical awareness of cultural differences, thereby avoiding uncritical idealization or rejection of foreign cultures. |  |  |  |  |  |  |
|  | Are there any indicators that need to be added in this dimension? | | | | | | | |
|  |  | | | | | | | |
|  |  |  |  |  |  |  |  |  |
| C. Local Expression and Application | C1 Cultural Comparison | Learners' ability to compare local culture with target culture in cross-cultural communication and reflect cultural differences in expression. |  |  |  |  |  |  |
|  | C2 Interdisciplinary Knowledge Application | Learners' ability to integrate interdisciplinary knowledge of local culture (such as history, art, geography, etc.) in English expression. |  |  |  |  |  |  |
|  | C3 Adaptation of Language to Reflect Local Norms | Learners' ability to adjust language expression according to local cultural and social norms in cross-cultural communication. |  |  |  |  |  |  |
|  | C4 Use of English for Local Storytelling | Learners' ability to freely tell stories related to local culture in English |  |  |  |  |  |  |
|  | C5 Promotion and Preservation of Local Image | Learners' initiative to promote and preserve local image in cross-cultural communication, showcasing local cultural values through English. |  |  |  |  |  |  |
|  | Are there any indicators that need to be added in this dimension? | | | | | | | |
|  |  | | | | | | | |
|  |  |  |  |  |  |  |  |  |
| Other advice: |  | | | | | | | |
|  |  |  |  |  |  |  |  |  |

**Note**

**Importance Level Key:**

**5 - Very Important**

**4 - Important**

**3 - Moderately Important**

**2 - Not Important**

**1 - Not at All Important**
