# Supplementary material for: Assessing local cultural awareness in university EFL learners: A Delphi and AHP-based index framework
Source: PLoS One. 2025 Oct 8;20(10):e0332233. doi: 10.1371/journal.pone.0332233 (PMC12507305; doi:10.1371/journal.pone.0332233)
Supplement: S5 Table — (DOCX) [file pone.0332233.s006.docx]

### S5 Table. Operationalization framework for local cultural awareness assessment

| **First-Level Indicator** | **Second-Level Indicator** | **Example Assessment Task** | **Example Assessment Criteria** |
| --- | --- | --- | --- |
| Ⅰ-1 Local Cognition and Understanding | Ⅱ-1 Local History | Create an English poster of "Local History Timeline" with key events, figures, and their cultural impacts labeled. | **Historical English Accuracy** (40%): English descriptions of event timelines and figures' deeds have ≤10% deviation from historical facts, and terminology use complies with basic EFL academic writing standards.  **Cultural English Interpretation** (40%): Clearly expound the influence of historical events on modern local culture in English, with logic conforming to basic English writing structures.  **Presentation Effect** (20%): The English poster features a clear narrative structure, fluent language, and fonts/color schemes in line with English visual habits. |
|  | Ⅱ-2 Local Cultural Practices, Art, and Literature | Adapt local folk songs/legends into English (e.g., translate tea-picking opera lyrics into an English short play). | **Retention of Artistic Elements in English** (30%): The English adaptation preserves core cultural symbols of the original art form and conforms to basic EFL drama script formats.  **Oral Interpretation** (50%): Use relevant academic vocabulary in English interpretation, with clear pronunciation and ≥80% grammatical accuracy.  **Audience Interaction** (20%): Respond to questions in English with complete sentences. |
|  | Ⅱ-3 Local Development Achievements | Create an English report on "Local Development Data Visualization" (e.g., GDP contribution ratio of cultural and creative industries). | **Data Accuracy in English** (30%): Accurate translation of data in the English report (e.g., "GDP contribution ratio"), with local statistical terminology cited.  **Trend Analysis** (40%): Use logical words when analyzing data trends in English.  **Visualization Effect** (30%): Concise and accurate English titles and labels for charts. |
|  | Ⅱ-4 Daily Life Experiences | Film an English micro-video titled "Culture in Daily Life". | **Typicality of Cultural Scenes in English** (30%): English commentary accurately reflects local characteristics, with vocabulary close to everyday English.  **Lens Language** (40%): Natural English dubbing, with concise annotations for dialectal vocabulary.  **Cultural Significance Extraction** (30%): Extract 1-2 cultural elements in English, with complete explanatory sentences. |
|  | Ⅱ-5 National Virtues and Qualities | Host a "Virtue Story Sharing Session" to tell local figures' deeds in English (e.g., anti-epidemic volunteers, inheritors of intangible cultural heritage). | **Authenticity of Stories in English** (30%): No errors in key information of English stories (e.g., figures' identities, event timelines), with correct use of past tense.  **Interpretation of Virtues in English** (40%): Explain the connection between virtues and local culture in English.  **Oral Fluency** (30%): ≤5 hesitations per minute in English speeches, with natural intonation (e.g., stress on key words). |
|  | Ⅱ-6 Local Ethical and Legal Systems | Debate local ethical conflict cases in English. | **Ethical English Citation** (30%): Cite 1-2 local ethical concepts in English.  **Legal Provision Citation** (40%): Accurately cite relevant legal provisions, interpret them in line with legislative intent, and reasonably apply them to solve cases.  **Debate Logic** (30%): Clear English arguments with relevant evidence. |
|  | Ⅱ-7 Regional Geography | Design an English tour route of "Geography-Culture". | **English Association of Geography and Culture** (30%): Simply associate geography with culture in English, using complete sentences.  **Tour Practicality** (40%): Concise English tour guide words without grammatical errors, with route duration ≤2 hours.  **Multilingual Adaptation** (30%): Consistent information between English and Chinese signs, avoiding literal translation errors. |
|  | Ⅱ-8 Local and Global Issues in Everyday Contexts | Host an English debate on "Local Issues in Global Perspective" (e.g., "Ancient Town Tourism Development vs. Cultural Authenticity Protection"). | **Issue Analysis** (30%): Propose 1-2 solutions in English.  **Solution Feasibility** (40%): Solutions are operable (e.g., "phased development"), with ≥70% grammatical accuracy.  **Critical Thinking** (30%): Clearly express personal views in sentences and reasonably analyze and respond to different views. |
|  | Ⅱ-9 Local Language and Dialect Varieties | Create a "Dialect vs. English" comparison manual (e.g., English transliteration of dialectal vocabulary + cultural annotations). | **Transliteration Accuracy** (30%): English transliteration of dialectal vocabulary is close to the pronunciation.  **Annotation Conciseness** (40%): Cultural annotations briefly explain the usage in English.  **Design Practicality** (30%): English comparison cards have clear fonts, corresponding text and images, and are easy to memorize. |
| Ⅰ-2 Local Emotional Attitude (0.3301) | Ⅱ-10 Cultural Pride | Pride Portfolio: Curate 5 cultural artifacts with English reflections addressing: - Personal significance - Historical context - Contemporary relevance | **Affective Authenticity** (40%): Use specific personal anecdotes in English to avoid generic statements.  **Contextualization** (40%): Social-historical links.  **Critical Awareness** (20%): Acknowledge both positive and challenging aspects in English. |
|  | Ⅱ-11 Interest in Cross-Cultural Understanding | Design a "Cultural Exchange Package": Introduce local culture in English and exchange cultural materials with overseas students. | **English Material Richness** (30%): English introduction includes at least 3 cultural dimensions, with vocabulary ≥100 words and no major grammatical errors.  **Interactive Initiative** (40%): Take the initiative to ask questions in English.  **Cross-Cultural Etiquette** (30%): Polite English expression, avoiding sensitive topics. |
|  | Ⅱ-12 Motivation to Express and Communicate Local Identity | Create an English public account for "Local Culture Promotion" and regularly publish cultural stories, event previews, etc. | **English Content Originality** (30%): Original English stories with specific details, and ≥75% grammatical accuracy.  **Community Activity** (40%): Publish ≥1 English article monthly, reply to ≥1 comment per week, and use interactive words.  **Communication Willingness** (30%): Invite others to follow in English, with membership growth ≥3 people/month. |
|  | Ⅱ-13 Emotional Attachment to Local Culture | Write an English diary of "Cultural Memory" to record emotional experiences related to local culture. | **English Emotional Delicacy** (30%): Describe specific emotions in English.  **Memory Uniqueness** (40%): Stories reflect personal connections with culture, with vocabulary ≥80 words per piece.  **Sustained Recording** (30%): 1 piece per week for 4 weeks, with language progress. |
| Ⅰ-3 Local Expression and Application (0.4527) | Ⅱ-14 Use of English for Local Storytelling | "Local Culture Podcast" project: Record English audio stories (e.g., folk legends, entrepreneurial histories of time-honored brands). | **Language Accuracy** (30%): ≤3 grammatical errors per 5 minutes in English audio, with accurate term translation.  **Story Attractiveness** (40%): Narrative with beginning and end, clear sound quality.  **Communication Effect** (30%): Podcast plays ≥50 times on English platforms, with ≥3 listener messages. |
|  | Ⅱ-15 Promotion and Preservation of Local Image | International communication scenario simulation: Clarify cultural misunderstandings. | **Information Clarity** (30%): Accurately identify culturally misunderstood contents and clearly and accurately expound the misunderstood contents and their impacts in English.  **Expression Logic** (40%): Rigorous clarification and refutation logic, clear argumentation, and effective support for views.  **Cross-Cultural Communication Effect** (30%): Fully consider the cultural background of the target audience, adopt appropriate communication strategies, and effectively avoid cultural conflicts. |
|  | Ⅱ-16 Interdisciplinary Knowledge Application | Design an English project of "Culture + X" (e.g., English popular science on "Chemistry - Traditional Dye Formulas"). | **English Disciplinary Integration** (50%): The English project connects culture with 1 discipline, with accurate terminology.  **Popular Science Understandability** (40%): Explain in accessible English.  **Communication Form** (10%): English popular science combines text and images. |
|  | Ⅱ-17 Adaptation of Language to Reflect Local Norms | Rewrite local folk stories for three types of English audiences: children, scholars, and tourists. | **Stylistic Sensitivity** (50%): Significant style transformation.  **Content Adaptability** (40%): Match audience needs.  **Consistency** (20%): Maintain core meaning. |
|  | Ⅱ-18 Cultural Comparison | Create a parallel English glossary to compare ≥5 representative concepts (e.g., "treat") including their contextual usages and behavioral expressions. | **English Concept Selection** (20%): Select representative local cultural concepts in line with EFL teaching priorities.  **Comparison Depth** (50%): English annotations explain contextual differences.  **Practical Value** (30%): The English glossary is useful in cross-cultural interactions, evaluated by teachers for applicability. |
