# Supplementary material for: Assessing local cultural awareness in university EFL learners: A Delphi and AHP-based index framework
Source: PLoS One. 2025 Oct 8;20(10):e0332233. doi: 10.1371/journal.pone.0332233 (PMC12507305; doi:10.1371/journal.pone.0332233)
Supplement: S6 Table — (DOCX) [file pone.0332233.s007.docx]

# S6 Table. Index system with CEFR alignment

| **Primary Indicators** | **Secondary Indicators** | **Descriptor**  **Alignment** |
| --- | --- | --- |
| Ⅰ-1 Local Cognition and Understanding (0.2172) | Ⅱ-1 Local History | B1: (understands straightforward factual info on everyday topics)  B2: (relaying specific information - discusses historical contexts) |
|  | Ⅱ-2 Local Cultural Practices, Art, and Literature | B2: (Mediating Concepts - explains viewpoints on cultural topics)  C1: (analyzes art forms as cultural expressions) |
|  | Ⅱ-3 Local Development Achievements | B2: (Explaining Data - links local achievements to cultural values)  C1: (evaluates impact on cultural identity) |
|  | Ⅱ-4 Daily Life Experiences | B1: (describes daily practices)  B2: (connects daily life to community experiences) |
|  | Ⅱ-5 National Virtues and Qualities | B2: (interprets societal values)  C1: (analyzes virtues in cross-cultural context) |
|  | Ⅱ-6 Local Ethical and Legal Systems | B2: (explains legal frameworks)  C1: (mediates ethical norms in discourse) |
|  | Ⅱ-7 Local Scenic Beauty | B1: (describes geographical features)  B2: (connects landscapes to cultural practices) |
|  | Ⅱ-8 Local and Global Issues in Everyday Contexts | B2: (analyzes societal issues)  C1: (links local challenges to global relevance) |
|  | Ⅱ-9 Local Language and Dialect Varieties | B2: (discusses language varieties)  C1: (compares dialects with Standard English) |
| Ⅰ-2 Local Affective Attitudes (0.3301) | Ⅱ-10 Cultural Pride | B2: (demonstrates cultural confidence) C1: (expresses pride in cross-cultural settings) |
|  | Ⅱ-11 Openness in Cross-Cultural Engagement | B2: (maintains open-minded approach) C1: (critically evaluates cultural similarities/differences) |
|  | Ⅱ-12 Motivation to Express Local Identity | B2: (initiates cultural expression)  C1: (strategizes local identity communication) |
|  | Ⅱ-13 Emotional Attachment to Local Culture | B2: (articulates emotional connection) C1: (integrates emotional attachment into discourse) |
| Ⅰ-3 Local Expression and Application (0.4527) | Ⅱ-14 Use of English for Local Storytelling | B2: (constructs cultural narratives)  C1: (crafts complex storytelling with cultural depth) |
|  | Ⅱ-15 Promotion and Preservation of Local Image | C1: (promotes local image in public discourse)  C2: (strategically preserves cultural identity) |
|  | Ⅱ-16 Interdisciplinary Knowledge Application | B2: (integrates basic interdisciplinary knowledge)  C1: (synthesizes complex interdisciplinary insights) |
|  | Ⅱ-17 Adaptation of Language to Reflect Local Norms | C1: (adjusts language to cultural norms) C2: (masterfully balances linguistic adaptation) |
|  | Ⅱ-18 Cultural Comparison | C1: (compares cultures with critical analysis)  C2: (mediates cultural differences in complex scenarios) |
