# Supplementary material for: Assessing local cultural awareness in university EFL learners: A Delphi and AHP-based index framework
Source: PLoS One. 2025 Oct 8;20(10):e0332233. doi: 10.1371/journal.pone.0332233 (PMC12507305; doi:10.1371/journal.pone.0332233)
Supplement: S7 Table — (DOCX) [file pone.0332233.s008.docx]

# S7 Table. ACTFL-OPI integration

| **ACTFL-OPI**  **Level** | **Cultural**  **Competency**  **Task** | **Cultural Weight (%)** | **Mapped**  **Secondary**  **Indicators** |
| --- | --- | --- | --- |
| Intermediate | Describe local traditions, customs, and daily life | 20% | Ⅱ-1 Local History  Ⅱ-2 Local Cultural Practices, Art, and Literature  Ⅱ-4 Daily Life Experiences  Ⅱ-7 Local Scenic Beauty |
| Advanced | Analyze cultural issues, compare local and global contexts | 30% | Ⅱ-3 Local Development Achievements  Ⅱ-5 National Virtues and Qualities  Ⅱ-6 Local Ethical and Legal Systems  Ⅱ-8 Local and Global Issues in Everyday Contexts  Ⅱ-9 Local Language and Dialect Varieties |
| Superior | Synthesize cultural values, promote local identity in complex contexts | 50% | Ⅱ-10 Cultural Pride  Ⅱ-11 Openness in Cross-Cultural Engagement  Ⅱ-12 Motivation to Express and Communicate Local Identity  Ⅱ-13 Emotional Attachment to Local Culture  Ⅱ-14 Use of English for Local Storytelling  Ⅱ-15 Promotion and Preservation of Local Image  Ⅱ-16 Interdisciplinary Knowledge Application  Ⅱ-17 Adaptation of Language to Reflect Local Norms  Ⅱ-18 Cultural Comparison |
