# Supplementary material for: Assessing local cultural awareness in university EFL learners: A Delphi and AHP-based index framework
Source: PLoS One. 2025 Oct 8;20(10):e0332233. doi: 10.1371/journal.pone.0332233 (PMC12507305; doi:10.1371/journal.pone.0332233)
Supplement: Attachment — Submitted filename: PONE-D-25-18530.pdf [file pone.0332233.s009.pdf]

PLOS ONE

Assessing local cultural awareness in university EFL learners: A Delphi and AHP-based index framework

--Manuscript Draft--

Manuscript Number: PONE-D-25-18530

Article Type: Research Article

Full Title: Assessing local cultural awareness in university EFL learners: A Delphi and AHP-based index framework

Short Title: Assessing local cultural awareness in university EFL learners

Corresponding Author: Lunan Fan
Guizhou Police College
Guiyang, Guizhou Province CHINA

Keywords: Local Cultural Awareness; EFL learners; Delphi method; AHP

Abstract: The aim of this study is to use the Delphi method and the analytical hierarchy process (AHP) to construct a local cultural awareness evaluation index system for English as a foreign language (EFL) learning. Addressing gaps in frameworks for prioritizing English cultural content, the study identified three key dimensions: <i>Local Cognition and Understanding</i>, <i>Local Affective Attitudes</i>, and <i>Local Expression and Application</i>, with the latter being prioritized. Under the primary indicators, secondary indicators like <i>Use of English for Local Storytelling</i>, <i>Promotion and Preservation of Local Image</i>, <i>Cultural Pride</i>, and <i>Openness in Cross - Cultural Engagement</i> were highlighted. The final index system can offer educators and researchers an effective tool for evaluating local cultural awareness in EFL learners, which can inform curriculum development and teaching practices.

Order of Authors:
Lunan Fan
Guiping Cheng
Na Yan

Additional Information:

Question	Response
**Financial Disclosure**<br><br>Enter a financial disclosure statement that describes the sources of funding for the work included in this submission. Review the submission guidelines for detailed requirements. View published research articles from *PLOS ONE* for specific examples.<br><br>This statement is required for submission and **will appear in the published article** if the submission is accepted. Please make sure it is accurate.	Yes

**Funded studies**<br>Enter a statement with the following details:<br>• Initials of the authors who received each award<br>• Grant numbers awarded to each author<br>• The full name of each funder<br>• URL of each funder website<br>• Did the sponsors or funders play any role in the study design, data collection and analysis, decision to publish, or preparation of the manuscript?<br><br>Did you receive funding for this work?	
Please add funding details.<br>as follow-up to "**Financial Disclosure**<br><br>Enter a financial disclosure statement that describes the sources of funding for the work included in this submission. Review the submission guidelines for detailed requirements. View published research articles from *PLOS ONE* for specific examples.<br><br>This statement is required for submission and **will appear in the published article** if the submission is accepted. Please make sure it is accurate.<br><br>**Funded studies**<br>Enter a statement with the following details:<br>• Initials of the authors who received each award<br>• Grant numbers awarded to each author<br>• The full name of each funder<br>• URL of each funder website<br>• Did the sponsors or funders play any role in the study design, data collection and analysis, decision to publish, or preparation of the manuscript?<br><br>Did you receive funding for this work?"	The initials of the authors who received each award: F.L.<br>This study was supported by the "2023 Guizhou Provincial Education Science Planning Project and Yue-Qian Special Project" from the Guizhou Provincial Department of Education. The project is titled "Constructing National Identity in College English through the Integration of Guizhou's Local Cultural Resources" and has been awarded the project number: 2023B035.<br>For further information regarding the funding support, please refer to the following links:<br>Guizhou Provincial Department of Education website:<br>https://jyt.guizhou.gov.cn/index.html<br>Announcement of the project establishment:<br>https://jyt.guizhou.gov.cn/xwzx/tzgg/202311/t20231113_83074270.html<br>The funding body was primarily involved in the release of the project guidelines and the selection of high-quality projects during the establishment process.
Please select the country of your main research funder (please select carefully as in some cases this is used in fee calculation).	CHINA - CN

as follow-up to "**Financial Disclosure**<br><br>Enter a financial disclosure statement that describes the sources of funding for the work included in this submission. Review the submission guidelines for detailed requirements. View published research articles from *PLOS ONE* for specific examples.<br><br>This statement is required for submission and **will appear in the published article** if the submission is accepted. Please make sure it is accurate.<br><br>**Funded studies**<br>Enter a statement with the following details:<br>• Initials of the authors who received each award<br>• Grant numbers awarded to each author<br>• The full name of each funder<br>• URL of each funder website<br>• Did the sponsors or funders play any role in the study design, data collection and analysis, decision to publish, or preparation of the manuscript?<br><br>Did you receive funding for this work?"	
**Competing Interests**<br><br>Use the instructions below to enter a competing interest statement for this submission. On behalf of all authors, disclose any competing interests that could be perceived to bias this work—acknowledging all financial support and any other relevant financial or non-financial competing interests.<br><br>This statement is required for submission and **will appear in the published article** if the submission is accepted. Please make sure it is accurate and that any funding sources listed in your Funding Information later in the submission form are also declared in your Financial Disclosure statement.	The authors have declared that no competing interests exist.

View published research articles from *PLOS ONE* for specific examples.<br><br>**NO authors have competing interests**<br><br>Enter: *The authors have declared that no competing interests exist.*<br><br>**Authors with competing interests**<br><br>Enter competing interest details beginning with this statement:<br><br>*I have read the journal's policy and the authors of this manuscript have the following competing interests: [insert competing interests here]*<br><br>* typeset	
**Ethics Statement**<br><br>Enter an ethics statement for this submission. This statement is required if the study involved:<br><br>• Human participants<br>• Human specimens or tissue<br>• Vertebrate animals or cephalopods<br>• Vertebrate embryos or tissues<br>• Field research<br><br>Write "N/A" if the submission does not require an ethics statement.<br><br>General guidance is provided below. Consult the submission guidelines for detailed instructions. **Make sure that all information entered here is included in the Methods section of the manuscript.**	Human Subject Research (involving human participants)<br><br>The name of the institutional review board：Institutional Review Board of Guizhou Police College<br><br>The approval number:GZPC20231127<br><br>Participants volunteered to fill out the questionnaire. Before filling out the questionnaire, the purpose of the questionnaire was informed to them, and it was made clear that submitting the questionnaire would be regarded as informed consent. Participants could withdraw from the questionnaire - filling process at any time.

Powered by Editorial Manager® and ProduXion Manager® from Aries Systems Corporation

**Format for specific study types**<br><br>**Human Subject Research (involving human participants and/or tissue)**<br>• Give the name of the institutional review board or ethics committee that approved the study<br>• Include the approval number and/or a statement indicating approval of this research<br>• Indicate the form of consent obtained (written/oral) or the reason that consent was not obtained (e.g. the data were analyzed anonymously)<br><br>**Animal Research (involving vertebrate animals, embryos or tissues)**<br>• Provide the name of the Institutional Animal Care and Use Committee (IACUC) or other relevant ethics board that reviewed the study protocol, and indicate whether they approved this research or granted a formal waiver of ethical approval<br>• Include an approval number if one was obtained<br>• If the study involved *non-human primates*, add *additional details* about animal welfare and steps taken to ameliorate suffering<br>• If anesthesia, euthanasia, or any kind of animal sacrifice is part of the study, include briefly which substances and/or methods were applied<br><br>**Field Research**<br><br>Include the following details if this study involves the collection of plant, animal, or other materials from a natural setting:<br>• Field permit number<br>• Name of the institution or relevant body that granted permission	
**Data Availability**<br><br>Authors are required to make all data underlying the findings described fully available, without restriction, and from the time of publication. PLOS allows rare exceptions to address legal and ethical concerns. See the PLOS Data Policy and FAQ for detailed information.	Yes - all data are fully available without restriction

A Data Availability Statement describing where the data can be found is required at submission. Your answers to this question constitute the Data Availability Statement and **will be published in the article**, if accepted.<br><br>**Important:** Stating 'data available on request from the author' is not sufficient. If your data are only available upon request, select 'No' for the first question and explain your exceptional situation in the text box.<br><br>Do the authors confirm that all data underlying the findings described in their manuscript are fully available without restriction?	
**Describe where the data may be found in full sentences. If you are copying our sample text, replace any instances of XXX with the appropriate details.**<br><br>• If the data are **held or will be held in a public repository**, include URLs, accession numbers or DOIs. If this information will only be available after acceptance, indicate this by ticking the box below. For example: *All XXX files are available from the XXX database (accession number(s) XXX, XXX.*).<br>• If the data are all contained **within the manuscript and/or Supporting Information files**, enter the following: *All relevant data are within the manuscript and its Supporting Information files.*<br>• If neither of these applies but you are able to provide **details of access elsewhere**, with or without limitations, please do so. For example:<br><br>*Data cannot be shared publicly because of [XXX]. Data are available from the XXX Institutional Data Access / Ethics Committee (contact via XXX) for researchers who meet the criteria for access to confidential data.*<br><br>*The data underlying the results presented in the study are available from (include the name of the third party*	All relevant data are within the manuscript and its Supporting Information files.

and contact information or URL).<br>• This text is appropriate if the data are owned by a third party and authors do not have permission to share the data.<br><br>* typeset	
Additional data availability information:	Tick here if the URLs/accession numbers/DOIs will be available only after acceptance of the manuscript for publication so that we can ensure their inclusion before publication.; Tick here if your circumstances are not covered by the questions above and you need the journal's help to make your data available.

Manuscript
Click here to access/download;Manuscript;Assessing local cultural awareness.docx

Assessing local cultural awareness in university EFL learners: A Delphi and AHP-based index framework

Fan Lunan[1] , Cheng Guiping [2], Yanna[3]

1 Department of Theoretical Education, Guizhou Police College, Guiyang, Guizhou Province, China, 2 Department of Theoretical Education, Guizhou Police College, Guiyang, Guizhou Province, China,3 School of Foreign Languages, Guizhou Education University, Guiyang, Guizhou Province, China

Abstract

The aim of this study is to use the Delphi method and the analytical hierarchy process (AHP) to construct a local cultural awareness evaluation index system for English as a foreign language (EFL) learning.Addressing gaps in frameworks for prioritizing English cultural content, the study identified three key dimensions: *Local Cognition and Understanding*, *Local Affective Attitudes*, and *Local Expression and Application*, with the latter being prioritized. Under the primary indicators, secondary indicators like *Use of English for Local Storytelling , Promotion and Preservation of Local Image*, *Cultural Pride*, and *Openness in Cross-Cultural Engagement* were highlighted. The final index system can offer educators and researchers an effective tool for evaluating local cultural awareness in EFL learners, which can inform curriculum development and teaching practices.

Introduction

In today's globalized world, English proficiency is regarded as an essential skill for communication across borders. English as a Foreign Language (EFL) programs are increasingly designed not only to equip learners with language proficiency but also to develop their ability to engage with the global community. Learners face "Cultural Aphasia", a term describing their struggle to articulate local identities through foreign language discourse. This linguistic-cultural disjuncture has catalyzed reforms in EFL curricula, where there is growing recognition of the importance of integrating localized content into language learning to make it more relevant and meaningful to learners' lived experiences [1, 2].

Although the advantages of integrating local elements into the EFL curriculum have gained more attention, it is still challenging to effectively assess how learners exercise their agency and demonstrate local identity through their plurilingual competence in English language education.[3]. At present, most of the evaluation systems excessively focus on the assessment of language skills and cross-cultural communication ability. For example, frameworks such as the CEFR are effective in benchmarking grammatical accuracy. However, they inadequately capture learners' ability to articulate culturally - grounded narratives. This lack of assessment is particularly prominent in areas with significant cultural diversity, because the local cultural characteristics profoundly affect the actual application scenarios of language.

Given the importance of evaluating local cultural awareness in EFL learning, this study aims to develop a comprehensive evaluation index system. And the system will focus on three key dimensions: cognitive, affective, and behavioral. Such dimensions have been identified in previous research as critical components of language learning and intercultural competence [4, 5]. In particular, the cognitive dimension will measure learners' understanding of local cultural, social, and historical contexts, and their ability to express this knowledge in English. The affective dimension will evaluate learners' attitudes toward expressing local content, including their emotional connection to their region and their motivation to share local stories. Finally,the behavioral dimension mainly focuses on how learners use English to express and spread local culture in practical communication.

The evaluation system constructed by this research will serve as a valuable resource for educators, enabling them to evaluate how well learners can interact with and represent their local culture when using English. The goal is to promote a comprehensive approach to language learning that integrates both global and local perspectives, fostering learners who can confidently and skillfully interact within diverse cultural contexts.

Literature Review

Local Cultural Awareness in EFL Learning

Local cultural awareness in EFL learning refers to the conscious integration of learners' local context into the language learning process. The idea of embedding local content in EFL curricula stems from the understanding that language learning should not be isolated from the socio-cultural background.[1, 7]. Rooted in sociocultural theory from Vygotsky [8], local cultural consciousness emphasizes the importance of situational sensitivity. Educators need to be able to recognize and respond to the complex interactions within the sociocultural context in which learners are situated, such as language use, historical background, and community connections. By utilizing cultural symbols and designing collaborative learning activities, educators can help students integrate theoretical knowledge with their local experiences, forming a meaningful knowledge network. This approach aims to foster the co-development of students' cognitive growth, cultural identity, and critical thinking skills [9]. Tomlinson [10] conceptualizes it as a dynamic metacognitive skill that enables learners to "notice, analyze, and negotiate linguistic hybridity" , such as code-switching patterns in multilingual communities. This construct extends beyond passive cultural literacy.

The theoretical framework of local cultural awareness is also rooted in other disciplines. Under the model of cultural ecology, Kramsch [11] put forward the concept of "third space", believing that EFL classroom should be a place for

dynamic negotiation between local culture and global culture. From the perspective of intercultural communication theory, Byram [4] "Intercultural competency model" emphasizes that EFL learners need to develop critical reflection by comparing local cultures with target cultures. Integrating research from multilingual studies and sociolinguistics, Canagarajah[12] advocates that multilingual speakers incorporate their own languages and values into English. This integration initiates various negotiation strategies that assist them in interpreting other distinct varieties of English and in constructing new norms.

Cognitive and Interdisciplinary Demands in Tertiary-Level EFL Education

English plays a vital role in cultural integration within foreign language education, making cultural inclusion in TEFL instruction critically important [13]. Contemporary university-level EFL teaching requires combining higher-order thinking (HOT) with cross-disciplinary cognitive skills. Advanced learners at this level must move beyond basic language mastery to demonstrate analytical abilities for processing complex, multi-subject content [14]. Integrating HOT into curricula pushes students past memorization, developing their critical analysis, problem-solving, and information synthesis across disciplines [2,10]. Learners tackle interdisciplinary challenges in English, demanding knowledge integration from multiple fields to form sophisticated responses [15]. For example, Chubko et al.[16] showed how digital storytelling (DST) improves STEM literacy by merging language skills with technical

knowledge and social identity expression, proving cross-subject methods' value in EFL contexts.

Moreover, studies in Content and Language Integrated Learning (CLIL) confirms HOT and interdisciplinary skills' necessity for advanced EFL education. CLIL approaches emphasize the teaching of both content and language simultaneously. By encouraging learners to use English as a medium to acquire knowledge from subjects such as history, geography, and science, both language proficiency and cognitive development of EFL learners was strengthened [1,18]. Complex cognitive skills such as critical analysis, reflection, and synthesis are required,building competencies that correspond to modern educational goals.

Benefits of Local Content Integration in EFL Instruction

Despite the importance of local content in language learning, EFL programs have traditionally emphasized the cultures of English-speaking countries, which can lead to a disconnect between learners and their local realities. Indonesian junior high textbooks exemplify this issue, prioritizing Western cultural references while neglecting regional traditions [1]. This imbalance leaves students more familiar with foreign customs than their own, limiting discussions about local values. Such detachment frames English as an external code rather than a meaningful communication tool, potentially reducing motivation and hindering acquisition [20].

Emerging studies highlight local culture integration's positive impacts on EFL outcomes. Kanok-Xirapathum and Chanu [1] observed Thai students responding positively to lessons incorporating community content. Gay [22] emphasized that culturally relevant teaching strengthens language skills by validating learners' backgrounds. These findings demonstrate how localized approaches enhance engagement, motivation, and proficiency. Emotional benefits are also evident, as students demonstrate greater motivation when local culture is integrated into learning, as shown by Aminullah et al. [23] in Indonesia and Gashi [24] in Kosovo. Porto [2] observed that local cultural narratives in Argentine English curricula improve language skills and foster cultural pride. From a cognitive perspective, Kramsch [25] emphasized the inseparability of language and culture, noting that language learning is not only about acquiring linguistic forms but also about understanding cultural meanings, which deepens the cognitive process of language acquisition.

Moreover, research indicates that integrating local content enhances intercultural competence and practical language skills. Canagarajah [12] holds that navigating both local and global cultures through English enhances learners' communicative competence, while Juan-Garau and Jacob [26] show that when learners engage with real-world, culturally relevant scenarios, they develop practical communication skills. Wu and Miller [27] demonstrated that Chinese EFL learners improved intercultural skills by using WeChat to discuss regional festivals and traditions. Their study showed how digital platforms can connect language

practice with cultural knowledge application. Gu and Lee [28] highlight the need to blend international and local viewpoints in teaching. This dual focus allows students to apply English meaningfully in both global exchanges and community contexts. These collective findings confirm that effective EFL instruction requires balancing worldwide communication needs with culturally rooted learning experiences.

Gaps in Current Assessment Frameworks

Although integrating local content into EFL curricula shows clear benefits, measuring learners' cultural engagement through English remains challenging. Widely used evaluation frameworks, such as the Common European Framework of Reference for Languages (CEFR), focus primarily on assessing linguistic proficiency and intercultural competence but do not adequately address the assessment of local cultural awareness [29]. For example, the CEFR's "can do" description focuses on specific language tasks that learners can accomplish at different language levels. While these standards partially address cross-cultural awareness, they lack tools to evaluate how students articulate their cultural identities in English [1].

This limitation extends to other widely adopted frameworks. The ACTFL Proficiency Guidelines, while emphasizing task-based performance in real-world contexts, primarily measures learners' ability to "describe local cultural practices" as a sub-skill of interpersonal communication, without establishing explicit criteria for evaluating the depth or authenticity of cultural identity expression [30]. Similarly,

UNESCO's Global Citizenship Education Framework (2015), though containing a socio-emotional domain that addresses "sense of belonging to local communities," lacks operationalized indicators for assessing how learners articulate their cultural roots through language [31].

Even frameworks explicitly incorporating cultural dimensions exhibit gaps. China's Standards of English Ability (CSE) mandate "introducing Chinese traditional culture in English" as a competence requirement, but its assessment rubrics remain anchored in linguistic accuracy rather than cultural authenticity [32]. The German National Educational Standards, while integrating intercultural competence as a curricular component, have not adequately addressed the assessment of local cultural awareness. Existing assessment frameworks predominantly focus on the comparison of cultural differences and the understanding of target cultures, neglecting the evaluation of learners' awareness and expression of their own local culture [33].

These gaps stem from two structural limitations in current frameworks. Firstly, most separate linguistic competence from cultural identity assessment. For instance, CEFR's pluricultural competence descriptors (e.g., "mediating cultural ambiguities") remain isolated from language production scales. Secondly,frameworks like CEFR measure adaptability to external cultures but fail to capture the dynamic process of reconstructing local identity through L2 [34] .

Recent attempts to address these gaps through CEFR companion volumes show limited progress. While introducing "mediation scales" for cultural interpretation, they still lack descriptors for self-referential cultural expression. As McNamara [35]

critically notes, "The dominant frameworks reduce culture to a set of learnable behaviors rather than a lived identity that interacts with language acquisition" .

To address this gap, this study aims to establish a comprehensive evaluation index system through a series of surveys using the Delphi method, allowing researchers to analyze the opinions of expert panels by collecting, combining and quantifying multiple opinions on complex issues. By creating a tailored assessment system, this study aims to contribute to the growing body of literature on English learning by providing educators with a tool to assess learners' ability to integrate into local culture through English. This framework will enable educators to assess not only language proficiency, but also the learners' ability to use English as a medium to express their local identity. Ultimately, the goal is to promote a more comprehensive approach to language learning that values both global and local perspectives and fostering learners who have the confidence and ability to navigate different cultural environments.

Methods

Participants

In accordance with the Delphi method guidelines[36] that suggest a sample size of 15-20 experts for consensus - driven studies, 15 EFL educators (**Table 1**) were invited to participate in the study and formed the expert panel. The recruitment period for this study spanned from November 30, 2023, to March 1,

2024. These 15 participants were recruited from a variety of academic institutions across China, which guaranteed geographical and institutional diversity. All participants provided written informed consent, and the study was approved by the Institutional Review Board of GPC College (GZPC20231127).

The expert panel was composed of EFL specialists who were required to meet the following three selection criteria: (1) a minimum of 10 years' experience in EFL curriculum design; (2) research conducted in the field of EFL; and (3) holding senior professional titles such as Professor or Associate Professor. This rigorous selection process ensured that the panel's input was both authoritative and relevant to the study's objectives.

Table 1. Demographic Characteristics of Expert Participants (N=15)

Category	Subgroup	n	Category	Subgroup	n
**Age**	≤35 years	4	**Research experience[a]**	Extensive	9
	36-45 years	9		Moderate	5
	46-55 years	2		Limited	1
**Years of EFL teaching experience**	10-19 years	13	**Teaching experience[b]**	Extensive	11
	20-30 years	2		Moderate	4
**Highest degree**	Ph.D.	13	**Current teaching position**	Full Professor	10
	Master's	2		Associate Professor	5

[a] Research experience criteria: Extensive (≥10 publications), Moderate (5-9), Limited (<5).

[b] Teaching experience criteria: Extensive (≥10 years), Moderate (5-9), Limited (<5).

Preliminary Establishment of Indicators

To comprehensively and systematically assess the awareness of native culture among advanced foreign language learners, this study adopted a multi-method approach to construct an initial set of indicators. First, researchers searched the Web of Science database using keywords such as "foreign language native awareness," "cultural competence assessment," and "place-based pedagogy." This search aimed to identify existing literature relevant to the topic, providing a solid theoretical foundation for the establishment of indicators. Concurrently, two rounds of focus group discussions were conducted with the research team members (all front-line foreign language teachers at universities) to clarify concepts, perform redundancy checks, ensure domain coverage, and verify operational feasibility. These steps are crucial for refining the initial set of indicators, ensuring that the selected indicators are not only conceptually sound but also practically feasible and applicable in the context of assessing the awareness of native culture among advanced foreign language learners.

The assessment framework was grounded in a tripartite model of cultural competence [4, 37], which posits cultural competence as a synthesis of cognitive, affective, and behavioral dimensions. The selection of this model is justified by its holistic capacity to capture both internal development (e.g., knowledge and attitudes) and external manifestations (e.g., communicative behaviors) of cultural awareness, a

duality critical for evaluating EFL learners' ability to navigate local-global identity tensions [38]. Specifically, the cognitive dimension focuses on learners' knowledge and understanding of local cultural, social, and historical contexts and their ability to express this knowledge in English, as measured through indicators such as cultural schema activation and contextualized language production [1,39]. The affective dimension, in turn, targets learners' attitudes toward local-global hybridity, with affective indicators measuring their interest in using English as a tool for self-expression and their pride in local cultural narratives [40]. Finally, the behavioral dimension assesses how learners utilize their knowledge of local content in practical settings, highlighting adaptive communication strategies that conform to local linguistic standards and social customs.[41]. Cultural understanding, attitude formation, and adaptive communication define key dimensions. They serve as organizing principles for categorizing indicators.Furthermore, the tripartite structure aligns with contemporary pedagogical goals of fostering not only linguistic proficiency but also intercultural citizenship, enabling learners to act as mediators between their local context and global discourse [2].

Guided by the theoretical synthesis, a preliminary evaluation system comprising three primary indicators was developed. Underpinning these primary indicators, 24 secondary indicators were formulated for the initial consultation phase (S1Table). Specifically, under the *Local Cognition and Understanding* primary indicator, there are 14 secondary indicators including *Local History*, *National and Regional Geography*, and *Local Cultural Practices, Art, and Literature*; under the *Local*

Affective Attitudes primary indicator, there are 4 secondary indicators including *Motivation to Express and Communicate Local Identity*, *Cultural Pride*, and *Oneness in Cross-Cultural Understanding*; under the *Local Expression and Application* indicator, 6 secondary indicators were initially proposed, such as *Cultural Comparison Skill*, *Contextualized Language Use*, and *Promotion and Preservation of Local Image*.

Procedures

In this study, three rounds of expert consultations (**S 1** Fig.) were conducted via email.

Delphi Procedure. The Delphi method was used to gather expert opinions on the key indicators for assessing local cultural awareness in EFL learners. The method involved organizing several rounds of questionnaires to solicit feedback from experts in the field of language education, shown in **S 1** Fig. Through iterative surveys and feedback processes, consensus on specific evaluation indicators was sought. Anonymity in the process allowed for more candid expert feedback, while the statistical analysis provided insight into the central tendencies and variances in expert opinions. These characteristics make the Delphi method particularly effective for educational research where diverse expert perspectives are required [36].

In the first round, opinions were collected from experts on the necessity of the three primary indicators and 24 secondary indicators. The analysis involved descriptive statistics, including the calculation of the mean (M) and standard deviation (SD) to validate initial indicators. This survey lasted for about 4 weeks, during which experts were asked to use a 5-point scale to evaluate each indicator, ranging from "not

important" to "very important". In this round of evaluation, participants also rated their familiarity with evaluation criteria (Cs) and described their judgment rationale (Ca). The basis for judgment (Ca) includes practical experience, logical reasoning, and an understanding of domestic and international language education trends. These self-assessments help ensure that the feedback provided is based on a well-informed perspective. The formula for calculating the expert authority coefficient Cr is: Cr = (Ca + Cs) / 2. It is generally believed that when the expert authority coefficient Cr is greater than or equal to 0.7 [42], the research results are considered reliable. The assignments for Ca and Cs are shown in **Table 2** and

Table 3 respectively.

Table 2 Assignment for Expert Judgment Basis (Ca)

**Judgment Basis**	**Impact Level**		
	High Impact	Moderate Impact	Low Impact
Deductive Reasoning	0.3	0.2	0.1
Practical Experience	0.5	0.4	0.3
Understanding of Domestic and International Language Education Trends	0.1	0.1	0.1
Intuition	0.1	0.1	0.1
Total	1.0	0.7	0.6

Table 3. Familiarity Coefficient Assignment (Cs)

**Familiarity Level**	**Coefficient (Cs)**
Extremely Familiar	1.0
Very Familiar	0.75
Moderately Familiar	0.50
Slightly Familiar	0.25
Not Familiar	0.00

The second round focused on revising and refining the indicators based on the feedback from the first round. A revised version of the assessment system was created for this round. After collecting and analyzing the feedback from the first round, indicators considered irrelevant or meaningless were removed. Researchers, based on the degree of concentration and coordination of expert opinions, re-sorted the remaining indicators and revised the content of the consultation questionnaire to ensure its accuracy and applicability. Indicators with low consensus ($M < 3.5$, $SD > 1.0$, $CV > 25\%$) were eliminated to ensure a higher level of agreement among experts.

Analytic Hierarchy Process (AHP). The final round was dedicated to confirming consensus among experts and constructing the Analytic Hierarchy Process (AHP) hierarchy. The AHP was employed to assign weights to the various indicators of local cultural awareness based on expert judgment. By breaking down the complex task of evaluating learners' local cultural awareness into multiple hierarchical levels, AHP allows for precise pairwise comparisons of factors such as Local Cognition and Understanding, Local Affective Attitudes, and Local Expression and Application. These comparisons helped determine the relative importance of each indicator within the overall evaluation framework. The use of the Saaty 1~9 scale [43] in AHP provided a structured approach for experts to rate the importance of each indicator, ensuring that the weight distribution reflects the collective expert judgment.

To ensure the reliability of the pairwise comparisons, the consistency ratio (CR) was calculated using the formula $CR = CI/RI$, where CI is the consistency index and RI is the random consistency index. According to Saaty's[43] criteria, a CR value less

than 0.1 indicates acceptable consistency. This verification step ensures that the judgments made by experts are logically consistent and free from significant biases or errors.

Results

Expert Authority Analysis

As shown in **Table 1**, the participants all have a high educational background, 87% held doctoral degrees with mean experience of 15.9 years (SD=4.01). Rich experience in university foreign language teaching, and good professional representation, which can provide valuable information for the structural rationality of the construction of the index system.

In the three rounds of discussion and consultation, 15 experts all participated in the whole consultation process, and the recovery rate of the questionnaire was 100%, showing the high participation and input of the participants, and providing a strong support for the promotion of the research. At the same time, the participants conducted self-evaluation on the judgment basis of the problem and the familiarity with the problem. After calculation, the expert authority coefficient (Cr) was 0.867, indicating that the experts had a high degree of familiarity with the indicators and a high authority in the judgment of the indicators, and the consultation results have practical reference significance [44]. The authority coefficient of the experts is shown in **Table 4**.

Table 4. Authority Coefficients of 15 Experts

Expert Number	Familiarity Coefficient (Cs)	Basis of Judgment (Ca)	Authority Coefficient (Cr)
1	1.00	1.00	1.000
2	0.75	0.90	0.825
3	0.75	1.00	0.875
4	1.00	0.90	0.950
5	1.00	0.70	0.850
6	0.50	0.90	0.700
7	1.00	0.70	0.850
8	0.75	1.00	0.875
9	0.75	0.90	0.825
10	1.00	1.00	1.000
11	0.75	0.70	0.725
12	0.75	1.00	0.875
13	0.75	1.00	0.875
14	1.00	0.90	0.950
15	0.75	0.90	0.825
Mean	0.83	0.90	0.867

Notes:

1. Cr is calculated as Cr=(Cs+Ca)/2.
2. The mean Cr value is derived from precise calculations (retaining four decimal places) before rounding.
3. A Cr value ≥ 0.7 indicates high reliability of expert opinions.

Consensus Development Analysis

To assess the degree of agreement among the experts, the Kendall coordination coefficient (W) was calculated. This non-parametric statistic was used to measure the consistency of a group of judges or raters on the items they ranked. The Kendall's W coefficient and chi-square tests revealed significant consensus improvement across Delphi rounds (**Table 5**).

Table 5. Expert Consensus Analysis Across Delphi Rounds

Round	Experts (n)	Indicators (k)	Kendall's W	χ^2(df)	p-value	Interpretation

1	15	24	0.634	267.63 (23)	<0.001	Moderate consensus
2	15	19*	0.855	217.9 (18)	<0.001	Strong consensus

Notes:
1.*5 indicators eliminated after Round 1 screening
2.Degrees of freedom (df) calculated as df=k−1.

The moderate consensus (W=0.634) in Round 1 reflects expected divergence during initial indicator exploration. The significant χ^2 value ($p<0.001$) nevertheless confirms non-random agreement patterns, warranting progression to refinement rounds [45].

The second round of expert consultation yielded a Kendall's coordination coefficient W=0.855 and a p-value of <0.001. According to prior studies, a Kendall's W value between 0.6 and 0.8 [46] typically indicates strong consensus among experts. While the observed value (0.855) slightly exceeds this range, it further demonstrates an exceptionally high level of agreement among participants in the second round, confirming the reliability of the refined results. Notably, the W coefficient increased by 34.8% (from 0.634 in Round 1 to 0.855 in Round 2), demonstrating the Delphi method's efficacy in reconciling expert perspectives through iterative, structured feedback. This significant improvement highlights the robustness of the consensus-building process in narrowing divergent opinions and achieving actionable outcomes.

First Delphi survey results

In the first round of the Delphi survey, 15 experts evaluated a preliminary set of

24 secondary indicators across three primary dimensions (Cognitive, Affective, Behavioral). Statistical thresholds (Mean M ≥ 3.5, Standard Deviation SD < 1, Coefficient of Variation CV < 0.25) were applied to assess indicator necessity. As is shown in

Table 6, 17 indicators (M_{range}=3.87-5.00, SD_{range}=0.41-0.83, CV_{range}=0.08-0.21) met all criteria and were retained for the second round.

Table 7 shows that 7 indicators failed to meet thresholds, primarily due to low relevance (M<3.12)or high opinion divergence(SD>1.01).

Table 6. Retained Indicators After First Delphi Round (n=17)

Indicator Name	Mean (M)	SD	CV
Local History	4.47	0.74	0.17
Local Ethical and Legal Systems	4.07	0.59	0.15
Regional Geography	3.87	0.83	0.22
Local Cultural Practices, Art, and Literature	4.80	0.56	0.12
Local Development Achievements	4.67	0.62	0.13
National Virtues and Qualities	4.80	0.41	0.09
Daily Life Experiences	4.67	0.49	0.10
Local Language and Dialect Varieties	3.87	0.52	0.13
Motivation to Express and Communicate Local Identity	4.80	0.41	0.09
Emotional Attachment to Local Culture	4.13	0.64	0.15
Cultural Pride	5.00	0.00	0.00
Openness in Cross-Cultural Engagement	5.00	0.00	0.00
Cultural Comparison	4.00	0.65	0.16
Interdisciplinary Knowledge Application	4.67	0.49	0.10
Adaptation of Language to Reflect Local Norms	4.07	0.88	0.22
Use of English for Local Storytelling	5.00	0.00	0.00
Promotion and Preservation of Local Image	4.60	0.63	0.14

Table 7. Unretained / Modified Indicators After First Delphi Round (n=7)

Indicator Name	Mean (M)	SD	CV	Action Taken
Local Political System and Governance	1.40	0.63	0.45	Removed
Local Economic System	3.07	0.70	0.23	Removed
Local Social Issues	2.88	1.06	0.37	Integrated into a new indicator
International Relations and Global Issues	2.93	0.80	0.27	Removed
Local Educational System	3.13	1.25	0.40	Removed
Local Environment and Sustainable Development	3.00	0.93	0.31	Merged into A4 Local Development Achievements
Participation in Culturally Relevant Discussions	2.27	0.70	0.31	Removed

Removal of Redundant Indicators: *Local Political System and Governance* (M = 1.40) and *Participation in Culturally Relevant Discussions* (M = 2.27) were excluded due to low relevance and measurement challenges.

Revisions Based on Feedback. *Local Economic System* (M=3.07), *Local Social Issues* (M=2.88), *International Relations and Global Issues* (M=2.93) and *Local Educational System* (M=3.13) were advised to consolidate into a new indicator "Local and Global Issues in Everyday Contexts" under the primary indicator *Local Cognition and Understanding*, in order to focus on practical topics (e.g., community governance, local economic challenges). *Environment and Sustainable Development* (M=3.00)

were suggested to merge into then indicator *Local Development Achievements*.

Descriptive Adjustments. *Cognition of National and Regional Geography* was renamed *Cognition of Local Scenic Beauty* to emphasize cultural connections (e.g., linking landscapes to festivals). At the same time, several experts recommended revising the descriptions of the indicators related to local history, as well as the ethical and legal systems. These revisions aim to align more closely with the objectives of EFL education and to ensure that the assessment is both practical and operational.

The revised indicator set was advanced to the second Delphi round for further validation.

Second Delphi survey results

The second Delphi survey (S2Table) assessed expert consensus on the appropriateness of 18 indicators across three dimensions. All indicators met retention criteria ($M \geq 3.5$, $SD < 1$, $CV < 0.25$), with mean values ranging from 3.93 to 5.00, standard deviations ranging from 0.00 to 0.45, and coefficients of variation ranging from 0.08 to 0.09, confirming high agreement among experts. The results are presented below, organized by dimension.

Local Cognition and Understanding. Experts exhibited strong consensus on indicators under this dimension (**Table 8**). Two indicators—*Local Cultural Practices, Art, and Literature* and *National Virtues and Qualities*—achieved perfect scores (M = 5.00, SD = 0.00, CV = 0.00), reflecting unanimous agreement on their necessity for

evaluating learners' cultural knowledge. Notably, Daily Life Experiences (M = 5.00, SD = 0.00) and Local Development Achievements (M=4.93, SD=0.26) also demonstrated high reliability (CV < 0.05), highlighting their role in connecting abstract cultural concepts to tangible contexts.

Table 8. Two-Round Delphi Survey Results for Secondary Indicators under the Primary Indicator Local Cognition and Understanding

Secondary Indicator	Mean (out of 5)		SD		CV		Action Taken
	1st	2nd	1st	2nd	1st	2nd	
Local History	4.47	4.93	0.74	0.26	0.17	0.05	Retained
Local Scenic Beauty	3.87	3.93	0.83	0.26	0.22	0.07	Retained
Local Cultural Practices, Art, and Literature	4.80	5.00	0.56	0.00	0.12	0.00	Retained
Local Development Achievements	4.67	4.93	0.62	0.26	0.13	0.05	Retained
National Virtues and Qualities	4.80	5.00	0.41	0.00	0.09	0.00	Retained
Daily Life Experiences	4.67	5.00	0.49	0.00	0.10	0.00	Retained
Local Language and Dialect Varieties	3.87	3.93	0.52	0.26	0.13	0.07	Retained
Local Ethical and Legal Systems	4.07	4.73	0.59	0.46	0.15	0.10	Retained
Local and Global Issues in Everyday Contexts	–	3.93	–	0.26	–	0.07	Retained

When looking at the improved indicators, *Local History* showed significant progress, with its mean increasing from 4.47 (SD = 0.74) in the first round to 4.93 (SD = 0.26) in the second round, and its CV decreasing from 0.17 to 0.05. This improvement suggests that the revised description of the indicator better aligns with EFL learning objectives. Similarly, *Local Ethical and Legal Systems* progressed from

moderate consensus (M = 4.07, CV = 0.15) to higher agreement (M = 4.73, CV = 0.10), indicating enhanced clarity in its operational definition.

Regarding the newly added indicator, *Local and Global Issues* in Everyday Contexts (M = 3.93, SD = 0.26, CV = 0.07) was introduced in the second round to address practical cultural engagement. Although its mean was slightly lower compared to other retained indicators, it met all statistical thresholds, justifying its inclusion in the final evaluation system.

Local Affective Attitudes. As is shown in

Table 9, the appropriateness of indicators related to the "emotion" dimension as reflected by experts in two rounds of surveys is shown in Table 8. All affective indicators received strong endorsement.

Cultural Pride (M = 5.00, SD = 0.00, CV = 0.00) and *Critical Openness in Cross-Cultural Engagement* (M = 5.00, SD = 0.00, CV = 0.00) achieved perfect consensus in both rounds, reflecting unanimous agreement on their critical role in fostering cultural confidence and balanced intercultural perspectives. Emotional *Attachment to Local Culture* maintained a high level of agreement (M = 4.00, SD = 0.38, CV = 0.09) in the second round, though its mean slightly decreased from 4.13 in the first round. The low CV value (0.09) confirms its reliability as a measure of learners' emotional connection to their cultural heritage.

Table 9. **Two-Round Delphi Survey Results for Secondary Indicators under the Primary Indicator Local Affective Attitudes**

Secondary Indicator	Mean (out of 5)		SD		CV		Action Taken
	1st	2nd	1st	2nd	1st	2nd	
Motivation to Express and Communicate Local Identity	4.80	4.93	0.41	0.26	0.09	0.05	Retained
Emotional Attachment to Local Culture	4.13	4.00	0.64	0.38	0.15	0.09	Retained
Cultural Pride	5.00	5.00	0.00	0.00	0.00	0.00	Retained
Critical Openness in Cross-Cultural Engagement	5.00	5.00	0.00	0.00	0.00	0.00	Retained

Local Expression and Application. Table 10 summarizes results of this dimension, demonstrating strong expert consensus on their relevance to assessing learners' ability to express and apply local culture through English.

Two indicators—*Use of English for Local Storytelling* and *Promotion and Preservation of Local Image*—received unanimous support (M = 5.00, SD = 0.00), emphasizing storytelling and advocacy as critical skills for cultural dissemination. Though other indicators show slight fluctuations, the high consensus across indicators (CV < 0.10) validates their collective role in translating cultural knowledge into actionable language skills.

Table 10. **Two-Round Delphi Survey Results for Secondary Indicators under the Primary Indicator Local Expression and Application**

Secondary Indicator	Mean (out of 5)	SD	CV	Action Taken

	1st	2nd	1st	2nd	1st	2nd	
Cultural Comparison	4.00	4.07	0.59	0.26	0.16	0.06	Retained
Interdisciplinary Knowledge Application	4.67	4.93	0.49	0.26	0.10	0.05	Retained
Adaptation of Language to Reflect Local Norms	4.07	4.07	0.88	0.26	0.22	0.06	Retained
Use of English for Local Storytelling	5.00	5.00	0.00	0.00	0.00	0.00	Retained
Promotion and Preservation of Local Image	4.60	5.00	0.63	0.00	0.14	0.00	Retained

On the whole, all the indicators in the second round of Delphi survey received a "Retained" status, confirming that they were deemed relevant and essential by the experts. The retention of all indicators suggests a strong consensus among the experts on the comprehensive nature of the evaluation framework for assessing EFL learners' local cultural awareness. This unanimity demonstrates the importance of including both cognitive, affective, and behavioral dimensions in the assessment, ensuring that learners' understanding, emotional connection, and practical application of local culture are all adequately measured.

Analytic Hierarchy Process (AHP) Weighting Outcomes

The third consultation phase employed the Analytic Hierarchy Process (AHP) to determine the relative weights of indicators within the evaluation system. Following AHP protocol, a hierarchical model (**S 2** Fig.)was constructed based on the finalized primary and secondary indicators of EFL Learners' Local Cultural Awareness. Fifteen experts from the Delphi panel were invited to complete pairwise comparison matrices for the hierarchical structure(S3Table,S4 Table).

The expert evaluations were analyzed using AHP software to calculate eigenvectors, perform hierarchical single ranking, and conduct consistency checks. All four judgment matrices exhibited satisfactory consistency, with consistency ratios (CR) below the threshold of 0.1 and consistency indices (CI) approaching zero (**Table 11**). These results confirm the logical coherence of expert judgments and validate the weight assignments.

Table 11. AHP Consistency Metrics

Hierarchy Level	λmax	CI	CR
Primary Dimensions	3.1008	0.0504	0.0970
Expression Sub-criteria	5.3500	0.0875	0.0781
Affective Sub-criteria	4.2364	0.0788	0.0886
Cognitive Sub-criteria	9.3900	0.0488	0.0334

The final weights (**Table 12**) revealed distinct priorities among the three primary dimensions *Expression and Application* (0.4527) received the highest weight, reflecting its centrality in achieving EFL's ultimate goal of enabling learners to actively articulate and apply cultural knowledge. *Local Affective Attitudes* (0.3301) and *Local Cognition and Understanding* (0.2172) followed, emphasizing the complementary roles of emotional engagement and foundational cultural knowledge.

Table 12. Hierarchical Evaluation Index System with AHP Weight Allocation for EFL Learners' Local Cultural Awareness

Primary indicators	Secondary indicators	Weight	Combined weight	Description of Secondary Indicator

I-1Local Cognition and Understanding (0.2172)	II-1Local History	0.1544	0.0335	Learners demonstrate their ability to use English to explore and discuss various aspects of local history, including key events, influential figures, and their cultural and societal significance, etc.
	II-2 Local Cultural Practices, Art, and Literature	0.1544	0.0335	Learners demonstrate their ability to use English to interpret and discuss local art forms and literary works, highlighting their significance as expressions of local culture and creativity, etc.
	II-3 Local Development Achievements	0.1544	0.0335	Learners demonstrate their ability to use English to interpret and discuss local art forms and literary works, highlighting their significance as expressions of local culture and creativity, etc.
	II-4 Daily Life Experiences	0.1544	0.0335	Learners demonstrate their ability to use English to describe and discuss aspects of local daily life, such as diet, family structure, education, and work environments, emphasizing their relevance to personal and community experiences, etc.

II-5 National Virtues and Qualities	0.1524	0.0331	Learners demonstrate their ability to use English to interpret and discuss their nation's core values, such as resilience, hospitality, and social virtues.
II-6 Local Ethical and Legal Systems	0.0921	0.0200	Learners demonstrate their ability to use English to discuss local ethical principles and legal frameworks, emphasizing their practical role in shaping societal order, resolving conflicts, and guiding everyday behavior within the community.
II-7 Regional Geography	0.0510	0.0111	Learners demonstrate their ability to use English to describe and connect geographical features, such as landscapes, rivers, mountains, and scenic spots, with local cultural practices, tourism, and community identity, etc.
II-8 Local and Global Issues in Everyday Contexts	0.0438	0.0095	Learners demonstrate their ability to use English to understand, discuss, and analyze broader societal issues that influence their daily lives, such as community governance, economic development, environmental challenges, and

				international relations, with a focus on their practical impact and global relevance
	II-9 Local Language and Dialect Varieties	0.0430	0.0093	Learners demonstrate their ability to use English to discuss local language varieties, including dialects or regional expressions, and their connections to Standard English in terms of usage, meaning, and cultural significance.
	II-10 Cultural Pride	0.3578	0.1181	Learners' high identification and pride in local culture, and confidence demonstrated in cross-cultural communication.
I-2Local Affective Attitudes (0.3301)	II-11 Interest in Cross-Cultural Understanding	0.3578	0.1181	Learners' interest in understanding and discussing other cultures and the similarities and differences with their local culture through English, coupled with the ability to maintain a critical and open-minded approach during foreign language learning and cross-cultural communication,

				avoiding blind adoration or rejection of other cultures.
	II-12 Motivation to Express and Communicate Local Identity	0.1729	0.0571	Learners' motivation and willingness to express and disseminate local culture, identity, and values through English learning.
	II-13 Emotional Attachment to Local Culture	0.1115	0.0368	Learners' emotional attachment and identification with local culture.
	II-14 Use of English for Local Storytelling	0.3019	0.1367	Learners' ability to freely tell stories related to local culture in English
I-3 Local Expression and Application (0.4527)	II-15 Promotion and Preservation of Local Image	0.3019	0.1367	Learners' initiative to promote and preserve local image in cross-cultural communication, showcasing local cultural values through English.
	II-16 Interdisciplinary Knowledge Application	0.1843	0.0834	Learners' ability to integrate interdisciplinary knowledge of local culture (such as history, art, geography, etc.) in English expression.

II-17 Adaptation of Language to Reflect Local Norms	0.1178	0.0533	Learners' ability to adjust language expression according to local cultural and social norms in cross-cultural communication.
II-18 Cultural Comparison	0.0942	0.0426	Learners' ability to compare local culture with target culture in cross-cultural communication.

In terms of secondary indicator weights, *Use of English for Local Storytelling* (0.3019) and *Promotion and Preservation of Local Image* (0.3019) received the highest global weights (0.1367 each), emphasizing narrative-driven cultural advocacy as a core skill.*Cultural Pride* (0.3578) and *Critical Openness in Cross-Cultural Engagement* (0.3578) dominated the dimension of *Local Affective Attitudes* (global weight = 0.1181, each), reflecting experts' emphasis on balancing cultural confidence. *Local History* (0.1544), *Local Cultural Practices, Art, and Literature* (0.1544), and *Daily Life Experiences* (0.1544) held equal weights (global weight=0.0335, each), indicating their foundational equivalence in cultural knowledge acquisition.

This three-phase process, combining Delphi consensus-building with AHP weighting, yielded a robust evaluation system comprising 3 primary and 18 secondary indicators. The hierarchical structure systematically integrates cognitive, affective, and behavioral dimensions of local cultural awareness, providing a quantifiable framework for assessing EFL learners' intercultural competence.

Discussion

This study constructed an evaluation index system for local cultural awareness in EFL learning through the Delphi method and Analytic Hierarchy Process (AHP), addressing the gap in existing frameworks that prioritize Anglophone cultural content over learners' native cultural identities. Below, we interpret these findings through the lens of cultural pedagogy and language acquisition theories, addressing why certain indicators were revised, retained, or removed, and their implications for EFL instruction.

The study confirmed that local cultural awareness in EFL learning can be systematically evaluated through three dimensions: *Local Cognition and Understanding, Local Affective Attitudes*, and *Local Expression and Application*. The AHP-derived weights revealed that *Local Expression and Application* (0.4527) holds the highest priority, followed by *Local Affective Attitudes* (0.3301) and *Local Cognition and Understanding* (0.2172). Among secondary indicators, *Use of English for Local Storytelling, Promotion and Preservation of Local Image* emerged as the most critical skills, while *Cultural Pride* and *Critical Openness in Cross-Cultural Engagement* dominated the affective dimension. Meanwhile, *Local History*, *Local Cultural Practices, Art, and Literature*, *Local Development Achievements*, and *Daily Life Experiences* exhibit relatively high weights under the dimension of Cognition, revealing the multifaceted emphasis on local cognition and understanding in English learning.

The prioritization of Local Expression and Application resonates with Byram's (1997) model of intercultural communicative competence (ICC), which emphasizes transforming cultural knowledge into actionable skills. The high emphasis on storytelling and cultural advocacy reflects the growing importance of narrative-driven pedagogy in English [1,2], where learners actively articulate cultural identity rather than passively absorb content.

In the affective dimension, the study revealed a balanced emphasis on *Cultural Pride* (0.3578) and *Openness in Cross-Cultural Engagement* (0.3578). These results are consistent with Kramsch's [25] view that language education should avoid cultural essentialism and foster students' ability to negotiate mixed identities. The same weighting of *Cultural Pride* and *Openness in Cross-Cultural Engagement* effectively addresses criticism of early patterns. Those patterns either overemphasize cultural loyalty or prioritize the global perspective at the expense of local roots [47] . By resisting binary oppositions between local loyalty and global assimilation, our findings realize what Dervin [48] terms "Critical Cultural Awareness", where cultural confidence coexists with reflexive skepticism toward both native and target cultures.

Under the primary indicator *Local Cognition and Understanding*, three secondary indicators, *Daily Life Experiences*, *Local Cultural Practices, Art, and Literature,* and *National Virtues and Qualities,* achieved near-perfect consensus (CV = 0.00). These findings highlight the importance of concentrating on content that is culturally relevant, enabling learners to readily place it in context and engage in discussions using English. This finding corresponds to Alakrash's [1] finding that

cultural content directly relatable to learners' experiences enhances both language retention and identity formation. By prioritizing tangible cultural practices, this study echoes the broader trend in language education that emphasizes context-based learning, where learners engage more deeply with the material, making the learning process more relevant and meaningful. Interestingly, the equal weight assigned to *Local Development Achievements* suggests that learners should focus not only on traditional aspects of culture but also on contemporary achievements. *Understanding Local Development Achievements* is integral to fostering modern cultural identity, especially in rapidly developing societies. Achievements in science and technology, the economy, and other fields are the concrete embodiments of cultural values such as innovation and collaboration. Interpreting local achievements in English requires translating abstract cultural values into concrete cases, which aligns with Kramsch's [49] theory of "language as symbolic practice". This theory posits that language is not merely a neutral conduit for communication, but a dynamic system of signs that actively constructs meaning through culturally embedded symbolic resources.

The study also observed significant divergence in expert opinions during the first Delphi round, particularly regarding abstract cultural indicators such as the *Local Political System* and *Local Economic System* (CV = 0.45 and CV = 0.36, respectively). These high coefficients of variation suggest that experts questioned the relevance of such institutional topics in the context of EFL learning. This is in line with Coyle's [50] emphasis on integrating language learning with meaningful

content and communicative practice, rather than focusing solely on abstract cultural knowledge. Coyle argues that in CLIL (Content and Language Integrated Learning) settings, the focus should be on "learning content through language and learning language through content" , highlighting the potential disengagement caused by overly complex cultural content that diverts learners' attention from communicative competence. Therefore, by removing or merging these abstract topics, the study ensures that the cultural content remains accessible and relevant to students. This approach is consistent with Porto's call [2] to prioritize the application of cultural knowledge over its memorization, thereby enhancing students' ability to use cultural knowledge in real-life communicative contexts.

The study retained the indicator *Local and Global Issues in Everyday Contexts* (M = 3.93) regarding its crucial role in bridging local identity with global citizenship. Our experts want to keep the indicator. Even though it has a relatively lower average score. Some experts emphasized that, although mastery of this indicator demands advanced language skills, its value lies in cultivating critical global citizenship. Learners are encouraged to neither uncritically adopt global norms nor strictly cling to local traditions. This conclusion is consistent with Gu's [28] findings that, while internationalized programs risk curricular homogeneity, they can paradoxically foster cultural heterogeneity by exposing students to diverse values and prompting critical reflections on globalization. And Gu's research warns against seeing English proficiency as the same as internationalization. The retained indicator implements Gu's call for curricula, which combines practical language

skills with cultural awareness, enabling learners to maintain local identities while developing flexibility for global communication.

Conclusion

This study establishes a foundational framework for evaluating local cultural awareness in EFL education based on the Delphi method and Analytic Hierarchy Process (AHP). The evaluation index system covers three first-level indicators: *Local Expression & Application* (45.72%), *Local Affective Attitudes* (33.01%), and *Local Cognition & Understanding* (21.27%). By converting Kramsch's[49] symbolic competence theory into quantifiable metrics—such as *Cultural Comparison* (0.0426) and *Promotion and Preservation of Local Image* (0.1367)—this framework bridges a critical gap between sociocultural language theories and classroom assessment practices. These dimensions offer practical strategies aimed at reducing the excessive focus on Anglophone cultural content within standardized curricula. The evaluation system is further subdivided into 18 secondary indicators, such as *Local Development Achievement*, *Cultural Pride*, *Openness in Cross-Cultural Engagement*, *and Promotion and Preservation of Local Image.*

The proposed evaluation framework offers flexible applications across diverse EFL educational contexts. It can function as both a diagnostic and developmental tool. Primarily, curriculum developers can use it to assess cultural balance in materials, such as comparing coverage of local festivals versus Western holidays in

textbooks. In blended learning environments, this framework dynamically adjusts instructional content based on learners' cultural engagement levels. For instance, AI tutors can personalize feedback in virtual heritage activities by prioritizing *Cultural Pride* metrics when students demonstrate strong local identity connections. Additionally, policy makers might adopt the hierarchy to benchmark institutional performance. They can assess how effectively schools cultivate glocal citizenship through metrics like *Promotion and Preservation of Local Image.* Crucially, the system empowers learners as self-reflective cultural agents. For example, students can use integrated digital portfolios to document how they blend cultural influences, such as creating English vlogs comparing regional cooking traditions. These real-world uses highlight two key strengths of the framework: it serves as an evidence-based evaluation tool while actively supporting teaching methods that value and balance cultural diversity. Lastly, the evaluation mechanism addresses biases in learners' foreign language narratives. By emphasizing local narratives, educators can identify and correct biases, fostering learners' objective and comprehensive understanding of local culture. This approach avoids excessive exaggeration or distortion of facts, prevents perceptual blindness, and promotes students' holistic development in cognition, emotion, and behavior.

While this study provides valuable insights into the evaluation of local cultural awareness in EFL learning, there are some limitations that should be considered for future research. First, the number of experts is only 15, and all are from China. Future research should consider a broader range of participants and contexts to

validate the robustness of the evaluation index system. Meanwhile, the study focuses primarily on the evaluation of local cultural awareness, but further research is needed to explore the long-term impact of this consciousness on students' language proficiency and global citizenship.

Despite these limitations, this study offers a foundation for future research and practical applications in assessing how well the local content was integrated in EFL education. By addressing the limitations identified, future studies can further refine the evaluation index system and explore its application in diverse educational contexts. Furthermore, more research should be conducted to collect and analyze quantitative and qualitative data to identify the specific needs of learners to navigate through cultural hybridization. This could enable educators to develop teaching interventions that amplify local voices while making students agile participants in global discourse.

Supporting information

S 1Fig.Research Workflow Diagram.

(TIFF)

S 2Fig.Hierarchical Model of EFL Learners' Local Cultural Awareness Evaluation System.

(TIFF)

S1Table.First Delphi Round Consultation on Secondary Indicators for EFL Learners' Local Awareness Evaluation System.

(Table)

S2Table.Second Delphi Round Consultation on Secondary Indicators for EFL Learners' Local Awareness Evaluation System.

(Table)

S3Table.Pairwise Comparison Matrix for Primary Indicators.

(Table)

S4Table.Pairwise Comparison Matrix for Secondary Indicators

(Table)

Reference

1. Alakrash H, Edam B, Bustan E, Armnazi M, Enayat A, Bustan T. Developing English language skills and confidence using local culture-based materials in EFL curriculum. *Linguistica Antverpiensia*. 2021;20(1):548-564.
2. Porto M. *Does education for intercultural citizenship lead to language learning?* Language, Culture and Curriculum. 2018;32(1):16-33. https://doi.org/10.1080/07908318.2017.1421642
3. Scally J, Parrish A, Montgomery A. Intercultural competence and languages: inextricably linked or linked inexplicably? *Compare: a journal of comparative and international education*. 2022;52(3):492-510.https://doi.org/10.1016/j.system.2018.07.005
4. Byram M. *Intercultural Communicative Competence in Foreign Language Education*. Clevedon: Multilingual Matters; 1997.
5. Zhang, H., Ge, S., & Mohd Saad, M. R. B. (2024). Formative assessment in K-12 English as a foreign language education: A systematic review. *Heliyon*, 10(10), e31367. https://doi.org/10.1016/j.heliyon.2024.e31367
6. Risager K. *Language and culture: Global flows and local complexity.* Clevedon, UK: Multilingual Matters; 2006.
7. Saavedra AR, Opfer VD. *Teaching and learning 21st century skills: Lessons from the learning sciences.* New York: Asia Society; 2012.
8. Vygotsky LS. Mind in society: the development of higher psychological processes. *Harvard University Press*. 1978.

9. Helot C, Young A. Language education and identity: bridging the local and the global. *International Journal of Bilingual Education and Bilingualism*. 2005;8(4):256-268.. https://doi.org/10.1080/13670050508668610

10. Tomlinson B, editor. Developing materials for language teaching. *Bloomsbury Publishing*. 2023.

11. Kramsch C. *Context and culture in language teaching*. Oxford: Oxford University Press; 1993.

12. Canagarajah S. *Translingual practice: Global Englishes and cosmopolitan relations.* New York: Routledge; 2012.

13. Lavrenteva E, Orland-Barak L. *The treatment of culture in the foreign language curriculum: an analysis of national curriculum documents.* Journal of Curriculum Studies. 2015;47(5):653-684. https://doi.org/10.1080/00220272.2015.1056233

14. Guo Q, Jamil H, Ismail L, Luo S, Sun Z. *Effects of problem-based learning on EFL learning: A systematic review.* PLoS One. 2024;19(12):e0307819. https://doi.org/10.1371/journal.pone.0307819

15. Yang M. *Making interdisciplinary subjects relevant to students: an interdisciplinary approach.* Teaching in Higher Education. 2009;14(6):597-606. https://doi.org/10.1080/13562510903315019

16. Chubko N, Morris JE, McKinnon DH, et al. *Digital storytelling as a disciplinary literacy enhancement tool for EFL students.* Education Tech Research Dev. 2020;68:3587-3604. https://doi.org/10.1007/s11423-020-09833-x

17. Dalton-Puffer C. *Outcomes and processes in Content and Language Integrated Learning (CLIL): current research from Europe*. na. 2008.

18. Lasagabaster D. *English achievement and student motivation in CLIL and EFL settings*. Innovation in Language Learning and Teaching. 2011;5(1):3–18. https://doi.org/10.1080/17501229.2010.519030

19. Margianti SF. The Inclusion of Local Cultural Contents in English Classes: Teachers' Perceptions. *Langue (Journal of Language and Education)*. 2022;1(1):97–105.https://doi.org/10.22437/langue.v1i1.22609

20. Wu JG, Miller L. Raising native cultural awareness through WeChat: a case study with Chinese EFL students. *Computer Assisted Language Learning*. 2019;34(4):552–582.https://doi.org/10.1080/09588221.2019.1629962

21. Kanoksilapatham B, Channuan P. EFL learners' and teachers' positive attitudes towards local community-based instruction. *Indonesian Journal of Applied Linguistics*. 2018;7(3):504-514.

22. Gay G. *Culturally Responsive Teaching: Theory, Research, and Practice*. 2nd ed. New York: Teachers College Press; 2018.

23. Aminullah F, Sada C, Sudarsono S. Local culture-based reading materials for EFL context. In: Proceedings of the International Conference on Teaching and Education (ICoTE). Vol 2, No 2. 2019.

24. Gashi L. Intercultural awareness through English language teaching: the case of Kosovo. *Interchange*. 2021;52:357-375.https://doi.org/10.1007/s10780-021-09441-5

25. Kramsch CJ. *Language and Culture*. Oxford: Oxford University Press; 1998.

26. Juan-Garau M, Jacob K. Developing English learners' transcultural skills through content-and task-based lessons. *System* 2015;54:55-68. https://doi.org/10.1016/j.system.2015.04.017

27. Wu JG, Miller L. Raising native cultural awareness through WeChat: a case study with Chinese EFL students. *Computer Assisted Language Learning*. 2019;34(4):552-582. https://doi.org/10.1080/09588221.2019.1629962

28. Gu MM, Lee JC-K. "They lost internationalization in pursuit of internationalization": students' language practices and identity construction in a cross-disciplinary EMI program in a university in China. *High Educ*. 2019;78:389-405. https://doi.org/10.1007/s10734-018-0342-2.

29. Figueras N. The impact of the CEFR. *ELT Journal*. 2012;66(4):477-485. https://doi.org/10.1093/elt/ccs037

30. ACTFL. *ACTFL proficiency guidelines 2012*. https://www.actfl.org.

31. Ait-bouzid H. Exploring global citizenship as a cross-curricular theme in Moroccan ELT textbooks. *Eurasian Journal of Applied Linguistics*. 2020;6(2):229-242. https://doi.org/10.32601/ejal.775801.

32. Ye W, Ren W, Zheng S. The Construct of the Story Continuation Writing Task: Insights From the China's Standards of English Language Ability. *Chinese Journal of Applied Linguistics*. 2021;44(3):382-398. https://doi.org/10.1515/CJAL-2021-0024.

33. Lange K. Perspectives on intercultural competence: a textbook analysis and an empirical study of teachers' and students' attitudes. *Intercultural Pragmatics*. 2011;8(2):183-208.

34. Wernicke M. Plurilingualism as agentive resource in L2 teacher identity. *System*. 2018;79:91-102.https://doi.org/10.1016/j.system.2018.07.005

35. McNamara T. Managing learning: authority and language assessment. *Language Teaching*. 2011;44(4):500-515. https://doi.org/10.1017/S0261444811000073

36. Hsu CC, Sandford BA. *The Delphi Technique: Making Sense of Consensus*. Practical Assessment, Research, and Evaluation. 2007;12(1):10.https://doi.org/10.7275/pdz9-th90

37. Deardorff, D. K. Identification and assessment of intercultural competence as a student outcome of internationalization. *Journal of Studies in International Education*. 2006;10(3):241-266.https://doi.org/10.1177/1028315306287002

38. Fang F, Baker W. "A more inclusive mind towards the world": English language teaching and study abroad in China from intercultural citizenship and English as a lingua franca perspectives. *Language Teaching Research*. 2018;22(5):608-624.https://doi.org/10.1177/1362168817718574

39. Zhang D, Wu JG. Enhancing EFL learners' native cultural awareness via project-based learning. *TESL-EJ*. 2023;27(1):n1.

40. Dewaele JM, MacIntyre PD. The two faces of Janus? Anxiety and enjoyment in the foreign language classroom. *Studies in Second Language Learning and Teaching*. 2014;4(2):237-274.https://doi.org/10.14746/ssllt.2014.4.2.5

41. Oktarina Y, Inderawati R, Petrus I. Developing local culture-based EFL reading materials for the 21st-century learning. *Studies in English Language and Education*. 2022;9(3):1128-1147. https://doi.org/10.24815/siele.v9i3.24660

42. Polit DF, Beck CT. The content validity index: are you sure you know what's being reported? Critique and recommendations. *Research in Nursing & Health*. 2006;29(5):489-497.**https://doi.org/10.1002/nur.20147**

43. Saaty TL. The analytic hierarchy process (AHP). *The Journal of the Operational Research Society*. 1980;41(11):1073-1076.

44. Hasson F, Keeney S, McKenna H. Research guidelines for the Delphi survey technique. *Journal of Advanced Nursing*. 2000;32(4):1008-1015. **https://doi.org/10.1046/j.1365-2648.2000.t01-1-01567.x**

45. Schmidt RC. Managing Delphi surveys using nonparametric statistical techniques. *Decision Sciences*. 1997;28(3):763-774. **https://doi.org/10.1111/j.1540-5915.1997.tb01330.x**

46. Price J, Rushton A, Tyros V, Heneghan NR. Expert consensus on the important chronic non-specific neck pain motor control and segmental exercise and dosage variables: An international e-Delphi study. *PLoS One*. 2021;16(7):e0253523. https://doi.org/10.1371/journal.pone.0253523

47. Keles U, Yazan B. Representation of cultures and communities in a global ELT textbook: A diachronic content analysis. *Language Teaching Research*. 2023;27(5):1325-1346.https://doi.org/10.1177/1362168820976922

48. Dervin F. *Interculturality in Education: A Theoretical and Methodological Toolbox*. London: Palgrave Macmillan; 2016. pp. 14, 16.

49. Kramsch C. The symbolic dimensions of the intercultural. *Language Teaching*. 2011;44(3):354-367.

50. Coyle D. Content and language integrated learning: towards a connected research agenda for CLIL pedagogies. *International Journal of Bilingual Education and Bilingualism*. 2007;10(5):543-562.https://doi.org/10.2167/beb459.0

S1 Fig.Research Workflow Diagram.

Click here to access/download;Figure;S1_Fig.tif.tif

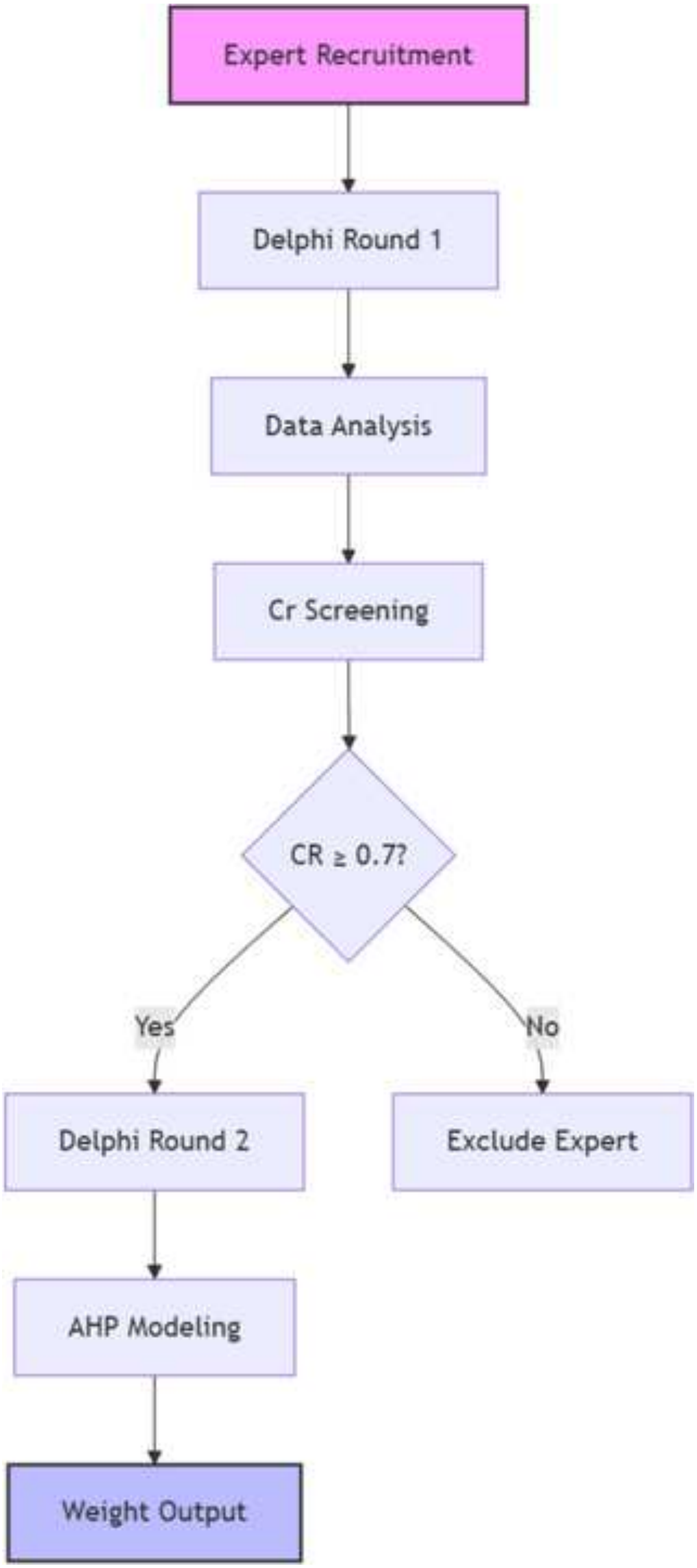

S2 Fig.Hierarchical Model of EFL Learners' Local Cultural Awareness Evaluation System.

Click here to access/download;Figure;S2_Fig.tif.tiff

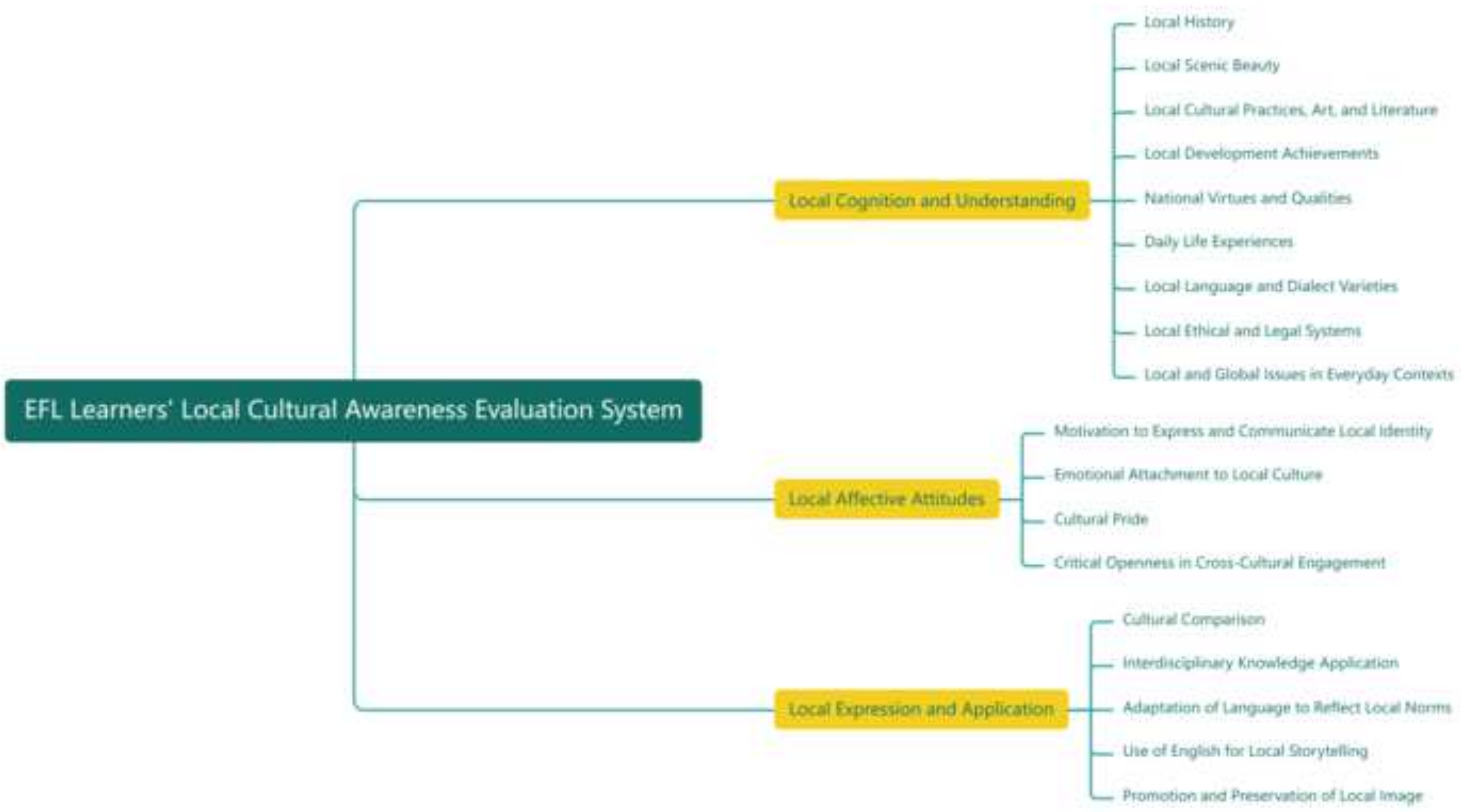

S1_TABLE.First Delphi Round Consultation on Secondary Indicators for EFL Learners' Local Awareness Evaluation System

Click here to access/download
Supporting Information
S1_Table.docx

S2_TABLE.Second Delphi Round Consultation on Secondary Indicators for EFL Learners' Local Awareness Evaluation System

Click here to access/download
Supporting Information
S2_Table.docx

S3_TABLE.Pairwise Comparison Matrix for Primary Indicators

Click here to access/download
Supporting Information
S3_Table.xlsx

S3_TABLE.Pairwise Comparison Matrix for Secondary Indicators

Click here to access/download
Supporting Information
S4_Table.xlsx
