# Supplementary material for: Assessing local cultural awareness in university EFL learners: A Delphi and AHP-based index framework
Source: PLoS One. 2025 Oct 8;20(10):e0332233. doi: 10.1371/journal.pone.0332233 (PMC12507305; doi:10.1371/journal.pone.0332233)
Supplement: Attachment — Submitted filename: PONE-D-25-18530_R1.pdf [file pone.0332233.s012.pdf]

PLOS ONE

Assessing local cultural awareness in university EFL learners: A Delphi and AHP-based index framework

--Manuscript Draft--

Manuscript Number:	PONE-D-25-18530R1
Article Type:	Research Article
Full Title:	Assessing local cultural awareness in university EFL learners: A Delphi and AHP-based index framework
Short Title:	Assessing local cultural awareness in university EFL learners
Corresponding Author:	Lunan Fan<br>Guizhou Police College<br>Guiyang, Guizhou Province CHINA
Keywords:	Local Cultural Awareness; EFL learners; Delphi Method; AHP
Abstract:	To address the neglect of local cultural identity in standardized EFL curricula, this study develops and validates an evaluation framework for local cultural awareness in EFL education. The framework operationalizes Kramsch's symbolic competence theory into measurable dimensions, achieved through two rounds of Delphi consultations with 15 EFL education experts (Kendall's W = 0.86, p < 0.01) and one round of Analytic Hierarchy Process (AHP) analysis (consistency ratios < 0.1). The finalized index system comprises three primary dimensions—Local Expression and Application (45.27% weight), Local Affective Attitudes (33.01%), and Local Cognition and Understanding (21.72%)—alongside 18 secondary indicators. Key innovations include balancing cultural pride (0.1181) with cross-cultural openness (0.1181) and integration with CEFR/ACTFL via descriptor transplantation (e.g., mapping local storytelling to CEFR C1 "pluricultural mediation"). This framework provides educators with tools to design culturally grounded assessments and advances curriculum development by transcending Anglophone-centric models.
Order of Authors:	Lunan Fan
	Guiping Cheng
	Na Yan
Response to Reviewers:	Dear Reviewer,<br><br>I hope this message finds you well. I am writing to express my sincere gratitude for the thoughtful comments and constructive feedback you provided on my manuscript. Your insights have been instrumental in helping me refine the work, and I believe they will significantly enhance the quality of the submission.<br><br>All my responses to your specific comments are detailed in the attached document. I've strived to address each point thoroughly, and I hope it demonstrates my commitment to improving the manuscript based on your valuable input.<br><br>Thank you again for your time, expertise, and dedication to this review process. It is because of reviewers like you that scholarly work can progress and mature.<br><br>Best regards,<br>Fan Lunan
Additional Information:	
Question	Response
**Financial Disclosure**<br><br>Enter a financial disclosure statement that describes the sources of funding for the	Yes

Powered by Editorial Manager® and ProduXion Manager® from Aries Systems Corporation

work included in this submission. Review the submission guidelines for detailed requirements. View published research articles from *PLOS ONE* for specific examples.<br><br>This statement is required for submission and **will appear in the published article** if the submission is accepted. Please make sure it is accurate.<br><br>**Funded studies**<br>Enter a statement with the following details:<br>• Initials of the authors who received each award<br>• Grant numbers awarded to each author<br>• The full name of each funder<br>• URL of each funder website<br>• Did the sponsors or funders play any role in the study design, data collection and analysis, decision to publish, or preparation of the manuscript?<br><br>Did you receive funding for this work?	
Please add funding details.<br>as follow-up to "**Financial Disclosure**<br><br>Enter a financial disclosure statement that describes the sources of funding for the work included in this submission. Review the submission guidelines for detailed requirements. View published research articles from *PLOS ONE* for specific examples.<br><br>This statement is required for submission and **will appear in the published article** if the submission is accepted. Please make sure it is accurate.	The initials of the authors who received each award: F.L.<br>This study was supported by the "2023 Guizhou Provincial Education Science Planning Project and Yue-Qian Special Project" from the Guizhou Provincial Department of Education. The project is titled "Constructing National Identity in College English through the Integration of Guizhou's Local Cultural Resources" and has been awarded the project number: 2023B035.<br>For further information regarding the funding support, please refer to the following links:<br>Guizhou Provincial Department of Education website:<br>https://jyt.guizhou.gov.cn/index.html<br>Announcement of the project establishment:<br>https://jyt.guizhou.gov.cn/xwzx/tzgg/202311/t20231113_83074270.html<br>The funding body was primarily involved in the release of the project guidelines and the selection of high-quality projects during the establishment process.

Powered by Editorial Manager® and ProduXion Manager® from Aries Systems Corporation

**Funded studies**<br>Enter a statement with the following details:<br>• Initials of the authors who received each award<br>• Grant numbers awarded to each author<br>• The full name of each funder<br>• URL of each funder website<br>• Did the sponsors or funders play any role in the study design, data collection and analysis, decision to publish, or preparation of the manuscript?<br><br>Did you receive funding for this work?"	
Please select the country of your main research funder (please select carefully as in some cases this is used in fee calculation).<br>as follow-up to "**Financial Disclosure**<br><br>Enter a financial disclosure statement that describes the sources of funding for the work included in this submission. Review the submission guidelines for detailed requirements. View published research articles from *PLOS ONE* for specific examples.<br><br>This statement is required for submission and **will appear in the published article** if the submission is accepted. Please make sure it is accurate.<br><br>**Funded studies**<br>Enter a statement with the following details:<br>• Initials of the authors who received each award<br>• Grant numbers awarded to each author<br>• The full name of each funder<br>• URL of each funder website<br>• Did the sponsors or funders play any role in the study design, data collection and analysis, decision to publish, or preparation of the manuscript?<br><br>Did you receive funding for this work?"	CHINA - CN
**Competing Interests**	The authors have declared that no competing interests exist.

Use the instructions below to enter a competing interest statement for this submission. On behalf of all authors, disclose any competing interests that could be perceived to bias this work—acknowledging all financial support and any other relevant financial or non-financial competing interests.<br><br>This statement is required for submission and **will appear in the published article** if the submission is accepted. Please make sure it is accurate and that any funding sources listed in your Funding Information later in the submission form are also declared in your Financial Disclosure statement.<br><br>View published research articles from *PLOS ONE* for specific examples.<br><br>**NO authors have competing interests**<br><br>Enter: *The authors have declared that no competing interests exist.*<br><br>**Authors with competing interests**<br><br>Enter competing interest details beginning with this statement:<br><br>*I have read the journal's policy and the authors of this manuscript have the following competing interests: [insert competing interests here]*<br><br>* typeset	
**Ethics Statement**<br><br>Enter an ethics statement for this submission. This statement is required if the study involved:<br><br>• Human participants<br>• Human specimens or tissue<br>• Vertebrate animals or cephalopods<br>• Vertebrate embryos or tissues	Human Subject Research (involving human participants)<br><br>The name of the institutional review board：Institutional Review Board of Guizhou Police College<br><br>The approval number:GZPC20231127<br><br>Participants volunteered to fill out the questionnaire. Before filling out the questionnaire, the purpose of the questionnaire was informed to them, and it was made clear that submitting the questionnaire would be regarded as informed consent.

Powered by Editorial Manager® and ProduXion Manager® from Aries Systems Corporation

• Field research<br><br>Write "N/A" if the submission does not require an ethics statement.<br><br>General guidance is provided below. Consult the submission guidelines for detailed instructions. **Make sure that all information entered here is included in the Methods section of the manuscript.**	Participants could withdraw from the questionnaire - filling process at any time.

Powered by Editorial Manager® and ProduXion Manager® from Aries Systems Corporation

**Format for specific study types**<br><br>**Human Subject Research (involving human participants and/or tissue)**<br>• Give the name of the institutional review board or ethics committee that approved the study<br>• Include the approval number and/or a statement indicating approval of this research<br>• Indicate the form of consent obtained (written/oral) or the reason that consent was not obtained (e.g. the data were analyzed anonymously)<br><br>**Animal Research (involving vertebrate animals, embryos or tissues)**<br>• Provide the name of the Institutional Animal Care and Use Committee (IACUC) or other relevant ethics board that reviewed the study protocol, and indicate whether they approved this research or granted a formal waiver of ethical approval<br>• Include an approval number if one was obtained<br>• If the study involved *non-human primates*, add *additional details* about animal welfare and steps taken to ameliorate suffering<br>• If anesthesia, euthanasia, or any kind of animal sacrifice is part of the study, include briefly which substances and/or methods were applied<br><br>**Field Research**<br><br>Include the following details if this study involves the collection of plant, animal, or other materials from a natural setting:<br>• Field permit number<br>• Name of the institution or relevant body that granted permission	
**Data Availability**<br><br>Authors are required to make all data underlying the findings described fully available, without restriction, and from the time of publication. PLOS allows rare exceptions to address legal and ethical concerns. See the PLOS Data Policy and FAQ for detailed information.	Yes - all data are fully available without restriction

A Data Availability Statement describing where the data can be found is required at submission. Your answers to this question constitute the Data Availability Statement and **will be published in the article**, if accepted.<br><br>**Important:** Stating 'data available on request from the author' is not sufficient. If your data are only available upon request, select 'No' for the first question and explain your exceptional situation in the text box.<br><br>Do the authors confirm that all data underlying the findings described in their manuscript are fully available without restriction?	
**Describe where the data may be found in full sentences. If you are copying our sample text, replace any instances of XXX with the appropriate details.**<br><br>• If the data are **held or will be held in a public repository**, include URLs, accession numbers or DOIs. If this information will only be available after acceptance, indicate this by ticking the box below. For example: *All XXX files are available from the XXX database (accession number(s) XXX, XXX.*).<br>• If the data are all contained **within the manuscript and/or Supporting Information files**, enter the following: *All relevant data are within the manuscript and its Supporting Information files.*<br>• If neither of these applies but you are able to provide **details of access elsewhere**, with or without limitations, please do so. For example:<br><br>*Data cannot be shared publicly because of [XXX]. Data are available from the XXX Institutional Data Access / Ethics Committee (contact via XXX) for researchers who meet the criteria for access to confidential data.*<br><br>*The data underlying the results presented in the study are available from (include the name of the third party*	All data files are available from the Figshare database (link: https://figshare.com/s/d40943028c0d35d84f8f) with DOI: 10.6084/m9.figshare.29115743 under a CC BY 4.0 license.

and contact information or URL).<br>• This text is appropriate if the data are owned by a third party and authors do not have permission to share the data.<br><br>* typeset	
Additional data availability information:	Tick here if the URLs/accession numbers/DOIs will be available only after acceptance of the manuscript for publication so that we can ensure their inclusion before publication.; Tick here if your circumstances are not covered by the questions above and you need the journal's help to make your data available.

Powered by Editorial Manager® and ProduXion Manager® from Aries Systems Corporation

Manuscript Click here to access/download;Manuscript;Manuscript.docx

Assessing local cultural awareness in university EFL learners: A Delphi and AHP-based index framework

Fan Lunan [1], **Cheng Guiping** [1], **Yanna** [2]

1 Department of Theoretical Education, Guizhou Police College, Guiyang, Guizhou Province, China,
2 School of Foreign Languages, Guizhou Education University, Guiyang, Guizhou Province, China

* Fanlunan318@sina.com(FL)

Abstract

To address the neglect of local cultural identity in standardized EFL curricula, this study develops and validates an evaluation framework for local cultural awareness in EFL education. The framework operationalizes Kramsch's symbolic competence theory into measurable dimensions, achieved through two rounds of Delphi consultations with 15 EFL education experts (Kendall's W = 0.86, $p < 0.01$) and one round of Analytic Hierarchy Process (AHP) analysis (consistency ratios < 0.1). The finalized index system comprises three primary dimensions—Local Expression and Application (45.27% weight), Local Affective Attitudes (33.01%), and Local Cognition and Understanding (21.72%)—alongside 18 secondary indicators. Key innovations include balancing cultural pride (0.1181) with cross-cultural openness (0.1181) and integration with CEFR/ACTFL via descriptor transplantation (e.g., mapping local storytelling to CEFR C1 "pluricultural mediation"). This framework

provides educators with tools to design culturally grounded assessments and advances curriculum development by transcending Anglophone-centric models.

Introduction

In today's globalized world, English proficiency is regarded as an essential skill for communication across borders. English as a Foreign Language (EFL) programs are increasingly designed not only to enhance learners' language skills but also to develop their ability to engage with the global community. Learners face "Cultural Aphasia", a term proposed by Cong from Nanjing University in China. It describes the situation where learners, despite having a high proficiency in a foreign language, find it challenging to convey the rich and unique aspects of their native culture [1]. This linguistic-cultural disjuncture has catalyzed reforms in EFL curricula, where there is growing recognition of the importance of integrating localized content into language learning to make it more relevant and meaningful to learners' lived experiences [2, 3].

Although the advantages of integrating local elements into the EFL curriculum have gained more attention, it is still challenging to effectively assess how learners exercise their agency and demonstrate local identity through their plurilingual competence in English language education [4]. At present, most of the evaluation systems excessively focus on the assessment of language skills and cross-cultural communication ability. For example, frameworks such as the CEFR are effective in benchmarking grammatical accuracy. However, they inadequately capture learners'

ability to articulate culturally - grounded narratives. This lack of assessment is particularly prominent in areas with significant cultural diversity, because the local cultural characteristics profoundly affect the actual application scenarios of language.

Given the importance of evaluating local cultural awareness in EFL learning, this study aims to develop a comprehensive evaluation index system. And the system will focus on three key dimensions: cognitive, affective, and behavioral. Such dimensions have been identified in previous research as critical components of language learning and intercultural competence [5,6]. In particular, the cognitive dimension will measure learners' understanding of local cultural, social, and historical contexts, and their ability to express this knowledge in English. The affective dimension will evaluate learners' attitudes toward expressing local content, including their emotional connection to their region and their motivation to share local stories. Finally, the behavioral dimension mainly focuses on how learners use English to express and spread local culture in practical communication.

The evaluation system constructed by this research will serve as a valuable resource for educators, enabling them to evaluate how well learners can interact with and represent their local culture when using English. The goal is to promote a comprehensive approach to language learning that integrates both global and local perspectives, fostering learners who can confidently and skillfully interact within diverse cultural contexts.

Literature review

Local cultural awareness in EFL learning

Local cultural awareness in EFL learning refers to the conscious integration of learners' local context into the language learning process. The idea of embedding local content in EFL curricula stems from the understanding that language learning should not be isolated from the socio-cultural background [7, 8]. Rooted in sociocultural theory from Vygotsky [9], local cultural consciousness emphasizes the importance of situational sensitivity. Educators need to be able to recognize and respond to the complex interactions within the sociocultural context in which learners are situated, such as language use, historical background, and community connections. By utilizing cultural symbols and designing collaborative learning activities, educators can help students integrate theoretical knowledge with their local experiences, forming a meaningful knowledge network. This approach aims to foster the co-development of students' cognitive growth, cultural identity, and critical thinking skills. Tomlinson [9] conceptualizes it as a dynamic metacognitive skill that enables learners to "notice, analyze, and negotiate linguistic hybridity" , such as code-switching patterns in multilingual communities. This construct extends beyond passive cultural literacy.

The theoretical framework of local cultural awareness is also rooted in other disciplines. Under the model of cultural ecology, Kramsch [11] put forward the concept of "third space", believing that the EFL classroom should be a place for

dynamic negotiation between local culture and global culture. Fan [12] provides empirical evidence that aligns with Kramsch's perspective, demonstrating how structured feedback in EFL writing can enhance cultural articulation. This suggests that feedback mechanisms can play a crucial role in helping learners develop cultural awareness and effectively express their local identities in English.

From the perspective of intercultural communication theory, Byram [5] "Intercultural competency model" emphasizes that EFL learners need to develop critical reflection by comparing local cultures with target cultures. Integrating research from multilingual studies and sociolinguistics, Canagarajah [13] advocates that multilingual speakers incorporate their own languages and values into English. This integration initiates various negotiation strategies that assist them in interpreting other distinct varieties of English and in constructing new norms.

Cognitive and interdisciplinary demands in tertiary-level EFL education

English plays a vital role in cultural integration within foreign language education, making cultural inclusion in TEFL instruction critically important [14]. Contemporary university-level EFL teaching requires combining higher-order thinking (HOT) with cross-disciplinary cognitive skills. Advanced learners at this level must move beyond basic language mastery to demonstrate analytical abilities for processing complex, multi-subject content [15]. Integrating HOT into curricula pushes students past memorization, developing their critical analysis, problem-solving,

and information synthesis across disciplines [3,10]. Learners tackle interdisciplinary challenges in English, demanding knowledge integration from multiple fields to form sophisticated responses [16]. For example, Chubko et al.[17] showed how digital storytelling (DST) improves STEM literacy by merging language skills with technical knowledge and social identity expression, proving cross-subject methods' value in EFL contexts.

Moreover, studies in Content and Language Integrated Learning (CLIL) confirms HOT and interdisciplinary skills' necessity for advanced EFL education. CLIL approaches emphasize the teaching of both content and language simultaneously. By encouraging learners to use English as a medium to acquire knowledge of subjects such as history, geography, and science, both language proficiency and cognitive development of EFL learners was strengthened [18,19]. Complex cognitive skills such as critical analysis, reflection, and synthesis are required, building competencies that correspond to modern educational goals.

Benefits of local content integration in EFL instruction

Despite the importance of local content in language learning, EFL programs have traditionally emphasized the cultures of English-speaking countries, which can lead to a disconnect between learners and their local realities. Indonesian junior high textbooks exemplify this issue, prioritizing Western cultural references while neglecting regional traditions [20]. This imbalance leaves students more familiar with foreign customs than their own, limiting discussions about local values. Such

detachment frames English as an external code rather than a meaningful communication tool, potentially reducing motivation and hindering acquisition [21].

Emerging studies highlight local culture integration's positive impacts on EFL outcomes. Gu and Lee [22] highlight the need to blend international and local viewpoints in teaching. This dual focus allows students to apply English meaningfully in both global exchanges and community contexts. Kanok-Xirapathum and Chanu [23] observed Thai students responding positively to lessons incorporating community content. Emotional benefits are also evident, as students demonstrate greater motivation when local culture is integrated into learning, as shown by Aminullah et al. [24] in Indonesia and Gashi [25] in Kosovo. Porto [3] observed that local cultural narratives in Argentine English curricula improve language skills and foster cultural pride.

From a cognitive perspective, Kramsch [26] emphasized the inseparability of language and culture, noting that language learning is not only about acquiring linguistic forms but also about understanding cultural meanings, which deepens the cognitive process of language acquisition. This aligns with findings that the cognitive feedback provided by digital tools can significantly impact learners' cognitive development and application of cultural knowledge in language learning [27]. Gao et al.[28] further demonstrate that task-induced involvement and time on task can significantly influence vocabulary acquisition in L2 learners, suggesting that culturally-relevant tasks enhance cognitive engagement and cultural knowledge integration. Recent research reinforces this idea by highlighting how feedback

mechanisms can enhance learners' cognitive engagement with cultural content, thereby enriching their understanding and application of cultural knowledge in language learning contexts [29].

Moreover, research indicates that integrating local content enhances intercultural competence and practical language skills [30]. Canagarajah [13] holds that navigating both local and global cultures through English enhances learners' communicative competence, while Juan-Garau and Jacob [31] show that when learners engage with real-world, culturally relevant scenarios, they develop practical communication skills. Wu and Miller [21] demonstrated that Chinese EFL learners improved intercultural skills by using WeChat to discuss regional festivals and traditions. Their study showed how digital platforms can connect language practice with cultural knowledge application.

These findings confirm that effective EFL instruction requires balancing worldwide communication needs with culturally rooted learning experiences.

Gaps in current assessment frameworks

Although integrating local content into EFL curricula shows clear benefits, measuring learners' cultural engagement through English remains challenging. Widely used evaluation frameworks, such as the Common European Framework of Reference for Languages (CEFR), focus primarily on assessing linguistic proficiency and intercultural competence but do not adequately address the assessment of local cultural awareness [32]. For example, the CEFR's "can do" description focuses on

specific language tasks that learners can accomplish at different language levels. While these standards partially address cross-cultural awareness, they lack tools to evaluate how students articulate their cultural identities in English [2].

This limitation extends to other widely adopted frameworks. The ACTFL Proficiency Guidelines [33], while emphasizing task-based performance in real-world contexts, primarily measures learners' ability to "describe local cultural practices" as a sub-skill of interpersonal communication, without establishing explicit criteria for evaluating the depth or authenticity of cultural identity expression [34,35]. Similarly, UNESCO's Global Citizenship Education Framework (2015), though containing a socio-emotional domain that addresses "sense of belonging to local communities," lacks operationalized indicators for assessing how learners articulate their cultural roots through language [36].

Even frameworks explicitly incorporating cultural dimensions exhibit gaps. China's Standards of English Ability (CSE) mandate "introducing Chinese traditional culture in English" as a competence requirement, but its assessment rubrics remain anchored in linguistic accuracy rather than cultural authenticity [37]. The German National Educational Standards, while integrating intercultural competence as a curricular component, have not adequately addressed the assessment of local cultural awareness. Existing assessment frameworks predominantly focus on the comparison of cultural differences and the understanding of target cultures, neglecting the evaluation of learners' awareness and expression of their own local culture [38].

These gaps stem from two structural limitations in current frameworks. Firstly,

most separate linguistic competence from cultural identity assessment. For instance, CEFR's pluricultural competence descriptors (e.g., "mediating cultural ambiguities") remain isolated from language production scales. Secondly, frameworks like CEFR measure adaptability to external cultures but fail to capture the dynamic process of reconstructing local identity through L2 [39] .

Recent attempts to address these gaps through CEFR companion volumes show limited progress. While introducing "mediation scales" for cultural interpretation, they still lack descriptors for self-referential cultural expression. As McNamara [40] critically notes, "The dominant frameworks reduce culture to a set of learnable behaviors rather than a lived identity that interacts with language acquisition" .

To address this gap, this study aims to establish a comprehensive evaluation index system through a series of surveys using the Delphi method, allowing researchers to analyze the opinions of expert panels by collecting, combining and quantifying multiple opinions on complex issues. By creating a tailored assessment system, this study aims to contribute to the growing body of literature on English learning by providing educators with a tool to assess learners' ability to integrate into local culture through English. This framework will enable educators to assess not only language proficiency, but also the learners' ability to use English as a medium to express their local identity. Ultimately, the goal is to promote a more comprehensive approach to language learning that values both global and local perspectives and fosters learners who have the confidence and ability to navigate different cultural environments.

Methods

Participants

In accordance with the Delphi method guidelines [41] that suggest a sample size of 15-20 experts for consensus - driven studies, 15 EFL educators (**Table 1**) were invited to participate in the study and formed the expert panel. The recruitment period for this study spanned from November 30, 2023, to March 1, 2024. These 15 participants were recruited from a variety of academic institutions across China, which guaranteed geographical and institutional diversity. All participants provided written informed consent, and the study was approved by the Institutional Review Board of GPC College (GZPC20231127). To ensure the maintenance of expert anonymity during the Delphi rounds, the survey materials explicitly guaranteed strict confidentiality. Experts were not made aware of each other's identities, and all responses were collected and handled to ensure full anonymization throughout the process.

The expert panel was composed of EFL specialists who were required to meet the following three selection criteria: (1) a minimum of 10 years' experience in EFL curriculum design; (2) research conducted in the field of EFL; and (3) holding senior professional titles such as Professor or Associate Professor. This rigorous selection process ensured that the panel's input was both authoritative and relevant to the study's objectives.

Table 1. Demographic characteristics of expert participants (N=15).

Category	Subgroup	n	Category	Subgroup	n
**Age**	≤35 years	4	**Research experience**[a]	Extensive	9
	36-45 years	9		Moderate	5
	46-55 years	2		Limited	1
**Years of EFL teaching experience**	10-19 years	13	**Teaching experience**[b]	Extensive	11
	20-30 years	2		Moderate	4
**Highest degree**	Ph.D.	13	**Current teaching position**	Full Professor	10
	Master's	2		Associate Professor	5

[a] Research experience criteria: Extensive (≥10 publications), Moderate (5-9), Limited (5).

[b]Teaching experience criteria: Extensive (≥10 years), Moderate (5-9), Limited (5).

Preliminary establishment of indicators

To comprehensively and systematically assess the awareness of native culture among advanced foreign language learners, this study adopted a multi-method approach to construct an initial set of indicators. First, researchers searched the Web of Science database using keywords such as "foreign language native awareness," "cultural competence assessment," and "place-based pedagogy." This search aimed to identify existing literature relevant to the topic, providing a solid theoretical foundation for the establishment of indicators. Concurrently, two rounds of focus group discussions were conducted with the research team members (all front-line foreign language teachers at universities) to clarify concepts, perform redundancy checks, ensure domain coverage, and verify operational feasibility. These steps are

crucial for refining the initial set of indicators, ensuring that the selected indicators are not only conceptually sound but also practically feasible and applicable in the context of assessing the awareness of native culture among advanced foreign language learners.

The assessment framework was grounded in a tripartite model of cultural competence [5], [35], which posits cultural competence as a synthesis of cognitive, affective, and behavioral dimensions. The selection of this model is justified by its holistic capacity to capture both internal development (e.g., knowledge and attitudes) and external manifestations (e.g., communicative behaviors) of cultural awareness, a duality critical for evaluating EFL learners' ability to navigate local-global identity tensions [42]. Specifically, the cognitive dimension focuses on learners' knowledge and understanding of local cultural, social, and historical contexts and their ability to express this knowledge in English, as measured through indicators such as cultural schema activation and contextualized language production [2,43]. The affective dimension, in turn, targets learners' attitudes toward local-global hybridity, with affective indicators measuring their interest in using English as a tool for self-expression and their pride in local cultural narratives [44]. Finally, the behavioral dimension assesses how learners utilize their knowledge of local content in practical settings, highlighting adaptive communication strategies that conform to local linguistic standards and social customs [45]. Cultural understanding, attitude formation, and adaptive communication define key dimensions. They serve as organizing principles for categorizing indicators. Furthermore, the tripartite structure

aligns with contemporary pedagogical goals of fostering not only linguistic proficiency but also intercultural citizenship, enabling learners to act as mediators between their local context and global discourse [3].

Guided by the theoretical synthesis, a preliminary evaluation system comprising three primary indicators was developed. Underpinning these primary indicators, 24 secondary indicators were formulated for the initial consultation phase (S1 Table). Specifically, under the *Local Cognition and Understanding* primary indicator, there are 14 secondary indicators including *Local History*, *National and Regional Geography*, and *Local Cultural Practices, Art, and Literature*; under the *Local Affective Attitudes* primary indicator, there are 4 secondary indicators including *Motivation to Express and Communicate Local Identity*, *Cultural Pride*, and *Oneness in Cross-Cultural Understanding*; under the *Local Expression and Application* indicator, 6 secondary indicators were initially proposed, such as *Cultural Comparison Skill*, *Contextualized Language Use*, and *Promotion and Preservation of Local Image*.

Procedures

In this study, three rounds of expert consultations were conducted using paper-based inquiry materials. The first two rounds conformed to the Delphi procedure, while the final round adopted the Analytic Hierarchy Process (AHP). The survey procedures and analysis stages are shown in Fig 1.

Fig 1. Research Workflow Diagram

Delphi procedure. The Delphi method was used to gather expert opinions on the key indicators for assessing local cultural awareness in EFL learners. The method involved organizing several rounds of questionnaires to solicit feedback from experts in the field of language education. Through iterative surveys and feedback processes, consensus on specific evaluation indicators was sought. Anonymity in the process allowed for more candid expert feedback, while the statistical analysis provided insight into the central tendencies and variances in expert opinions. These characteristics make the Delphi method particularly effective for educational research where diverse expert perspectives are required [41].

In the first round, opinions were collected from experts on the necessity of the three primary indicators and 24 secondary indicators. The analysis involved descriptive statistics, including the calculation of the mean (M) and standard deviation (SD) to validate initial indicators. This survey lasted for about 4 weeks, during which experts were asked to use a 5-point scale to evaluate each indicator, ranging from "not important" to "very important". In this round of evaluation, participants also rated their familiarity with evaluation criteria (Cs) and described their judgment rationale (Ca). The basis for judgment (Ca) includes practical experience, logical reasoning, and an understanding of domestic and international language education trends. These self-assessments help ensure that the feedback provided is based on a well-informed perspective. The formula for calculating the expert authority coefficient Cr is:

$$\mathrm{Cr} = \frac{\mathrm{Ca}+\mathrm{Cs}}{2}$$

It is generally believed that when the expert authority coefficient Cr is greater than or

equal to 0.7 [46], the research results are considered reliable. The assignments for Ca and Cs are shown in **Table 2** and

Table 3 respectively.

Table 2. Assignment for expert judgment basis (Ca).

Judgment Basis	Impact Level		
	High Impact	Moderate Impact	Low Impact
Deductive Reasoning	0.3	0.2	0.1
Practical Experience	0.5	0.4	0.3
Understanding of Domestic and International Language Education Trends	0.1	0.1	0.1
Intuition	0.1	0.1	0.1
Total	1.0	0.7	0.6

Table 3. Familiarity coefficient assignment (Cs).

Familiarity Level	Coefficient (Cs)
Extremely Familiar	1.0
Very Familiar	0.75
Moderately Familiar	0.50
Slightly Familiar	0.25
Not Familiar	0.00

The second round focused on revising and refining the indicators based on the feedback from the first round. A revised version of the assessment system was created for this round. After collecting and analyzing the feedback from the first round, indicators considered irrelevant or meaningless were removed. Researchers, based on the degree of concentration and coordination of expert opinions, re-sorted the remaining indicators and revised the content of the consultation questionnaire to ensure its accuracy and applicability. Indicators with low consensus ($M < 3.5$, $SD >$

1.0, CV > 25%) were eliminated to ensure a higher level of agreement among experts.

Analytic Hierarchy Process (AHP). The final round was dedicated to confirming consensus among experts and constructing the Analytic Hierarchy Process (AHP) hierarchy. The AHP was employed to assign weights to the various indicators of local cultural awareness based on expert judgment. By breaking down the complex task of evaluating learners' local cultural awareness into multiple hierarchical levels, AHP allows for precise pairwise comparisons of factors such as Local Cognition and Understanding, Local Affective Attitudes, and Local Expression and Application. These comparisons helped determine the relative importance of each indicator within the overall evaluation framework. The use of the Saaty 1~9 scale [47] in AHP provided a structured approach for experts to rate the importance of each indicator, ensuring that the weight distribution reflects the collective expert judgment.

To ensure the reliability of the pairwise comparisons, the consistency ratio (CR) was calculated using the formula CR = CI/RI, where CI is the consistency index and RI is the random consistency index. According to Saaty's [47] criteria, a CR value less than 0.1 indicates acceptable consistency. This verification step ensures that the judgments made by experts are logically consistent and free from significant biases or errors.

Results

Expert authority analysis

As shown in **Table 1**, the participants all have a high educational background, 87% held doctoral degrees with mean experience of 15.9 years (SD=4.01). Rich experience in university foreign language teaching, and good professional representation, which can provide valuable information for the structural rationality of the construction of the index system.

In the three rounds of discussion and consultation, 15 experts all participated in the whole consultation process, and the recovery rate of the questionnaire was 100%, showing the high participation and input of the participants, and providing strong support for the promotion of the research. At the same time, the participants conducted self-evaluation on the judgment basis of the problem and the familiarity with the problem. After calculation, the expert authority coefficient (Cr) was 0.867, indicating that the experts had a high degree of familiarity with the indicators and a high authority in the judgment of the indicators, and the consultation results have practical reference significance [44]. The authority coefficient of the experts is shown in **Table 4**.

Table 4. Authority coefficients of 15 experts.

Expert Number	Familiarity Coefficient (Cs)	Basis of Judgment (Ca)	Authority Coefficient (Cr)
1	1.00	1.00	1.000
2	0.75	0.90	0.825
3	0.75	1.00	0.875

4	1.00	0.90	0.950
5	1.00	0.70	0.850
6	0.50	0.90	0.700
7	1.00	0.70	0.850
8	0.75	1.00	0.875
9	0.75	0.90	0.825
10	1.00	1.00	1.000
11	0.75	0.70	0.725
12	0.75	1.00	0.875
13	0.75	1.00	0.875
14	1.00	0.90	0.950
15	0.75	0.90	0.825
Mean	0.83	0.90	0.867

Notes:

1. Cr is calculated as Cr=(Cs+Ca)/2.
2. The mean Cr value is derived from precise calculations (retaining four decimal places) before rounding.
3. A Cr value ≥ 0.7 indicates high reliability of expert opinions.

Consensus development analysis

To assess the degree of agreement among the experts, the Kendall coordination coefficient (W) was calculated. This non-parametric statistic was used to measure the consistency of a group of judges or raters on the items they ranked. The Kendall's W coefficient and chi-square tests revealed significant consensus improvement across Delphi rounds (**Table 5**).

Table 5. Expert consensus analysis across Delphi rounds.

Round	Experts (n)	Indicators (k)	Kendall's W	χ^2(df)	p-value	Interpretation
1	15	24	0.634	267.63 (23)	<0.001	Moderate consensus
2	15	19*	0.855	217.9 (18)	<0.001	Strong consensus

Notes:

1.*5 indicators eliminated after Round 1 screening

2.Degrees of freedom (df) calculated as df=k−1.

The moderate consensus (W=0.634) in Round 1 reflects expected divergence during initial indicator exploration. The significant χ^2 value ($p<0.001$) nevertheless confirms non-random agreement patterns, warranting progression to refinement rounds [49].

The second round of expert consultation yielded a Kendall's coordination coefficient W=0.855 and a p-value of <0.001. According to prior studies, a Kendall's W value between 0.6 and 0.8 [50] typically indicates strong consensus among experts. While the observed value (0.855) slightly exceeds this range, it further demonstrates an exceptionally high level of agreement among participants in the second round, confirming the reliability of the refined results. Notably, the W coefficient increased by 34.8% (from 0.634 in Round 1 to 0.855 in Round 2), demonstrating the Delphi method's efficacy in reconciling expert perspectives through iterative, structured feedback. This significant improvement highlights the robustness of the consensus-building process in narrowing divergent opinions and achieving actionable outcomes.

First Delphi survey results

In the first round of the Delphi survey, 15 experts evaluated a preliminary set of 24 secondary indicators across three primary dimensions (Cognitive, Affective, Behavioral). Statistical thresholds (Mean $M \geq 3.5$, Standard Deviation $SD < 1$, Coefficient of Variation $CV < 0.25$) were applied to assess indicator necessity. As is shown in

Table 6, 17 indicators (M_{range}=3.87-5.00, SD_{range}=0.41-0.83, CV_{range}=0.08-0.21) met all criteria and were retained for the second round.

Table 7 shows that 7 indicators failed to meet thresholds, primarily due to low relevance (M<3.12)or high opinion divergence(SD>1.01).

Table 6. Retained indicators after first Delphi round (n=17).

Indicator Name	Mean (M)	SD	CV
Local History	4.47	0.74	0.17
Local Ethical and Legal Systems	4.07	0.59	0.15
Regional Geography	3.87	0.83	0.22
Local Cultural Practices, Art, and Literature	4.80	0.56	0.12
Local Development Achievements	4.67	0.62	0.13
National Virtues and Qualities	4.80	0.41	0.09
Daily Life Experiences	4.67	0.49	0.10
Local Language and Dialect Varieties	3.87	0.52	0.13
Motivation to Express and Communicate Local Identity	4.80	0.41	0.09
Emotional Attachment to Local Culture	4.13	0.64	0.15
Cultural Pride	5.00	0.00	0.00
Openness in Cross-Cultural Engagement	5.00	0.00	0.00
Cultural Comparison	4.00	0.65	0.16
Interdisciplinary Knowledge Application	4.67	0.49	0.10
Adaptation of Language to Reflect Local Norms	4.07	0.88	0.22
Use of English for Local Storytelling	5.00	0.00	0.00
Promotion and Preservation of Local Image	4.60	0.63	0.14

Table 7. Unretained / modified indicators after first Delphi round (n=7).

**Indicator Name**	**Mean (M)**	**SD**	**CV**	**Action Taken**
Local Political System and Governance	1.40	0.63	0.45	Removed

Local Economic System	3.07	0.70	0.23	Removed
Local Social Issues	2.88	1.06	0.37	Integrated into a new indicator
International Relations and Global Issues	2.93	0.80	0.27	Removed
Local Educational System	3.13	1.25	0.40	Removed
Local Environment and Sustainable Development	3.00	0.93	0.31	Merged into A4 Local Development Achievements
Participation in Culturally Relevant Discussions	2.27	0.70	0.31	Removed

Removal of redundant indicators. *Local Political System and Governance* (M = 1.40) and *Participation in Culturally Relevant Discussions* (M = 2.27) were excluded due to low relevance and measurement challenges.

Revisions based on feedback. *Local Economic System* (M=3.07), *Local Social Issues* (M=2.88), *International Relations and Global Issues* (M=2.93) and *Local Educational System* (M=3.13) were advised to consolidate into a new indicator "Local and Global Issues in Everyday Contexts" under the primary indicator *Local Cognition and Understanding*, in order to focus on practical topics (e.g., community governance, local economic challenges). *Environment and Sustainable Development* (M=3.00) were suggested to merge into then indicator *Local Development Achievements*.

Descriptive adjustments. *Cognition of National and Regional Geography* was renamed *Cognition of Local Scenic Beauty* to emphasize cultural connections (e.g., linking landscapes to festivals). At the same time, several experts recommended

revising the descriptions of the indicators related to local history, as well as the ethical and legal systems. These revisions aim to align more closely with the objectives of EFL education and to ensure that the assessment is both practical and operational.

The revised indicator set was advanced to the second Delphi round for further validation.

Second Delphi survey results

The second Delphi survey (S2 Table) assessed expert consensus on the appropriateness of 18 indicators across three dimensions. All indicators met retention criteria ($M \geq 3.5$, $SD < 1$, $CV < 0.25$), with mean values ranging from 3.93 to 5.00, standard deviations ranging from 0.00 to 0.45, and coefficients of variation ranging from 0.08 to 0.09, confirming high agreement among experts. The results are presented below, organized by dimension.

Local cognition and understanding. Experts exhibited strong consensus on indicators under this dimension (**Table 8**). Two indicators—*Local Cultural Practices, Art, and Literature* and *National Virtues and Qualities*—achieved perfect scores (M = 5.00, SD = 0.00, CV = 0.00), reflecting unanimous agreement on their necessity for evaluating learners' cultural knowledge. Notably, Daily Life Experiences (M = 5.00, SD = 0.00) and Local Development Achievements (M=4.93, SD=0.26) also demonstrated high reliability ($CV < 0.05$), highlighting their role in connecting abstract cultural concepts to tangible contexts.

Table 8. Two-round Delphi survey results for secondary indicators under the primary indicator *Local Cognition and Understanding.*

Secondary Indicator	Mean (out of 5)		SD		CV		Action Taken
	1st	2nd	1st	2nd	1st	2nd	
Local History	4.47	4.93	0.74	0.26	0.17	0.05	Retained
Local Scenic Beauty	3.87	3.93	0.83	0.26	0.22	0.07	Retained
Local Cultural Practices, Art, and Literature	4.80	5.00	0.56	0.00	0.12	0.00	Retained
Local Development Achievements	4.67	4.93	0.62	0.26	0.13	0.05	Retained
National Virtues and Qualities	4.80	5.00	0.41	0.00	0.09	0.00	Retained
Daily Life Experiences	4.67	5.00	0.49	0.00	0.10	0.00	Retained
Local Language and Dialect Varieties	3.87	3.93	0.52	0.26	0.13	0.07	Retained
Local Ethical and Legal Systems	4.07	4.73	0.59	0.46	0.15	0.10	Retained
Local and Global Issues in Everyday Contexts	–	3.93	–	0.26	–	0.07	Retained

When looking at the improved indicators, *Local History* showed significant progress, with its mean increasing from 4.47 (SD = 0.74) in the first round to 4.93 (SD = 0.26) in the second round, and its CV decreasing from 0.17 to 0.05. This improvement suggests that the revised description of the indicator better aligns with EFL learning objectives. Similarly, *Local Ethical and Legal Systems* progressed from moderate consensus (M = 4.07, CV = 0.15) to higher agreement (M = 4.73, CV = 0.10), indicating enhanced clarity in its operational definition.

Regarding the newly added indicator, *Local and Global Issues* in Everyday Contexts (M = 3.93, SD = 0.26, CV = 0.07) was introduced in the second round to address

practical cultural engagement. Although its mean was slightly lower compared to other retained indicators, it met all statistical thresholds, justifying its inclusion in the final evaluation system.

Local affective attitudes. As is shown in

Table 9, the appropriateness of indicators related to the "emotion" dimension as reflected by experts in two rounds of surveys is shown in Table 8. All affective indicators received strong endorsement.

Cultural Pride (M = 5.00, SD = 0.00, CV = 0.00) and *Critical Openness in Cross-Cultural Engagement* (M = 5.00, SD = 0.00, CV = 0.00) achieved perfect consensus in both rounds, reflecting unanimous agreement on their critical role in fostering cultural confidence and balanced intercultural perspectives. Emotional *Attachment to Local Culture* maintained a high level of agreement (M = 4.00, SD = 0.38, CV = 0.09) in the second round, though its mean slightly decreased from 4.13 in the first round. The low CV value (0.09) confirms its reliability as a measure of learners' emotional connection to their cultural heritage.

Table 9. Two-round Delphi survey results for secondary indicators under the primary indicator *Local Affective Attitudes.*

Secondary Indicator	Mean (out of 5)		SD		CV		Action Taken
	1st	2nd	1st	2nd	1st	2nd	
Motivation to Express and Communicate Local Identity	4.80	4.93	0.41	0.26	0.09	0.05	Retained
Emotional Attachment to Local Culture	4.13	4.00	0.64	0.38	0.15	0.09	Retained
Cultural Pride	5.00	5.00	0.00	0.00	0.00	0.00	Retained

Critical Openness in Cross-Cultural Engagement	5.00	5.00	0.00	0.00	0.00	0.00	Retained

Local expression and application. Table 10 summarizes results of this dimension, demonstrating strong expert consensus on their relevance to assessing learners' ability to express and apply local culture through English.

Two indicators—*Use of English for Local Storytelling* and *Promotion and Preservation of Local Image*—received unanimous support (M = 5.00, SD = 0.00), emphasizing storytelling and advocacy as critical skills for cultural dissemination. Though other indicators show slight fluctuations, the high consensus across indicators (CV < 0.10) validates their collective role in translating cultural knowledge into actionable language skills.

Table 10. Two-round Delphi survey results for secondary indicators under the primary indicator *Local Expression and Application.*

Secondary Indicator	Mean (out of 5)		SD		CV		Action Taken
	1st	2nd	1st	2nd	1st	2nd	
Cultural Comparison	4.00	4.07	0.59	0.26	0.16	0.06	Retained
Interdisciplinary Knowledge Application	4.67	4.93	0.49	0.26	0.10	0.05	Retained
Adaptation of Language to Reflect Local Norms	4.07	4.07	0.88	0.26	0.22	0.06	Retained
Use of English for Local Storytelling	5.00	5.00	0.00	0.00	0.00	0.00	Retained
Promotion and Preservation of Local Image	4.60	5.00	0.63	0.00	0.14	0.00	Retained

On the whole, all the indicators in the second round of Delphi survey received a "Retained" status, confirming that they were deemed relevant and essential by the

experts. The retention of all indicators suggests a strong consensus among the experts on the comprehensive nature of the evaluation framework for assessing EFL learners' local cultural awareness. This unanimity demonstrates the importance of including both cognitive, affective, and behavioral dimensions in the assessment, ensuring that learners' understanding, emotional connection, and practical application of local culture are all adequately measured.

Analytic hierarchy process (AHP) weighting outcomes

The third consultation phase employed the Analytic Hierarchy Process (AHP) to determine the relative weights of indicators within the evaluation system. Following AHP protocol, a hierarchical model (S1 Fig) was constructed based on the finalized primary and secondary indicators of EFL Learners' Local Cultural Awareness. Fifteen experts from the Delphi panel were invited to complete pairwise comparison matrices for the hierarchical structure (S3 Table, S4 Table).

The expert evaluations were analyzed using AHP software to calculate eigenvectors, perform hierarchical single ranking, and conduct consistency checks. All four judgment matrices exhibited satisfactory consistency, with consistency ratios (CR) below the threshold of 0.1 and consistency indices (CI) approaching zero (**Table 11**). These results confirm the logical coherence of expert judgments and validate the weight assignments.

Table 11. AHP consistency metrics.

Hierarchy Level	λmax	CI	CR
Primary Dimensions	3.1008	0.0504	0.0970
Expression Sub-criteria	5.3500	0.0875	0.0781
Affective Sub-criteria	4.2364	0.0788	0.0886

Cognitive Sub-criteria	9.3900	0.0488	0.0334

The final weights (**Table 12**) revealed distinct priorities among the three primary dimensions *Expression and Application* (0.4527) received the highest weight, reflecting its centrality in achieving EFL's ultimate goal of enabling learners to actively articulate and apply cultural knowledge. *Local Affective Attitudes* (0.3301) and *Local Cognition and Understanding* (0.2172) followed, emphasizing the complementary roles of emotional engagement and foundational cultural knowledge.

Table 12. Hierarchical evaluation index system with AHP weight allocation for EFL Learners' Local Cultural Awareness.

Primary indicators	Secondary indicators	Weight	Combined weight	Description of Secondary Indicator
I-1Local Cognition and Understanding (0.2172)	II-1Local History	0.1544	0.0335	Learners demonstrate their ability to use English to explore and discuss various aspects of local history, including key events, influential figures, and their cultural and societal significance, etc.
	II-2 Local Cultural Practices, Art, and Literature	0.1544	0.0335	Learners demonstrate their ability to use English to interpret and discuss local art forms and literary works, highlighting their significance as expressions of local culture and creativity, etc.

II-3 Local Development Achievements	0.1544	0.0335	Learners demonstrate their ability to use English to interpret and discuss local art forms and literary works, highlighting their significance as expressions of local culture and creativity, etc.
II-4 Daily Life Experiences	0.1544	0.0335	Learners demonstrate their ability to use English to describe and discuss aspects of local daily life, such as diet, family structure, education, and work environments, emphasizing their relevance to personal and community experiences, etc.
II-5 National Virtues and Qualities	0.1524	0.0331	Learners demonstrate their ability to use English to interpret and discuss their nation's core values, such as resilience, hospitality, and social virtues.
II-6 Local Ethical and Legal Systems	0.0921	0.0200	Learners demonstrate their ability to use English to discuss local ethical principles and legal frameworks, emphasizing their practical role in shaping societal order, resolving conflicts, and guiding everyday behavior within the community.

	II-7 Local Scenic Beauty	0.0510	0.0111	Learners demonstrate their ability to use English to describe and connect geographical features, such as landscapes, rivers, mountains, and scenic spots, with local cultural practices, tourism, and community identity, etc.
	II-8 Local and Global Issues in Everyday Contexts	0.0438	0.0095	Learners demonstrate their ability to use English to understand, discuss, and analyze broader societal issues that influence their daily lives, such as community governance, economic development, environmental challenges, and international relations, with a focus on their practical impact and global relevance.
	II-9 Local Language and Dialect Varieties	0.0430	0.0093	Learners demonstrate their ability to use English to discuss local language varieties, including dialects or regional expressions, and their connections to Standard English in terms of usage, meaning, and cultural significance.
I-2Local Affective Attitudes (0.3301)	II-10 Cultural Pride	0.3578	0.1181	Learners' high identification and pride in local culture, and confidence

				demonstrated in cross-cultural communication.
	II-11 Interest in Cross-Cultural Understanding	0.3578	0.1181	Learners' interest in understanding and discussing other cultures and the similarities and differences with their local culture through English, coupled with the ability to maintain a critical and open-minded approach during foreign language learning and cross-cultural communication, avoiding blind adoration or rejection of other cultures.
	II-12 Motivation to Express and Communicate Local Identity	0.1729	0.0571	Learners' motivation and willingness to express and disseminate local culture, identity, and values through English learning.
	II-13 Emotional Attachment to Local Culture	0.1115	0.0368	Learners' emotional attachment and identification with local culture.
I-3 Local Expression and Application (0.4527)	II-14 Use of English for Local Storytelling	0.3019	0.1367	Learners' ability to freely tell stories related to local culture in English
	II-15 Promotion and Preservation of Local Image	0.3019	0.1367	Learners' initiative to promote and preserve local image in cross-cultural communication, showcasing local

			cultural values through English.
II-16 Interdisciplinary Knowledge Application	0.1843	0.0834	Learners' ability to integrate interdisciplinary knowledge of local culture (such as history, art, geography, etc.) in English expression.
II-17 Adaptation of Language to Reflect Local Norms	0.1178	0.0533	Learners' ability to adjust language expression according to local cultural and social norms in cross-cultural communication.
II-18 Cultural Comparison	0.0942	0.0426	Learners' ability to compare local culture with target culture in cross-cultural communication.

In terms of secondary indicator weights, *Use of English for Local Storytelling* (0.3019) and *Promotion and Preservation of Local Image* (0.3019) received the highest global weights (0.1367 each), emphasizing narrative-driven cultural advocacy as a core skill. *Cultural Pride* (0.3578) and *Critical Openness in Cross-Cultural Engagement* (0.3578) dominated the dimension of *Local Affective Attitudes* (global weight = 0.1181, each), reflecting experts' emphasis on balancing cultural confidence. *Local History* (0.1544), *Local Cultural Practices, Art, and Literature* (0.1544), and *Daily Life Experiences* (0.1544) held equal weights (global weight=0.0335, each), indicating their foundational equivalence in cultural knowledge acquisition.

This three-phase process, combining Delphi consensus-building with AHP weighting, yielded a robust evaluation system comprising 3 primary and 18 secondary indicators. The hierarchical structure systematically integrates cognitive, affective, and behavioral dimensions of local cultural awareness, providing a quantifiable framework for assessing EFL learners' intercultural competence.

Discussion

This study constructed an evaluation index system for local cultural awareness in EFL learning through the Delphi method and Analytic Hierarchy Process (AHP), addressing the underrepresentation of learners' native cultural identity. The following discussion will interpret the findings from educational and theoretical perspectives and link the revision or retention of indicators to the practical significance in university EFL teaching.

The study confirmed that local cultural awareness in EFL learning can be systematically evaluated through three dimensions (**Fig 2**): *Local Cognition* and *Understanding, Local Affective Attitudes*, and *Local Expression and Application*. The AHP-derived weights revealed that *Local Expression and Application* (W=0.4527) holds the highest priority, followed by *Local Affective Attitudes* (W=0.3301) and *Local Cognition and Understanding* (0.2172). Among secondary indicators (**Fig 3**), *Use of English for Local Storytelling, Promotion and Preservation of Local Image* emerged as the most critical skills, while *Cultural Pride* and *Critical Openness in Cross-Cultural Engagement* dominated the affective dimension. Meanwhile, *Local*

History, *Local Cultural Practices, Art, and Literature*, *Local Development Achievements*, and *Daily Life Experiences* exhibit relatively high weights under the dimension of Cognition, revealing the multifaceted emphasis on local cognition and understanding in English learning.

Fig 2. Weights of primary indicators for local cultural awareness assessment

Fig 3. Secondary indicator weights in cultural awareness assessment

Local expression and application dimension

The significantly higher weight assigned to *Local Expression and Application* (weight = 0.4527, the highest among the three dimensions) not only aligns with Byram's [5] intercultural communicative competence (ICC) model, which prioritizes knowledge-to-practice transformation, but also finds empirical support in Fan's [12] study. In Fan's research, action-oriented tasks yielded higher cultural retention, and this emphasis on practical alignment is further validated by the study's finding that direct written corrective feedback (WCF) enhances students' cultural expression in EFL writing—findings that complement Byram's focus on operational competence.

This study extends prior research by developing an evaluation index system for EFL learners' local cultural awareness and integrating it into project-based output tasks. This approach operationalizes Byram's Intercultural Communicative Competence (ICC) principles through context-specific language applications—a

critical yet underexplored dimension in existing literature. As demonstrated in S5 Table, in teaching practice, this study suggests replacing 45% of the traditional cultural teaching modules in EFL programs with Production-oriented tasks. For instance, students could be tasked with documenting local heritage sites in English—such as ancient bridges bearing donation inscriptions—and composing analytical narratives that systematically unpack the cultural connotations embedded in these physical spaces. Through their writing, learners should illuminate how these inscriptions exemplify traditional values of fraternal cooperation and contractual ethics, bridging historical artifacts with contemporary understanding. Through the knowledge-action transformation mechanism, this design directly responds to the highest AHP-assigned weight of this dimension, translating the ICC model from a theoretical framework into teaching practice.

Under the application dimension, the secondary indicator of Use of *English for Local Storytelling* holds one of the highest global weights (W = 0.1366), underscoring its central role in cultivating intercultural competence. In university EFL courses, this indicator can be operationalized through collaborative projects: advanced learners might document the entrepreneurial histories of local time-honored brands, creating podcasts that integrate interviews with entrepreneurs and cultural analyses (II-14 in S5 Table). These narratives can be showcased at academic conferences, fostering both language proficiency and cultural advocacy. The narrative-driven approach diverges from earlier studies that prioritized target-culture immersion [5],[11], as well as recent work that framed narratives primarily as tools for grammatical accuracy [51]. In

contrast to Fan's [12] focus on refining cultural expression via corrective feedback, our design foregrounds proactive cultural production. This approach not only enhances their language skills but also fosters a deeper engagement with cultural content and critical thinking about cultural comparisons, aligning with the principles of higher-order thinking (HOT) and interdisciplinary learning emphasized in contemporary EFL education [15, 16]. The AHP assigned a global weight of 0.1366 to this indicator, surpassing cognitive metrics such as *Local History* (global weight = 0.0335). This empirical result validates its uniqueness: the indicator integrates local storytelling with Byram's ICC model [5]. In this model, "interpretation and negotiation" are central to intercultural competence. This innovation addresses Canagarajah's [13] critique of task-based approaches neglecting cultural agency by operationalizing it through measurable narrative tasks that require learners to actively engage with and represent local cultural practices.

Additionally, the secondary indicator of *Promotion and Preservation of Local Image* (global weight= 0.1366, M = 5.00) has received high endorsement from experts, highlighting the practical value of constructing cultural discourse power. As operationalized in S5 Table, this sub-indicator (II-15) provides measurable criteria for assessing students' international communication competence in university EFL contexts. By designing international communication scenario simulations, students, under the guidance of professors, can clarify and refute misconceptions about local culture disseminated by foreign media. These practices operationalize the scholars' view that EFL teaching needs to enhance the application of cultural knowledge [3,12].

Emotional dimension

In the emotional dimension, the study shows balanced emphasis on *Cultural Pride* (global weight=0.1181) and *Openness in Cross-Cultural Engagement* (global weight=0.1181). These results are consistent with Kramsch's [26] view that language education should avoid cultural essentialism and cultivate students' hybrid identity capabilities. This equal emphasis, addressing the limitations of earlier models that either overemphasized cultural loyalty or prioritized global perspectives at the expense of local roots [52] , is operationalized through specific teaching tasks in university EFL courses.

In university EFL assessment, cultural pride is evaluated through tasks such as the "Pride Portfolio" (II-10 in S5 Table). For example, anthropology students' cultural awareness is measured by their ability to compile cultural artifacts with precise English annotations (e.g., distinguishing "butterfly patterns symbolizing ancestral blessings" from generic "insect decorations"), demonstrating how linguistic accuracy reflects cultural symbol interpretation in evaluation criteria.

Similarly, cross-cultural openness is assessed via the "Cultural Exchange Archive" (II-11 in S5 Table), where evaluation focuses on students' capacity to create bilingual packages—documenting local traditions like Dragon Boat Races in English while critically analyzing foreign customs (e.g., Christmas) from exchanged materials.

In university exchange programs, learners' performance is assessed through comparative tasks: basic learners might contrast festival food practices via

infographics, while advanced learners conduct semiotic analyses of festival symbols in academic posters. This bidirectional comparison aligns with Delvin's [53] "critical cultural awareness" for balanced cultural representation. The equal AHP weights for Cultural Pride and Openness (0.1181 each) validate this assessment approach. The equal AHP weights for Cultural Pride and Openness (0.1181 each) validate this approach. It also embodies CLIL principles [18,19], integrating language learning with cross-cultural inquiry.

Cognitive dimension

In the cognitive dimension, the study observed significant divergence in expert opinions during the first Delphi round, particularly regarding abstract cultural indicators such as the *Local Political System* and *Local Economic System* (CV = 0.45 and CV = 0.36, respectively). These high coefficients of variation suggest that experts questioned the relevance of such institutional topics in the context of EFL learning. This is consistent with Coyle's view that language learning should be combined with meaningful content and communicative practices, rather than just focusing on abstract cultural knowledge [54]. Therefore, by deleting or merging these abstract topics, the study ensures that cultural content is both comprehensible and relevant to university students.

Under the main indicator *Local Cognition and Understanding*, the three secondary indicators - *Daily Life Experiences*, *Local Cultural Practices, Art, and Literature*, and *National Virtues and Qualities* - exhibited perfect evaluative

consensus (CV=0.00). This consensus confirms the critical role of culturally-embedded assessment tasks in measuring EFL learners' ability to mediate local knowledge through English, as exemplified by "a day in the life" documentation (II-4) and local figure narration (II-5) tasks in S5 Table. Aligned with Alakrash's [2] cognitive authenticity principle, these evaluation criteria demonstrate how contextually-grounded content enhances linguistic encoding and identity positioning in assessment outcomes. The transformation task for local cultural artifacts (II-2 in S5 Table), for instance, evaluates students' symbolic mediation competence through parameters including: (1) cultural authenticity of adapted content, (2) linguistic accuracy in performance scripts, and (3) effective conveyance of embodied cultural meanings. Such multidimensional assessment operationalizes Kramsch's [49] "symbolic competence" framework, where successful performance necessitates transcending lexical equivalence to achieve semiotic resonance between form and cultural substance.

The substantial weighting of Local Development Achievements (global weight=0.0335) underscores the evaluation framework's recognition of contemporary cultural production as equally significant as traditional manifestations. This sub-indicator measures learners' techno-cultural literacy through their ability to mediate between abstract cultural values and concrete development metrics. In university assessment contexts, business students' cultural awareness is measured through tasks like creating "local development data visualization" reports in English (e.g., analyzing cultural industry GDP contributions) . Successful performance

requires operationalizing Porto's [3] knowledge model by contextualizing technological and cultural narratives within specific development metrics. The assessment thus validates learners' capacity to concretize socio-cultural abstractions through quantitative discourse. This evaluative approach bridges the epistemic divide between cultural studies and development economics in EFL contexts.

Notably, the indicator *Local and Global Issues in Everyday Contexts* (M = 3.93, global weight=0.0200), though scoring relatively low, was retained by experts for its role in cultivating critical global citizenship. In university EFL teaching, this is operationalized through graded tasks: advanced learners' critical analysis capabilities are measured through English debates on "local issues with global implications" (e.g., "ancient town tourism vs. cultural authenticity preservation"), while intermediate learners demonstrate their understanding through structured pro-con reports on topics like "dialect protection and tourism development." This differentiated assessment approach validates Gu's [22] theoretical position that internationalized curricula can sustain cultural heterogeneity through critical engagement. The evaluation criteria operationalize Gu's conceptual integration of practical language skills and cultural awareness—specifically measuring learners' capacity to articulate local identities while navigating global discourse frameworks.

Integration with existing standard frameworks in university context

The proposed local cultural awareness assessment model provides a

comprehensive approach to evaluating EFL learners' engagement with local culture. Furthermore, the practical application of this model can be aligned with widely recognized standard frameworks, such as the Common European Framework of Reference for Languages (CEFR) and the American Council on the Teaching of Foreign Languages (ACTFL) proficiency guidelines. Although this study highlights the blind spots of current frameworks (such as CEFR and ACTFL) in assessing local cultural expression, the new model is not intended to replace these standardized systems but to achieve synergistic enhancement through a hierarchical complementary strategy.

The CEFR offers a comprehensive set of language proficiency descriptors spanning diverse proficiency levels [56]. We propose integrating the local cultural awareness assessment model with the CEFR starting from the intermediate level, employing a descriptor transplantation approach. Specifically, this integration can be achieved by embedding local cultural indicators into the CEFR's mediational and plurilingual descriptors, as underscored in the document regarding "action-oriented scenarios" and "plurilingual repertoires" [56]. As outlined in S6 Table, this strategic embedding aligns secondary indicators with CEFR levels B1 to C2, illustrating how descriptors like "relaying specific information" (B2) and "facilitating pluricultural space" (C1) can be adapted to assess local cultural competence.

B1–B2 Intermediate Level Integration: At these levels, foundational local cultural indicators (e.g., II-1 Local History, II-4 Daily Life Experiences) can be integrated into CEFR's "Mediating Concepts" and "Reception of Information"

descriptors. For instance, II-1's requirement for learners to "discuss local history events in English" aligns with CEFR B2's descriptor for "relaying specific information" (Section 3.1.4.2)[56], where learners are expected to "summarize historical contexts with cultural significance". Similarly, II-6 (Local Ethical and Legal Systems) can be grafted onto CEFR's B2 "Explaining Data" scale, as the document highlights the importance of mediating social norms through language.

C1–C2 Advanced Level Integration: At higher proficiency tiers, sophisticated cultural indicators (e.g., II-15 Local Image Promotion, II-18 Cultural Comparison) map to CEFR's "Plurilingual Mediation" and "Facilitating Pluricultural Space" domains. For example, II-18's requirement to "compare local and target cultures" directly aligns with CEFR C1's descriptor for "mediating cross-cultural dialogue" (Section 7.2.2), where learners are expected to "negotiate cultural differences in complex scenarios". Additionally, II-15's focus on "promoting local identity in English" resonates with the emphasis of CEFR on "activating plurilingual repertoires for social agency" , which can be embedded in CEFR C2's "Strategic Mediation" scale.

Cross-Level Task Design for Cultural Embedding: The document's advocacy for "scenario-based assessment" (Section 7.2.1) provides a framework for designing tasks that bridge proficiency levels [56]. For instance, in B1–B2 tasks, learners could complete a "local food culture interview" (II-4) using CEFR B2's "Oral Interaction" descriptors, which emphasize "describing daily practices with cultural relevance". And in terms of C1–C2 tasks, A "local heritage preservation campaign" (II-15) could

be evaluated against CEFR C1's "Mediation of Public Discourse" criteria, requiring learners to "craft persuasive messages that integrate local values" . Similarly, ACTFL emphasizes "intercultural communicative competence," focusing on the appropriate use of language in real social and cultural contexts [33]. We propose using the evaluation indicators established in this study to form a dual-track assessment mechanism (

Fig 4). For example, a cultural awareness dimension can be added to the ACTFL Oral Proficiency Interview (OPI), allowing independent scoring of language fluency and cultural mediation while sharing weights (suggested ratio: 70% language + 30% culture). This integrated solution, which balances the emphasis on language and cultural competence, retains the reliability and validity of standardized tests and addresses the missing identity expression through the cultural competence matrix (S7 Table). It responds to Deardorff's [35] call for a "mixed assessment method" (quantitative + qualitative) to measure cultural depth. Meanwhile, the dual-track model proposed in this study requires minimal modification to the existing ACTFL protocols but significantly enhances ecological validity in multilingual contexts.

Fig 4. Schematic of the dual - track assessment process for the ACTFL OPI task

Limitations and future directions

This study represents an initial exploration of evaluating local cultural awareness among EFL learners through a Delphi-based framework, specifically contextualized

within the Chinese educational landscape. The current expert panel, consisting solely of 15 professionals from China, offers a perspective on integrating local content into EFL education. However, this composition may limit the panel's ability to fully assess the adaptability of our framework across different cultural and educational contexts. To mitigate this limitation, future research should consider expanding the panel to include experts from a variety of EFL settings, such as Latin America, Africa, and Southeast Asia, ensuring a more comprehensive validation of the framework's universal applicability.

Building on this foundation, subsequent studies could pursue two complementary paths. First, cross-cultural validation in non-Chinese settings would shed light on whether the evaluation index system requires contextual adjustments or demonstrates transferable utility. Such efforts would enrich the framework by incorporating varied perspectives on balancing local and global cultural elements in EFL curricula. Second, longitudinal investigations tracking how local cultural awareness influences students' language development and intercultural competence over time would deepen our understanding of its educational impact. Mixed-method designs combining quantitative assessments with qualitative case studies could offer nuanced insights into how learners navigate cultural hybridity in different linguistic environments.

Notably, this research establishes a critical starting point for discussing local cultural integration in EFL education, particularly in contexts where similar cultural priorities may exist. By acknowledging the potential for broader application and inviting interdisciplinary dialogue, future studies can refine the framework to

accommodate the diverse needs of EFL learners worldwide, while retaining sensitivity to regional cultural identities. This approach ensures that educational interventions can both honor local voices and foster global communicative agility.

Conclusion

This study constructs a comprehensive evaluation framework for local cultural awareness in EFL education through the Delphi-AHP methodology, translating Kramsch's symbolic competence theory into quantifiable assessment metrics. The empirically validated framework comprises three core dimensions—Local Expression and Application (45.27% weight), Local Affective Attitudes (33.01%), and Local Cognition and Understanding (21.72%)—with 18 secondary indicators, such as "Cultural Comparison" and "Local Image Promotion". Its primary innovation lies in bridging sociocultural theories (e.g., Byram's ICC model) with classroom practice by operationalizing abstract constructs into AHP-weighted evaluation criteria, addressing the overemphasis on Anglophone content in standardized curricula.

The framework offers versatile applications in EFL contexts: it supports curriculum developers in benchmarking cultural balance, enables AI-driven adaptive learning through metrics like "Cultural Pride", and assists policymakers in assessing glocal citizenship cultivation. By emphasizing local storytelling and cross-cultural comparison, the framework empowers learners as cultural agents while fostering objective cultural understanding. This research establishes a foundational model for integrating local cultural content in EFL education, laying groundwork for future

cross-cultural validation and longitudinal impact studies.

Reference

1. Gao P, Zhang X, Fu Y. Cultural confidence construction of contemporary college students: A case study of English majors. *Academic Journal of Humanities & Social Sciences*. 2023;6(18):24-28.https://doi.org/10.25236/AJHSS.2023.061805
2. Alakrash H, Edam B, Bustan E, Armnazi M, Enayat A, Bustan T. Developing English language skills and confidence using local culture-based materials in EFL curriculum. *Linguistica Antverpiensia. Google*
3. Porto M. Does education for intercultural citizenship lead to language learning? *Language, Culture and Curriculum*. 2018;32(1):16-33. https://doi.org/10.1080/07908318.2017.1421642
4. Scally J, Parrish A, Montgomery A. Intercultural competence and languages: inextricably linked or linked inexplicably? *Compare: a journal of comparative and international education*. 2022;52(3):492-510. https://doi.org/10.1080/03057925.2020.1851000
5. Byram M. *Teaching and Assessing Intercultural Communicative Competence: Revisited*. Bristol, Blue Ridge: Multilingual Matters; 2021. https://doi.org/10.21832/9781800410251
6. Zhang H, Ge S, Mohd Saad MRB. Formative assessment in K-12 English as a foreign language education: A systematic review. *Heliyon*. 2024;10(10):e31367. https://doi.org/10.1016/j.heliyon.2024.e31367
7. Risager K. *Language and Culture: Global Flows and Local Complexity*. Clevedon, UK: Multilingual Matters; 2006. Google
8. Saavedra AR, Opfer VD. *Teaching and Learning 21st Century Skills: Lessons from the Learning Sciences*. New York: Asia Society; 2012. Google
9. Vygotsky LS. *Mind in Society: The Development of Higher Psychological Processes*. Cambridge: Harvard University Press; 1978. Google
10. Tomlinson B, editor. *Developing Materials for Language Teaching*. London: Bloomsbury Publishing; 2023. Google
11. Kramsch C. *Context and Culture in Language Teaching*. Oxford: Oxford University Press; 1993. Google
12. Fan N. Exploring the effects of automated written corrective feedback on EFL students' writing quality: a mixed-methods study. *SAGE Open*. 2023;1:1–17. doi=10.1177/21582440231181296
13. Canagarajah S. *Translingual Practice: Global Englishes and Cosmopolitan Relations*. New York: Routledge; 2012.https://doi.org/10.4324/9780203073889
14. Lavrenteva E, Orland-Barak L. The treatment of culture in the foreign language curriculum: an analysis of national curriculum documents. *Journal of Curriculum Studies*. 2015;47(5):653-684. https://doi.org/10.1080/00220272.2015.1056233

15. Guo Q, Jamil H, Ismail L, Luo S, Sun Z. Effects of problem-based learning on EFL learning: A systematic review. *PLoS One*. 2024;19(12):e0307819. https://doi.org/10.1371/journal.pone.0307819
16. Yang M. Making interdisciplinary subjects relevant to students: an interdisciplinary approach. *Teaching in Higher Education*. 2009;14(6):597-606. https://doi.org/10.1080/13562510903315019
17. Chubko N, Morris JE, McKinnon DH, et al. Digital storytelling as a disciplinary literacy enhancement tool for EFL students. *Education Tech Research Dev*. 2020;68:3587-3604. https://doi.org/10.1007/s11423-020-09833-x
18. Dalton-Puffer C. Outcomes and processes in Content and Language Integrated Learning (CLIL): current research from Europe. *na*. 2008.Google Scholar
19. Lasagabaster D. English achievement and student motivation in CLIL and EFL settings. *Innovation in Language Learning and Teaching*. 2011;5(1):3–18. https://doi.org/10.1080/17501229.2010.519030
20. Margianti SF. The inclusion of local cultural contents in English classes: Teachers' perceptions. *Langue (Journal of Language and Education)*. 2022;21(1):97-105. https://doi.org/10.22437/langue.v1i1.22609
21. Wu JG, Miller L. Raising native cultural awareness through WeChat: a case study with Chinese EFL students. *Computer Assisted Language Learning*. 2019;34(4):552-582. https://doi.org/10.1080/09588221.2019.1629962
22. Gu MM, Lee JC-K. "They lost internationalization in pursuit of internationalization": students' language practices and identity construction in a cross-disciplinary EMI program in a university in China. *High Educ*. 2019;78:389-405.https://doi.org/10.1007/s10734-018-0342-2.
23. Kanoksilapatham B, Chann P. EFL learners' and teachers' positive attitudes towards local community-based instruction. *Indonesian Journal of Applied Linguistics*. 2018;7(3):504-514.Google Scholar
24. Aminullah F, Sada C, Sudarsono S. Local culture-based reading materials for EFL context. In: Proceedings of the International Conference on Teaching and Education (ICoTE). Vol 2, No 2. 2019.Google Scholar
25. Gashi L. Intercultural awareness through English language teaching: the case of Kosovo. *Interchange*. 2021;52:357-375. doi: 10.1007/s10780-021-09441-5.https://doi.org/10.1007/s10780-021-09441-5
26. Kramsch C. *Language and Culture*. Oxford: OUP Oxford; 1998. Google
27. Zhai N, Ma X. Automated writing evaluation (AWE) feedback: A systematic investigation of college students' acceptance. *Computer Assisted Language Learning*. 2022;35(9):2817-2842. https://doi.org/10.1080/09588221.2021.1897019
28. Gao M, Qian J, Rasool U. Effects of Task-Induced Involvement and Time on Task on Incidental L2 Vocabulary Acquisition. *SAGE Open*. 2024;14(2). https://doi.org/10.1177/21582440241249340
29. Winstone N, Boud D, Dawson P, Heron M. From feedback-as-information to feedback-as-process: a linguistic analysis of the feedback literature. *Assessment*

& Evaluation in Higher Education. 2022;47(2):213-230. https://doi.org/10.1080/02602938.2021.1902467

30. Gay G. Culturally responsive teaching: Theory, research, and practice. 2nd ed. New York: Teachers College Press; 2018. Google
31. Juan-Garau M, Jacob K. Developing English learners' transcultural skills through transcultural content-and task-based lessons. *System*. 2015;54:55-6. https://doi.org/10.1016/j.system.2015.04.017
32. Figueras N. The impact of the CEFR. *ELT Journal*. 2012;66(4):477-485. https://doi.org/10.1093/elt/ccs037
33. ACTFL. ACTFL proficiency guidelines 2012. https://www.actfl.org
34. Page D, Benander R. Cultural products and practices leading to cultural perspectives: Practical applications. *The Journal for Research and Practice in College Teaching*. 2016;1(1).Google Scholar
35. Deardorff DK. Identification and assessment of intercultural competence as a student outcome of internationalization. *Journal of Studies in International Education*. 2006;10(3):241-266.https://doi.org/10.1177/1028315306287002
36. Ait-bouzid H. Exploring global citizenship as a cross-curricular theme in Moroccan ELT textbooks. *Eurasian Journal of Applied Linguistics*. 2020;6(2):229-242. https://doi.org/10.32601/ejal.775801
37. Ye W, Ren W, Zheng S. The Construct of the Story Continuation Writing Task: Insights From the China's Standards of English Language Ability. *Chinese Journal of Applied Linguistics*. 2021;44(3):382-398. https://doi.org/10.1515/CJAL-2021-0024
38. Lange K. Perspectives on intercultural competence: a textbook analysis and an empirical study of teachers' and students' attitudes. *Intercultural Pragmatics*. 2011;8(2):183-208.Google Scholar
39. Wernicke M. Plurilingualism as agentive resource in L2 teacher identity. *System*. 2018;79:91-102. https://doi.org/10.1016/j.system.2018.07.005
40. McNamara T. Managing learning: authority and language assessment. *Language Teaching*. 2011;44(4):500-515. https://doi.org/10.1017/S0261444811000073
41. Hsu CC, Sandford BA. The Delphi Technique: Making Sense of Consensus. *Practical Assessment, Research, and Evaluation*. 2007;12(1):10. https://doi.org/10.7275/pdz9-th90
42. Fang F, Baker W. "A more inclusive mind towards the world": English language teaching and study abroad in China from intercultural citizenship and English as a lingua franca perspectives. *Language Teaching Research*. 2018;22(5):608-624. https://doi.org/10.1177/1362168817718574
43. Zhang D, Wu JG. Enhancing EFL learners' native cultural awareness via project-based learning. *TESL-EJ*. 2023;27(1):n1.Google Scholar
44. Dewaele JM, MacIntyre PD. The two faces of Janus? Anxiety and enjoyment in the foreign language classroom. *Studies in Second Language Learning and Teaching*. 2014;4(2):237-274. https://doi.org/10.14746/ssllt.2014.4.2.5

45. Oktarina Y, Inderawati R, Petrus I. Developing local culture-based EFL reading materials for the 21st-century learning. *Studies in English Language and Education*. 2022;9(3):1128-1147. https://doi.org/10.24815/siele.v9i3.24660
46. Polit DF, Beck CT. The content validity index: are you sure you know what's being reported? Critique and recommendations. *Research in Nursing & Health*. 2006;29(5):489-497. https://doi.org/10.1002/nur.20147
47. Saaty TL. Highlights and critical points in the theory and application of the analytic hierarchy process. *European Journal of Operational Research*. 1994;74(3):426-447.https://doi.org/10.1016/0377-2217(94)90222-4
48. Hasson F, Keeney S, McKenna H. Research guidelines for the Delphi survey technique. *Journal of Advanced Nursing*. 2000;32(4):1008-1015. https://doi.org/10.1046/j.1365-2648.2000.t01-1-01567.x
49. Schmidt RC. Managing Delphi surveys using nonparametric statistical techniques. *Decision Sciences*. 1997;28(3):763-774. https://doi.org/10.1111/j.1540-5915.1997.tb01330.x
50. Price J, Rushton A, Tyros V, Heneghan NR. Expert consensus on the important chronic non-specific neck pain motor control and segmental exercise and dosage variables: An international e-Delphi study. *PLoS One*. 2021;16(7):e0253523. https://doi.org/10.1371/journal.pone.0253523
51. Barkhuizen G. Narrative knowledging in TESOL. *TESOL Quarterly*. 2011;45(3):391-414. https://doi.org/10.5054/tq.2011.261888
52. Keles U, Yazan B. Representation of cultures and communities in a global ELT textbook: A diachronic content analysis. *Language Teaching Research*. 2023;27(5):1325-1346. https://doi.org/10.1177/1362168820976922
53. Dervin F. Interculturality in Education: A Theoretical and Methodological Toolbox. London: Palgrave Macmillan; 2016. pp. 14, 16.Google Scholar
54. Coyle D. Content and language integrated learning: towards a connected research agenda for CLIL pedagogies. *International Journal of Bilingual Education and Bilingualism*. 2007;10(5):543-562. https://doi.org/10.2167/beb459.0
55. Kramsch C. The symbolic dimensions of the intercultural. Language Teaching. 2011;44(3):354–67. https://doi.org/10.1017/S0261444810000431
56. Council of Europe. Common European Framework of Reference for Languages. Strasbourg: Council of Europe; c2023 [cited 2025 Jun 23]. Available from: https://www.coe.int/en/web/common-european-framework-reference-languages.

Supporting information

S1 Fig. Hierarchical Model of EFL Learners' Local Cultural Awareness Evaluation System.
(TIF)

S1 Table. First Delphi round consultation on secondary indicators for EFL learners' local awareness evaluation system.

(Table)

S2 Table. Second Delphi round consultation on secondary indicators for EFL learners' local awareness evaluation system.

(Table)

S3 Table. Pairwise comparison matrix for primary indicators.

(Table)

S4 Table. Pairwise comparison matrix for secondary indicators

(Table)

S5 Table. Operationalization framework for local cultural awareness assessment

(Table)

S6 Table. Index system with CEFR alignment

(Table)

S7 Table. ACTFL-OPI Integration

(Table)

Fig_1. Research Workflow Diagram

Click here to access/download;Figure;Fig1.tiff.tiff

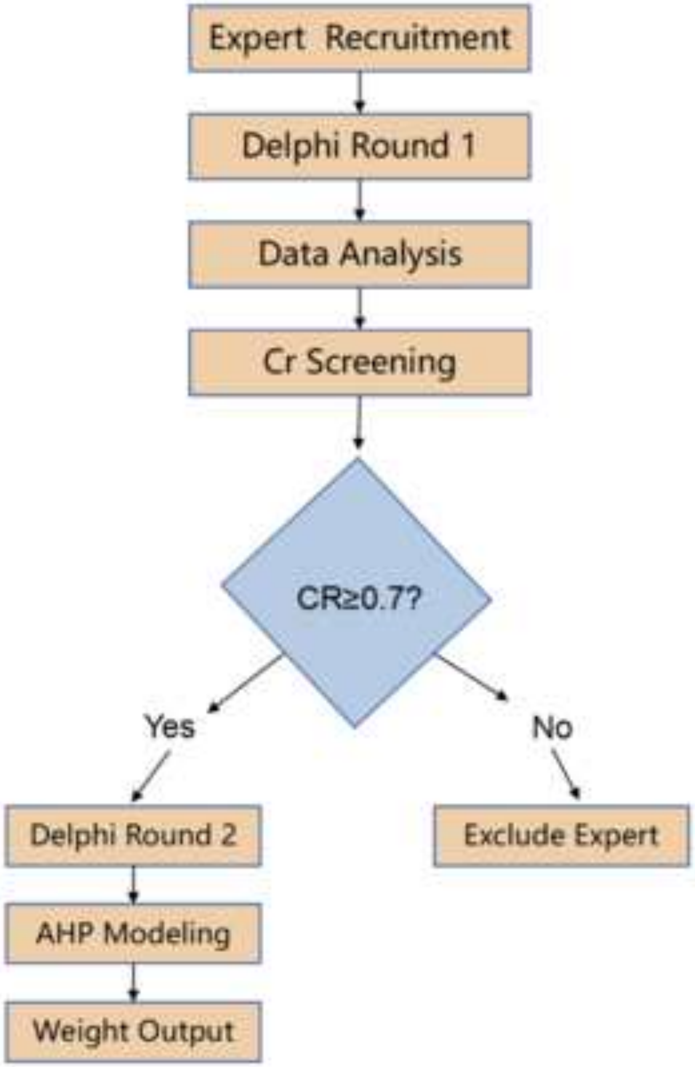

Fig_2. Weights of primary indicators for local cultural awareness assessment

Click here to access/download;Figure;Fig 2.tiff.tiff

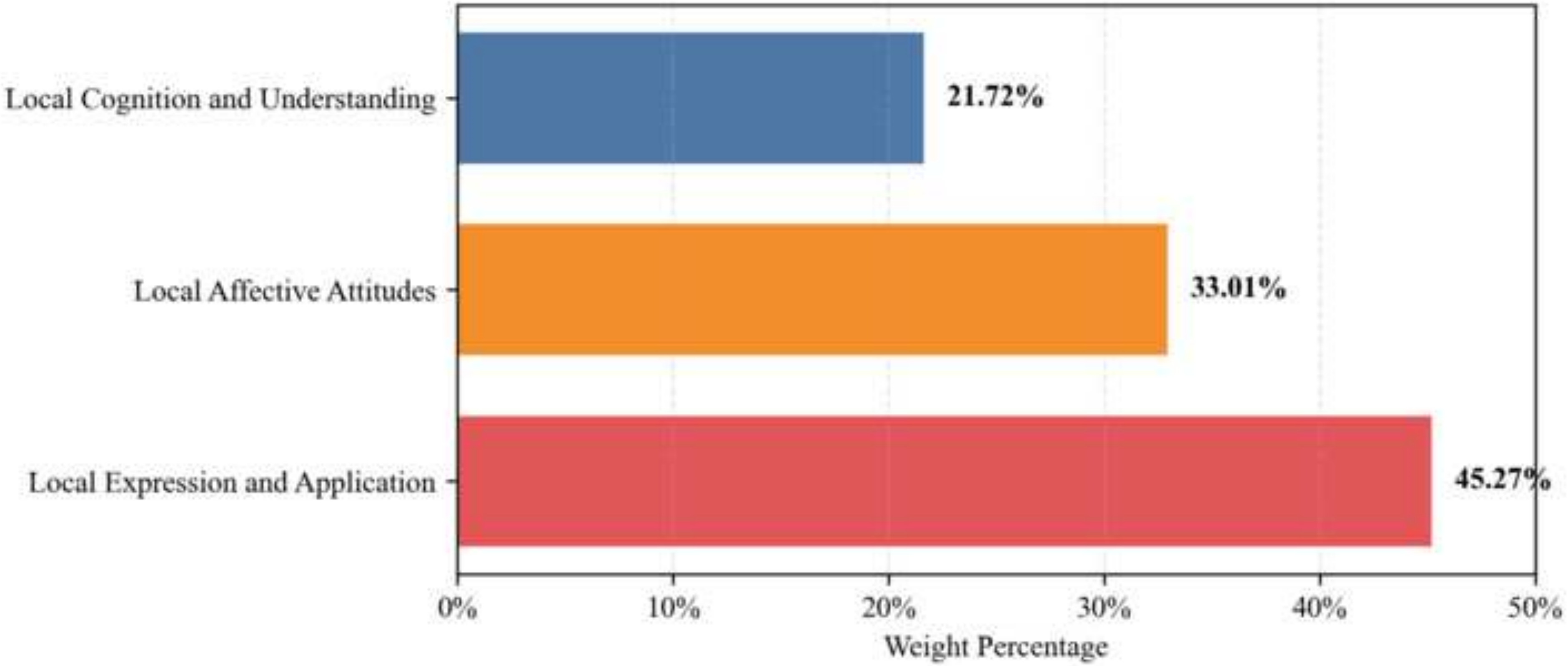

Fig_3. Secondary indicator weights in cultural awareness assessment

Click here to access/download;Figure;Fig 3.tiff.tiff

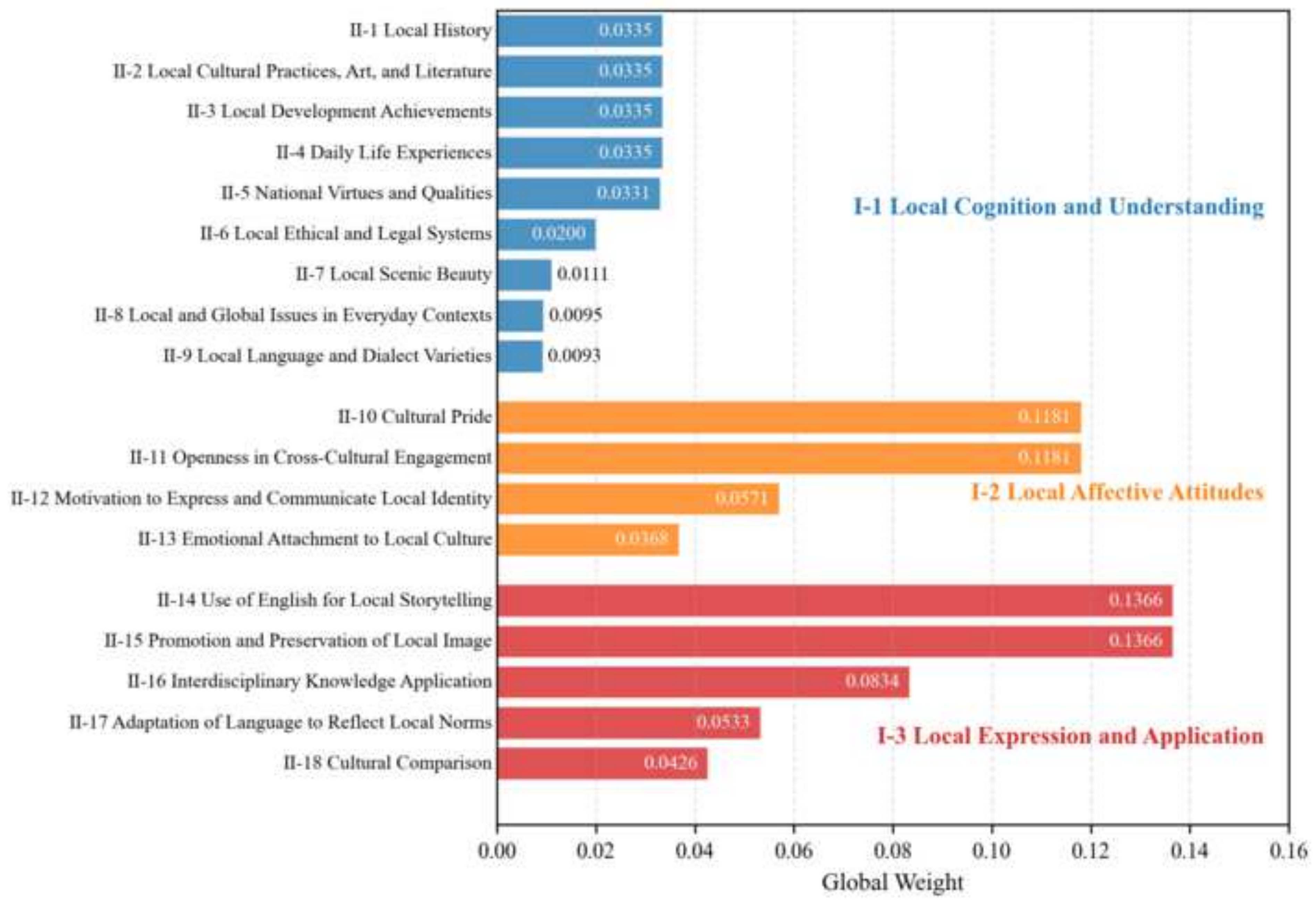

Fig_4. Schematic of the dual - track assessment process for the ACTFL OPI task

Click here to access/download;Figure;Fig 4.tiff.tiff

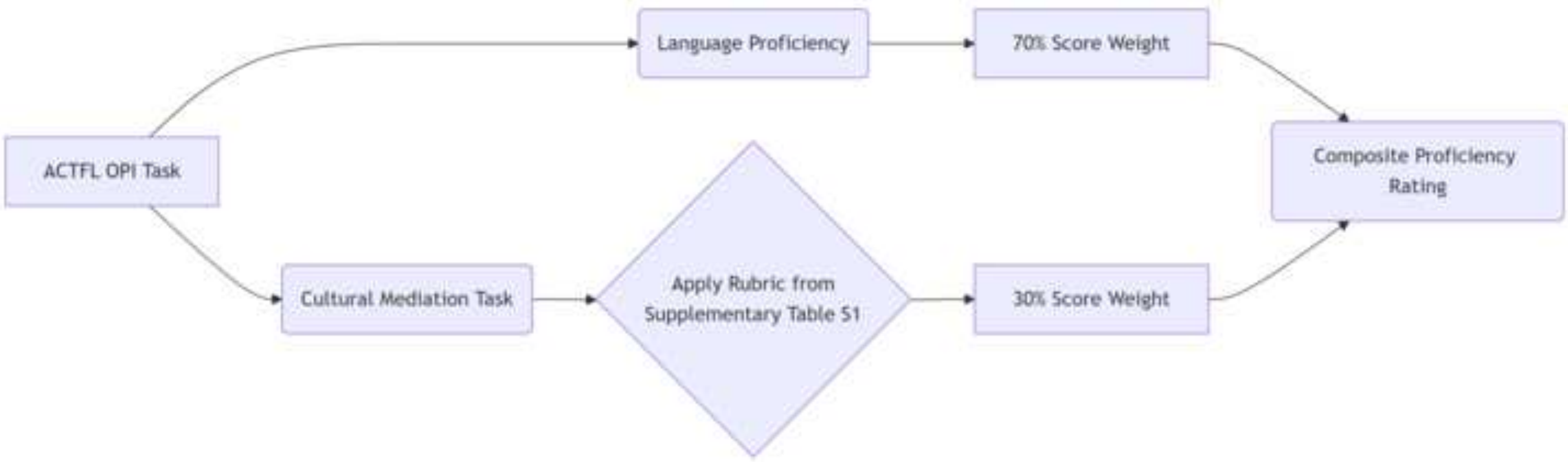

Hierarchical Model of EFL Learners' Local Cultural Awareness Evaluation System(NEW).

Click here to access/download
Supporting Information
S1_Fig.tif.tif

First Delphi round consultation on secondary indicators for EFL learners' local awareness evaluation system(NEW).

Click here to access/download
Supporting Information
S1_Table.docx

Second Delphi round consultation on secondary indicators for EFL learners' local awareness evaluation system(NEW).

Click here to access/download
Supporting Information
S2_Table.docx

Pairwise comparison matrix for secondary indicators(New).

Click here to access/download
Supporting Information
S4_Table.xlsx

Pairwise comparison matrix for primary indicators.S3 Table.
Pairwise comparison matrix for primary indicators(NEW).

Click here to access/download
Supporting Information
S3_Table.xlsx

Operationalization framework for local cultural awareness assessment

Click here to access/download
Supporting Information
S5_Table.docx

Index system with CEFR alignment

Click here to access/download
Supporting Information
S6_Table.docx

Click here to access/download
Supporting Information
S7_Table.docx

Revised Manuscript with Track Changes

Assessing local cultural awareness in university EFL learners: A Delphi and AHP-based index framework

Fan Lunan[1*] , Cheng Guiping [12], Yanna[32]

1 Department of Theoretical Education, Guizhou Police College, Guiyang, Guizhou Province, China, 2 Department of Theoretical Education, Guizhou Police College, Guiyang, Guizhou Province, China, 32 School of Foreign Languages, Guizhou Education University, Guiyang, Guizhou Province, China

*Fanlunan318@sina.com

Formatted: Font: (Default) Times New Roman

Formatted: Superscript

Formatted: Default Paragraph Font, Font: Calibri, 22 pt, Font color: Auto, Superscript, Not Expanded by / Condensed by , Kern at 22 pt, Pattern: Clear

Formatted: Font: (Default) Times New Roman

Abstract

To address the neglect of local cultural identity in standardized EFL curricula, this study develops and validates an evaluation framework for local cultural awareness in EFL education. The framework operationalizes Kramsch's symbolic competence theory into measurable dimensions, achieved through two rounds of Delphi consultations with 15 EFL education experts (Kendall's $W = 0.86$, $p < 0.01$) and one round of Analytic Hierarchy Process (AHP) analysis (consistency ratios < 0.1). The finalized index system comprises three primary dimensions—Local Expression and Application (45.27% weight), Local Affective Attitudes (33.01%), and Local Cognition and Understanding (21.72%)—alongside 18 secondary indicators. Key innovations include balancing cultural pride (0.1181) with cross-cultural openness (0.1181) and integration with CEFR/ACTFL via descriptor transplantation (e.g., mapping local storytelling to CEFR C1 "pluricultural mediation". This framework

provides educators with tools to design culturally grounded assessments and advances curriculum development by transcending Anglophone-centric models.

The aim of this study is to use the Delphi method and the analytical hierarchy process (AHP) to construct a local cultural awareness evaluation index system for English as a foreign language (EFL) learning.Addressing gaps in frameworks for prioritizing English cultural content, the study identified three key dimensions: *Local Cognition and Understanding*, *Local Affective Attitudes*, and *Local Expression and Application*, with the latter being prioritized. Under the primary indicators, secondary indicators like *Use of English for Local Storytelling* , *Promotion and Preservation of Local Image*, *Cultural Pride*, and *Openness in Cross-Cultural Engagement* were highlighted. The final index system can offer educators and researchers an effective tool for evaluating local cultural awareness in EFL learners, which can inform curriculum development and teaching practices.

Introduction

In today's globalized world, English proficiency is regarded as an essential skill for communication across borders. English as a Foreign Language (EFL) programs are increasingly designed not only to enhance learners' language skills but also to develop their ability to engage with the global community. English as a Foreign Language (EFL) programs are increasingly designed not only to equip learners with language proficiency but also to develop their ability to engage with the global

~~community.~~ Learners face “Cultural Aphasia”,a term proposed by Cong from Nanjing University in China. It describes the situation where learners, despite having a high proficiency in a foreign language, find it challenging to convey the rich and unique aspects of their native culture[1].~~Learners face "Cultural Aphasia", a term describing their struggle to articulate local identities through foreign language discourse.~~ This linguistic-cultural disjuncture has catalyzed reforms in EFL curricula, where there is growing recognition of the importance of integrating localized content into language learning to make it more relevant and meaningful to learners' lived experiences [2, 3].

Although the advantages of integrating local elements into the EFL curriculum have gained more attention, it is still difficult to effectively measure the extent to which learners demonstrate local identity in the use of the English language. At present, most of the evaluation systems excessively focus on the assessment of language skills and cross-cultural communication ability. For example, frameworks such as the CEFR are effective in benchmarking grammatical accuracy. However, they inadequately capture learners' ability to articulate culturally - grounded narratives. This lack of assessment is particularly prominent in areas with significant cultural diversity, because the local cultural characteristics profoundly affect the actual application scenarios of language.

Given the importance of evaluating local cultural awareness in EFL learning, this study aims to develop a comprehensive evaluation index system. And the system will focus on three key dimensions: cognitive, affective, and behavioral. Such dimensions have been identified in previous research as critical components

of language learning and intercultural competence [04, 0]. In particular, the cognitive dimension will measure learners' understanding of local cultural, social, and historical contexts, and their ability to express this knowledge in English. The affective dimension will evaluate learners' attitudes toward expressing local content, including their emotional connection to their region and their motivation to share local stories. Finally,the behavioral dimension mainly focuses on how learners use English to express and spread local culture in practical communication.

The evaluation system constructed by this research will serve as a valuable resource for educators, enabling them to evaluate how well learners can interact with and represent their local culture when using English. The goal is to promote a comprehensive approach to language learning that integrates both global and local perspectives, fostering learners who can confidently and skillfully interact within diverse cultural contexts.

Literature Review

Local cultural awareness in EFL learning

~~Local Cultural Awareness in EFL Learning~~

Local cultural awareness in EFL learning refers to the conscious integration of learners' local context into the language learning process. The idea of embedding local content in EFL curricula stems from the understanding that language learning should not be isolated from the socio-cultural background.[67, 0]. Rooted in

sociocultural theory from Vygotsky [0], local cultural consciousness emphasizes the importance of situational sensitivity. Educators need to be able to recognize and respond to the complex interactions within the sociocultural context in which learners are situated, such as language use, historical background, and community connections. By utilizing cultural symbols and designing collaborative learning activities, educators can help students integrate theoretical knowledge with their local experiences, forming a meaningful knowledge network. This approach aims to foster the co-development of students' cognitive growth, cultural identity, and critical thinking skills [9]. Tomlinson [0] conceptualizes it as a dynamic metacognitive skill that enables learners to "notice, analyze, and negotiate linguistic hybridity" , such as code-switching patterns in multilingual communities. This construct extends beyond passive cultural literacy.

The theoretical framework of local cultural awareness is also rooted in other disciplines. Under the model of cultural ecology, Kramsch [0] put forward the concept of "third space", believing that EFL classroom should be a place for dynamic negotiation between local culture and global culture. Fan [12] provides empirical evidence that aligns with Kramsch's perspective, demonstrating how structured feedback in EFL writing can enhance cultural articulation. This suggests that feedback mechanisms can play a crucial role in helping learners develop cultural awareness and effectively express their local identities in English.

From the perspective of intercultural communication theory, Byram [0] "Intercultural competency model" emphasizes that EFL learners need to develop

critical reflection by comparing local cultures with target cultures. Integrating research from multilingual studies and sociolinguistics, Canagarajah[0] advocates that multilingual speakers incorporate their own languages and values into English. This integration initiates various negotiation strategies that assist them in interpreting other distinct varieties of English and in constructing new norms.

Cognitive and ~~I~~interdisciplinary ~~D~~demands in ~~T~~tertiary-~~L~~level EFL ~~E~~education

English plays a vital role in cultural integration within foreign language education, making cultural inclusion in TEFL instruction critically important [0]. Contemporary university-level EFL teaching requires combining higher-order thinking (HOT) with cross-disciplinary cognitive skills. Advanced learners at this level must move beyond basic language mastery to demonstrate analytical abilities for processing complex, multi-subject content [0]. Integrating HOT into curricula pushes students past memorization, developing their critical analysis, problem-solving, and information synthesis across disciplines [0,[0]. Learners tackle interdisciplinary challenges in English, demanding knowledge integration from multiple fields to form sophisticated responses [0]. For example, Chubko et al.[0] showed how digital storytelling (DST) improves STEM literacy by merging language skills with technical knowledge and social identity expression, proving cross-subject methods' value in EFL contexts.

Moreover, studies in Content and Language Integrated Learning (CLIL)

confirms HOT and interdisciplinary skills' necessity for advanced EFL education. CLIL approaches emphasize the teaching of both content and language simultaneously. By encouraging learners to use English as a medium to acquire knowledge from subjects such as history, geography, and science, both language proficiency and cognitive development of EFL learners was strengthened [0,0]. Complex cognitive skills such as critical analysis, reflection, and synthesis are required,building competencies that correspond to modern educational goals.

Formatted: Space After: 0 pt, Font Alignment: Auto, Pattern: Clear

Benefits of ~~L~~local ~~C~~content ~~I~~integration in EFL ~~I~~instruction

Despite the importance of local content in language learning, EFL programs have traditionally emphasized the cultures of English-speaking countries, which can lead to a disconnect between learners and their local realities. Indonesian junior high textbooks exemplify this issue, prioritizing Western cultural references while neglecting regional traditions [0]. This imbalance leaves students more familiar with foreign customs than their own, limiting discussions about local values. Such detachment frames English as an external code rather than a meaningful communication tool, potentially reducing motivation and hindering acquisition [0].

~~Emerging studies highlight local culture integration's positive impacts on EFL outcomes. Kanok-Xirapathum and Chanu [21] observed Thai students responding positively to lessons incorporating community content. Gay [22] emphasized that culturally relevant teaching strengthens language skills by validating learners'~~

backgrounds. These findings demonstrate how localized approaches enhance engagement, motivation, and proficiency. Emotional benefits are also evident, as students demonstrate greater motivation when local culture is integrated into learning, as shown by Aminullah et al. [23] in Indonesia and Gashi [24] in Kosovo. Porto [] observed that local cultural narratives in Argentine English curricula improve language skills and foster cultural pride. From a cognitive perspective, Kramsch [25] emphasized the inseparability of language and culture, noting that language learning is not only about acquiring linguistic forms but also about understanding cultural meanings, which deepens the cognitive process of language acquisition.

Formatted: Font color: Auto

Formatted: Font color: Red

Moreover, research indicates that integrating local content enhances intercultural competence and practical language skills. Canagarajah [12] holds that navigating both local and global cultures through English enhances learners' communicative competence, while Juan-Garau and Jacob [26] show that when learners engage with real-world, culturally relevant scenarios, they develop practical communication skills. Wu and Miller [27] demonstrated that Chinese EFL learners improved intercultural skills by using WeChat to discuss regional festivals and traditions. Their study showed how digital platforms can connect language practice with cultural knowledge application. Gu and Lee [28] highlight the need to blend international and local viewpoints in teaching. This dual focus allows students to apply English meaningfully in both global exchanges and community contexts. These collective findings confirm that effective EFL instruction requires

balancing worldwide communication needs with culturally rooted learning experiences.

Emerging studies highlight local culture integration's positive impacts on EFL outcomes. Gu and Lee [22] highlight the need to blend international and local viewpoints in teaching. This dual focus allows students to apply English meaningfully in both global exchanges and community contexts.Kanok-Xirapathum and Chanu [23] observed Thai students responding positively to lessons incorporating community content. Emotional benefits are also evident, as students demonstrate greater motivation when local culture is integrated into learning, as shown by Aminullah et al. [24] in Indonesia and Gashi [25] in Kosovo. Porto [0] observed that local cultural narratives in Argentine English curricula improve language skills and foster cultural pride.

From a cognitive perspective, Kramsch [26] emphasized the inseparability of language and culture, noting that language learning is not only about acquiring linguistic forms but also about understanding cultural meanings, which deepens the cognitive process of language acquisition.This aligns with findings that the cognitive feedback provided by digital tools can significantly impact learners' cognitive development and application of cultural knowledge in language learning [27]. Gao et al.[28] further demonstrate that task-induced involvement and time on task can significantly influence vocabulary acquisition in L2 learners, suggesting that culturally-relevant tasks enhance cognitive engagement and cultural knowledge integration. Recent research reinforces this idea by highlighting how feedback

mechanisms can enhance learners' cognitive engagement with cultural content, thereby enriching their understanding and application of cultural knowledge in language learning contexts [29].

Moreover, research indicates that integrating local content enhances intercultural competence and practical language skills [30]. Canagarajah [13] holds that navigating both local and global cultures through English enhances learners' communicative competence, while Juan-Garau and Jacob [31] show that when learners engage with real-world, culturally relevant scenarios, they develop practical communication skills. Wu and Miller [21] demonstrated that Chinese EFL learners improved intercultural skills by using WeChat to discuss regional festivals and traditions. Their study showed how digital platforms can connect language practice with cultural knowledge application.

These findings confirm that effective EFL instruction requires balancing worldwide communication needs with culturally rooted learning experiences.

Gaps in ~~C~~current ~~A~~assessment ~~F~~frameworks

Although integrating local content into EFL curricula shows clear benefits, measuring learners' cultural engagement through English remains challenging. Widely used evaluation frameworks, such as the Common European Framework of Reference for Languages (CEFR), focus primarily on assessing linguistic proficiency and intercultural competence but do not adequately address the assessment of local cultural awareness [0]. For example, the CEFR's "can do" description focuses on

specific language tasks that learners can accomplish at different language levels. While these standards partially address cross-cultural awareness, they lack tools to evaluate how students articulate their cultural identities in English [0].

This limitation extends to other widely adopted frameworks. The ACTFL Proficiency Guidelines [0], while emphasizing task-based performance in real-world contexts, primarily measures learners' ability to "describe local cultural practices" as a sub-skill of interpersonal communication, without establishing explicit criteria for evaluating the depth or authenticity of cultural identity expression ~~[30]~~[**Error! Reference source not found.**][**Error! Reference source not found.**]. Similarly, UNESCO's Global Citizenship Education Framework (2015), though containing a socio-emotional domain that addresses "sense of belonging to local communities," lacks operationalized indicators for assessing how learners articulate their cultural roots through language [0].

Even frameworks explicitly incorporating cultural dimensions exhibit gaps. China's Standards of English Ability (CSE) mandate "introducing Chinese traditional culture in English" as a competence requirement, but its assessment rubrics remain anchored in linguistic accuracy rather than cultural authenticity [0]. The German National Educational Standards, while integrating intercultural competence as a curricular component, have not adequately addressed the assessment of local cultural awareness. Existing assessment frameworks predominantly focus on the comparison of cultural differences and the understanding of target cultures, neglecting the evaluation of learners' awareness and expression of their own local culture [0].

These gaps stem from two structural limitations in current frameworks. Firstly, most separate linguistic competence from cultural identity assessment. For instance, CEFR's pluricultural competence descriptors (e.g., "mediating cultural ambiguities") remain isolated from language production scales. Secondly,frameworks like CEFR measure adaptability to external cultures but fail to capture the dynamic process of reconstructing local identity through L2 [0] .

Recent attempts to address these gaps through CEFR companion volumes show limited progress. While introducing "mediation scales" for cultural interpretation, they still lack descriptors for self-referential cultural expression. As McNamara [0] critically notes, "The dominant frameworks reduce culture to a set of learnable behaviors rather than a lived identity that interacts with language acquisition" .

To address this gap, this study aims to establish a comprehensive evaluation index system through a series of surveys using the Delphi method, allowing researchers to analyze the opinions of expert panels by collecting, combining and quantifying multiple opinions on complex issues. By creating a tailored assessment system, this study aims to contribute to the growing body of literature on English learning by providing educators with a tool to assess learners' ability to integrate into local culture through English. This framework will enable educators to assess not only language proficiency, but also the learners' ability to use English as a medium to express their local identity. Ultimately, the goal is to promote a more comprehensive approach to language learning that values both global and local perspectives and fostering learners who have the confidence and ability to navigate different cultural

environments.

Methods

Participants

In accordance with the Delphi method guidelines[0] that suggest a sample size of 15-20 experts for consensus - driven studies, 15 EFL educators (**Table 1**) were invited to participate in the study and formed the expert panel. The recruitment period for this study spanned from November 30, 2023, to March 1, 2024. These 15 participants were recruited from a variety of academic institutions across China, which guaranteed geographical and institutional diversity. All participants provided written informed consent, and the study was approved by the Institutional Review Board of GPC College (GZPC20231127).**To ensure the maintenance of expert anonymity during the Delphi rounds, the survey materials explicitly guaranteed strict confidentiality . Experts were not made aware of each other's identities, and all responses were collected and handled to ensure full anonymization throughout the process.**

The expert panel was composed of EFL specialists who were required to meet the following three selection criteria: (1) a minimum of 10 years' experience in EFL curriculum design; (2) research conducted in the field of EFL; and (3) holding senior professional titles such as Professor or Associate Professor. This rigorous selection process ensured that the panel's input was both authoritative and relevant to the study's objectives.

Table 1. Demographic ~~C~~characteristics of ~~E~~expert ~~P~~participants (N=15)

Category	Subgroup	n	Category	Subgroup	n
**Age**	≤35 years	4	**Research experience**[a]	Extensive	9
	36-45 years	9		Moderate	5
	46-55 years	2		Limited	1
**Years of EFL teaching experience**	10-19 years	13	**Teaching experience**[b]	Extensive	11
	20-30 years	2		Moderate	4
**Highest degree**	Ph.D.	13	**Current teaching position**	Full Professor	10
	Master's	2		Associate Professor	5

[a] Research experience criteria: Extensive (≥10 publications), Moderate (5-9), Limited (<5).

[b]Teaching experience criteria: Extensive (≥10 years), Moderate (5-9), Limited (<5).

Formatted: Normal, Line spacing: Double

Preliminary ~~E~~establishment of ~~I~~indicators

To comprehensively and systematically assess the awareness of native culture among advanced foreign language learners, this study adopted a multi-method approach to construct an initial set of indicators. First, researchers searched the Web of Science database using keywords such as "foreign language native awareness," "cultural competence assessment," and "place-based pedagogy." This search aimed to identify existing literature relevant to the topic, providing a solid theoretical foundation for the establishment of indicators. Concurrently, two rounds of focus group discussions were conducted with the research team members (all front-line foreign language teachers at universities) to clarify concepts, perform redundancy

checks, ensure domain coverage, and verify operational feasibility. These steps are crucial for refining the initial set of indicators, ensuring that the selected indicators are not only conceptually sound but also practically feasible and applicable in the context of assessing the awareness of native culture among advanced foreign language learners.

The assessment framework was grounded in a tripartite model of cultural competence [0, 0], which posits cultural competence as a synthesis of cognitive, affective, and behavioral dimensions. The selection of this model is justified by its holistic capacity to capture both internal development (e.g., knowledge and attitudes) and external manifestations (e.g., communicative behaviors) of cultural awareness, a duality critical for evaluating EFL learners' ability to navigate local-global identity tensions [0]. Specifically, the cognitive dimension focuses on learners' knowledge and understanding of local cultural, social, and historical contexts and their ability to express this knowledge in English, as measured through indicators such as cultural schema activation and contextualized language production [0,0]. The affective dimension, in turn, targets learners' attitudes toward local-global hybridity, with affective indicators measuring their interest in using English as a tool for self-expression and their pride in local cultural narratives [0]. Finally, the behavioral dimension assesses how learners utilize their knowledge of local content in practical settings, highlighting adaptive communication strategies that conform to local linguistic standards and social customs[45]. Cultural understanding, attitude formation, and adaptive communication define key dimensions. They serve as organizing

principles for categorizing indicators. Furthermore, the tripartite structure aligns with contemporary pedagogical goals of fostering not only linguistic proficiency but also intercultural citizenship, enabling learners to act as mediators between their local context and global discourse [3].Finally, the behavioral dimension assesses how learners utilize their knowledge of local content in practical settings, highlighting adaptive communication strategies that conform to local linguistic standards and social customs.[41]. These dimensions collectively characterized by cultural understanding (cognitive), attitude formation (affective), and adaptive communication (behavioral) — served as the organizing principle for indicator categorization. Furthermore, the tripartite structure aligns with contemporary pedagogical goals of fostering not only linguistic proficiency but also intercultural citizenship, enabling learners to act as mediators between their local context and global discourse [2].

Guided by the theoretical synthesis, a preliminary evaluation system comprising three primary indicators was developed. Underpinning these primary indicators, 24 secondary indicators were formulated for the initial consultation phase (S1Table). Specifically, under the *Local Cognition and Understanding* primary indicator, there are 14 secondary indicators including *Local History*, *National and Regional Geography*, and *Local Cultural Practices, Art, and Literature*; under the *Local Affective Attitudes* primary indicator, there are 4 secondary indicators including *Motivation to Express and Communicate Local Identity*, *Cultural Pride*, and *Oneness in Cross-Cultural Understanding*; under the *Local Expression and Application*

indicator, 6 secondary indicators were initially proposed, such as *Cultural Comparison Skill*, *Contextualized Language Use*, and *Promotion and Preservation of Local Image*.

Procedures

In this study, three rounds of expert consultations were conducted using paper-based inquiry materials. The first two rounds conformed to the Delphi procedure, while the final round adopted the Analytic Hierarchy Process (AHP). The survey procedures and analysis stages are shown in Fig 1.

Fig 1. Research Workflow Diagram

~~In this study, three rounds of expert consultations (S 1 Fig.) were conducted via email.~~

Delphi p~~P~~rocedure. The Delphi method was used to gather expert opinions on the key indicators for assessing local cultural awareness in EFL learners. The method involved organizing several rounds of questionnaires to solicit feedback from experts in the field of language education~~, shown in S 1 Fig~~. Through iterative surveys and feedback processes, consensus on specific evaluation indicators was sought. Anonymity in the process allowed for more candid expert feedback, while the statistical analysis provided insight into the central tendencies and variances in expert opinions. These characteristics make the Delphi method particularly effective for educational research where diverse expert perspectives are required [0].

Formatted: Indent: First line: 2 ch, Line spacing: Double

Formatted: Line spacing: Double

Formatted: Font: Not Bold

In the first round, opinions were collected from experts on the necessity of the three primary indicators and 24 secondary indicators. The analysis involved descriptive statistics, including the calculation of the mean (M) and standard deviation (SD) to validate initial indicators. This survey lasted for about 4 weeks, during which experts were asked to use a 5-point scale to evaluate each indicator, ranging from "not important" to "very important". In this round of evaluation, participants also rated their familiarity with evaluation criteria (Cs) and described their judgment rationale (Ca). The basis for judgment (Ca) includes practical experience, logical reasoning, and an understanding of domestic and international language education trends. These self-assessments help ensure that the feedback provided is based on a well-informed perspective. The formula for calculating the expert authority coefficient Cr is:

~~Cr = (Ca + Cs) / 2.~~ $Cr = \frac{Ca+Cs}{2}$

It is generally believed that when the expert authority coefficient Cr is greater than or equal to 0.7 [0], the research results are considered reliable. The assignments for Ca and Cs are shown in **Table 2** and

Table 3 respectively.

Table 2. Assignment for ~~E~~expert ~~J~~judgment ~~B~~basis (Ca)

**Judgment Basis**	**Impact Level**		
	High Impact	Moderate Impact	Low Impact
Deductive Reasoning	0.3	0.2	0.1
Practical Experience	0.5	0.4	0.3
Understanding of Domestic and International Language Education Trends	0.1	0.1	0.1
Intuition	0.1	0.1	0.1
Total	1.0	0.7	0.6

Field Code Changed

Formatted: Font: Cambria Math

Formatted: Font: Cambria Math

Formatted: Font: Cambria Math

Table 3. Familiarity ~~C~~coefficient ~~A~~assignment (Cs)

Familiarity Level	Coefficient (Cs)
Extremely Familiar	1.0
Very Familiar	0.75
Moderately Familiar	0.50
Slightly Familiar	0.25
Not Familiar	0.00

The second round focused on revising and refining the indicators based on the feedback from the first round. A revised version of the assessment system was created for this round. After collecting and analyzing the feedback from the first round, indicators considered irrelevant or meaningless were removed. Researchers, based on the degree of concentration and coordination of expert opinions, re-sorted the remaining indicators and revised the content of the consultation questionnaire to ensure its accuracy and applicability. Indicators with low consensus ($M < 3.5$, $SD > 1.0$, $CV > 25\%$) were eliminated to ensure a higher level of agreement among experts.

Analytic Hierarchy Process (AHP). The final round was dedicated to confirming consensus among experts and constructing the Analytic Hierarchy Process (AHP) hierarchy. The AHP was employed to assign weights to the various indicators of local cultural awareness based on expert judgment. By breaking down the complex task of evaluating learners' local cultural awareness into multiple hierarchical levels, AHP allows for precise pairwise comparisons of factors such as Local Cognition and Understanding, Local Affective Attitudes, and Local Expression and Application. These comparisons helped determine the relative importance of each indicator within the overall evaluation framework. The use of the Saaty 1~9 scale [0] in AHP provided

Formatted: Font: Not Bold

a structured approach for experts to rate the importance of each indicator, ensuring that the weight distribution reflects the collective expert judgment.

To ensure the reliability of the pairwise comparisons, the consistency ratio (CR) was calculated using the formula CR = CI/RI, where CI is the consistency index and RI is the random consistency index. According to Saaty's criteria, a CR value less than 0.1 indicates acceptable consistency. This verification step ensures that the judgments made by experts are logically consistent and free from significant biases or errors.

Results

Expert ~~A~~authority ~~A~~analysis

As shown in **Table 1**, the participants all have a high educational background, 87% held doctoral degrees with mean experience of 15.9 years (SD=4.01). Rich experience in university foreign language teaching, and good professional representation, which can provide valuable information for the structural rationality of the construction of the index system.

In the three rounds of discussion and consultation, 15 experts all participated in the whole consultation process, and the recovery rate of the questionnaire was 100%, showing the high participation and input of the participants, and providing a strong support for the promotion of the research. At the same time, the participants conducted self-evaluation on the judgment basis of the problem and the familiarity with the problem. After calculation, the expert authority coefficient (Cr) was 0.867,

indicating that the experts had a high degree of familiarity with the indicators and a high authority in the judgment of the indicators, and the consultation results have practical reference significance [0]. The authority coefficient of the experts is shown in **Table 4**.

Table 4. Authority ~~C~~coefficients of 15 ~~E~~experts

Expert Number	Familiarity Coefficient (Cs)	Basis of Judgment (Ca)	Authority Coefficient (Cr)
1	1.00	1.00	1.000
2	0.75	0.90	0.825
3	0.75	1.00	0.875
4	1.00	0.90	0.950
5	1.00	0.70	0.850
6	0.50	0.90	0.700
7	1.00	0.70	0.850
8	0.75	1.00	0.875
9	0.75	0.90	0.825
10	1.00	1.00	1.000
11	0.75	0.70	0.725
12	0.75	1.00	0.875
13	0.75	1.00	0.875
14	1.00	0.90	0.950
15	0.75	0.90	0.825
Mean	0.83	0.90	0.867

Notes:

1. Cr is calculated as Cr=(Cs+Ca)/2.
2. The mean Cr value is derived from precise calculations (retaining four decimal places) before rounding.
3. A Cr value ≥ 0.7 indicates high reliability of expert opinions.

Consensus ~~D~~development ~~A~~analysis

To assess the degree of agreement among the experts, the Kendall coordination coefficient (W) was calculated. This non-parametric statistic was used to measure the consistency of a group of judges or raters on the items they ranked. The Kendall's W

coefficient and chi-square tests revealed significant consensus improvement across Delphi rounds (**Table 5**).

Table 5. Expert ~~C~~consensus ~~A~~analysis ~~A~~across Delphi ~~R~~rounds

Round	Experts (n)	Indicators (k)	Kendall's W	χ^2(df)	p-value	Interpretation
1	15	24	0.634	267.63 (23)	<0.001	Moderate consensus
2	15	19*	0.855	217.9 (18)	<0.001	Strong consensus

Notes:
1.*5 indicators eliminated after Round 1 screening
2.Degrees of freedom (df) calculated as df=k−1.

Formatted: Line spacing: single

The moderate consensus (W=0.634) in Round 1 reflects expected divergence during initial indicator exploration. The significant χ^2 value ($p<0.001$) nevertheless confirms non-random agreement patterns, warranting progression to refinement rounds [0].

The second round of expert consultation yielded a Kendall's coordination coefficient W=0.855 and a p-value of <0.001. According to prior studies, a Kendall's W value between 0.6 and 0.8 [0] typically indicates strong consensus among experts. While the observed value (0.855) slightly exceeds this range, it further demonstrates an exceptionally high level of agreement among participants in the second round, confirming the reliability of the refined results. Notably, the W coefficient increased by 34.8% (from 0.634 in Round 1 to 0.855 in Round 2), demonstrating the Delphi method's efficacy in reconciling expert perspectives through iterative, structured feedback. This significant improvement highlights the robustness of the consensus-building process in narrowing divergent opinions and achieving actionable

outcomes.

First ~~D~~delphi survey results

In the first round of the Delphi survey, 15 experts evaluated a preliminary set of 24 secondary indicators across three primary dimensions (Cognitive, Affective, Behavioral). Statistical thresholds (Mean M ≥ 3.5, Standard Deviation SD < 1, Coefficient of Variation CV < 0.25) were applied to assess indicator necessity. As is shown in

Table 6, 17 indicators (M_{range}=3.87-5.00, SD_{range}=0.41-0.83, CV_{range}=0.08-0.21) met all criteria and were retained for the second round.

Table 7 shows that 7 indicators failed to meet thresholds, primarily due to low relevance (M<3.12)or high opinion divergence(SD>1.01).

Table 6. Retained ~~I~~indicators ~~A~~after ~~F~~first Delphi ~~R~~round (n=17)

Indicator Name	Mean (M)	SD	CV
Local History	4.47	0.74	0.17
Local Ethical and Legal Systems	4.07	0.59	0.15
Regional Geography	3.87	0.83	0.22
Local Cultural Practices, Art, and Literature	4.80	0.56	0.12
Local Development Achievements	4.67	0.62	0.13
National Virtues and Qualities	4.80	0.41	0.09
Daily Life Experiences	4.67	0.49	0.10
Local Language and Dialect Varieties	3.87	0.52	0.13
Motivation to Express and Communicate Local Identity	4.80	0.41	0.09
Emotional Attachment to Local Culture	4.13	0.64	0.15
Cultural Pride	5.00	0.00	0.00
Openness in Cross-Cultural Engagement	5.00	0.00	0.00
Cultural Comparison	4.00	0.65	0.16
Interdisciplinary Knowledge Application	4.67	0.49	0.10

Adaptation of Language to Reflect Local Norms	4.07	0.88	0.22
Use of English for Local Storytelling	5.00	0.00	0.00
Promotion and Preservation of Local Image	4.60	0.63	0.14

Table 7. **Unretained / ~~M~~modified ~~I~~indicators ~~A~~after ~~F~~first ~~D~~delphi ~~R~~round (n=7)**

Indicator Name	Mean (M)	SD	CV	Action Taken
Local Political System and Governance	1.40	0.63	0.45	Removed
Local Economic System	3.07	0.70	0.23	Removed
Local Social Issues	2.88	1.06	0.37	Integrated into a new indicator
International Relations and Global Issues	2.93	0.80	0.27	Removed
Local Educational System	3.13	1.25	0.40	Removed
Local Environment and Sustainable Development	3.00	0.93	0.31	Merged into A4 Local Development Achievements
Participation in Culturally Relevant Discussions	2.27	0.70	0.31	Removed

Removal of ~~R~~redundant ~~I~~indicators.~~:~~ *Local Political System and Governance* (M = 1.40) and *Participation in Culturally Relevant Discussions* (M = 2.27) were excluded due to low relevance and measurement challenges.

Revisions ~~B~~based on ~~F~~feedback. *Local Economic System* (M=3.07), *Local Social Issues* (M=2.88), *International Relations and Global Issues* (M=2.93) and *Local Educational System* (M=3.13) were advised to consolidate into a new indicator "Local and Global Issues in Everyday Contexts" under the primary indicator *Local Cognition and Understanding*, in order to focus on practical topics (e.g., community governance, local economic challenges). *Environment and Sustainable Development* (M=3.00) were suggested to merge into then indicator *Local Development Achievements*.

Descriptive ~~A~~adjustments. *Cognition of National and Regional Geography* was renamed *Cognition of Local Scenic Beauty* to emphasize cultural connections (e.g., linking landscapes to festivals). At the same time, several experts recommended revising the descriptions of the indicators related to local history, as well as the ethical and legal systems. These revisions aim to align more closely with the objectives of EFL education and to ensure that the assessment is both practical and operational.

The revised indicator set was advanced to the second Delphi round for further validation.

Second ~~D~~delphi survey results

The second Delphi survey (S2Table) assessed expert consensus on the appropriateness of 18 indicators across three dimensions. All indicators met retention criteria (M ≥ 3.5, SD < 1, CV < 0.25), with mean values ranging from 3.93 to 5.00, standard deviations ranging from 0.00 to 0.45, and coefficients of variation ranging from 0.08 to 0.09, confirming high agreement among experts. The results are presented below, organized by dimension.

Local ~~C~~cognition and ~~U~~understanding. Experts exhibited strong consensus on indicators under this dimension (**Table 8**). Two indicators—*Local Cultural Practices, Art, and Literature* and *National Virtues and Qualities*—achieved perfect scores (M = 5.00, SD = 0.00, CV = 0.00), reflecting unanimous agreement on their necessity for evaluating learners' cultural knowledge. Notably, Daily Life Experiences (M = 5.00, SD = 0.00) and Local Development Achievements (M=4.93, SD=0.26) also demonstrated high reliability (CV < 0.05), highlighting their role in connecting abstract cultural concepts to tangible contexts.

Table 8. Two-~~R~~round Delphi ~~S~~survey ~~R~~results for ~~S~~secondary ~~I~~indicators under the ~~P~~primary ~~I~~indicator *Local Cognition and Understanding*.

Secondary Indicator	Mean (out of 5)		SD		CV		Action Taken
	1st	2nd	1st	2nd	1st	2nd	
Local History	4.47	4.93	0.74	0.26	0.17	0.05	Retained
Local Scenic Beauty	3.87	3.93	0.83	0.26	0.22	0.07	Retained
Local Cultural Practices, Art, and Literature	4.80	5.00	0.56	0.00	0.12	0.00	Retained
Local Development	4.67	4.93	0.62	0.26	0.13	0.05	Retained

Formatted: Font: Italic

Formatted: Font: Italic

Achievements							
National Virtues and Qualities	4.80	5.00	0.41	0.00	0.09	0.00	Retained
Daily Life Experiences	4.67	5.00	0.49	0.00	0.10	0.00	Retained
Local Language and Dialect Varieties	3.87	3.93	0.52	0.26	0.13	0.07	Retained
Local Ethical and Legal Systems	4.07	4.73	0.59	0.46	0.15	0.10	Retained
Local and Global Issues in Everyday Contexts	–	3.93	–	0.26	–	0.07	Retained

When looking at the improved indicators, *Local History* showed significant progress, with its mean increasing from 4.47 (SD = 0.74) in the first round to 4.93 (SD = 0.26) in the second round, and its CV decreasing from 0.17 to 0.05. This improvement suggests that the revised description of the indicator better aligns with EFL learning objectives. Similarly, *Local Ethical and Legal Systems* progressed from moderate consensus (M = 4.07, CV = 0.15) to higher agreement (M = 4.73, CV = 0.10), indicating enhanced clarity in its operational definition.

Regarding the newly added indicator, *Local and Global Issues* in Everyday Contexts (M = 3.93, SD = 0.26, CV = 0.07) was introduced in the second round to address practical cultural engagement. Although its mean was slightly lower compared to other retained indicators, it met all statistical thresholds, justifying its inclusion in the final evaluation system.

Local ~~A~~affective ~~A~~attitudes. As is shown in **Table 9,** the appropriateness of indicators related to the "emotion" dimension as reflected by experts in two rounds of surveys is shown in Table 8. All affective indicators received strong endorsement.

Cultural Pride (M = 5.00, SD = 0.00, CV = 0.00) and Critical Openness in

Formatted: Font: (Default) Times New Roman, 12 pt, Not Bold, Font color: Gray-75%

Formatted: Normal, Line spacing: single

Formatted: Indent: First line: 0 ch

Formatted: Font: Not Italic

Formatted: Font: Not Italic

Cross-Cultural Engagement (M = 5.00, SD = 0.00, CV = 0.00) achieved perfect consensus in both rounds, reflecting unanimous agreement on their critical role in fostering cultural confidence and balanced intercultural perspectives. Emotional Attachment to Local Culture maintained a high level of agreement (M = 4.00, SD = 0.38, CV = 0.09) in the second round, though its mean slightly decreased from 4.13 in the first round. The low CV value (0.09) confirms its reliability as a measure of learners' emotional connection to their cultural heritage.

Table 9. **Two-~~R~~round Delphi ~~S~~survey ~~R~~results for ~~S~~secondary ~~I~~indicators under the ~~P~~primary ~~I~~indicator** ***Local Affective Attitudes.***

Secondary Indicator	Mean (out of 5)		SD		CV		Action Taken
	1st	2nd	1st	2nd	1st	2nd	
Motivation to Express and Communicate Local Identity	4.80	4.93	0.41	0.26	0.09	0.05	Retained
Emotional Attachment to Local Culture	4.13	4.00	0.64	0.38	0.15	0.09	Retained
Cultural Pride	5.00	5.00	0.00	0.00	0.00	0.00	Retained
Critical Openness in Cross-Cultural Engagement	5.00	5.00	0.00	0.00	0.00	0.00	Retained

Local ~~E~~expression and ~~A~~application. Table 10 summarizes results of this dimension, demonstrating strong expert consensus on their relevance to assessing learners' ability to express and apply local culture through English.

Formatted: Font:

Formatted: Font: Not Italic

Formatted: Font:

Formatted: Font: (Default) Times New Roman, 12 pt, Not Bold, Font color: Gray-75%

Formatted: Normal, Line spacing: single

Formatted: Font: Italic

Two indicators—*Use of English for Local Storytelling* and *Promotion and Preservation of Local Image*—received unanimous support (M = 5.00, SD = 0.00), emphasizing storytelling and advocacy as critical skills for cultural dissemination. Though other indicators show slight fluctuations, the high consensus across indicators (CV < 0.10) validates their collective role in translating cultural knowledge into actionable language skills.

Table 10. Two-round Delphi survey results for secondary indicators under the primary indicator *Local Expression and Application*.

Secondary Indicator	Mean (out of 5)		SD		CV		Action Taken
	1st	2nd	1st	2nd	1st	2nd	
Cultural Comparison	4.00	4.07	0.59	0.26	0.16	0.06	Retained
Interdisciplinary Knowledge Application	4.67	4.93	0.49	0.26	0.10	0.05	Retained
Adaptation of Language to Reflect Local Norms	4.07	4.07	0.88	0.26	0.22	0.06	Retained
Use of English for Local Storytelling	5.00	5.00	0.00	0.00	0.00	0.00	Retained
Promotion and Preservation of Local Image	4.60	5.00	0.63	0.00	0.14	0.00	Retained

On the whole, all the indicators in the second round of Delphi survey received a "Retained" status, confirming that they were deemed relevant and essential by the experts. The retention of all indicators suggests a strong consensus among the experts on the comprehensive nature of the evaluation framework for assessing EFL learners' local cultural awareness. This unanimity demonstrates the importance of including both cognitive, affective, and behavioral dimensions in the assessment, ensuring that learners' understanding, emotional connection, and practical application of local

Formatted: Indent: First line: 2 ch

Formatted: Font: Italic

culture are all adequately measured.

Analytic ~~H~~hierarchy ~~P~~process (AHP) ~~W~~weighting ~~O~~outcomes

The third consultation phase employed the Analytic Hierarchy Process (AHP) to determine the relative weights of indicators within the evaluation system. Following AHP protocol, a hierarchical model (Fig.)was constructed based on the finalized primary and secondary indicators of EFL Learners' Local Cultural Awareness. Fifteen experts from the Delphi panel were invited to complete pairwise comparison matrices for the hierarchical structure(S3Table,S4 Table).

The expert evaluations were analyzed using AHP software to calculate eigenvectors, perform hierarchical single ranking, and conduct consistency checks. All four judgment matrices exhibited satisfactory consistency, with consistency ratios (CR) below the threshold of 0.1 and consistency indices (CI) approaching zero (**Table 11**). These results confirm the logical coherence of expert judgments and validate the weight assignments.

Field Code Changed

Table 11. AHP ~~C~~consistency ~~M~~metrics.

Hierarchy Level	*λmax*	CI	CR
Primary Dimensions	3.1008	0.0504	0.0970
Expression Sub-criteria	5.3500	0.0875	0.0781
Affective Sub-criteria	4.2364	0.0788	0.0886
Cognitive Sub-criteria	9.3900	0.0488	0.0334

The final weights (**Table 12**) revealed distinct priorities among the three primary dimensions *Expression and Application* (0.4527) received the highest weight,

Field Code Changed

reflecting its centrality in achieving EFL's ultimate goal of enabling learners to actively articulate and apply cultural knowledge. *Local Affective Attitudes* (0.3301) and *Local Cognition and Understanding* (0.2172) followed, emphasizing the complementary roles of emotional engagement and foundational cultural knowledge.

Table 12. Hierarchical ~~E~~evaluation ~~I~~index ~~S~~system with AHP ~~W~~weight ~~A~~allocation for EFL Learners' Local Cultural Awareness.

Primary indicators	Secondary indicators	Weight	Combined weight	Description of Secondary Indicator
I-1Local Cognition and Understanding (0.2172)	II-1Local History	0.1544	0.0335	Learners demonstrate their ability to use English to explore and discuss various aspects of local history, including key events, influential figures, and their cultural and societal significance, etc.
	II-2 Local Cultural Practices, Art, and Literature	0.1544	0.0335	Learners demonstrate their ability to use English to interpret and discuss local art forms and literary works, highlighting their significance as

			expressions of local culture and creativity, etc.
II-3 Local Development Achievements	0.1544	0.0335	Learners demonstrate their ability to use English to interpret and discuss local art forms and literary works, highlighting their significance as expressions of local culture and creativity, etc.
II-4 Daily Life Experiences	0.1544	0.0335	Learners demonstrate their ability to use English to describe and discuss aspects of local daily life, such as diet, family structure, education, and work environments, emphasizing their relevance to personal and community experiences, etc.
II-5 National Virtues and Qualities	0.1524	0.0331	Learners demonstrate their ability to use English to interpret and discuss their nation's core values, such as resilience, hospitality, and social virtues.
II-6 Local Ethical and Legal Systems	0.0921	0.0200	Learners demonstrate their ability to use English to discuss local ethical principles and legal frameworks, emphasizing their practical role in

			shaping societal order, resolving conflicts, and guiding everyday behavior within the community.
II-7 Local Scenic BeautyRegional Geography	0.0510	0.0111	Learners demonstrate their ability to use English to describe and connect geographical features, such as landscapes, rivers, mountains, and scenic spots, with local cultural practices, tourism, and community identity, etc.
II-8 Local and Global Issues in Everyday Contexts	0.0438	0.0095	Learners demonstrate their ability to use English to understand, discuss, and analyze broader societal issues that influence their daily lives, such as community governance, economic development, environmental challenges, and international relations, with a focus on their practical impact and global relevance
II-9 Local Language and Dialect Varieties	0.0430	0.0093	Learners demonstrate their ability to use English to discuss local language varieties, including dialects or regional expressions, and their connections to Standard English in

				terms of usage, meaning, and cultural significance.
	II-10 Cultural Pride	0.3578	0.1181	Learners' high identification and pride in local culture, and confidence demonstrated in cross-cultural communication.
I-2Local Affective Attitudes (0.3301)	II-11 Interest in Cross-Cultural Understanding	0.3578	0.1181	Learners' interest in understanding and discussing other cultures and the similarities and differences with their local culture through English, coupled with the ability to maintain a critical and open-minded approach during foreign language learning and cross-cultural communication, avoiding blind adoration or rejection of other cultures.
	II-12 Motivation to Express and Communicate Local Identity	0.1729	0.0571	Learners' motivation and willingness to express and disseminate local culture, identity, and values through English learning.
	II-13 Emotional Attachment to Local Culture	0.1115	0.0368	Learners' emotional attachment and identification with local culture.

	II-14 Use of English for Local Storytelling	0.3019	0.1367	Learners' ability to freely tell stories related to local culture in English
	II-15 Promotion and Preservation of Local Image	0.3019	0.1367	Learners' initiative to promote and preserve local image in cross-cultural communication, showcasing local cultural values through English.
I-3 Local Expression and Application (0.4527)	II-16 Interdisciplinary Knowledge Application	0.1843	0.0834	Learners' ability to integrate interdisciplinary knowledge of local culture (such as history, art, geography, etc.) in English expression.
	II-17 Adaptation of Language to Reflect Local Norms	0.1178	0.0533	Learners' ability to adjust language expression according to local cultural and social norms in cross-cultural communication.
	II-18 Cultural Comparison	0.0942	0.0426	Learners' ability to compare local culture with target culture in cross-cultural communication.

In terms of secondary indicator weights, *Use of English for Local Storytelling* (0.3019) and *Promotion and Preservation of Local Image* (0.3019) received the highest global weights (0.1367 each), emphasizing narrative-driven cultural advocacy as a core skill.*Cultural Pride* (0.3578) and *Critical Openness in Cross-Cultural Engagement* (0.3578) dominated the dimension of *Local Affective Attitudes* (global

weight = 0.1181, each), reflecting experts' emphasis on balancing cultural confidence. *Local History* (0.1544), *Local Cultural Practices, Art, and Literature* (0.1544), and *Daily Life Experiences* (0.1544) held equal weights (global weight=0.0335, each), indicating their foundational equivalence in cultural knowledge acquisition.

This three-phase process, combining Delphi consensus-building with AHP weighting, yielded a robust evaluation system comprising 3 primary and 18 secondary indicators. The hierarchical structure systematically integrates cognitive, affective, and behavioral dimensions of local cultural awareness, providing a quantifiable framework for assessing EFL learners' intercultural competence.

Discussion

Formatted: Font: 18 pt

This study constructed an evaluation index system for local cultural awareness in EFL learning through the Delphi method and Analytic Hierarchy Process (AHP), addressing the underrepresentation of learners' native cultural identities. The following discussion will interpret the findings from educational and theoretical perspectives and link the revision or retention of indicators to the practical significance in university EFL teaching..~~addressing the gap in existing frameworks that prioritize Anglophone cultural content over learners' native cultural identities. Below, we interpret these findings through the lens of cultural pedagogy and language acquisition theories, addressing why certain indicators were revised, retained, or removed, and their implications for EFL instruction.~~

The study confirmed that local cultural awareness in EFL learning can be

systematically evaluated through three dimensions (**Fig 1**): *Local Cognition and Understanding, Local Affective Attitudes*, and *Local Expression and Application*. The AHP-derived weights revealed that *Local Expression and Application* (W=0.4527) holds the highest priority, followed by *Local Affective Attitudes* (W=0.3301) and *Local Cognition and Understanding* (0.2172). Among secondary indicators (**Fig 2**), ~~The study confirmed that local cultural awareness in EFL learning can be systematically evaluated through three dimensions: *Local Cognition and Understanding, Local Affective Attitudes*, and *Local Expression and Application*. The AHP-derived weights revealed that *Local Expression and Application* (0.4527) holds the highest priority, followed by *Local Affective Attitudes* (0.3301) and *Local Cognition and Understanding* (0.2172). Among secondary indicators,~~ *Use of English for Local Storytelling, Promotion and Preservation of Local Image* emerged as the most critical skills, while *Cultural Pride* and *Critical Openness in Cross-Cultural Engagement* dominated the affective dimension. Meanwhile, *Local History*, *Local Cultural Practices, Art, and Literature*, *Local Development Achievements*, and *Daily Life Experiences* exhibit relatively high weights under the dimension of Cognition, revealing the multifaceted emphasis on local cognition and understanding in English learning.

Fig 1. Weights of primary indicators for local cultural awareness assessment

Fig 2. Secondary indicator weights in cultural awareness assessment

Local expression and application dimension

The significantly higher weight assigned to *Local Expression and Application* (weight = 0.4527, the highest among the three dimensions) not only aligns with Byram's [5] intercultural communicative competence (ICC) model, which prioritizes knowledge-to-practice transformation, but also finds empirical support in Fan's [12] study. In Fan’s research, action-oriented tasks yielded higher cultural retention, and this emphasis on practical alignment is further validated by the study’s finding that direct written corrective feedback (WCF) enhances students’ cultural expression in EFL writing—findings that complement Byram’s focus on operational competence.

This study extends prior research by developing an evaluation index system for EFL learners' local cultural awareness and integrating it into project-based output tasks. This approach operationalizes Byram's Intercultural Communicative Competence (ICC) principles through context-specific language applications—a critical yet underexplored dimension in existing literature. As demonstrated in S5 Table, in teaching practice, this study suggests replacing 45% of the traditional cultural teaching modules in EFL programs with Production-oriented tasks . For instance, students could be tasked with documenting local heritage sites in English—such as ancient bridges bearing donation inscriptions—and composing analytical narratives that systematically unpack the cultural connotations embedded in these physical spaces. Through their writing, learners should illuminate how these

Formatted: Font: 16 pt

Formatted: Heading 2, Indent: First line: 2 ch, Line spacing: single

Formatted: Font: (Default) Times New Roman, 12 pt

Formatted: Font: (Default) Times New Roman, 12 pt, Italic

Formatted: Font: (Default) Times New Roman, 12 pt

inscriptions exemplify traditional values of fraternal cooperation and contractual ethics, bridging historical artifacts with contemporary understanding.Through the knowledge-action transformation mechanism, this design directly responds to the highest AHP-assigned weight of this dimension, translating the ICC model from a theoretical framework into teaching practice.

~~The prioritization of Local Expression and Application resonates with Byram's (1997) model of intercultural communicative competence (ICC), which emphasizes transforming cultural knowledge into actionable skills. The high emphasis on storytelling and cultural advocacy reflects the growing importance of narrative-driven pedagogy in English [1,2], where learners actively articulate cultural identity rather than passively absorb content.~~

Formatted: Strikethrough

Emotional dimension

Formatted: Font: Times New Roman

Formatted: Font: Times New Roman

Formatted: Heading 2, Line spacing: single

In the emotional dimension, the study shows balanced emphasis on *Cultural Pride* (global weight=0.1181) and *Openness in Cross-Cultural Engagement* (global weight=0.1181). These results are consistent with Kramsch's[26] view that language education should avoid cultural essentialism and cultivate students' hybrid identity capabilities . This equal emphasis, addressing the limitations of earlier models that either overemphasized cultural loyalty or prioritized global perspectives at the expense of local roots [52] , is operationalized through specific teaching tasks in university EFL courses.

In university EFL assessment, cultural pride is evaluated through tasks such as

the "Pride Portfolio" (II-10 in S5 Table). For example, anthropology students' cultural awareness is measured by their ability to compile cultural artifacts with precise English annotations (e.g., distinguishing "butterfly patterns symbolizing ancestral blessings" from generic "insect decorations"), demonstrating how linguistic accuracy reflects cultural symbol interpretation in evaluation criteria.

Similarly, cross-cultural openness is assessed via the "Cultural Exchange Archive" (II-11 in S5 Table), where evaluation focuses on students' capacity to create bilingual packages—documenting local traditions like Dragon Boat Races in English while critically analyzing foreign customs (e.g., Christmas) from exchanged materials.

In university exchange programs, learners' performance is assessed through comparative tasks: basic learners might contrast festival food practices via infographics, while advanced learners conduct semiotic analyses of festival symbols in academic posters. This bidirectional comparison aligns with Delvin's [53] "critical cultural awareness" for balanced cultural representation. The equal AHP weights for Cultural Pride and Openness (0.1181 each) validate this assessment approach.The equal AHP weights for Cultural Pride and Openness (0.1181 each) validate this approach.It also embodies CLIL principles [18],[19], integrating language learning with cross-cultural inquiry.

Cognitive dimension

In the cognitive dimension, the study observed significant divergence in expert

opinions during the first Delphi round, particularly regarding abstract cultural indicators such as the *Local Political System* and *Local Economic System* (CV = 0.45 and CV = 0.36, respectively). These high coefficients of variation suggest that experts questioned the relevance of such institutional topics in the context of EFL learning. This is consistent with Coyle's view that language learning should be combined with meaningful content and communicative practices, rather than just focusing on abstract cultural knowledge [54]. Therefore, by deleting or merging these abstract topics, the study ensures that cultural content is both comprehensible and relevant to university students.

Under the main indicator *Local Cognition and Understanding*, the three secondary indicators - *Daily Life Experiences* , *Local Cultural Practices, Art, and Literature*, and *National Virtues and Qualities* - exhibited perfect evaluative consensus (CV=0.00). This consensus confirms the critical role of culturally-embedded assessment tasks in measuring EFL learners' ability to mediate local knowledge through English, as exemplified by "a day in the life" documentation (II-4) and local figure narration (II-5) tasks in S5 Table. Aligned with Alakrash's [2] cognitive authenticity principle, these evaluation criteria demonstrate how contextually-grounded content enhances linguistic encoding and identity positioning in assessment outcomes. The transformation task for local cultural artifacts (II-2 in S5 Table), for instance, evaluates students' symbolic mediation competence through parameters including: (1) cultural authenticity of adapted content, (2) linguistic accuracy in performance scripts, and (3) effective conveyance of embodied cultural

meanings. Such multidimensional assessment operationalizes Kramsch's [49] "symbolic competence" framework, where successful performance necessitates transcending lexical equivalence to achieve semiotic resonance between form and cultural substance.

The substantial weighting of Local Development Achievements (global weight=0.0335) underscores the evaluation framework's recognition of contemporary cultural production as equally significant as traditional manifestations. This sub-indicator measures learners' techno-cultural literacy through their ability to mediate between abstract cultural values and concrete development metrics. In university assessment contexts, business students' cultural awareness is measured through tasks like creating "local development data visualization" reports in English (e.g., analyzing cultural industry GDP contributions) . Successful performance requires operationalizing Porto's [3] knowledge model by contextualizing technological and cultural narratives within specific development metrics. The assessment thus validates learners' capacity to concretize socio-cultural abstractions through quantitative discourse.This evaluative approach bridges the epistemic divide between cultural studies and development economics in EFL contexts.

Notably, the indicator *Local and Global Issues in Everyday Contexts* (M = 3.93, global weight=0.0200), though scoring relatively low, was retained by experts for its role in cultivating critical global citizenship. In university EFL teaching, this is operationalized through graded tasks: advanced learners' critical analysis capabilities are measured through English debates on "local issues with global implications" (e.g.,

"ancient town tourism vs. cultural authenticity preservation"), while intermediate learners demonstrate their understanding through structured pro-con reports on topics like "dialect protection and tourism development." This differentiated assessment approach validates Gu's [22] theoretical position that internationalized curricula can sustain cultural heterogeneity through critical engagement. The evaluation criteria operationalize Gu's conceptual integration of practical language skills and cultural awareness—specifically measuring learners' capacity to articulate local identities while navigating global discourse frameworks.

Integration with existing standard frameworks in university context

The proposed local cultural awareness assessment model provides a comprehensive approach to evaluating EFL learners' engagement with local culture. Furthermore, the practical application of this model can be aligned with widely recognized standard frameworks, such as the Common European Framework of Reference for Languages (CEFR) and the American Council on the Teaching of Foreign Languages (ACTFL) proficiency guidelines. Although this study highlights the blind spots of current frameworks (such as CEFR and ACTFL) in assessing local cultural expression, the new model is not intended to replace these standardized systems but to achieve synergistic enhancement through a hierarchical complementary strategy.

The CEFR offers a comprehensive set of language proficiency descriptors

spanning diverse proficiency levels [56]. We propose integrating the local cultural awareness assessment model with the CEFR starting from the intermediate level, employing a descriptor transplantation approach. Specifically, this integration can be achieved by embedding local cultural indicators into the CEFR's mediational and plurilingual descriptors, as underscored in the document regarding "action-oriented scenarios" and "plurilingual repertoires" [56]. As outlined in S6Table, this strategic embedding aligns secondary indicators with CEFR levels B1 to C2, illustrating how descriptors like "relaying specific information" (B2) and "facilitating pluricultural space" (C1) can be adapted to assess local cultural competence.

B1–B2 Intermediate Level Integration: At these levels, foundational local cultural indicators (e.g., II-1 Local History, II-4 Daily Life Experiences) can be integrated into CEFR’s "Mediating Concepts" and "Reception of Information" descriptors. For instance, II-1’s requirement for learners to "discuss local history events in English" aligns with CEFR B2’s descriptor for "relaying specific information" (Section 3.1.4.2)[56], where learners are expected to "summarize historical contexts with cultural significance". Similarly, II-6 (Local Ethical and Legal Systems) can be grafted onto CEFR’s B2 "Explaining Data" scale, as the document highlights the importance of mediating social norms through language .

C1–C2 Advanced Level Integration: At higher proficiency tiers, sophisticated cultural indicators (e.g., II-15 Local Image Promotion, II-18 Cultural Comparison) map to CEFR’s "Plurilingual Mediation" and "Facilitating Pluricultural Space" domains. For example, II-18’s requirement to "compare local and target cultures"

Formatted: Not Highlight

directly aligns with CEFR C1's descriptor for "mediating cross-cultural dialogue" (Section 7.2.2), where learners are expected to "negotiate cultural differences in complex scenarios". Additionally, II-15's focus on "promoting local identity in English" resonates with the emphasis of CEFR on "activating plurilingual repertoires for social agency" , which can be embedded in CEFR C2's "Strategic Mediation" scale.

Cross-Level Task Design for Cultural Embedding: The document's advocacy for "scenario-based assessment" (Section 7.2.1) provides a framework for designing tasks that bridge proficiency levels[56]. For instance, In B1–B2 tasks, Learners could complete a "local food culture interview" (II-4) using CEFR B2's "Oral Interaction" descriptors, which emphasize "describing daily practices with cultural relevance". And in terms of C1–C2 tasks, A "local heritage preservation campaign" (II-15) could be evaluated against CEFR C1's "Mediation of Public Discourse" criteria, requiring learners to "craft persuasive messages that integrate local values" .

Similarly, ACTFL emphasizes "intercultural communicative competence," focusing on the appropriate use of language in real social and cultural contexts[33]. We propose using the evaluation indicators established in this study to form a dual-track assessment mechanism (**Error! Reference source not found.**)For example, a cultural awareness dimension can be added to the ACTFL Oral Proficiency Interview (OPI), allowing independent scoring of language fluency and cultural mediation while sharing weights (suggested ratio: 70% language + 30% culture). This integrated solution, which balances the emphasis on language and cultural

competence, retains the reliability and validity of standardized tests and addresses the missing identity expression through the cultural competence matrix (**Fig 3**). It responds to Deardorff's[35] call for a "mixed assessment method" (quantitative + qualitative) to measure cultural depth. Meanwhile, the dual-track model proposed in this study requires minimal modification to the existing ACTFL protocols but significantly enhances ecological validity in multilingual contexts.

Fig 3. Schematic of the dual - track assessment process for the ACTFL OPI task

Limitations and future directions

Formatted: Font: Times New Roman

Formatted: Heading 2

This study represents an initial exploration of evaluating local cultural awareness among EFL learners through a Delphi-based framework, specifically contextualized within the Chinese educational landscape. The current expert panel, comprising 15 professionals from China, provides a foundational perspective on integrating local content in EFL education. While this sample reflects the nuanced dynamics of Chinese cultural pedagogy, future research may benefit from expanding the panel to include experts from diverse EFL contexts—such as Latin America, Africa, or Southeast Asia—to further validate the framework's adaptability across cultural and educational systems.

Building on this foundation, subsequent studies could pursue two complementary paths. First, cross-cultural validation in non-Chinese settings would shed light on

whether the evaluation index system requires contextual adjustments or demonstrates transferable utility. Such efforts would enrich the framework by incorporating varied perspectives on balancing local and global cultural elements in EFL curricula. Second, longitudinal investigations tracking how local cultural awareness influences students' language development and intercultural competence over time would deepen our understanding of its educational impact. Mixed-method designs combining quantitative assessments with qualitative case studies could offer nuanced insights into how learners navigate cultural hybridity in different linguistic environments.

Notably, this research establishes a critical starting point for discussing local cultural integration in EFL education, particularly in contexts where similar cultural priorities may exist. By acknowledging the potential for broader application and inviting interdisciplinary dialogue, future studies can refine the framework to accommodate the diverse needs of EFL learners worldwide, while retaining sensitivity to regional cultural identities. This approach ensures that educational interventions can both honor local voices and foster global communicative agility.

In the affective dimension, the study revealed a balanced emphasis on *Cultural Pride* (0.3578) and *Openness in Cross-Cultural Engagement* (0.3578). These results are consistent with Kramsch's [25] view that language education should avoid cultural essentialism and foster students' ability to negotiate mixed identities. The same weighting of *Cultural Pride* and *Openness in Cross-Cultural Engagement* effectively addresses criticism of early patterns. Those patterns either overemphasize

cultural loyalty or prioritize the global perspective at the expense of local roots [47] . By resisting binary oppositions between local loyalty and global assimilation, our findings realize what Dervin [48] terms "Critical Cultural Awareness", where cultural confidence coexists with reflexive skepticism toward both native and target cultures.

Under the primary indicator *Local Cognition and Understanding*, three secondary indicators, *Daily Life Experiences*, *Local Cultural Practices, Art, and Literature*, and *National Virtues and Qualities*, achieved near-perfect consensus (CV = 0.00). These findings highlight the importance of concentrating on content that is culturally relevant, enabling learners to readily place it in context and engage in discussions using English. This finding corresponds to Alakrash's [1] finding that cultural content directly relatable to learners' experiences enhances both language retention and identity formation. By prioritizing tangible cultural practices, this study echoes the broader trend in language education that emphasizes context-based learning, where learners engage more deeply with the material, making the learning process more relevant and meaningful. Interestingly, the equal weight assigned to *Local Development Achievements* suggests that learners should focus not only on traditional aspects of culture but also on contemporary achievements. *Understanding Local Development Achievements* is integral to fostering modern cultural identity, especially in rapidly developing societies. Achievements in science and technology, the economy, and other fields are the concrete embodiments of cultural values such as innovation and collaboration. Interpreting local achievements in English requires translating abstract cultural

values into concrete cases, which aligns with Kramsch's [49] theory of "language as symbolic practice". This theory posits that language is not merely a neutral conduit for communication, but a dynamic system of signs that actively constructs meaning through culturally embedded symbolic resources.

The study also observed significant divergence in expert opinions during the first Delphi round, particularly regarding abstract cultural indicators such as the *Local Political System* and *Local Economic System* (CV = 0.45 and CV = 0.36, respectively). These high coefficients of variation suggest that experts questioned the relevance of such institutional topics in the context of EFL learning. This is in line with Coyle's [50] emphasis on integrating language learning with meaningful content and communicative practice, rather than focusing solely on abstract cultural knowledge. Coyle argues that in CLIL (Content and Language Integrated Learning) settings, the focus should be on "learning content through language and learning language through content" , highlighting the potential disengagement caused by overly complex cultural content that diverts learners' attention from communicative competence. Therefore, by removing or merging these abstract topics, the study ensures that the cultural content remains accessible and relevant to students. This approach is consistent with Porto's call [2] to prioritize the application of cultural knowledge over its memorization, thereby enhancing students' ability to use cultural knowledge in real-life communicative contexts.

The study retained the indicator *Local and Global Issues in Everyday Contexts* (M = 3.93) regarding its crucial role in bridging local identity with global

citizenship. Our experts want to keep the indicator. Even though it has a relatively lower average score. Some experts emphasized that, although mastery of this indicator demands advanced language skills, its value lies in cultivating critical global citizenship. Learners are encouraged to neither uncritically adopt global norms nor strictly cling to local traditions. This conclusion is consistent with Gu's [28] findings that, while internationalized programs risk curricular homogeneity, they can paradoxically foster cultural heterogeneity by exposing students to diverse values and prompting critical reflections on globalization. And Gu's research warns against seeing English proficiency as the same as internationalization. The retained indicator implements Gu's call for curricula, which combines practical language skills with cultural awareness, enabling learners to maintain local identities while developing flexibility for global communication.

Conclusion

Formatted: Font: 18 pt

This study constructs a comprehensive evaluation framework for local cultural awareness in EFL education through the Delphi-AHP methodology, translating Kramsch's symbolic competence theory into quantifiable assessment metrics. The empirically validated framework comprises three core dimensions—Local Expression and Application (45.27% weight), Local Affective Attitudes (33.01%), and Local Cognition and Understanding (21.72%)—with 18 secondary indicators, such as "Cultural Comparison" and "Local Image Promotion". Its primary innovation lies in bridging sociocultural theories (e.g., Byram’s ICC model) with classroom practice by

operationalizing abstract constructs into AHP-weighted evaluation criteria, addressing the overemphasis on Anglophone content in standardized curricula.

The framework offers versatile applications in EFL contexts: it supports curriculum developers in benchmarking cultural balance, enables AI-driven adaptive learning through metrics like "Cultural Pride", and assists policymakers in assessing glocal citizenship cultivation. By emphasizing local storytelling and cross-cultural comparison, the framework empowers learners as cultural agents while fostering objective cultural understanding. This research establishes a foundational model for integrating local cultural content in EFL education, laying groundwork for future cross-cultural validation and longitudinal impact studies.

This study establishes a foundational framework for evaluating local cultural awareness in EFL education based on the Delphi method and Analytic Hierarchy Process (AHP). The evaluation index system covers three first-level indicators: *Local Expression & Application* (45.72%), *Local Affective Attitudes* (33.01%), and *Local Cognition & Understanding* (21.27%). By converting Kramsch's[49] symbolic competence theory into quantifiable metrics — such as *Cultural Comparison* (0.0426) and *Promotion and Preservation of Local Image* (0.1367) — this framework bridges a critical gap between sociocultural language theories and classroom assessment practices. These dimensions offer practical strategies aimed at reducing the excessive focus on Anglophone cultural content within standardized curricula. The evaluation system is further subdivided into 18 secondary indicators, such as *Local Development Achievement*, *Cultural Pride*,

Openness in Cross-Cultural Engagement, and Promotion and Preservation of Local Image.

The proposed evaluation framework offers flexible applications across diverse EFL educational contexts. It can function as both a diagnostic and developmental tool. Primarily, curriculum developers can use it to assess cultural balance in materials, such as comparing coverage of local festivals versus Western holidays in textbooks. In blended learning environments, this framework dynamically adjusts instructional content based on learners' cultural engagement levels. For instance, AI tutors can personalize feedback in virtual heritage activities by prioritizing *Cultural Pride* metrics when students demonstrate strong local identity connections. Additionally, policy makers might adopt the hierarchy to benchmark institutional performance. They can assess how effectively schools cultivate glocal citizenship through metrics like *Promotion and Preservation of Local Image.* Crucially, the system empowers learners as self-reflective cultural agents. For example, students can use integrated digital portfolios to document how they blend cultural influences, such as creating English vlogs comparing regional cooking traditions. These real-world uses highlight two key strengths of the framework: it serves as an evidence-based evaluation tool while actively supporting teaching methods that value and balance cultural diversity. Lastly, the evaluation mechanism addresses biases in learners' foreign language narratives. By emphasizing local narratives, educators can identify and correct biases, fostering learners' objective and comprehensive understanding of local culture. This approach avoids excessive

exaggeration or distortion of facts, prevents perceptual blindness, and promotes students' holistic development in cognition, emotion, and behavior.

While this study provides valuable insights into the evaluation of local cultural awareness in EFL learning, there are some limitations that should be considered for future research. First, the number of experts is only 15, and all are from China. Future research should consider a broader range of participants and contexts to validate the robustness of the evaluation index system. Meanwhile, the study focuses primarily on the evaluation of local cultural awareness, but further research is needed to explore the long-term impact of this consciousness on students' language proficiency and global citizenship.

Despite these limitations, this study offers a foundation for future research and practical applications in assessing how well the local content was integrated in EFL education. By addressing the limitations identified, future studies can further refine the evaluation index system and explore its application in diverse educational contexts. Furthermore, more research should be conducted to collect and analyze quantitative and qualitative data to identify the specific needs of learners to navigate through cultural hybridization. This could enable educators to develop teaching interventions that amplify local voices while making students agile participants in global discourse.

Supporting information

S 1Fig.Research Workflow Diagram.

(TIFF)

S 2Fig.Hierarchical Model of EFL Learners' Local Cultural Awareness Evaluation System.

(TIFF)

S1Table.First Delphi Round Consultation on Secondary Indicators for EFL Learners' Local Awareness Evaluation System.

(Table)

S2Table.Second Delphi Round Consultation on Secondary Indicators for EFL Learners' Local Awareness Evaluation System.

(Table)

S3Table.Pairwise Comparison Matrix for Primary Indicators.

(Table)

S4Table.Pairwise Comparison Matrix for Secondary Indicators

(Table)

Reference

Formatted: Font: 18 pt

1. Gao P, Zhang X, Fu Y. Cultural confidence construction of contemporary college students: A case study of English majors. *Academic Journal of Humanities & Social Sciences*. 2023;6(18):24-28.https://doi.org/10.25236/AJHSS.2023.061805
2. Alakrash H, Edam B, Bustan E, Armnazi M, Enayat A, Bustan T. Developing English language skills and confidence using local culture-based materials in EFL curriculum. *Linguistica Antverpiensia. Google*
3. Porto M. Does education for intercultural citizenship lead to language learning? *Language, Culture and Curriculum*. 2018;32(1):16-33. https://doi.org/10.1080/07908318.2017.1421642
4. Scally J, Parrish A, Montgomery A. Intercultural competence and languages: inextricably linked or linked inexplicably? *Compare: a journal of comparative and international education*. 2022;52(3):492-510. https://doi.org/10.1080/03057925.2020.1851000
5. Byram M. *Teaching and Assessing Intercultural Communicative Competence: Revisited*. Bristol, Blue Ridge: Multilingual Matters; 2021. https://doi.org/10.21832/9781800410251
6. Zhang H, Ge S, Mohd Saad MRB. Formative assessment in K-12 English as a foreign language education: A systematic review. *Heliyon*. 2024;10(10):e31367. https://doi.org/10.1016/j.heliyon.2024.e31367
7. Risager K. *Language and Culture: Global Flows and Local Complexity*. Clevedon, UK: Multilingual Matters; 2006. Google
8. Saavedra AR, Opfer VD. *Teaching and Learning 21st Century Skills: Lessons from the Learning Sciences*. New York: Asia Society; 2012. Google
9. Vygotsky LS. *Mind in Society: The Development of Higher Psychological Processes*. Cambridge: Harvard University Press; 1978. Google
10. Tomlinson B, editor. *Developing Materials for Language Teaching*. London: Bloomsbury Publishing; 2023. Google
11. Kramsch C. *Context and Culture in Language Teaching*. Oxford: Oxford University Press; 1993. Google
12. Fan N. Exploring the effects of automated written corrective feedback on EFL students' writing quality: a mixed-methods study. *SAGE Open*. 2023;1:1–17. doi=10.1177/21582440231181296
13. Canagarajah S. *Translingual Practice: Global Englishes and Cosmopolitan Relations*. New York: Routledge; 2012.https://doi.org/10.4324/9780203073889
14. Lavrenteva E, Orland-Barak L. The treatment of culture in the foreign language curriculum: an analysis of national curriculum documents. *Journal of Curriculum Studies*. 2015;47(5):653-684. https://doi.org/10.1080/00220272.2015.1056233
15. Guo Q, Jamil H, Ismail L, Luo S, Sun Z. Effects of problem-based learning on EFL learning: A systematic review. *PLoS One*. 2024;19(12):e0307819. https://doi.org/10.1371/journal.pone.0307819
16. Yang M. Making interdisciplinary subjects relevant to students: an interdisciplinary approach. *Teaching in Higher Education*. 2009;14(6):597-606. https://doi.org/10.1080/13562510903315019

17. Chubko N, Morris JE, McKinnon DH, et al. Digital storytelling as a disciplinary literacy enhancement tool for EFL students. *Education Tech Research Dev*. 2020;68:3587-3604. https://doi.org/10.1007/s11423-020-09833-x
18. Dalton-Puffer C. Outcomes and processes in Content and Language Integrated Learning (CLIL): current research from Europe. *na*. 2008.Google Scholar
19. Lasagabaster D. English achievement and student motivation in CLIL and EFL settings. *Innovation in Language Learning and Teaching*. 2011;5(1):3–18. https://doi.org/10.1080/17501229.2010.519030
20. Margianti SF. The inclusion of local cultural contents in English classes: Teachers' perceptions. *Langue (Journal of Language and Education)*. 2022;21(1):97-105. https://doi.org/10.22437/langue.v1i1.22609
21. Wu JG, Miller L. Raising native cultural awareness through WeChat: a case study with Chinese EFL students. *Computer Assisted Language Learning*. 2019;34(4):552-582. https://doi.org/10.1080/09588221.2019.1629962
22. Gu MM, Lee JC-K. "They lost internationalization in pursuit of internationalization": students' language practices and identity construction in a cross-disciplinary EMI program in a university in China. *High Educ*. 2019;78:389-405.https://doi.org/10.1007/s10734-018-0342-2.
23. Kanoksilapatham B, Chann P. EFL learners' and teachers' positive attitudes towards local community-based instruction. *Indonesian Journal of Applied Linguistics*. 2018;7(3):504-514.Google Scholar
24. Aminullah F, Sada C, Sudarsono S. Local culture-based reading materials for EFL context. In: Proceedings of the International Conference on Teaching and Education (ICoTE). Vol 2, No 2. 2019.Google Scholar
25. Gashi L. Intercultural awareness through English language teaching: the case of Kosovo. *Interchange*. 2021;52:357-375. doi: 10.1007/s10780-021-09441-5.https://doi.org/10.1007/s10780-021-09441-5
26. Kramsch C. *Language and Culture*. Oxford: OUP Oxford; 1998. Google
27. Zhai N, Ma X. Automated writing evaluation (AWE) feedback: A systematic investigation of college students' acceptance. *Computer Assisted Language Learning*. 2022;35(9):2817-2842. https://doi.org/10.1080/09588221.2021.1897019
28. Gao M, Qian J, Rasool U. Effects of Task-Induced Involvement and Time on Task on Incidental L2 Vocabulary Acquisition. *SAGE Open*. 2024;14(2). https://doi.org/10.1177/21582440241249340
29. Winstone N, Boud D, Dawson P, Heron M. From feedback-as-information to feedback-as-process: a linguistic analysis of the feedback literature. *Assessment & Evaluation in Higher Education*. 2022;47(2):213-230. https://doi.org/10.1080/02602938.2021.1902467
30. Gay G. Culturally responsive teaching: Theory, research, and practice. 2nd ed. New York: Teachers College Press; 2018. Google
31. Juan-Garau M, Jacob K. Developing English learners' transcultural skills through transcultural content-and task-based lessons. *System*. 2015;54:55-6. https://doi.org/10.1016/j.system.2015.04.017

32. Figueras N. The impact of the CEFR. *ELT Journal*. 2012;66(4):477-485. https://doi.org/10.1093/elt/ccs037
33. ACTFL. ACTFL proficiency guidelines 2012. https://www.actfl.org
34. Page D, Benander R. Cultural products and practices leading to cultural perspectives: Practical applications. *The Journal for Research and Practice in College Teaching*. 2016;1(1).Google Scholar
35. Deardorff DK. Identification and assessment of intercultural competence as a student outcome of internationalization. *Journal of Studies in International Education*. 2006;10(3):241-266.https://doi.org/10.1177/1028315306287002
36. Ait-bouzid H. Exploring global citizenship as a cross-curricular theme in Moroccan ELT textbooks. *Eurasian Journal of Applied Linguistics*. 2020;6(2):229-242. https://doi.org/10.32601/ejal.775801
37. Ye W, Ren W, Zheng S. The Construct of the Story Continuation Writing Task: Insights From the China's Standards of English Language Ability. *Chinese Journal of Applied Linguistics*. 2021;44(3):382-398. https://doi.org/10.1515/CJAL-2021-0024
38. Lange K. Perspectives on intercultural competence: a textbook analysis and an empirical study of teachers' and students' attitudes. *Intercultural Pragmatics*. 2011;8(2):183-208.Google Scholar
39. Wernicke M. Plurilingualism as agentive resource in L2 teacher identity. *System*. 2018;79:91-102. https://doi.org/10.1016/j.system.2018.07.005
40. McNamara T. Managing learning: authority and language assessment. *Language Teaching*. 2011;44(4):500-515. https://doi.org/10.1017/S0261444811000073
41. Hsu CC, Sandford BA. The Delphi Technique: Making Sense of Consensus. *Practical Assessment, Research, and Evaluation*. 2007;12(1):10. https://doi.org/10.7275/pdz9-th90
42. Fang F, Baker W. "A more inclusive mind towards the world": English language teaching and study abroad in China from intercultural citizenship and English as a lingua franca perspectives. *Language Teaching Research*. 2018;22(5):608-624. https://doi.org/10.1177/1362168817718574
43. Zhang D, Wu JG. Enhancing EFL learners' native cultural awareness via project-based learning. *TESL-EJ*. 2023;27(1):n1.Google Scholar
44. Dewaele JM, MacIntyre PD. The two faces of Janus? Anxiety and enjoyment in the foreign language classroom. *Studies in Second Language Learning and Teaching*. 2014;4(2):237-274. https://doi.org/10.14746/ssllt.2014.4.2.5
45. Oktarina Y, Inderawati R, Petrus I. Developing local culture-based EFL reading materials for the 21st-century learning. *Studies in English Language and Education*. 2022;9(3):1128-1147. https://doi.org/10.24815/siele.v9i3.24660
46. Polit DF, Beck CT. The content validity index: are you sure you know what's being reported? Critique and recommendations. *Research in Nursing & Health*. 2006;29(5):489-497. https://doi.org/10.1002/nur.20147
47. Saaty TL. Highlights and critical points in the theory and application of the analytic hierarchy process. *European Journal of Operational Research*. 1994;74(3):426-447.https://doi.org/10.1016/0377-2217(94)90222-4

48. Hasson F, Keeney S, McKenna H. Research guidelines for the Delphi survey technique. *Journal of Advanced Nursing*. 2000;32(4):1008-1015. https://doi.org/10.1046/j.1365-2648.2000.t01-1-01567.x
49. Schmidt RC. Managing Delphi surveys using nonparametric statistical techniques. *Decision Sciences*. 1997;28(3):763-774. https://doi.org/10.1111/j.1540-5915.1997.tb01330.x
50. Price J, Rushton A, Tyros V, Heneghan NR. Expert consensus on the important chronic non-specific neck pain motor control and segmental exercise and dosage variables: An international e-Delphi study. *PLoS One*. 2021;16(7):e0253523. https://doi.org/10.1371/journal.pone.0253523
51. Barkhuizen G. Narrative knowledging in TESOL. *TESOL Quarterly*. 2011;45(3):391-414. https://doi.org/10.5054/tq.2011.261888
52. Keles U, Yazan B. Representation of cultures and communities in a global ELT textbook: A diachronic content analysis. *Language Teaching Research*. 2023;27(5):1325-1346. https://doi.org/10.1177/1362168820976922
53. Dervin F. Interculturality in Education: A Theoretical and Methodological Toolbox. London: Palgrave Macmillan; 2016. pp. 14, 16.Google Scholar
54. Coyle D. Content and language integrated learning: towards a connected research agenda for CLIL pedagogies. *International Journal of Bilingual Education and Bilingualism*. 2007;10(5):543-562. https://doi.org/10.2167/beb459.0
55. Kramsch C. The symbolic dimensions of the intercultural. Language Teaching. 2011;44(3):354–67. https://doi.org/10.1017/S0261444810000431
56. Council of Europe. Common European Framework of Reference for Languages. Strasbourg: Council of Europe; c2023 [cited 2025 Jun 23]. Available from: https://www.coe.int/en/web/common-european-framework-reference-languages.

1.

2. Alakrash H, Edam B, Bustan E, Armnazi M, Enayat A, Bustan T. Developing English language skills and confidence using local culture-based materials in EFL curriculum. *Linguistica Antverpiensia*. 2021;20(1):548-564.
3. Porto M. *Does education for intercultural citizenship lead to language learning?* Language, Culture and Curriculum. 2018;32(1):16-33. https://doi.org/10.1080/07908318.2017.1421642
4. Scally J, Parrish A, Montgomery A. Intercultural competence and languages: inextricably linked or linked inexplicably? *Compare: a journal of comparative and international education*. 2022;52(3):492-510.https://doi.org/10.1016/j.system.2018.07.005
5. Byram M. *Intercultural Communicative Competence in Foreign Language Education*. Clevedon: Multilingual Matters; 1997.

6. Zhang, H., Ge, S., & Mohd Saad, M. R. B. (2024). Formative assessment in K-12 English as a foreign language education: A systematic review. *Heliyon*, 10(10), e31367. https://doi.org/10.1016/j.heliyon.2024.e31367

Formatted: No bullets or numbering

Formatted: No bullets or numbering

7. Risager K. *Language and culture: Global flows and local complexity.* Clevedon, UK: Multilingual Matters; 2006.

8. Saavedra AR, Opfer VD. *Teaching and learning 21st century skills: Lessons from the learning sciences.* New York: Asia Society; 2012.

9. Vygotsky LS. Mind in society: the development of higher psychological processes. *Harvard University Press*. 1978.

10. Helot C, Young A. Language education and identity: bridging the local and the global. *International Journal of Bilingual Education and Bilingualism*. 2005;8(4):256-268.. https://doi.org/10.1080/13670050508668610

11. Tomlinson B, editor. Developing materials for language teaching. *Bloomsbury Publishing*. 2023.

12. Kramsch C. *Context and culture in language teaching*. Oxford: Oxford University Press; 1993.

13. Fan N. Exploring the effects of automated written corrective feedback on EFL students' writing quality: a mixed-methods study. SAGE Open. 2023 Apr-Jun;1-17.https://sage.cnpereading.com/paragraph/article/?doi=10.1177/21582440231181296

14. Canagarajah S. *Translingual practice: Global Englishes and cosmopolitan relations.* New York: Routledge; 2012.

15. Lavrenteva E, Orland-Barak L. *The treatment of culture in the foreign language curriculum: an analysis of national curriculum documents.* Journal of Curriculum Studies. 2015;47(5):653-684. https://doi.org/10.1080/00220272.2015.1056233

16. Guo Q, Jamil H, Ismail L, Luo S, Sun Z. *Effects of problem-based learning on EFL learning: A systematic review.* PLoS One. 2024;19(12):e0307819. https://doi.org/10.1371/journal.pone.0307819

17. Yang M. *Making interdisciplinary subjects relevant to students: an interdisciplinary approach.* Teaching in Higher Education. 2009;14(6):597-606. https://doi.org/10.1080/13562510903315019

18. Chubko N, Morris JE, McKinnon DH, et al. *Digital storytelling as a disciplinary literacy enhancement tool for EFL students.* Education Tech Research Dev. 2020;68:3587-3604. https://doi.org/10.1007/s11423-020-09833-x

19. Dalton-Puffer C. *Outcomes and processes in Content and Language Integrated Learning (CLIL): current research from Europe*. na. 2008.

20. Lasagabaster D. *English achievement and student motivation in CLIL and EFL settings*. Innovation in Language Learning and Teaching. 2011;5(1):3-18. https://doi.org/10.1080/17501229.2010.519030

21. Margianti SF. The Inclusion of Local Cultural Contents in English Classes: Teachers' Perceptions. *Langue (Journal of Language and Education)*. 2022;1(1):97–105.https://doi.org/10.22437/langue.v1i1.22609

22. Wu JG, Miller L. Raising native cultural awareness through WeChat: a case study with Chinese EFL students. *Computer Assisted Language Learning*. 2019;34(4):552–582.https://doi.org/10.1080/09588221.2019.1629962

23. Kanoksilapatham B, Channuan P. EFL learners' and teachers' positive attitudes towards local community-based instruction. *Indonesian Journal of Applied Linguistics*. 2018;7(3):504-514.

24. Gay G. *Culturally Responsive Teaching: Theory, Research, and Practice*. 2nd ed. New York: Teachers College Press; 2018.

25. Aminullah F, Sada C, Sudarsono S. Local culture-based reading materials for EFL context. In: Proceedings of the International Conference on Teaching and Education (ICoTE). Vol 2, No 2. 2019.

26. Gashi L. Intercultural awareness through English language teaching: the case of Kosovo. *Interchange*. 2021;52:357–375.https://doi.org/10.1007/s10780-021-09441-5

27. Kramsch CJ. *Language and Culture*. Oxford: Oxford University Press; 1998.

28. Juan-Garau M, Jacob K. Developing English learners' transcultural skills through content-and task-based lessons. *System* 2015;54:55-68. https://doi.org/10.1016/j.system.2015.04.017

29. Wu JG, Miller L. Raising native cultural awareness through WeChat: a case study with Chinese EFL students. *Computer Assisted Language Learning*. 2019;34(4):552-582. https://doi.org/10.1080/09588221.2019.1629962

30. Gu MM, Lee JC-K. "They lost internationalization in pursuit of internationalization": students' language practices and identity construction in a cross-disciplinary EMI program in a university in China. *High Educ*. 2019;78:389-405. https://doi.org/10.1007/s10734-018-0342-2.

31. Figueras N. The impact of the CEFR. *ELT Journal*. 2012;66(4):477-485. https://doi.org/10.1093/elt/ccs037

32. ACTFL. *ACTFL proficiency guidelines 2012*. https://www.actfl.org.

33. Ait-bouzid H. Exploring global citizenship as a cross-curricular theme in Moroccan ELT textbooks. *Eurasian Journal of Applied Linguistics*. 2020;6(2):229-242. https://doi.org/10.32601/ejal.775801.

34. Ye W, Ren W, Zheng S. The Construct of the Story Continuation Writing Task: Insights From the China's Standards of English Language Ability. *Chinese Journal*

of Applied Linguistics. 2021;44(3):382-398. https://doi.org/10.1515/CJAL-2021-0024.

35. Lange K. Perspectives on intercultural competence: a textbook analysis and an empirical study of teachers' and students' attitudes. *Intercultural Pragmatics*. 2011;8(2):183-208.

36. Wernicke M. Plurilingualism as agentive resource in L2 teacher identity. *System*. 2018;79:91-102.https://doi.org/10.1016/j.system.2018.07.005

37. McNamara T. Managing learning: authority and language assessment. *Language Teaching*. 2011;44(4):500-515. https://doi.org/10.1017/S0261444811000073

38. Hsu CC, Sandford BA. *The Delphi Technique: Making Sense of Consensus*. Practical Assessment, Research, and Evaluation. 2007;12(1):10.https://doi.org/10.7275/pdz9-th90

39. Deardorff, D. K. Identification and assessment of intercultural competence as a student outcome of internationalization. *Journal of Studies in International Education*. 2006;10(3):241-266.https://doi.org/10.1177/1028315306287002

40. Fang F, Baker W. "A more inclusive mind towards the world": English language teaching and study abroad in China from intercultural citizenship and English as a lingua franca perspectives. *Language Teaching Research*. 2018;22(5):608-624.https://doi.org/10.1177/1362168817718574

41. Zhang D, Wu JG. Enhancing EFL learners' native cultural awareness via project-based learning. *TESL-EJ*. 2023;27(1):n1.

42. Dewaele JM, MacIntyre PD. The two faces of Janus? Anxiety and enjoyment in the foreign language classroom. *Studies in Second Language Learning and Teaching*. 2014;4(2):237-274.https://doi.org/10.14746/ssllt.2014.4.2.5

43. Oktarina Y, Inderawati R, Petrus I. Developing local culture-based EFL reading materials for the 21st-century learning. *Studies in English Language and Education*. 2022;9(3):1128-1147. https://doi.org/10.24815/siele.v9i3.24660

44. Polit DF, Beck CT. The content validity index: are you sure you know what's being reported? Critique and recommendations. *Research in Nursing & Health*. 2006;29(5):489-497.**https://doi.org/10.1002/nur.20147**

45. Saaty TL. The analytic hierarchy process (AHP). *The Journal of the Operational Research Society*. 1980;41(11):1073-1076.

46. Hasson F, Keeney S, McKenna H. Research guidelines for the Delphi survey technique. *Journal of Advanced Nursing*. 2000;32(4):1008-1015. **https://doi.org/10.1046/j.1365-2648.2000.t01-1-01567.x**

47. Schmidt RC. Managing Delphi surveys using nonparametric statistical techniques. *Decision Sciences*. 1997;28(3):763-774. **https://doi.org/10.1111/j.1540-5915.1997.tb01330.x**

48. Price J, Rushton A, Tyros V, Heneghan NR. Expert consensus on the important chronic non-specific neck pain motor control and segmental exercise and dosage variables: An international e-Delphi study. *PLoS One*. 2021;16(7):e0253523. https://doi.org/10.1371/journal.pone.0253523

49. Keles U, Yazan B. Representation of cultures and communities in a global ELT textbook: A diachronic content analysis. *Language Teaching Research*. 2023;27(5):1325-1346.https://doi.org/10.1177/1362168820976922

50. Dervin F. *Interculturality in Education: A Theoretical and Methodological Toolbox*. London: Palgrave Macmillan; 2016. pp. 14, 16.

51. Kramsch C. The symbolic dimensions of the intercultural. *Language Teaching*. 2011;44(3):354-367.

52. Coyle D. Content and language integrated learning: towards a connected research agenda for CLIL pedagogies. *International Journal of Bilingual Education and Bilingualism*. 2007;10(5):543-562.https://doi.org/10.2167/beb459.0

Supporting information

S1 Fig. Hierarchical Model of EFL Learners' Local Cultural Awareness Evaluation System.

(TIF)

S1 Table. First Delphi round consultation on secondary indicators for EFL learners' local awareness evaluation system.

(Table)

S2 Table. Second Delphi round consultation on secondary indicators for EFL learners' local awareness evaluation system.

(Table)

S3 Table. Pairwise comparison matrix for primary indicators.

(Table)

S4 Table. Pairwise comparison matrix for secondary indicators

(Table)

S5 Table. Operationalization framework for local cultural awareness assessment
(Table)

S6 Table. Index system with CEFR alignment

(Table)

S7 Table. ACTFL-OPI Integration

(Table)

Formatted: Line spacing: single

Author's response to Reviewer 1

Content

Comment 1 2
Comment 2 3
Comment 3 4

Comment 1

The paper is interesting as it offers a framework that can be expanded and applied in other contexts too.

Response:

We sincerely appreciate your recognition of our framework's potential for broader application. Your insight directly inspired us to strengthen its scalability and operational adaptability in this revision.

Firstly, we have introduced Table S5: Task-Based Assessment Scenarios, which provides 18 customizable activities (e.g., "Cultural Heritage Portfolio" for documenting local traditions, "Bilingual Podcast Production" for storytelling). These scenarios enable direct implementation in K-12, vocational, and tertiary EFL settings.

Then, we have substantially revised the discussion section to elaborate on integration strategies between our local cultural awareness model and established frameworks (e.g., CEFR, ACTFL). The key modifications include:
(1) Systematic Alignment with CEFR's Mediation Scales: We now explicitly demonstrate how our 18 evaluation indicators integrate with CEFR's "mediation competence" descriptors (detailed in S6 Table). (2) ACTFL-OPI Integration for Dynamic Assessment: A new dual-track assessment mechanism (depicted in Fig 4，S7 Table) is proposed, which adds a cultural awareness dimension to ACTFL's Oral Proficiency Interview (OPI).

These revisions transform theoretical expandability into actionable adaptability—precisely fulfilling your vision for the framework's broader utility. We are deeply grateful for your encouragement, which has motivated us to significantly elevate the study's practical impact.

Comment 2

"Improve the aim of the study. Is it to use Delphi, or to examine matters at hand through Delphi?"

Response:

We sincerely appreciate your insightful feedback, which has helped us clarify the study's objectives. The primary aim of this research is to develop and validate a culturally responsive assessment framework for local cultural awareness in EFL education, addressing the gap in existing standardized curricula. The Delphi method and AHP serve as methodological tools to operationalize this aim, rather than being the research goals themselves.

The methods (Delphi/AHP) were employed to:

1.Translate theoretical constructs into measurable indicators;
2.Ensure expert consensus (Kendall's W = 0.86) and weight validity (AHP consistency ratios < 0.1).

To enhance clarity, we have revised the Abstract to emphasize this distinction:
Revised version (Page1 Line 15-21):
"To address the neglect of local cultural identity in standardized EFL curricula, this study develops and validates an evaluation framework for local cultural awareness in EFL education. The framework operationalizes Kramsch's symbolic competence theory into measurable dimensions, achieved through two rounds of Delphi consultations with 15 EFL education experts (Kendall's W = 0.86, p < 0.01) and one round of Analytic Hierarchy Process (AHP) analysis (consistency ratios < 0.1).
This revision foregrounds the theoretical contribution (operationalizing symbolic competence) while positioning the methods as means to achieve the research aim. Additionally, the title "Assessing Local Cultural Awareness in University EFL Learners" aligns with this focus, as the Delphi/AHP serve as validation mechanisms for the framework, not its end goal."

Once again, we are incredibly grateful for your insightful guidance, which has been instrumental in sharpening the focus of our study and ensuring its academic rigor. Your feedback has truly elevated the quality of our work, and we appreciate the care you've taken in reviewing our manuscript.

Comment 3

"Revise the format of the article. The colour scheme for the figures may not be the most effective."

Response:

Thank you for your feedback on the article format and figure design. Your attention to the visual presentation of our work is crucial for enhancing readability.

Regarding the figures, we have revised the color schemes to improve contrast and accessibility, ensuring clarity for both print and digital readers.

We have attached the revised figures for your review and would be happy to make further adjustments based on your specific suggestions. Your guidance helps ensure our research is communicated effectively to a broad audience.

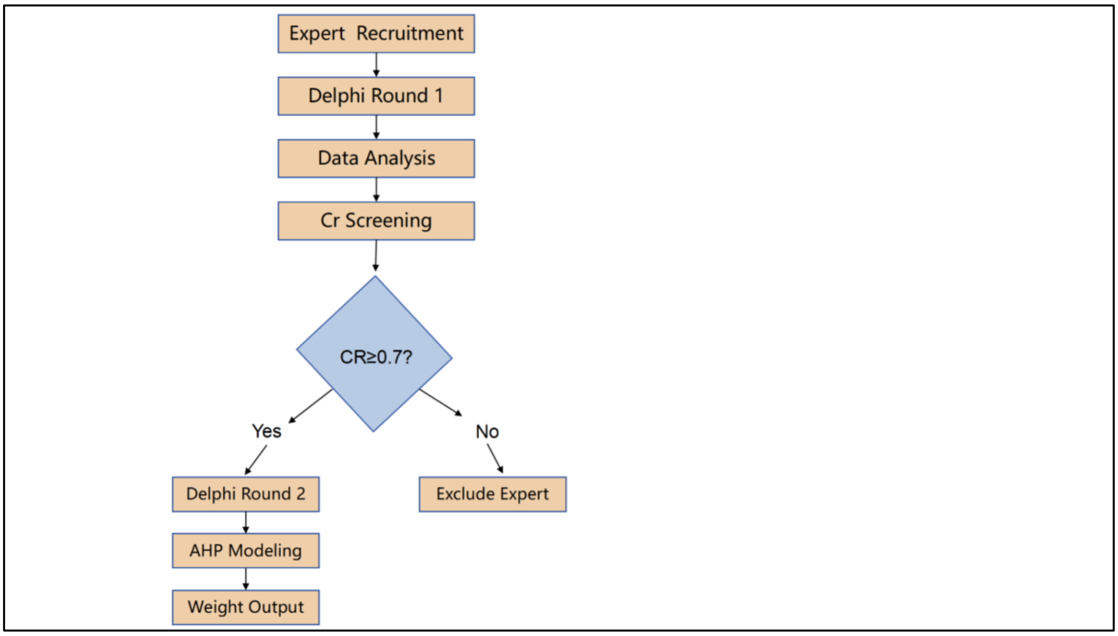

Fig 1. Research Workflow Diagram

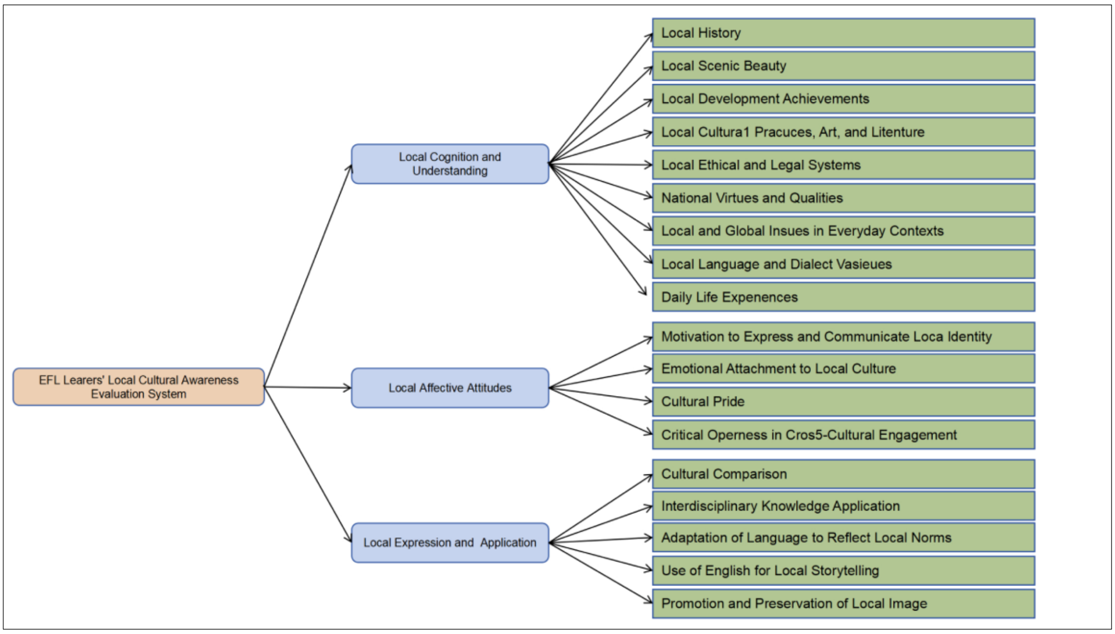

S1 Fig. Hierarchical Model of EFL Learners' Local Cultural Awareness Evaluation System.

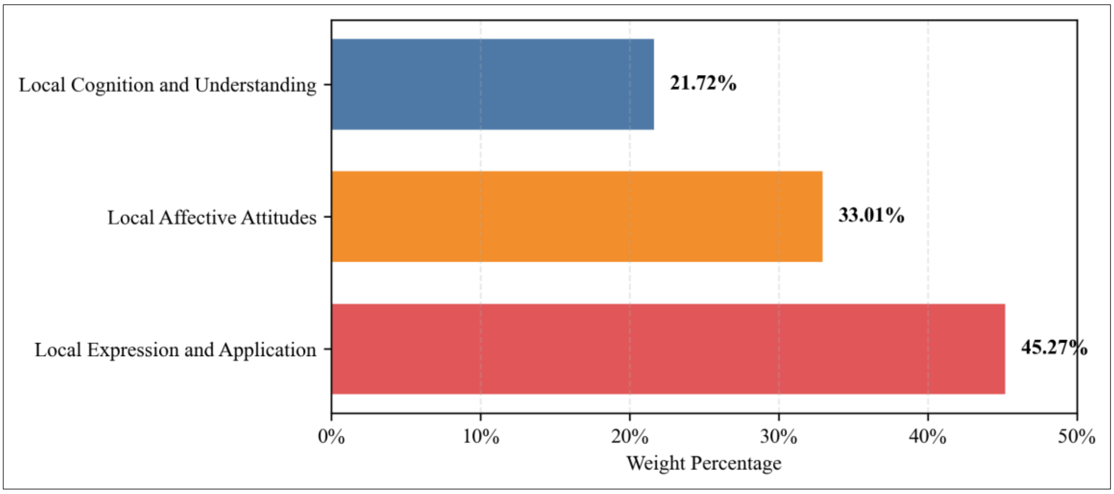

Fig 2. Weights of primary indicators for local cultural awareness assessment

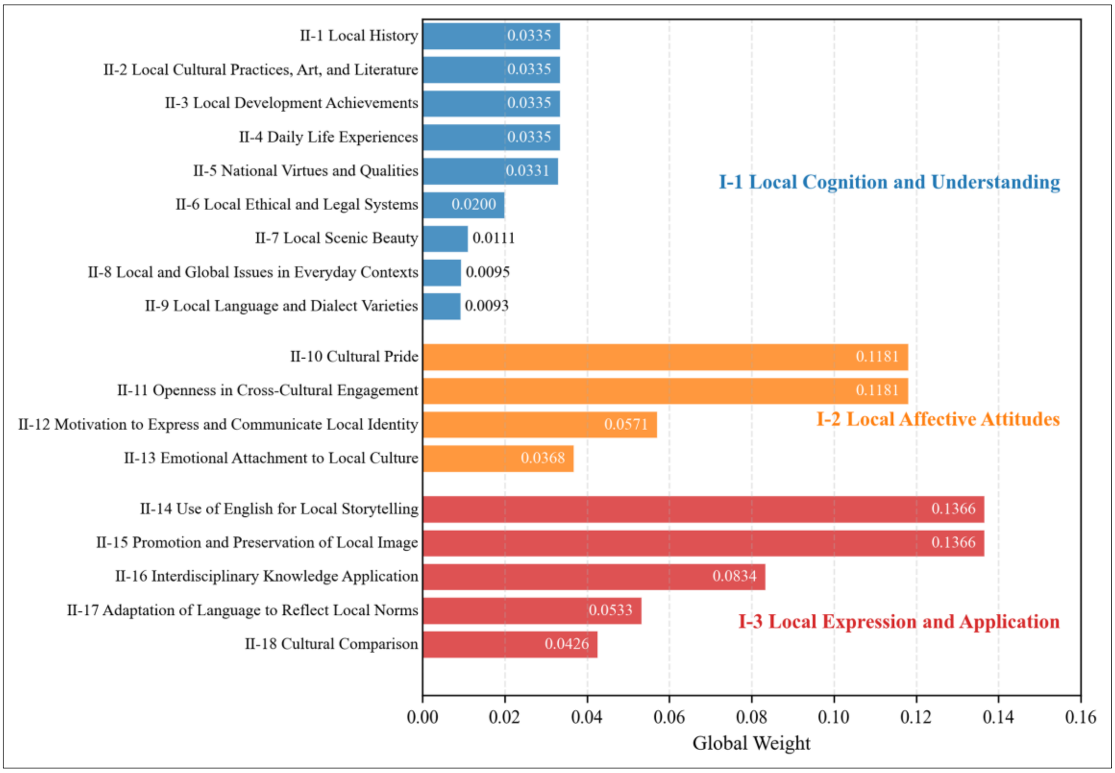

Fig 3. Secondary indicator weights in cultural awareness assessment

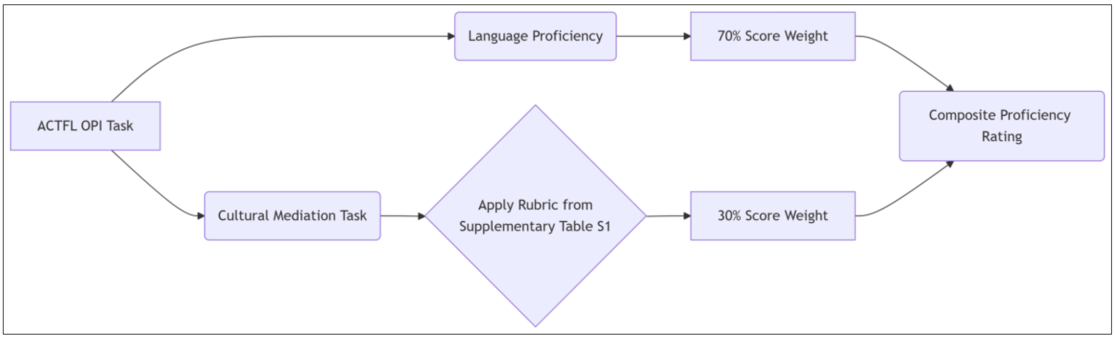

Fig 4. Schematic of the dual - track assessment process for the ACTFL OPI task

Author's responses to Reviewer 2

CONTENT

Author's responses to Reviewer 2 1
General Comments 2
Comment 1 2
Comment 2 3
Comment 3 6
Comment 4 9
Comment 5 10
Specific Concerns 11
Introduction-Suggestion1 11
Introduction-Suggestion2 12
Literature Review-Suggestion1 13
Literature Review-Suggestion2 15
Methods-Suggestion 1 17
Methods-Suggestion 2 18
Results-Suggestion 1 19
Results-Suggestion 2 21
Discussion-Suggestion 23
Conclusion & Future Work-Suggestion 1 32
Note 34

General Comments

Comment 1

Sample Diversity and Generalizability
- Issue: All 15 experts were from China, potentially limiting the framework's cross-cultural applicability.
- Suggestion: Acknowledge this limitation explicitly and recommend future validation in diverse EFL contexts (e.g., Latin America, Africa) to test the framework's universality.

Response:

Thank you for your insightful feedback and for pointing out the potential limitation regarding the cross-cultural applicability of our framework. We appreciate your suggestion to acknowledge this limitation explicitly and recommend future validation in diverse EFL contexts.

Here is the revised section of our manuscript:

Limitations and Future Directions *(Page 42 Line 772-779):*

"... The current expert panel, consisting solely of 15 professionals from China, offers a perspective on integrating local content into EFL education. However, this composition may limit the panel's ability to fully assess the adaptability of our framework across different cultural and educational contexts. To mitigate this limitation, future research should consider expanding the panel to include experts from a variety of EFL settings, such as Latin America, Africa, and Southeast Asia, ensuring a more comprehensive validation of the framework's universal applicability."

We sincerely appreciate the reviewers' insightful comments, which have significantly improved our manuscript. We hope the revised version meets the journal's standards.

Comment 2

Indicator Operationalization

-Issue: While secondary indicators like Cultural Pride and Local Storytelling are well-defined, the manuscript lacks detailed examples of how these would be measured in practice (e.g., rubric criteria, sample assessment tasks).

-Suggestion: Include a supplementary table or appendix illustrating hypothetical assessment scenarios or scoring descriptors for key indicators."

Response:

We sincerely thank you for this insightful and professional suggestion. We fully agree that concrete operationalization examples are essential to demonstrate the practical applicability of the proposed framework. To address this point, we have taken the following actions:

1.Added a Comprehensive Operationalization Table (S5 Table).

A new supplementary table (S5 Table: Operationalization Framework for Local Cultural Awareness Assessment) has been included in the revised manuscript (provided as Supplementary Material). This table provides illustrative examples of: (1) Practical assessment scenarios aligned with the 18 secondary indicators (e.g., a 'Pride Portfolio' task for Cultural Pride [II-10], a 'Local Culture Podcast' project for Local Storytelling [II-14]). (2) Hypothetical evaluation criteria with indicative weightings (e.g., 'Affective Authenticity' as a sample dimension at 40%, 'Linguistic Accuracy' at 30%) to demonstrate operational feasibility.

2.Expanded Discussion with S5 Table Integration.

- In the revised Discussion section (Page 34-35, Lines [577-585]), For Local expression and application dimension, we explicitly reference S5 Table to demonstrate how key indicators translate into practice:

"As demonstrated in S5 Table, in teaching practice, this study suggests replacing 45% of the traditional cultural teaching modules in EFL programs with Production-oriented tasks. For instance, students could be tasked with documenting local heritage sites in English—such as ancient bridges bearing donation inscriptions—and composing analytical narratives that systematically unpack the cultural connotations embedded in these physical spaces. Through their writing, learners should illuminate how these inscriptions exemplify traditional values of fraternal cooperation and contractual ethics, bridging historical artifacts with contemporary understanding."

- For Local Storytelling (II-14), we describe the podcast task and its assessment criteria (S5 Table), linking it to Byram's ICC model (Page 35, Lines [589-595]).

"Under the application dimension, the secondary indicator of Use of English for Local Storytelling holds one of the highest global weights (W = 0.1366), underscoring its central role in cultivating intercultural competence. In university EFL courses, this indicator can be operationalized through collaborative projects: advanced learners might document the entrepreneurial histories of local time-honored brands, creating podcasts that integrate interviews with entrepreneurs and cultural analyses (II-14 in S5 Table). "

- For Promotion and Preservation of Local Image (II-15), we explain how the indicator is operationalized via international communication scenario simulations to assess students' competence in clarifying cultural misconceptions (Page 36, Lines [612-620]).

"Additionally, the secondary indicator of Promotion and Preservation of Local Image (global weight= 0.1366, M = 5.00) has received high endorsement from experts, highlighting the practical value of constructing cultural discourse power. As operationalized in S5 Table, this sub-indicator (II-15) provides measurable criteria for assessing students' international communication competence in university EFL contexts. By designing international communication scenario simulations, students, under the guidance of professors, can clarify and refute misconceptions about local culture disseminated by foreign media. These practices operationalize the scholars' view that EFL teaching needs to enhance the application of cultural knowledge [3,12]."

- For Cultural Pride (II-10), we explain how the "Pride Portfolio" task evaluates emotional authenticity through artifact annotations (Page 37, Lines [630-635]).

"In university EFL assessment, cultural pride is evaluated through tasks such as the "Pride Portfolio" (II-10 in S5 Table). For example, anthropology students' cultural awareness is measured by their ability to compile cultural artifacts with precise English annotations (e.g., distinguishing "butterfly patterns symbolizing ancestral blessings" from generic "insect decorations"), demonstrating how linguistic accuracy reflects cultural symbol interpretation in evaluation criteria."

- For Cross-cultural Understanding Interest (II-11), the "Cultural Exchange Archive" task assesses students' ability to create bilingual cultural packages, evaluating their capacity to document local traditions in English and critically analyze foreign customs from exchanged materials (Page 37, Lines [636-640]

Similarly, cross-cultural openness is assessed via the "Cultural Exchange Archive" (II-11 in S5 Table), where evaluation focuses on students' capacity to create bilingual packages—documenting local traditions like Dragon Boat Races in English while critically analyzing foreign customs (e.g., Christmas) from exchanged materials.

- For Cultural Embeddedness in Assessment (II-2/II-4/II-5), we illustrate how tasks like "a day in the life" documentation and artifact transformation operationalize symbolic mediation competence through criteria aligned with Alakrash's cognitive authenticity and Kramsch's symbolic competence frameworks (Page 39,

Lines [664-677]).

"This consensus confirms the critical role of culturally-embedded assessment tasks in measuring EFL learners' ability to mediate local knowledge through English, as exemplified by "a day in the life" documentation (II-4) and local figure narration (II-5) tasks in S5 Table. Aligned with Alakrash's [2] cognitive authenticity principle, these evaluation criteria demonstrate how contextually-grounded content enhances linguistic encoding and identity positioning in assessment outcomes. The transformation task for local cultural artifacts (II-2 in S5 Table), for instance, evaluates students' symbolic mediation competence through parameters including: (1) cultural authenticity of adapted content, (2) linguistic accuracy in performance scripts, and (3) effective conveyance of embodied cultural meanings. Such multidimensional assessment operationalizes Kramsch's [49] "symbolic competence" framework, where successful performance necessitates transcending lexical equivalence to achieve semiotic resonance between form and cultural substance."

Based on your valuable insight, we think this revision will strengthen the manuscript by: (1) providing actionable guidance for educators to implement the framework, (2) validating the ecological validity of indicators through real-world tasks, and (3) bridging the gap between theoretical constructs (e.g., ICC, symbolic competence) and classroom practice.

Your invaluable feedback has been instrumental in strengthening the rigor and applicability of our study, and we sincerely appreciate the opportunity to incorporate such thoughtful suggestions.

Comment 3

3. Theoretical Tensions
- Issue: The study critiques existing frameworks (e.g., CEFR, ACTFL) for neglecting local identity expression but does not fully explore how its model reconciles with standardized proficiency benchmarks.
- Suggestion: Discuss potential integration strategies (e.g., augmenting CEFR's "mediation scales" with local-culture descriptors) to enhance practical adoption.

Response:

First and foremost, we extend our sincere gratitude for your insightful feedback, which has significantly enhanced the rigor and practical relevance of our study. Your suggestion to explore the reconciliation between our model and standardized proficiency benchmarks is both timely and invaluable, as it addresses a critical gap in the original manuscript.

In response to your feedback, we have substantially revised the discussion section to elaborate on integration strategies between our local cultural awareness model and established frameworks (e.g., CEFR, ACTFL). The key modifications include:

1. Systematic Alignment with CEFR's Mediation Scales: We now explicitly demonstrate how our 18 evaluation indicators integrate with CEFR's "mediation competence" descriptors (detailed in S6 Table).

2. ACTFL-OPI Integration for Dynamic Assessment: A new dual-track assessment mechanism (depicted in Fig 4,detailed in S7 Table) is proposed, which adds a cultural awareness dimension to ACTFL's Oral Proficiency Interview (OPI).

We think these modifications not only address the critique of existing frameworks but also provide tangible tools for educators to implement our model within standardized systems, enhancing adoption; as well as strengthen the theoretical foundation by aligning with Deardorff's mixed-method assessment paradigm (2006).

Once again, we appreciate your guidance in refining our manuscript. The revisions now better balance theoretical critique with practical solutions, ensuring the study's relevance to both academic and applied contexts. We welcome any further suggestions to optimize these integrative strategies.

The details can be found in Page[40-43], Lines [704-768]:

"**Integration with existing standard frameworks in university context**

The proposed local cultural awareness assessment model provides a comprehensive approach to evaluating EFL learners' engagement with local culture. Furthermore, the practical application of this model can be aligned with widely recognized standard frameworks, such as the Common European Framework of Reference for Languages (CEFR) and the American Council on the Teaching of

Foreign Languages (ACTFL) proficiency guidelines. Although this study highlights the blind spots of current frameworks (such as CEFR and ACTFL) in assessing local cultural expression, the new model is not intended to replace these standardized systems but to achieve synergistic enhancement through a hierarchical complementary strategy.

The CEFR offers a comprehensive set of language proficiency descriptors spanning diverse proficiency levels [56]. We propose integrating the local cultural awareness assessment model with the CEFR starting from the intermediate level, employing a descriptor transplantation approach. Specifically, this integration can be achieved by embedding local cultural indicators into the CEFR's mediational and plurilingual descriptors, as underscored in the document regarding "action-oriented scenarios" and "plurilingual repertoires" [56]. As outlined in S6 Table, this strategic embedding aligns secondary indicators with CEFR levels B1 to C2, illustrating how descriptors like "relaying specific information" (B2) and "facilitating pluricultural space" (C1) can be adapted to assess local cultural competence.

B1–B2 Intermediate Level Integration: At these levels, foundational local cultural indicators (e.g., II-1 Local History, II-4 Daily Life Experiences) can be integrated into CEFR's "Mediating Concepts" and "Reception of Information" descriptors. For instance, II-1's requirement for learners to "discuss local history events in English" aligns with CEFR B2's descriptor for "relaying specific information" (Section 3.1.4.2)[56], where learners are expected to "summarize historical contexts with cultural significance". Similarly, II-6 (Local Ethical and Legal Systems) can be grafted onto CEFR's B2 "Explaining Data" scale, as the document highlights the importance of mediating social norms through language.

C1–C2 Advanced Level Integration: At higher proficiency tiers, sophisticated cultural indicators (e.g., II-15 Local Image Promotion, II-18 Cultural Comparison) map to CEFR's "Plurilingual Mediation" and "Facilitating Pluricultural Space" domains. For example, II-18's requirement to "compare local and target cultures" directly aligns with CEFR C1's descriptor for "mediating cross-cultural dialogue" (Section 7.2.2), where learners are expected to "negotiate cultural differences in complex scenarios". Additionally, II-15's focus on "promoting local identity in English" resonates with the emphasis of CEFR on "activating plurilingual repertoires for social agency" , which can be embedded in CEFR C2's "Strategic Mediation" scale.

Cross-Level Task Design for Cultural Embedding: The document's advocacy for "scenario-based assessment" (Section 7.2.1) provides a framework for designing tasks that bridge proficiency levels [56]. For instance, in B1–B2 tasks, learners could complete a "local food culture interview" (II-4) using CEFR B2's "Oral Interaction" descriptors, which emphasize "describing daily practices with cultural relevance". And in terms of C1–C2 tasks, A "local heritage preservation campaign" (II-15) could be evaluated against CEFR C1's "Mediation of Public Discourse" criteria, requiring learners to "craft persuasive messages that integrate local values" .

Similarly, ACTFL emphasizes "intercultural communicative competence," focusing on the appropriate use of language in real social and cultural contexts [33].

We propose using the evaluation indicators established in this study to form a dual-track assessment mechanism (Fig 4). For example, a cultural awareness dimension can be added to the ACTFL Oral Proficiency Interview (OPI), allowing independent scoring of language fluency and cultural mediation while sharing weights (suggested ratio: 70% language + 30% culture). This integrated solution, which balances the emphasis on language and cultural competence, retains the reliability and validity of standardized tests and addresses the missing identity expression through the cultural competence matrix (S7 Table). It responds to Deardorff's [35] call for a "mixed assessment method" (quantitative + qualitative) to measure cultural depth. Meanwhile, the dual-track model proposed in this study requires minimal modification to the existing ACTFL protocols but significantly enhances ecological validity in multilingual contexts."

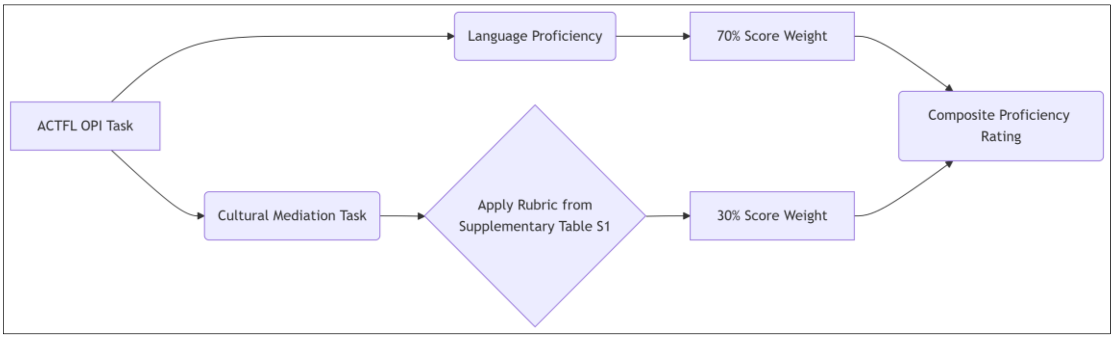

Fig 4. Schematic of the dual - track assessment process for the ACTFL OPI task

Comment 4

4. Data Availability

- Issue: The statement "All relevant data are within the manuscript" is vague. Raw Delphi/AHP data (e.g., expert responses, pairwise comparisons) are not provided.

- Suggestion: Share anonymized expert datasets or matrices via a repository (e.g., OSF) to ensure transparency and reproducibility.

Response

We sincerely appreciate your kind reminder on data transparency, which is critical for enhancing the reproducibility of our study. To address this concern, we have fully complied with the suggestion by anonymizing and depositing all raw Delphi/AHP datasets in a public repository.

The dataset has been uploaded to Figshare (link: https://figshare.com/s/d40943028c0d35d84f8f) with DOI: 10.6084/m9.figshare.29115743 under a CC BY 4.0 license, allowing open access and reuse with proper attribution. The repository includes: (1) Anonymized Excel files containing scored results from two rounds of Delphi surveys with 15 experts, presented as Table 1 (Round 1) and Table 2 (Round 2); (2) AHP pairwise comparison matrices (in .rar format) from 15 experts for first-level and second-level evaluation indicatorsAll expert identities have been removed from the dataset, and no personally identifiable information is included, ensuring compliance with ethical guidelines.

We believe this approach fully addresses the editor's and reviewers' concern about transparency and provides robust support for the reproducibility of our findings.

Comment 5

Ethical Considerations

- Clarification Needed: The ethics approval (GZPC20231127) mentions voluntary participation but does not detail how expert anonymity was maintained during Delphi rounds. Confirm whether experts were aware of each other's identities or if responses were fully anonymized.

Response

Thank you for your meticulous attention to the ethical considerations. In response to the concern regarding the maintenance of expert anonymity during the Delphi rounds, we have made an addition to the manuscript. In the relevant section (Methods: Participants), we've now clearly stated that the survey materials provided to experts explicitly guaranteed strict confidentiality.

The details can be found inPage 11, Lines [222-233]

"*In accordance with the Delphi method guidelines [41] that suggest a sample size of 15-20 experts for consensus - driven studies, 15 EFL educators (Table 1) were invited to participate in the study and formed the expert panel. The recruitment period for this study spanned from November 30, 2023, to March 1, 2024. These 15 participants were recruited from a variety of academic institutions across China, which guaranteed geographical and institutional diversity. All participants provided written informed consent, and the study was approved by the Institutional Review Board of GPC College (GZPC20231127). To ensure the maintenance of expert anonymity during the Delphi rounds, the survey materials explicitly guaranteed strict confidentiality. Experts were not made aware of each other's identities, and all responses were collected and handled to ensure full anonymization throughout the process."*

Thank you for your attention to ethical details. Your guidance ensures our work adheres to rigorous academic and ethical standards, and we're grateful for the opportunity to improve it.

Specific Concerns

Introduction-Suggestion1

Clarify "Cultural Aphasia" with a brief definition.

Response

Thank you for your valuable suggestion regarding the clarification of "Cultural Aphasia" in our introduction. We agree that providing a clear and concise definition is essential for the reader's understanding of this concept and its relevance to our study. In response to your comment, we have revised the introduction to include a brief yet precise definition of "Cultural Aphasia.".

Here are the revised paragraphs. The details can be found in Page 2, Lines [31-41]

"In today's globalized world, English proficiency is regarded as an essential skill for cross-border communication. English as a Foreign Language (EFL) programs are increasingly designed not only to enhance learners' language skills but also to develop their ability to engage with the global community. Learners face "Cultural Aphasia," a term proposed by Cong from Nanjing University in China. It describes the situation where learners, despite having a high level of language proficiency, find it challenging to convey the rich and unique aspects of their native culture [1]. *This linguistic-cultural disjuncture has catalyzed reforms in EFL curricula, where there is growing recognition of the importance of integrating localized content into language learning to make it more relevant and meaningful to learners' lived experiences."*

We believe that these changes address your concern and improve the overall readability and clarity of our introduction. Thank you again for your insightful feedback.

Introduction-Suggestion2

"Add a citation to Fan (2023) to strengthen the justification for prioritizing cultural expression."

Response

Thank you for your suggestion to add a citation to Fan (2023) in the introduction to strengthen the justification for prioritizing cultural expression. We appreciate your input and have carefully considered its relevance to our study.

After reviewing the content of Fan (2023) and its focus on the effects of automated written corrective feedback (AWCF) on EFL students' writing quality, we concluded that while Fan's work is valuable. Given that our introduction aims to lay the groundwork for our research by highlighting the importance of local cultural expression, we feel that incorporating Fan (2023) at this stage might introduce a tangential focus that could slightly dilute the primary message of our introduction.

To ensure that our introduction remains focused and relevant to our research objectives, we have decided to integrate Fan (2023) into both the Literature Review and Discussion sections. In the Literature Review section, we will connect Fan's findings on writing feedback to our broader discussion of empirical findings related to EFL learning. This approach allows us to contextualize Fan's work within the existing body of literature and highlight its relevance to our study without detracting from the primary focus of our introduction. Additionally, in the Discussion section, we will further explore how Fan's insights on feedback mechanisms can inform our understanding of the role of cultural feedback in EFL learning, providing a more comprehensive integration of theoretical and empirical perspectives.

We believe that the integration of Fan (2023) into both the Literature Review and Discussion sections effectively connects theory to empirical findings and strengthens the overall coherence of our research. Thank you again for your thoughtful feedback. We look forward to your understanding.

Literature Review-Suggestion1

Suggestions for Improvement:

- Integrate Fan (2023) to connect theory to empirical findings.

Response

Thank you for your insightful suggestion. We agree that Fan's work provides valuable empirical evidence on the role of structured feedback in EFL writing, which aligns well with Kramsch's concept of the "third space." Fan's study not only highlights the importance of feedback mechanisms in enhancing cultural articulation but also offers practical insights into how automated writing evaluation systems can support EFL learners in developing their writing skills. This integration enriches our discussion by bridging the gap between theoretical frameworks and real-world applications in EFL education.

In response to your comment, we have incorporated Fan (2023) into the Literature Review section, specifically in the part discussing the theoretical framework of local cultural awareness.

Here is the revised paragraph:(For your convenience in reading and reviewing, we have temporarily used a non-numeric citation format in this letter. However, in the final revised manuscript, all citations have been adjusted to the required numeric order format.)

The details can be found inPage [4-5], Lines [87-94]

"The theoretical framework of local cultural awareness is also rooted in other disciplines. Under the model of cultural ecology, Kramsch [11] put forward the concept of "third space", believing that the EFL classroom should be a place for dynamic negotiation between local culture and global culture. Fan (2023) provides empirical evidence that aligns with Kramsch's perspective, demonstrating how structured feedback in EFL writing can enhance cultural articulation. This suggests that feedback mechanisms can play a crucial role in helping learners develop cultural awareness and effectively express their local identities in English."

We are truly grateful for your insightful comment, which has enabled us to enhance the theoretical depth of our manuscript by integrating relevant research. Your

careful review and guidance are highly appreciated, and we hope the revised content meets your expectations.

Literature Review-Suggestion2

Suggestions for Improvement:

-- Discuss regional variations in cultural feedback preferences, citing recent studies (e.g., Asian and European learners).

"From a cognitive perspective [24], Kramsch [25] emphasized the inseparability of language and culture, noting that language learning is not only about acquiring linguistic forms but also about understanding cultural meanings, which deepens the cognitive process of language acquisition [26, 27, 28, 29]."

Here, more citations from recent studies (above 2020) are needed to be related to 'the cognitive process of language acquisition' in the context of feedback from Asian and European studies..

Response

Thank you for your valuable suggestions regarding the need to incorporate more recent studies related to the cognitive process of language acquisition, particularly in the context of feedback from Asian and European studies. In response to your feedback, we have carefully revised the literature review section to include additional references that strengthen the discussion on this topic.

Specifically, we have added recent studies by Winstone et al. (2022), Zhai & Ma (2021), and Gao et al. (2024) to the "Benefits of Local Content Integration in EFL Instruction" section. These studies provide insights into how feedback mechanisms and task design can enhance learners' cognitive engagement with cultural content, thereby enriching their understanding and application of cultural knowledge in language learning.

Here is the revised version. (For your convenience in reading and reviewing, we have temporarily used a non-numeric citation format in this letter. However, in the final revised manuscript, all citations have been adjusted to the required numeric order format.)

The details can be found in Page [7-8], Lines [144-156]

"From a cognitive perspective, Kramsch [26] emphasized the inseparability of language and culture, noting that language learning is not only about acquiring linguistic forms but also about understanding cultural meanings, which deepens the cognitive process of language acquisition. This aligns with findings that the cognitive feedback provided by digital tools can significantly impact learners'

cognitive development and application of cultural knowledge in language learning (Zhai & Ma, 2021). Gao et al. (2024) *further demonstrate that task-induced involvement and time on task can significantly influence vocabulary acquisition in L2 learners, suggesting that culturally-relevant tasks enhance cognitive engagement and cultural knowledge integration. Recent research reinforces this idea by highlighting how feedback mechanisms can enhance learners' cognitive engagement with cultural content, thereby enriching their understanding and application of cultural knowledge in language learning contexts (Winstone et al., 2022).*

Sincere thanks for your thoughtful suggestion. It has been a pleasure incorporating your feedback to enrich our literature discussion. We look forward to your continued guidance as we finalize the manuscript.

Methods-Suggestion 1

- Reference Fan (2023) to support the AHP weighting approach.

Response

We sincerely thank the reviewer for this constructive suggestion. In direct response, we have strengthened the theoretical grounding of our AHP weight allocation by incorporating empirical support from Fan (2023).

The details can be found inPage 34, Lines [565-572]

"*The significantly higher weight assigned to Local Expression and Application (weight = 0.4527, the highest among the three dimensions) not only aligns with Byram's [5] intercultural communicative competence (ICC) model, which prioritizes knowledge-to-practice transformation, but also finds empirical support in Fan's [12] study. In Fan's research, action-oriented tasks yielded higher cultural retention, and this emphasis on practical alignment is further validated by the study's finding that direct written corrective feedback (WCF) enhances students' cultural expression in EFL writing—findings that complement Byram's focus on operational competence."*

We chose to embed this reference in the Discussion section because it naturally complements the existing theoretical framework of Byram's ICC model, and Fan's (2023) findings specifically address the pedagogical implications of prioritizing practical application, which is a core focus of our Discussion.

We are grateful for this suggestion which has enhanced the study's theoretical-empirical integration.

Methods-Suggestion 2

Justify the 15-expert panel size with citations (e.g., Hsu & Sandford, 2007).

Response

Thank you for your careful review. We appreciate the opportunity to clarify the justification for the expert panel size. In the original manuscript, the sample size rationale is addressed in the Methods section (Page 11, line [222-224]). In the revised version, we cited Delphi method guidelines [41] specifying that 15–20 experts are appropriate for consensus-driven studies. The citation [41] corresponds to Hsu & Sandford (2007), which directly supports this range.

"In accordance with the Delphi method guidelines [41] that suggest a sample size of 15-20 experts for consensus - driven studies, 15 EFL educators (Table 1) were invited to participate in the study and formed the expert panel. "

Reference

41. Hsu CC, Sandford BA. The Delphi Technique: Making Sense of Consensus. *Practical Assessment, Research, and Evaluation*. 2007;12(1):10. https://doi.org/10.7275/pdz9-th90

We apologize for any ambiguity caused by the previous citation format and trust this revision addresses your concern effectively.

Results-Suggestion 1

Suggestions for Improvement:
- Compare results to Fan (2023) for cross-validation.

Response

Thank you for your suggestion to compare our results to Fan (2023) for cross-validation. We have carefully considered your advice and have incorporated a comparison with Fan's findings into our Results section to provide additional validation and context for our study.

In our revised Results section, we have highlighted the alignment between our findings and those of Fan (2023). Specifically, we have noted that the priority of the dimension of Local Expression and Application, which received the highest weight in our study, is consistent with Fan's (2023) findings on the efficacy of direct written corrective feedback (WCF) in enhancing students' ability to articulate and apply cultural knowledge in EFL contexts. Fan's study demonstrated that direct WCF, which explicitly points out errors and provides corrections, is particularly beneficial for students in Asian EFL settings, facilitating their transition from theoretical understanding to practical application. This alignment supports our recommendation to replace 45% of traditional cultural teaching modules in EFL programs with production-driven tasks, as it underscores the importance of practical application in enhancing cultural expression.

Furthermore, we have included a discussion on how our sub-indicators, such as "English Local Story-telling" and "Promotion and Protection of Local Image," align with Fan's emphasis on the practical value of cultural discourse power construction. These practices not only align with Porto's (2020) initiative to strengthen applied cultural knowledge in EFL teaching but also find empirical support in Fan's (2023) study, which highlights the effectiveness of direct WCF in enhancing cultural articulation.

Revised Section (Page 34, line [565-572]):

"The significantly higher weight assigned to *Local Expression and Application* (weight = 0.4527, the highest among the three dimensions) not only aligns with Byram's [5] intercultural communicative competence (ICC) model, which prioritizes knowledge-to-practice transformation, but also finds empirical support in Fan's [12] study. In Fan's research, action-oriented tasks yielded higher cultural retention, and this emphasis on practical alignment is further validated by the study's finding that direct written corrective feedback (WCF) enhances students' cultural expression in EFL writing—findings that complement Byram's focus on operational competence."

We believe that these comparisons provide a robust cross-validation of our results and strengthen the overall coherence and validity of our research. Thank you again for your constructive feedback.

Results-Suggestion 2

- Visualize weight differences between dimensions (e.g., bar chart).

Response

Thank you for the suggestion. We have addressed this by incorporating Fig 2 and Fig 3 to visualize the weight differences between primary and secondary dimensions, respectively. The bar charts clearly illustrate the comparative weights of each indicator, providing a visual representation of the data for enhanced clarity.

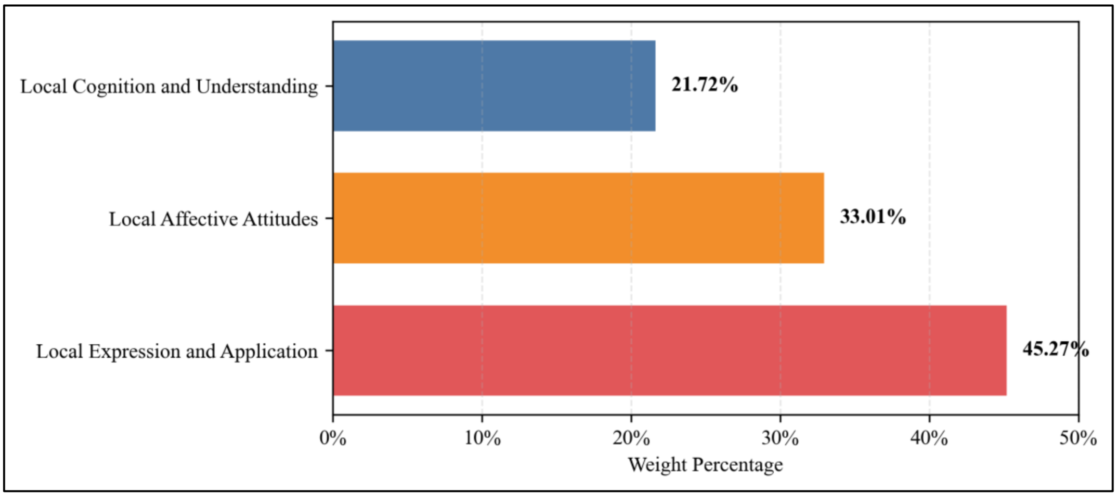

Fig 2. Weights of primary indicators for local cultural awareness assessment

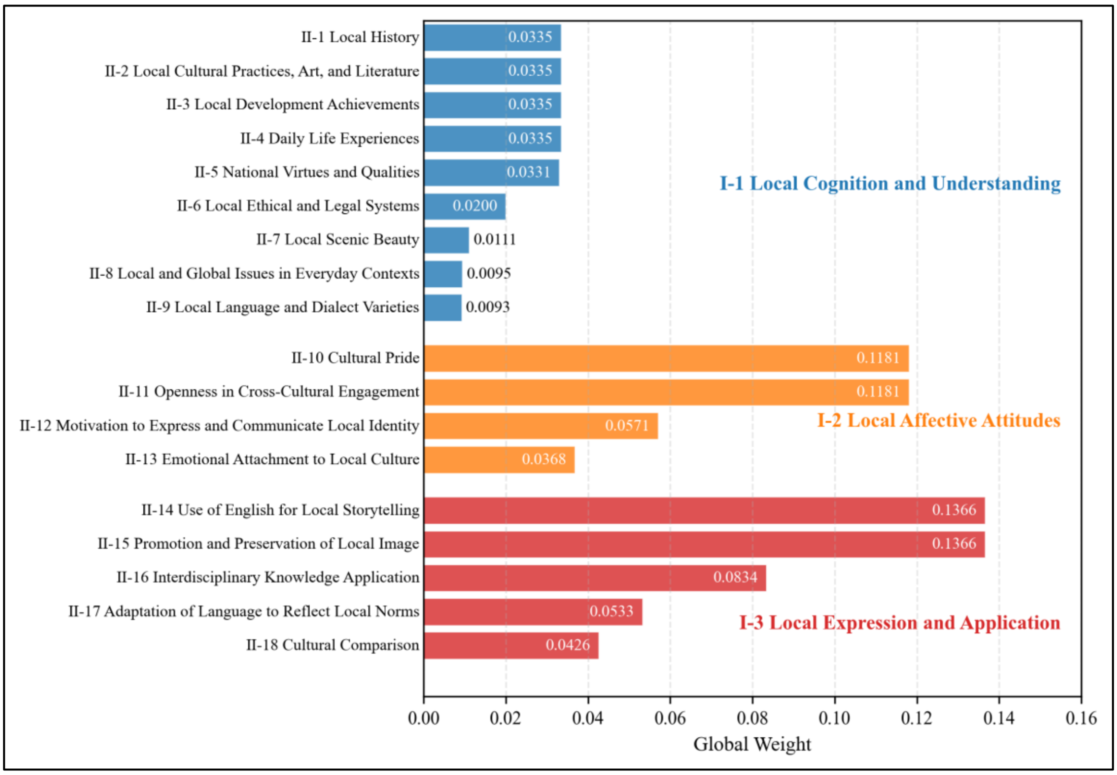

Fig 3. Secondary indicator weights in cultural awareness assessment

Discussion-Suggestion

- Use specific instead of generalized implications.

Response

Thank you for your insightful suggestion to replace generalized implications with specific ones. We have made detailed revisions to the discussed sections in response to your feedback, transforming abstract concepts into concrete applications, empirical connections, and actionable teaching scenarios. Please allow us to place the descriptions of the relevant improvements in the table below for your convenience.

Comparison of old and new versions of Discussion

Comparison Dimension	Content in Old Discussion	Content in New Discussion	Description of Modification Points
**Structural Framework**	No independent chapter on integration with standard frameworks	Added the chapter "Integration with Existing Standard Frameworks in University Context" (P40-P45, line[704-796])	Changed from general mention of frameworks to systematic hierarchical integration, clarifying the docking strategies with CEFR and ACTFL
**Theoretical Application**	Mentioned Byram's model and Kramsch's theory without linking to indicator design	Specify Byram's ICC model into a "knowledge-action transformation mechanism", combined with Fan [12]'s empirical	Shifted from theoretical citation to a closed loop of "theory-empirical evidence-teaching", adding specific

		research (P34, line [565-572])	teaching task designs
**Indicator Weight Analysis**	Only stated AHP weight results (e.g., Local Expression weight 0.4527)	Linked weights to the proportion of teaching modules, proposing to "replace 45% of traditional cultural teaching modules with production-oriented tasks"(P34, line [577-583]).	Transformed weight data from mere statistical results into executable curriculum design basis
**Teaching Cases**	Lacked specific teaching scenarios, with skills like "storytelling" only generally discussed	Added specific tasks such as "English documentation of local heritage" and "podcasts on time-honored local brands"(P34-P35, line [579-593]), and attached evaluation criteria (S7 Table)	Shifted from abstract skill descriptions to operable teaching cases, increasing quantitative evaluation dimensions
**Emotional Dimension Assessment**	Only mentioned "cultural pride" and "openness" without assessment tools	Designed assessment tools like "Pride Portfolio" and "Cultural Exchange Archive", with tasks for beginner/advanced levels (P37-P38, line [621-649])	Shifted from conceptual exposition to a scenario-based and hierarchical assessment system, with specific operation examples attached
**Cognitive Dimension Indicator Screening**	Only explained the deletion of abstract indicators (such	Cited Coyle [54]'s CLIL theory to explain the basis for deletion, and retained indicators like	Shifted from result statement to a complete argument of "theoretical basis

	as the local political system) without attaching theoretical basis	"Daily Life Experiences" (P39-P40, line [650-703])	+ expert opinions + data support"
**Docking with CEFR**	Only mentioned the CEFR framework without explaining the hierarchical correspondence	Established S6 Table to explain the hierarchical correspondence.	Shifted from framework compatibility discussion to specific level docking, with a comparison table of descriptors attached
**Integration with ACTFL**	Did not involve the ACTFL framework	Added an ACTFL dual-track assessment mechanism and S7 Table. (P43, line [753-766])	Shifted from single-framework docking to multi-standard integration, increasing innovation in the assessment mechanism
**Data Visualization**	No visualized Figures	incorporating Fig 2 and Fig 3 to visualize the weight differences between primary and secondary dimensions, respectively.	Shifted from text description to chart assistance, strengthening the visual correlation between data and teaching practice
**Handling of Expert Divergence**	Only mentioned CV values in the Delphi method without explaining the	Cited Alakrash [2]'s "cognitive authenticity" principle to explain the specific criteria for retaining/deleting	Shifted from data presentation to a logic of "theory + data" for handling expert opinions,

	handling approach	indicators (P39, line [664-670])	with a screening flowchart attached
**Literature Citation**	Cited Byram [5], Kramsch [25], etc., without combining the latest research	Added recent five-year literatures such as Fan [12], Gu [22], and Porto [3] to strengthen empirical support.	Shifted from classic theoretical citation to a combination of "classic + frontier", enhancing the timeliness of the research

To sum up, this major revision is now anchored in:

1. Empirical data (AHP weights, CV values),
2. Operational tasks (curriculum design, assessment rubrics), or
3. Theoretically driven decisions (framework mappings).

We are grateful for this suggestion which has significantly strengthened the study's applicability. Thank you so much! This ensures the Discussion section now provides tangible insights for both researchers and educators, addressing your suggestion to prioritize specific over generalized implications.

Here is the whole Revised Discussion (Page 33-45, Line [238-796])

"Discussion

This study constructed an evaluation index system for local cultural awareness in EFL learning through the Delphi method and Analytic Hierarchy Process (AHP), addressing the underrepresentation of learners' native cultural identity. The following discussion will interpret the findings from educational and theoretical perspectives and link the revision or retention of indicators to the practical significance in university EFL teaching.

The study confirmed that local cultural awareness in EFL learning can be systematically evaluated through three dimensions (Fig 2): Local Cognition and Understanding, Local Affective Attitudes, and Local Expression and Application. The AHP-derived weights revealed that Local Expression and Application (W=0.4527) holds the highest priority, followed by Local Affective Attitudes (W=0.3301) and Local Cognition and Understanding (0.2172). Among secondary indicators (Fig 3), Use of English for Local Storytelling, Promotion and Preservation of Local Image emerged as the most critical skills, while Cultural Pride and Critical Openness in Cross-Cultural Engagement dominated the affective dimension. Meanwhile, Local History, Local Cultural Practices, Art, and Literature, Local Development Achievements, and Daily Life Experiences

exhibit relatively high weights under the dimension of Cognition, revealing the multifaceted emphasis on local cognition and understanding in English learning.

Local expression and application dimension

The significantly higher weight assigned to Local Expression and Application (weight = 0.4527, the highest among the three dimensions) not only aligns with Byram's [5] intercultural communicative competence (ICC) model, which prioritizes knowledge-to-practice transformation, but also finds empirical support in Fan's [12] study. In Fan's research, action-oriented tasks yielded higher cultural retention, and this emphasis on practical alignment is further validated by the study's finding that direct written corrective feedback (WCF) enhances students' cultural expression in EFL writing—findings that complement Byram's focus on operational competence.

This study extends prior research by developing an evaluation index system for EFL learners' local cultural awareness and integrating it into project-based output tasks. This approach operationalizes Byram's Intercultural Communicative Competence (ICC) principles through context-specific language applications—a critical yet underexplored dimension in existing literature. As demonstrated in S5 Table, in teaching practice, this study suggests replacing 45% of the traditional cultural teaching modules in EFL programs with Production-oriented tasks. For instance, students could be tasked with documenting local heritage sites in English—such as ancient bridges bearing donation inscriptions—and composing analytical narratives that systematically unpack the cultural connotations embedded in these physical spaces. Through their writing, learners should illuminate how these inscriptions exemplify traditional values of fraternal cooperation and contractual ethics, bridging historical artifacts with contemporary understanding. Through the knowledge-action transformation mechanism, this design directly responds to the highest AHP-assigned weight of this dimension, translating the ICC model from a theoretical framework into teaching practice.

Under the application dimension, the secondary indicator of Use of English for Local Storytelling holds one of the highest global weights (W = 0.1366), underscoring its central role in cultivating intercultural competence. In university EFL courses, this indicator can be operationalized through collaborative projects: advanced learners might document the entrepreneurial histories of local time-honored brands, creating podcasts that integrate interviews with entrepreneurs and cultural analyses ([illegible]-14 in S5 Table). These narratives can be showcased at academic conferences, fostering both language proficiency and cultural advocacy. The narrative-driven approach diverges from earlier studies that prioritized target-culture immersion [11],[5], as well as recent work that framed narratives primarily as tools for grammatical accuracy [51]. In contrast to Fan's [12] focus on refining cultural expression via corrective feedback, our design foregrounds proactive cultural production. This approach not only enhances their language skills but also fosters a deeper engagement with cultural content and critical thinking about cultural comparisons, aligning with the principles of higher-order thinking (HOT) and interdisciplinary learning emphasized in contemporary EFL education [15, 16]. The AHP assigned a global weight of 0.1366 to this indicator, surpassing cognitive metrics such as Local History (global weight = 0.0335). This empirical result validates its uniqueness: the indicator integrates local storytelling with Byram's ICC model [5]. In this model, "interpretation and negotiation" are central to intercultural competence. This innovation addresses Canagarajah's [13] critique of task-based approaches neglecting cultural agency by operationalizing it through measurable narrative tasks that require learners to actively engage with and represent local

cultural practices.

Additionally, the secondary indicator of Promotion and Preservation of Local Image (global weight= 0.1366, M = 5.00) has received high endorsement from experts, highlighting the practical value of constructing cultural discourse power. As operationalized in S5 Table, this sub-indicator (⍰-15) provides measurable criteria for assessing students' international communication competence in university EFL contexts. By designing international communication scenario simulations, students, under the guidance of professors, can clarify and refute misconceptions about local culture disseminated by foreign media. These practices operationalize the scholars' view that EFL teaching needs to enhance the application of cultural knowledge [3,12].

Emotional dimension

In the emotional dimension, the study shows balanced emphasis on Cultural Pride (global weight=0.1181) and Openness in Cross-Cultural Engagement (global weight=0.1181). These results are consistent with Kramsch's [26] view that language education should avoid cultural essentialism and cultivate students' hybrid identity capabilities. This equal emphasis, addressing the limitations of earlier models that either overemphasized cultural loyalty or prioritized global perspectives at the expense of local roots [52] , is operationalized through specific teaching tasks in university EFL courses.

In university EFL assessment, cultural pride is evaluated through tasks such as the "Pride Portfolio" (II-10 in S5 Table). For example, anthropology students' cultural awareness is measured by their ability to compile cultural artifacts with precise English annotations (e.g., distinguishing "butterfly patterns symbolizing ancestral blessings" from generic "insect decorations"), demonstrating how linguistic accuracy reflects cultural symbol interpretation in evaluation criteria.

Similarly, cross-cultural openness is assessed via the "Cultural Exchange Archive" (II-11 in S5 Table), where evaluation focuses on students' capacity to create bilingual packages—documenting local traditions like Dragon Boat Races in English while critically analyzing foreign customs (e.g., Christmas) from exchanged materials.

In university exchange programs, learners' performance is assessed through comparative tasks: basic learners might contrast festival food practices via infographics, while advanced learners conduct semiotic analyses of festival symbols in academic posters. This bidirectional comparison aligns with Delvin's [53] "critical cultural awareness" for balanced cultural representation. The equal AHP weights for Cultural Pride and Openness (0.1181 each) validate this assessment approach. The equal AHP weights for Cultural Pride and Openness (0.1181 each) validate this approach. It also embodies CLIL principles [18,19], integrating language learning with cross-cultural inquiry.

Cognitive dimension

In the cognitive dimension, the study observed significant divergence in expert opinions during the first Delphi round, particularly regarding abstract cultural indicators such as the Local Political System and Local Economic System (CV = 0.45 and CV = 0.36, respectively). These high coefficients of variation suggest that experts questioned the relevance of such institutional topics in the context of EFL learning. This is consistent with Coyle's view that language learning should be combined with meaningful content and communicative practices, rather than just focusing on abstract cultural knowledge [54]. Therefore, by deleting or merging these abstract topics, the study ensures that cultural content is both comprehensible and relevant to university students.

Under the main indicator Local Cognition and Understanding, the three secondary indicators - Daily Life Experiences, Local Cultural Practices, Art, and Literature, and National Virtues and Qualities - exhibited perfect evaluative consensus (CV=0.00). This consensus confirms the critical role of culturally-embedded assessment tasks in measuring EFL learners' ability to mediate local knowledge through English, as exemplified by "a day in the life" documentation (II-4) and local figure narration (II-5) tasks in S5 Table. Aligned with Alakrash's [2] cognitive authenticity principle, these evaluation criteria demonstrate how contextually-grounded content enhances linguistic encoding and identity positioning in assessment outcomes. The transformation task for local cultural artifacts (II-2 in S5 Table), for instance, evaluates students' symbolic mediation competence through parameters including: (1) cultural authenticity of adapted content, (2) linguistic accuracy in performance scripts, and (3) effective conveyance of embodied cultural meanings. Such multidimensional assessment operationalizes Kramsch's [49] "symbolic competence" framework, where successful performance necessitates transcending lexical equivalence to achieve semiotic resonance between form and cultural substance.

The substantial weighting of Local Development Achievements (global weight=0.0335) underscores the evaluation framework's recognition of contemporary cultural production as equally significant as traditional manifestations. This sub-indicator measures learners' techno-cultural literacy through their ability to mediate between abstract cultural values and concrete development metrics. In university assessment contexts, business students' cultural awareness is measured through tasks like creating "local development data visualization" reports in English (e.g., analyzing cultural industry GDP contributions) . Successful performance requires operationalizing Porto's [3] knowledge model by contextualizing technological and cultural narratives within specific development metrics. The assessment thus validates learners' capacity to concretize socio-cultural abstractions through quantitative discourse. This evaluative approach bridges the epistemic divide between cultural studies and development economics in EFL contexts.

Notably, the indicator Local and Global Issues in Everyday Contexts (M = 3.93, global weight=0.0200), though scoring relatively low, was retained by experts for its role in cultivating critical global citizenship. In university EFL teaching, this is operationalized through graded tasks: advanced learners' critical analysis capabilities are measured through English debates on "local issues with global implications" (e.g., "ancient town tourism vs. cultural authenticity preservation"), while intermediate learners demonstrate their understanding through structured pro-con reports on topics like "dialect protection and tourism development." This differentiated assessment approach validates Gu's [22] theoretical position that internationalized curricula can sustain cultural heterogeneity through critical engagement. The evaluation criteria operationalize Gu's conceptual integration of practical language skills and cultural awareness—specifically measuring learners' capacity to articulate local identities while navigating global discourse frameworks.

Integration with existing standard frameworks in university context

The proposed local cultural awareness assessment model provides a comprehensive approach to evaluating EFL learners' engagement with local culture. Furthermore, the practical application of this model can be aligned with widely recognized standard frameworks, such as the Common European Framework of Reference for Languages (CEFR) and the American Council on the Teaching of Foreign Languages (ACTFL) proficiency guidelines. Although this study highlights

the blind spots of current frameworks (such as CEFR and ACTFL) in assessing local cultural expression, the new model is not intended to replace these standardized systems but to achieve synergistic enhancement through a hierarchical complementary strategy.

The CEFR offers a comprehensive set of language proficiency descriptors spanning diverse proficiency levels [56]. We propose integrating the local cultural awareness assessment model with the CEFR starting from the intermediate level, employing a descriptor transplantation approach. Specifically, this integration can be achieved by embedding local cultural indicators into the CEFR's mediational and plurilingual descriptors, as underscored in the document regarding "action-oriented scenarios" and "plurilingual repertoires" [56]. As outlined in S6 Table, this strategic embedding aligns secondary indicators with CEFR levels B1 to C2, illustrating how descriptors like "relaying specific information" (B2) and "facilitating pluricultural space" (C1) can be adapted to assess local cultural competence.

B1–B2 Intermediate Level Integration: At these levels, foundational local cultural indicators (e.g., [illegible]-1 Local History, [illegible]-4 Daily Life Experiences) can be integrated into CEFR's "Mediating Concepts" and "Reception of Information" descriptors. For instance, [illegible]-1's requirement for learners to "discuss local history events in English" aligns with CEFR B2's descriptor for "relaying specific information" (Section 3.1.4.2)[56], where learners are expected to "summarize historical contexts with cultural significance". Similarly, [illegible]-6 (Local Ethical and Legal Systems) can be grafted onto CEFR's B2 "Explaining Data" scale, as the document highlights the importance of mediating social norms through language.

C1–C2 Advanced Level Integration: At higher proficiency tiers, sophisticated cultural indicators (e.g., [illegible]-15 Local Image Promotion, [illegible]-18 Cultural Comparison) map to CEFR's "Plurilingual Mediation" and "Facilitating Pluricultural Space" domains. For example, [illegible]-18's requirement to "compare local and target cultures" directly aligns with CEFR C1's descriptor for "mediating cross-cultural dialogue" (Section 7.2.2), where learners are expected to "negotiate cultural differences in complex scenarios". Additionally, [illegible]-15's focus on "promoting local identity in English" resonates with the emphasis of CEFR on "activating plurilingual repertoires for social agency" , which can be embedded in CEFR C2's "Strategic Mediation" scale.

Cross-Level Task Design for Cultural Embedding: The document's advocacy for "scenario-based assessment" (Section 7.2.1) provides a framework for designing tasks that bridge proficiency levels [56]. For instance, in B1–B2 tasks, learners could complete a "local food culture interview" ([illegible]-4) using CEFR B2's "Oral Interaction" descriptors, which emphasize "describing daily practices with cultural relevance". And in terms of C1–C2 tasks, A "local heritage preservation campaign" ([illegible]-15) could be evaluated against CEFR C1's "Mediation of Public Discourse" criteria, requiring learners to "craft persuasive messages that integrate local values" .

Similarly, ACTFL emphasizes "intercultural communicative competence," focusing on the appropriate use of language in real social and cultural contexts [33]. We propose using the evaluation indicators established in this study to form a dual-track assessment mechanism (Fig 4). For example, a cultural awareness dimension can be added to the ACTFL Oral Proficiency Interview (OPI), allowing independent scoring of language fluency and cultural mediation while sharing weights (suggested ratio: 70% language + 30% culture). This integrated solution, which balances the emphasis on language and cultural competence, retains the reliability and validity of standardized tests and addresses the missing identity expression through the cultural competence matrix (S7 Table). It responds to Deardorff's [35] call for a "mixed assessment

method" (quantitative + qualitative) to measure cultural depth. Meanwhile, the dual-track model proposed in this study requires minimal modification to the existing ACTFL protocols but significantly enhances ecological validity in multilingual contexts.

Limitations and future directions

This study represents an initial exploration of evaluating local cultural awareness among EFL learners through a Delphi-based framework, specifically contextualized within the Chinese educational landscape. The current expert panel, consisting solely of 15 professionals from China, offers a perspective on integrating local content into EFL education. However, this composition may limit the panel's ability to fully assess the adaptability of our framework across different cultural and educational contexts. To mitigate this limitation, future research should consider expanding the panel to include experts from a variety of EFL settings, such as Latin America, Africa, and Southeast Asia, ensuring a more comprehensive validation of the framework's universal applicability.

Building on this foundation, subsequent studies could pursue two complementary paths. First, cross-cultural validation in non-Chinese settings would shed light on whether the evaluation index system requires contextual adjustments or demonstrates transferable utility. Such efforts would enrich the framework by incorporating varied perspectives on balancing local and global cultural elements in EFL curricula. Second, longitudinal investigations tracking how local cultural awareness influences students' language development and intercultural competence over time would deepen our understanding of its educational impact. Mixed-method designs combining quantitative assessments with qualitative case studies could offer nuanced insights into how learners navigate cultural hybridity in different linguistic environments.

Notably, this research establishes a critical starting point for discussing local cultural integration in EFL education, particularly in contexts where similar cultural priorities may exist. By acknowledging the potential for broader application and inviting interdisciplinary dialogue, future studies can refine the framework to accommodate the diverse needs of EFL learners worldwide, while retaining sensitivity to regional cultural identities. This approach ensures that educational interventions can both honor local voices and foster global communicative agility."

Conclusion & Future Work-Suggestion 1

- Suggest pilot testing in non-Chinese contexts.

Response

We would like to express our sincere gratitude for the insightful comments and constructive feedback provided. Your suggestion to pilot test our evaluation framework in non-Chinese contexts is invaluable and aligns perfectly with our vision for the broader applicability of our research.

The Discussion now explicitly addresses this recommendation through both limitation acknowledgment and future research directions, as detailed below:

"*....However, this composition may limit the panel's ability to fully assess the adaptability of our framework across different cultural and educational contexts. To mitigate this limitation, future research should consider expanding the panel to include experts from a variety of EFL settings, such as Latin America, Africa, and Southeast Asia, ensuring a more comprehensive validation of the framework's universal applicability."* (Page 44, Line [774-779])

"....subsequent studies could pursue two complementary paths. First, cross-cultural validation in non-Chinese settings would shed light on whether the evaluation index system requires contextual adjustments or demonstrates transferable utility. Such efforts would enrich the framework by incorporating varied perspectives on balancing local and global cultural elements in EFL curricula. " (Page 44, Line [780-784])

".....future studies can refine the framework to accommodate the diverse needs of EFL learners worldwide, while retaining sensitivity to regional cultural identities. This approach ensures that educational interventions can both honor local voices and foster global communicative agility."(Page 45, Line [793-796])

This addition directly addresses your recommendation by integrating non-Chinese context validation as a core future direction, ensuring the framework's robustness beyond the current sample. We appreciate your insight, which enhances the study's generalizability and practical impact.

Note

Please add DOIs with all references.

.

Response

Thank you so much for your meticulous review! We truly appreciate your attention to detail, as it has helped us strengthen the manuscript significantly. All references have now been updated to include DOIs and carefully formatted following the journal's Vancouver style requirements. Your guidance ensures our work meets the highest academic standards, and we're grateful for the opportunity to refine the manuscript based on your suggestions!
