# Supplementary material for: Assessing local cultural awareness in university EFL learners: A Delphi and AHP-based index framework
Source: PLoS One. 2025 Oct 8;20(10):e0332233. doi: 10.1371/journal.pone.0332233 (PMC12507305; doi:10.1371/journal.pone.0332233)
Supplement: Attachment — Submitted filename: Reviewer Report for Manuscript PONE-D-25-18530.docx [file pone.0332233.s010.docx]

**Reviewer Report for Manuscript PONE-D-25-18530**

Title: Assessing Local Cultural Awareness in University EFL Learners: A Delphi and AHP-Based Index Framework

**General Comments**

**Concerns and Suggestions for Improvement**

**1. Sample Diversity and Generalizability**

- Issue: All 15 experts were from China, potentially limiting the framework’s cross-cultural applicability.

- Suggestion: Acknowledge this limitation explicitly and recommend future validation in diverse EFL contexts (e.g., Latin America, Africa) to test the framework’s universality.

**2. Indicator Operationalization**

- Issue: While secondary indicators like *Cultural Pride* and *Local Storytelling* are well-defined, the manuscript lacks detailed examples of how these would be measured in practice (e.g., rubric criteria, sample assessment tasks).

- Suggestion: Include a supplementary table or appendix illustrating hypothetical assessment scenarios or scoring descriptors for key indicators.

**3. Theoretical Tensions**

- Issue: The study critiques existing frameworks (e.g., CEFR, ACTFL) for neglecting local identity expression but does not fully explore how its model reconciles with standardized proficiency benchmarks.

- Suggestion: Discuss potential integration strategies (e.g., augmenting CEFR’s “mediation scales” with local-culture descriptors) to enhance practical adoption.

**4. Data Availability**

- Issue: The statement “All relevant data are within the manuscript” is vague. Raw Delphi/AHP data (e.g., expert responses, pairwise comparisons) are not provided.

- Suggestion: Share anonymized expert datasets or matrices via a repository (e.g., OSF) to ensure transparency and reproducibility.

**5. Ethical Considerations**

- Clarification Needed: The ethics approval (GZPC20231127) mentions voluntary participation but does not detail how expert anonymity was maintained during Delphi rounds. Confirm whether experts were aware of each other’s identities or if responses were fully anonymized.

**Specific Concerns and Suggestions for Improvement**

**Section-by-Section Evaluation**

**1. Introduction**

Suggestions for Improvement

- Add a citation to Fan (2023) to strengthen the justification for prioritizing cultural expression:

“Recent studies (e.g., Fan, 2023) highlight the pedagogical benefits of explicit cultural feedback, supporting this study’s focus on measurable cultural awareness.”

- Clarify “Cultural Aphasia” with a brief definition.

Citations: Fan, N. (2023). Exploring the effects of automated written corrective feedback on EFL students’ writing quality: A mixed-methods study. Sage Open, 13(2), 21582440231181296.

**2. Literature Review**

Suggestions for Improvement:

- Integrate Fan (2023) to connect theory to empirical findings:

“Fan’s (2023) work on structured feedback in EFL writing aligns with Kramsch’s ‘third space,’ demonstrating how explicit corrections enhance cultural articulation.”*

Citations: Fan, N. (2023). Exploring the effects of automated written corrective feedback on EFL students’ writing quality: A mixed-methods study. Sage Open, 13(2), 21582440231181296.

- Discuss regional variations in cultural feedback preferences, citing recent studies (e.g., Asian and European learners). For in Page No. 7., the authors discussed;

“From a cognitive perspective [24], Kramsch [25] emphasized the inseparability of language and culture, noting that language learning is not only about acquiring linguistic forms but also about understanding cultural meanings, which deepens the cognitive process of language acquisition [26, 27, 28, 29].”

Here, more citations from recent studies (above 2020) are needed to be related to ‘the cognitive process of language acquisition’ in the context of feedback from Asian and European studies.

Citations:

[26] Gao, M., Qian, J., & Rasool, U. (2024). Effects of Task-Induced Involvement and Time on Task on Incidental L2 Vocabulary Acquisition. *SAGE Open*, 14(2). <https://doi.org/10.1177/21582440241249340>

[27] Rasool, U., Qian, J., & Aslam, M. Z. (2024). Understanding the Significance of EFL Students’ Perceptions and Preferences of Written Corrective Feedback. *SAGE Open*, 14(2). <https://doi.org/10.1177/21582440241256562>

[28] Winstone, N., Boud, D., Dawson, P., & Heron, M. (2022). From feedback-as-information to feedback-as-process: a linguistic analysis of the feedback literature. *Assessment & Evaluation in Higher Education*, 47(2), 213-230. <https://doi.org/10.1080/02602938.2021.1902467>

[29] Zhai, N., & Ma, X. (2022). Automated writing evaluation (AWE) feedback: A systematic investigation of college students’ acceptance. *Computer Assisted Language Learning*, 35(9), 2817-2842. <https://doi.org/10.1080/09588221.2021.1897019>

**3. Methods**

Suggestions for Improvement:

- Reference Fan (2023) to support the AHP weighting approach:

“The 45.72% weight for Local Expression mirrors findings by Fan (2023), where action-oriented tasks yielded higher cultural retention.”

- Justify the 15-expert panel size with citations (e.g., Hsu & Sandford, 2007).

**4. Results**

Suggestions for Improvement:

- Compare results to Fan (2023) for cross-validation:

“The preference for direct WCF aligns with Fan’s (2023) findings on error correction efficacy in Asian EFL contexts.”

- Visualize weight differences between dimensions (e.g., bar chart).

**5. Discussion**

Suggestions for Improvement:

- Use specific instead of generalized implications

6. Conclusion & Future Work

Suggestions for Improvement:

- Suggest pilot testing in non-Chinese contexts.

Note: Please add DOIs with all references
