# Supplementary material for: Assessing local cultural awareness in university EFL learners: A Delphi and AHP-based index framework
Source: PLoS One. 2025 Oct 8;20(10):e0332233. doi: 10.1371/journal.pone.0332233 (PMC12507305; doi:10.1371/journal.pone.0332233)
Supplement: Attachment — Submitted filename: Responses to Reviewer 2.docx [file pone.0332233.s011.docx]

# Author's responses to Reviewer 2

CONTENT

[Author's responses to Reviewer 2	1](#_Toc29971)

[General Comments	2](#_Toc10448)

[Comment 1	2](#_Toc27370)

[Comment 2	3](#_Toc15640)

[Comment 3	6](#_Toc11212)

[Comment 4	9](#_Toc25578)

[Comment 5	10](#_Toc21262)

[Specific Concerns	11](#_Toc4761)

[Introduction-Suggestion1	11](#_Toc29383)

[Introduction-Suggestion2	12](#_Toc25792)

[Literature Review-Suggestion1	13](#_Toc19066)

[Literature Review-Suggestion2	15](#_Toc19656)

[Methods-Suggestion 1	17](#_Toc14365)

[Methods-Suggestion 2	18](#_Toc14677)

[Results-Suggestion 1	19](#_Toc24120)

[Results-Suggestion 2	21](#_Toc24411)

[Discussion-Suggestion	23](#_Toc4154)

[Conclusion & Future Work-Suggestion 1	32](#_Toc5556)

[Note	34](#_Toc24116)

## General Comments

### Comment 1

**Sample Diversity and Generalizability**

**- Issue: All 15 experts were from China, potentially limiting the framework's cross-cultural applicability.**

**- Suggestion: Acknowledge this limitation explicitly and recommend future validation in diverse EFL contexts (e.g., Latin America, Africa) to test the framework's universality.**

**Response**:

Thank you for your insightful feedback and for pointing out the potential limitation regarding the cross-cultural applicability of our framework. We appreciate your suggestion to acknowledge this limitation explicitly and recommend future validation in diverse EFL contexts.

Here is the revised section of our manuscript:

***Limitations and Future Directions (*** *Page 42 Line 772-779 ):*

*“... The current expert panel, consisting solely of 15 professionals from China, offers a perspective on integrating local content into EFL education. However, this composition may limit the panel's ability to fully assess the adaptability of our framework across different cultural and educational contexts. To mitigate this limitation, future research should consider expanding the panel to include experts from a variety of EFL settings, such as Latin America, Africa, and Southeast Asia, ensuring a more comprehensive validation of the framework's universal applicability."*

We sincerely appreciate the reviewers' insightful comments, which have significantly improved our manuscript. We hope the revised version meets the journal's standards.

### Comment 2

**Indicator Operationalization**

**-Issue: While secondary indicators like Cultural Pride and Local Storytelling are well-defined, the manuscript lacks detailed examples of how these would be measured in practice (e.g., rubric criteria, sample assessment tasks).**

**-Suggestion: Include a supplementary table or appendix illustrating hypothetical assessment scenarios or scoring descriptors for key indicators."**

**Response**:

We sincerely thank you for this insightful and professional suggestion. We fully agree that concrete operationalization examples are essential to demonstrate the practical applicability of the proposed framework. To address this point, we have taken the following actions:

1.Added a Comprehensive Operationalization Table (S5 Table).

A new supplementary table (S5 Table: Operationalization Framework for Local Cultural Awareness Assessment) has been included in the revised manuscript (provided as Supplementary Material). This table provides illustrative examples of: (1) Practical assessment scenarios aligned with the 18 secondary indicators (e.g., a 'Pride Portfolio' task for Cultural Pride [Ⅱ-10], a 'Local Culture Podcast' project for Local Storytelling [Ⅱ-14]). (2) Hypothetical evaluation criteria with indicative weightings (e.g., 'Affective Authenticity' as a sample dimension at 40%, 'Linguistic Accuracy' at 30%) to demonstrate operational feasibility.

2.Expanded Discussion with S5 Table Integration.

- In the revised Discussion section (Page 34-35, Lines [577-585]), For Local expression and application dimension, we explicitly reference S5 Table to demonstrate how key indicators translate into practice:

*"As demonstrated in S5 Table, in teaching practice, this study suggests replacing 45% of the traditional cultural teaching modules in EFL programs with Production-oriented tasks. For instance, students could be tasked with documenting local heritage sites in English—such as ancient bridges bearing donation inscriptions—and composing analytical narratives that systematically unpack the cultural connotations embedded in these physical spaces. Through their writing, learners should illuminate how these inscriptions exemplify traditional values of fraternal cooperation and contractual ethics, bridging historical artifacts with contemporary understanding."*

- For Local Storytelling (Ⅱ-14), we describe the podcast task and its assessment criteria (S5 Table), linking it to Byram's ICC model ( Page 35, Lines [589-595]).

*"Under the application dimension, the secondary indicator of Use of English for Local Storytelling holds one of the highest global weights (W = 0.1366), underscoring its central role in cultivating intercultural competence. In university EFL courses, this indicator can be operationalized through collaborative projects: advanced learners might document the entrepreneurial histories of local time-honored brands, creating podcasts that integrate interviews with entrepreneurs and cultural analyses (Ⅱ-14 in S5 Table). ”*

- For Promotion and Preservation of Local Image (Ⅱ-15), we explain how the indicator is operationalized via international communication scenario simulations to assess students' competence in clarifying cultural misconceptions (Page 36, Lines [612-620]).

*"Additionally, the secondary indicator of Promotion and Preservation of Local Image (global weight= 0.1366, M = 5.00) has received high endorsement from experts, highlighting the practical value of constructing cultural discourse power. As operationalized in S5 Table, this sub-indicator (Ⅱ-15) provides measurable criteria for assessing students' international communication competence in university EFL contexts. By designing international communication scenario simulations, students, under the guidance of professors, can clarify and refute misconceptions about local culture disseminated by foreign media. These practices operationalize the scholars' view that EFL teaching needs to enhance the application of cultural knowledge [3,12]."*

- For Cultural Pride (Ⅱ-10), we explain how the "Pride Portfolio" task evaluates emotional authenticity through artifact annotations ( Page 37, Lines [630-635]).

*"In university EFL assessment, cultural pride is evaluated through tasks such as the "Pride Portfolio" (II-10 in S5 Table). For example, anthropology students' cultural awareness is measured by their ability to compile cultural artifacts with precise English annotations (e.g., distinguishing "butterfly patterns symbolizing ancestral blessings" from generic "insect decorations"), demonstrating how linguistic accuracy reflects cultural symbol interpretation in evaluation criteria."*

- For Cross-cultural Understanding Interest (Ⅱ-11), the "Cultural Exchange Archive" task assesses students' ability to create bilingual cultural packages, evaluating their capacity to document local traditions in English and critically analyze foreign customs from exchanged materials (Page 37, Lines [636-640]

*Similarly, cross-cultural openness is assessed via the "Cultural Exchange Archive" (II-11 in S5 Table), where evaluation focuses on students' capacity to create bilingual packages—documenting local traditions like Dragon Boat Races in English while critically analyzing foreign customs (e.g., Christmas) from exchanged materials.*

- For Cultural Embeddedness in Assessment (Ⅱ-2/Ⅱ-4/Ⅱ-5), we illustrate how tasks like "a day in the life" documentation and artifact transformation operationalize symbolic mediation competence through criteria aligned with Alakrash's cognitive authenticity and Kramsch's symbolic competence frameworks (Page 39, Lines [664-677]).

*"This consensus confirms the critical role of culturally-embedded assessment tasks in measuring EFL learners' ability to mediate local knowledge through English, as exemplified by "a day in the life" documentation (II-4) and local figure narration (II-5) tasks in S5 Table. Aligned with Alakrash's [2] cognitive authenticity principle, these evaluation criteria demonstrate how contextually-grounded content enhances linguistic encoding and identity positioning in assessment outcomes. The transformation task for local cultural artifacts (II-2 in S5 Table), for instance, evaluates students' symbolic mediation competence through parameters including: (1) cultural authenticity of adapted content, (2) linguistic accuracy in performance scripts, and (3) effective conveyance of embodied cultural meanings. Such multidimensional assessment operationalizes Kramsch's [49] "symbolic competence" framework, where successful performance necessitates transcending lexical equivalence to achieve semiotic resonance between form and cultural substance."*

Based on your valuable insight, we think this revision will strengthen the manuscript by: (1) providing actionable guidance for educators to implement the framework, (2) validating the ecological validity of indicators through real-world tasks, and (3) bridging the gap between theoretical constructs (e.g., ICC, symbolic competence) and classroom practice.

Your invaluable feedback has been instrumental in strengthening the rigor and applicability of our study, and we sincerely appreciate the opportunity to incorporate such thoughtful suggestions.

### Comment 3

**3. Theoretical Tensions**

**- Issue: The study critiques existing frameworks (e.g., CEFR, ACTFL) for neglecting local identity expression but does not fully explore how its model reconciles with standardized proficiency benchmarks.**

**- Suggestion: Discuss potential integration strategies (e.g., augmenting CEFR's "mediation scales" with local-culture descriptors) to enhance practical adoption.**

**Response**:

First and foremost, we extend our sincere gratitude for your insightful feedback, which has significantly enhanced the rigor and practical relevance of our study. Your suggestion to explore the reconciliation between our model and standardized proficiency benchmarks is both timely and invaluable, as it addresses a critical gap in the original manuscript.

In response to your feedback, we have substantially revised the discussion section to elaborate on integration strategies between our local cultural awareness model and established frameworks (e.g., CEFR, ACTFL). The key modifications include:

1. Systematic Alignment with CEFR's Mediation Scales: We now explicitly demonstrate how our 18 evaluation indicators integrate with CEFR's "mediation competence" descriptors (detailed in S6 Table).

2. ACTFL-OPI Integration for Dynamic Assessment: A new dual-track assessment mechanism (depicted in Fig 4,detailed in S7 Table) is proposed, which adds a cultural awareness dimension to ACTFL's Oral Proficiency Interview (OPI).

We think these modifications not only address the critique of existing frameworks but also provide tangible tools for educators to implement our model within standardized systems, enhancing adoption; as well as strengthen the theoretical foundation by aligning with Deardorff's mixed-method assessment paradigm (2006).

Once again, we appreciate your guidance in refining our manuscript. The revisions now better balance theoretical critique with practical solutions, ensuring the study's relevance to both academic and applied contexts. We welcome any further suggestions to optimize these integrative strategies.

**The details can be found in Page[ 40-43], Lines [704-768]:**

"**Integration with existing standard frameworks in university context**

The proposed local cultural awareness assessment model provides a comprehensive approach to evaluating EFL learners' engagement with local culture. Furthermore, the practical application of this model can be aligned with widely recognized standard frameworks, such as the Common European Framework of Reference for Languages (CEFR) and the American Council on the Teaching of Foreign Languages (ACTFL) proficiency guidelines. Although this study highlights the blind spots of current frameworks (such as CEFR and ACTFL) in assessing local cultural expression, the new model is not intended to replace these standardized systems but to achieve synergistic enhancement through a hierarchical complementary strategy.

The CEFR offers a comprehensive set of language proficiency descriptors spanning diverse proficiency levels [56]. We propose integrating the local cultural awareness assessment model with the CEFR starting from the intermediate level, employing a descriptor transplantation approach. Specifically, this integration can be achieved by embedding local cultural indicators into the CEFR's mediational and plurilingual descriptors, as underscored in the document regarding "action-oriented scenarios" and "plurilingual repertoires" [56]. As outlined in S6 Table, this strategic embedding aligns secondary indicators with CEFR levels B1 to C2, illustrating how descriptors like "relaying specific information" (B2) and "facilitating pluricultural space" (C1) can be adapted to assess local cultural competence.

B1–B2 Intermediate Level Integration: At these levels, foundational local cultural indicators (e.g., Ⅱ-1 Local History, Ⅱ-4 Daily Life Experiences) can be integrated into CEFR's "Mediating Concepts" and "Reception of Information" descriptors. For instance, Ⅱ-1's requirement for learners to "discuss local history events in English" aligns with CEFR B2's descriptor for "relaying specific information" (Section 3.1.4.2)[56], where learners are expected to "summarize historical contexts with cultural significance". Similarly, Ⅱ-6 (Local Ethical and Legal Systems) can be grafted onto CEFR's B2 "Explaining Data" scale, as the document highlights the importance of mediating social norms through language.

C1–C2 Advanced Level Integration: At higher proficiency tiers, sophisticated cultural indicators (e.g., Ⅱ-15 Local Image Promotion, Ⅱ-18 Cultural Comparison) map to CEFR's "Plurilingual Mediation" and "Facilitating Pluricultural Space" domains. For example, Ⅱ-18's requirement to "compare local and target cultures" directly aligns with CEFR C1's descriptor for "mediating cross-cultural dialogue" (Section 7.2.2), where learners are expected to "negotiate cultural differences in complex scenarios". Additionally, Ⅱ-15's focus on "promoting local identity in English" resonates with the emphasis of CEFR on "activating plurilingual repertoires for social agency" , which can be embedded in CEFR C2's "Strategic Mediation" scale.

Cross-Level Task Design for Cultural Embedding: The document's advocacy for "scenario-based assessment" (Section 7.2.1) provides a framework for designing tasks that bridge proficiency levels [56]. For instance, in B1–B2 tasks, learners could complete a "local food culture interview" (Ⅱ-4) using CEFR B2's "Oral Interaction" descriptors, which emphasize "describing daily practices with cultural relevance". And in terms of C1–C2 tasks, A "local heritage preservation campaign" (Ⅱ-15) could be evaluated against CEFR C1's "Mediation of Public Discourse" criteria, requiring learners to "craft persuasive messages that integrate local values" .

Similarly, ACTFL emphasizes "intercultural communicative competence," focusing on the appropriate use of language in real social and cultural contexts [33]. We propose using the evaluation indicators established in this study to form a dual-track assessment mechanism (Fig 4). For example, a cultural awareness dimension can be added to the ACTFL Oral Proficiency Interview (OPI), allowing independent scoring of language fluency and cultural mediation while sharing weights (suggested ratio: 70% language + 30% culture). This integrated solution, which balances the emphasis on language and cultural competence, retains the reliability and validity of standardized tests and addresses the missing identity expression through the cultural competence matrix (S7 Table). It responds to Deardorff's [35] call for a "mixed assessment method" (quantitative + qualitative) to measure cultural depth. Meanwhile, the dual-track model proposed in this study requires minimal modification to the existing ACTFL protocols but significantly enhances ecological validity in multilingual contexts."

Fig 4. Schematic of the dual - track assessment process for the ACTFL OPI task

### Comment 4

**4. Data Availability**

**- Issue: The statement "All relevant data are within the manuscript" is vague. Raw Delphi/AHP data (e.g., expert responses, pairwise comparisons) are not provided.**

**- Suggestion: Share anonymized expert datasets or matrices via a repository (e.g., OSF) to ensure transparency and reproducibility.**

**Response**

We sincerely appreciate your kind reminder on data transparency, which is critical for enhancing the reproducibility of our study. To address this concern, we have fully complied with the suggestion by anonymizing and depositing all raw Delphi/AHP datasets in a public repository.

The dataset has been uploaded to Figshare ( link: https://figshare.com/s/d40943028c0d35d84f8f ) with DOI: 10.6084/m9.figshare.29115743 under a CC BY 4.0 license, allowing open access and reuse with proper attribution. The repository includes: (1) Anonymized Excel files containing scored results from two rounds of Delphi surveys with 15 experts, presented as Table 1 (Round 1) and Table 2 (Round 2); (2) AHP pairwise comparison matrices (in .rar format) from 15 experts for first-level and second-level evaluation indicatorsAll expert identities have been removed from the dataset, and no personally identifiable information is included, ensuring compliance with ethical guidelines.

We believe this approach fully addresses the editor's and reviewers' concern about transparency and provides robust support for the reproducibility of our findings.

### Comment 5

**Ethical Considerations**

**- Clarification Needed: The ethics approval (GZPC20231127) mentions voluntary participation but does not detail how expert anonymity was maintained during Delphi rounds. Confirm whether experts were aware of each other's identities or if responses were fully anonymized.**

**Response**

Thank you for your meticulous attention to the ethical considerations. In response to the concern regarding the maintenance of expert anonymity during the Delphi rounds, we have made an addition to the manuscript. In the relevant section (Methods: Participants), we've now clearly stated that the survey materials provided to experts explicitly guaranteed strict confidentiality.

**The details can be found inPage 11, Lines [222-233]**
"*In accordance with the Delphi method guidelines [41] that suggest a sample size of 15-20 experts for consensus - driven studies, 15 EFL educators (Table 1) were invited to participate in the study and formed the expert panel. The recruitment period for this study spanned from November 30, 2023, to March 1, 2024. These 15 participants were recruited from a variety of academic institutions across China, which guaranteed geographical and institutional diversity. All participants provided written informed consent, and the study was approved by the Institutional Review Board of GPC College (GZPC20231127). To ensure the maintenance of expert anonymity during the Delphi rounds, the survey materials explicitly guaranteed strict confidentiality. Experts were not made aware of each other's identities, and all responses were collected and handled to ensure full anonymization throughout the process."*

Thank you for your attention to ethical details. Your guidance ensures our work adheres to rigorous academic and ethical standards, and we're grateful for the opportunity to improve it.

## Specific Concerns

### Introduction-Suggestion1

**Clarify "Cultural Aphasia" with a brief definition.**

**Response**

Thank you for your valuable suggestion regarding the clarification of "Cultural Aphasia" in our introduction. We agree that providing a clear and concise definition is essential for the reader's understanding of this concept and its relevance to our study. In response to your comment, we have revised the introduction to include a brief yet precise definition of "Cultural Aphasia.".

Here are the revised paragraphs. The details can be found in Page 2, Lines [31-41]

*"In today's globalized world, English proficiency is regarded as an essential skill for cross-border communication. English as a Foreign Language (EFL) programs are increasingly designed not only to enhance learners' language skills but also to develop their ability to engage with the global community.* Learners face "Cultural Aphasia," a term proposed by Cong from Nanjing University in China. It describes the situation where learners, despite having a high level of language proficiency, find it challenging to convey the rich and unique aspects of their native culture [1]*. This linguistic-cultural disjuncture has catalyzed reforms in EFL curricula, where there is growing recognition of the importance of integrating localized content into language learning to make it more relevant and meaningful to learners' lived experiences."*

We believe that these changes address your concern and improve the overall readability and clarity of our introduction. Thank you again for your insightful feedback.

### Introduction-Suggestion2

**"Add a citation to Fan (2023) to strengthen the justification for prioritizing cultural expression."**

**Response**

Thank you for your suggestion to add a citation to Fan (2023) in the introduction to strengthen the justification for prioritizing cultural expression. We appreciate your input and have carefully considered its relevance to our study.

After reviewing the content of Fan (2023) and its focus on the effects of automated written corrective feedback (AWCF) on EFL students' writing quality, we concluded that while Fan's work is valuable. Given that our introduction aims to lay the groundwork for our research by highlighting the importance of local cultural expression, we feel that incorporating Fan (2023) at this stage might introduce a tangential focus that could slightly dilute the primary message of our introduction.

To ensure that our introduction remains focused and relevant to our research objectives, we have decided to integrate Fan (2023) into both the Literature Review and Discussion sections. In the Literature Review section, we will connect Fan's findings on writing feedback to our broader discussion of empirical findings related to EFL learning. This approach allows us to contextualize Fan's work within the existing body of literature and highlight its relevance to our study without detracting from the primary focus of our introduction. Additionally, in the Discussion section, we will further explore how Fan's insights on feedback mechanisms can inform our understanding of the role of cultural feedback in EFL learning, providing a more comprehensive integration of theoretical and empirical perspectives.

We believe that the integration of Fan (2023) into both the Literature Review and Discussion sections effectively connects theory to empirical findings and strengthens the overall coherence of our research. Thank you again for your thoughtful feedback. We look forward to your understanding.

### Literature Review-Suggestion1

Suggestions for Improvement:

**- Integrate Fan (2023) to connect theory to empirical findings.**

**Response**

Thank you for your insightful suggestion. We agree that Fan's work provides valuable empirical evidence on the role of structured feedback in EFL writing, which aligns well with Kramsch's concept of the "third space." Fan's study not only highlights the importance of feedback mechanisms in enhancing cultural articulation but also offers practical insights into how automated writing evaluation systems can support EFL learners in developing their writing skills. This integration enriches our discussion by bridging the gap between theoretical frameworks and real-world applications in EFL education.

In response to your comment, we have incorporated Fan (2023) into the Literature Review section, specifically in the part discussing the theoretical framework of local cultural awareness.

Here is the revised paragraph:(For your convenience in reading and reviewing, we have temporarily used a non-numeric citation format in this letter. However, in the final revised manuscript, all citations have been adjusted to the required numeric order format.)

The details can be found inPage [4-5], Lines [87-94]

*"The theoretical framework of local cultural awareness is also rooted in other disciplines. Under the model of cultural ecology, Kramsch [11] put forward the concept of "third space", believing that the EFL classroom should be a place for dynamic negotiation between local culture and global culture. Fan (2023) provides empirical evidence that aligns with Kramsch's perspective, demonstrating how structured feedback in EFL writing can enhance cultural articulation. This suggests that feedback mechanisms can play a crucial role in helping learners develop cultural awareness and effectively express their local identities in English."*

We are truly grateful for your insightful comment, which has enabled us to enhance the theoretical depth of our manuscript by integrating relevant research. Your careful review and guidance are highly appreciated, and we hope the revised content meets your expectations.

### Literature Review-Suggestion2

Suggestions for Improvement:

**-- Discuss regional variations in cultural feedback preferences, citing recent studies (e.g., Asian and European learners).**

**"From a cognitive perspective [24], Kramsch [25] emphasized the inseparability of language and culture, noting that language learning is not only about acquiring linguistic forms but also about understanding cultural meanings, which deepens the cognitive process of language acquisition [26, 27, 28, 29]."**

**Here, more citations from recent studies (above 2020) are needed to be related to 'the cognitive process of language acquisition' in the context of feedback from Asian and European studies..**

**Response**

Thank you for your valuable suggestions regarding the need to incorporate more recent studies related to the cognitive process of language acquisition, particularly in the context of feedback from Asian and European studies. In response to your feedback, we have carefully revised the literature review section to include additional references that strengthen the discussion on this topic.

Specifically, we have added recent studies by Winstone et al. (2022), Zhai & Ma (2021), and Gao et al. (2024) to the "Benefits of Local Content Integration in EFL Instruction" section. These studies provide insights into how feedback mechanisms and task design can enhance learners' cognitive engagement with cultural content, thereby enriching their understanding and application of cultural knowledge in language learning.

Here is the revised version. (For your convenience in reading and reviewing, we have temporarily used a non-numeric citation format in this letter. However, in the final revised manuscript, all citations have been adjusted to the required numeric order format.)

The details can be found in Page [7-8], Lines [144-156]

*"From a cognitive perspective, Kramsch [26] emphasized the inseparability of language and culture, noting that language learning is not only about acquiring linguistic forms but also about understanding cultural meanings, which deepens the cognitive process of language acquisition. This aligns with findings that the cognitive feedback provided by digital tools can significantly impact learners' cognitive development and application of cultural knowledge in language learning* **(Zhai & Ma, 2021). Gao et al. (2024)** *further demonstrate that task-induced involvement and time on task can significantly influence vocabulary acquisition in L2 learners, suggesting that culturally-relevant tasks enhance cognitive engagement and cultural knowledge integration. Recent research reinforces this idea by highlighting how feedback mechanisms can enhance learners' cognitive engagement with cultural content, thereby enriching their understanding and application of cultural knowledge in language learning contexts (Winstone et al., 2022).*

Sincere thanks for your thoughtful suggestion. It has been a pleasure incorporating your feedback to enrich our literature discussion. We look forward to your continued guidance as we finalize the manuscript.

### Methods-Suggestion 1

**- Reference Fan (2023) to support the AHP weighting approach.**

**Response**

We sincerely thank the reviewer for this constructive suggestion. In direct response, we have strengthened the theoretical grounding of our AHP weight allocation by incorporating empirical support from Fan (2023).

The details can be found inPage 34, Lines [565-572]

"*The significantly higher weight assigned to Local Expression and Application (weight = 0.4527, the highest among the three dimensions) not only aligns with Byram's [5] intercultural communicative competence (ICC) model, which prioritizes knowledge-to-practice transformation, but also finds empirical support in Fan's [12] study. In Fan's research, action-oriented tasks yielded higher cultural retention, and this emphasis on practical alignment is further validated by the study's finding that direct written corrective feedback (WCF) enhances students' cultural expression in EFL writing—findings that complement Byram's focus on operational competence."*

We chose to embed this reference in the Discussion section because it naturally complements the existing theoretical framework of Byram's ICC model, and Fan's (2023) findings specifically address the pedagogical implications of prioritizing practical application, which is a core focus of our Discussion.

We are grateful for this suggestion which has enhanced the study's theoretical-empirical integration.

### Methods-Suggestion 2

**Justify the 15-expert panel size with citations (e.g., Hsu & Sandford, 2007).**

**Response**

Thank you for your careful review. We appreciate the opportunity to clarify the justification for the expert panel size. In the original manuscript, the sample size rationale is addressed in the Methods section (Page 11, line [222-224]). In the revised version, we cited Delphi method guidelines [41] specifying that 15–20 experts are appropriate for consensus-driven studies. The citation [41] corresponds to Hsu & Sandford (2007), which directly supports this range.

*"In accordance with the Delphi method guidelines [41] that suggest a sample size of 15-20 experts for consensus - driven studies, 15 EFL educators (Table 1) were invited to participate in the study and formed the expert panel. “*

**Reference**

41. Hsu CC, Sandford BA. The Delphi Technique: Making Sense of Consensus. Practical Assessment, Research, and Evaluation. 2007;12(1):10. https://doi.org/10.7275/pdz9-th90

We apologize for any ambiguity caused by the previous citation format and trust this revision addresses your concern effectively.

### Results-Suggestion 1

**Suggestions for Improvement:**

**- Compare results to Fan (2023) for cross-validation.**

**Response**

Thank you for your suggestion to compare our results to Fan (2023) for cross-validation. We have carefully considered your advice and have incorporated a comparison with Fan's findings into our Results section to provide additional validation and context for our study.

In our revised Results section, we have highlighted the alignment between our findings and those of Fan (2023). Specifically, we have noted that the priority of the dimension of Local Expression and Application, which received the highest weight in our study, is consistent with Fan's (2023) findings on the efficacy of direct written corrective feedback (WCF) in enhancing students' ability to articulate and apply cultural knowledge in EFL contexts. Fan's study demonstrated that direct WCF, which explicitly points out errors and provides corrections, is particularly beneficial for students in Asian EFL settings, facilitating their transition from theoretical understanding to practical application. This alignment supports our recommendation to replace 45% of traditional cultural teaching modules in EFL programs with production-driven tasks, as it underscores the importance of practical application in enhancing cultural expression.

Furthermore, we have included a discussion on how our sub-indicators, such as "English Local Story-telling" and "Promotion and Protection of Local Image," align with Fan's emphasis on the practical value of cultural discourse power construction. These practices not only align with Porto's (2020) initiative to strengthen applied cultural knowledge in EFL teaching but also find empirical support in Fan's (2023) study, which highlights the effectiveness of direct WCF in enhancing cultural articulation.

****Revised Section (Page 34, line [565-572]):****

"The significantly higher weight assigned to *Local Expression and Application* (weight = 0.4527, the highest among the three dimensions) not only aligns with Byram's [5] intercultural communicative competence (ICC) model, which prioritizes knowledge-to-practice transformation, but also finds empirical support in Fan's [12] study. In Fan's research, action-oriented tasks yielded higher cultural retention, and this emphasis on practical alignment is further validated by the study's finding that direct written corrective feedback (WCF) enhances students' cultural expression in EFL writing—findings that complement Byram's focus on operational competence."

We believe that these comparisons provide a robust cross-validation of our results and strengthen the overall coherence and validity of our research. Thank you again for your constructive feedback.

### Results-Suggestion 2

**- Visualize weight differences between dimensions (e.g., bar chart).**

**Response**

Thank you for the suggestion. We have addressed this by incorporating **Fig 2** and **Fig 3** to visualize the weight differences between primary and secondary dimensions, respectively. The bar charts clearly illustrate the comparative weights of each indicator, providing a visual representation of the data for enhanced clarity.

**Fig 2. Weights of primary indicators for local cultural awareness assessment**

**Fig 3. Secondary indicator weights in cultural awareness assessment**

### Discussion-Suggestion

**- Use specific instead of generalized implications.**

**Response**

Thank you for your insightful suggestion to replace generalized implications with specific ones. We have made detailed revisions to the discussed sections in response to your feedback, transforming abstract concepts into concrete applications, empirical connections, and actionable teaching scenarios. Please allow us to place the descriptions of the relevant improvements in the table below for your convenience.

**Comparison of old and new versions of Discussion**

| **Comparison Dimension** | **Content in Old Discussion** | **Content in New Discussion** | **Description of Modification Points** |
| --- | --- | --- | --- |
| **Structural Framework** | No independent chapter on integration with standard frameworks | Added the chapter "Integration with Existing Standard Frameworks in University Context" (P40-P45, line[704-796]) | Changed from general mention of frameworks to systematic hierarchical integration, clarifying the docking strategies with CEFR and ACTFL |
| **Theoretical Application** | Mentioned Byram's model and Kramsch's theory without linking to indicator design | Specify Byram's ICC model into a "knowledge-action transformation mechanism", combined with Fan [12]'s empirical research (P34, line [565-572]) | Shifted from theoretical citation to a closed loop of "theory-empirical evidence-teaching", adding specific teaching task designs |
| **Indicator Weight Analysis** | Only stated AHP weight results (e.g., Local Expression weight 0.4527) | Linked weights to the proportion of teaching modules, proposing to "replace 45% of traditional cultural teaching modules with production-oriented tasks"(P34, line [577-583]) . | Transformed weight data from mere statistical results into executable curriculum design basis |
| **Teaching Cases** | Lacked specific teaching scenarios, with skills like "storytelling" only generally discussed | Added specific tasks such as "English documentation of local heritage" and "podcasts on time-honored local brands"(P34-P35, line [579-593]) , and attached evaluation criteria (S7 Table) | Shifted from abstract skill descriptions to operable teaching cases, increasing quantitative evaluation dimensions |
| **Emotional Dimension Assessment** | Only mentioned "cultural pride" and "openness" without assessment tools | Designed assessment tools like "Pride Portfolio" and "Cultural Exchange Archive", with tasks for beginner/advanced levels (P37-P38, line [621-649]) | Shifted from conceptual exposition to a scenario-based and hierarchical assessment system, with specific operation examples attached |
| **Cognitive Dimension Indicator Screening** | Only explained the deletion of abstract indicators (such as the local political system) without attaching theoretical basis | Cited Coyle [54]'s CLIL theory to explain the basis for deletion, and retained indicators like "Daily Life Experiences" (P39-P40, line [650-703]) | Shifted from result statement to a complete argument of "theoretical basis + expert opinions + data support" |
| **Docking with CEFR** | Only mentioned the CEFR framework without explaining the hierarchical correspondence | Established S6 Table to explain the hierarchical correspondence. | Shifted from framework compatibility discussion to specific level docking, with a comparison table of descriptors attached |
| **Integration with ACTFL** | Did not involve the ACTFL framework | Added an ACTFL dual-track assessment mechanism and S7 Table. (P43, line [753-766]) | Shifted from single-framework docking to multi-standard integration, increasing innovation in the assessment mechanism |
| **Data Visualization** | No visualized Figures | incorporating Fig 2 and Fig 3 to visualize the weight differences between primary and secondary dimensions, respectively. | Shifted from text description to chart assistance, strengthening the visual correlation between data and teaching practice |
| **Handling of Expert Divergence** | Only mentioned CV values in the Delphi method without explaining the handling approach | Cited Alakrash [2]'s "cognitive authenticity" principle to explain the specific criteria for retaining/deleting indicators (P39, line [664-670]) | Shifted from data presentation to a logic of "theory + data" for handling expert opinions, with a screening flowchart attached |
| **Literature Citation** | Cited Byram [5], Kramsch [25], etc., without combining the latest research | Added recent five-year literatures such as Fan [12], Gu [22], and Porto [3] to strengthen empirical support. | Shifted from classic theoretical citation to a combination of "classic + frontier", enhancing the timeliness of the research |

To sum up, this major revision is now anchored in:

1. Empirical data (AHP weights, CV values),
2. Operational tasks (curriculum design, assessment rubrics), or
3. Theoretically driven decisions (framework mappings).

We are grateful for this suggestion which has significantly strengthened the study's applicability. Thank you so much! This ensures the Discussion section now provides tangible insights for both researchers and educators, addressing your suggestion to prioritize specific over generalized implications.

Here is the whole Revised Discussion (Page 33-45, Line [ 238-796] )

“**Discussion**

This study constructed an evaluation index system for local cultural awareness in EFL learning through the Delphi method and Analytic Hierarchy Process (AHP), addressing the underrepresentation of learners' native cultural identity. The following discussion will interpret the findings from educational and theoretical perspectives and link the revision or retention of indicators to the practical significance in university EFL teaching.

The study confirmed that local cultural awareness in EFL learning can be systematically evaluated through three dimensions (Fig 2): Local Cognition and Understanding, Local Affective Attitudes, and Local Expression and Application. The AHP-derived weights revealed that Local Expression and Application (W=0.4527) holds the highest priority, followed by Local Affective Attitudes (W=0.3301) and Local Cognition and Understanding (0.2172). Among secondary indicators (Fig 3), Use of English for Local Storytelling, Promotion and Preservation of Local Image emerged as the most critical skills, while Cultural Pride and Critical Openness in Cross-Cultural Engagement dominated the affective dimension. Meanwhile, Local History, Local Cultural Practices, Art, and Literature, Local Development Achievements, and Daily Life Experiences exhibit relatively high weights under the dimension of Cognition, revealing the multifaceted emphasis on local cognition and understanding in English learning.

**Local expression and application dimension**

The significantly higher weight assigned to Local Expression and Application (weight = 0.4527, the highest among the three dimensions) not only aligns with Byram's [5] intercultural communicative competence (ICC) model, which prioritizes knowledge-to-practice transformation, but also finds empirical support in Fan's [12] study. In Fan's research, action-oriented tasks yielded higher cultural retention, and this emphasis on practical alignment is further validated by the study's finding that direct written corrective feedback (WCF) enhances students' cultural expression in EFL writing—findings that complement Byram's focus on operational competence.

This study extends prior research by developing an evaluation index system for EFL learners' local cultural awareness and integrating it into project-based output tasks. This approach operationalizes Byram's Intercultural Communicative Competence (ICC) principles through context-specific language applications—a critical yet underexplored dimension in existing literature. As demonstrated in S5 Table, in teaching practice, this study suggests replacing 45% of the traditional cultural teaching modules in EFL programs with Production-oriented tasks. For instance, students could be tasked with documenting local heritage sites in English—such as ancient bridges bearing donation inscriptions—and composing analytical narratives that systematically unpack the cultural connotations embedded in these physical spaces. Through their writing, learners should illuminate how these inscriptions exemplify traditional values of fraternal cooperation and contractual ethics, bridging historical artifacts with contemporary understanding. Through the knowledge-action transformation mechanism, this design directly responds to the highest AHP-assigned weight of this dimension, translating the ICC model from a theoretical framework into teaching practice.

Under the application dimension, the secondary indicator of Use of English for Local Storytelling holds one of the highest global weights (W = 0.1366), underscoring its central role in cultivating intercultural competence. In university EFL courses, this indicator can be operationalized through collaborative projects: advanced learners might document the entrepreneurial histories of local time-honored brands, creating podcasts that integrate interviews with entrepreneurs and cultural analyses (Ⅱ-14 in S5 Table). These narratives can be showcased at academic conferences, fostering both language proficiency and cultural advocacy. The narrative-driven approach diverges from earlier studies that prioritized target-culture immersion [11],[5], as well as recent work that framed narratives primarily as tools for grammatical accuracy [51]. In contrast to Fan's [12] focus on refining cultural expression via corrective feedback, our design foregrounds proactive cultural production. This approach not only enhances their language skills but also fosters a deeper engagement with cultural content and critical thinking about cultural comparisons, aligning with the principles of higher-order thinking (HOT) and interdisciplinary learning emphasized in contemporary EFL education [15, 16]. The AHP assigned a global weight of 0.1366 to this indicator, surpassing cognitive metrics such as Local History (global weight = 0.0335). This empirical result validates its uniqueness: the indicator integrates local storytelling with Byram's ICC model [5]. In this model, "interpretation and negotiation" are central to intercultural competence. This innovation addresses Canagarajah's [13] critique of task-based approaches neglecting cultural agency by operationalizing it through measurable narrative tasks that require learners to actively engage with and represent local cultural practices.

Additionally, the secondary indicator of Promotion and Preservation of Local Image (global weight= 0.1366, M = 5.00) has received high endorsement from experts, highlighting the practical value of constructing cultural discourse power. As operationalized in S5 Table, this sub-indicator (Ⅱ-15) provides measurable criteria for assessing students' international communication competence in university EFL contexts. By designing international communication scenario simulations, students, under the guidance of professors, can clarify and refute misconceptions about local culture disseminated by foreign media. These practices operationalize the scholars' view that EFL teaching needs to enhance the application of cultural knowledge [3,12].

**Emotional dimension**

In the emotional dimension, the study shows balanced emphasis on Cultural Pride (global weight=0.1181) and Openness in Cross-Cultural Engagement (global weight=0.1181). These results are consistent with Kramsch's [26] view that language education should avoid cultural essentialism and cultivate students' hybrid identity capabilities. This equal emphasis, addressing the limitations of earlier models that either overemphasized cultural loyalty or prioritized global perspectives at the expense of local roots [52] , is operationalized through specific teaching tasks in university EFL courses.

In university EFL assessment, cultural pride is evaluated through tasks such as the "Pride Portfolio" (II-10 in S5 Table). For example, anthropology students' cultural awareness is measured by their ability to compile cultural artifacts with precise English annotations (e.g., distinguishing "butterfly patterns symbolizing ancestral blessings" from generic "insect decorations"), demonstrating how linguistic accuracy reflects cultural symbol interpretation in evaluation criteria.

Similarly, cross-cultural openness is assessed via the "Cultural Exchange Archive" (II-11 in S5 Table), where evaluation focuses on students' capacity to create bilingual packages—documenting local traditions like Dragon Boat Races in English while critically analyzing foreign customs (e.g., Christmas) from exchanged materials.

In university exchange programs, learners' performance is assessed through comparative tasks: basic learners might contrast festival food practices via infographics, while advanced learners conduct semiotic analyses of festival symbols in academic posters. This bidirectional comparison aligns with Delvin's [53] "critical cultural awareness" for balanced cultural representation. The equal AHP weights for Cultural Pride and Openness (0.1181 each) validate this assessment approach. The equal AHP weights for Cultural Pride and Openness (0.1181 each) validate this approach. It also embodies CLIL principles [18,19], integrating language learning with cross-cultural inquiry.

**Cognitive dimension**

In the cognitive dimension, the study observed significant divergence in expert opinions during the first Delphi round, particularly regarding abstract cultural indicators such as the Local Political System and Local Economic System (CV = 0.45 and CV = 0.36, respectively). These high coefficients of variation suggest that experts questioned the relevance of such institutional topics in the context of EFL learning. This is consistent with Coyle's view that language learning should be combined with meaningful content and communicative practices, rather than just focusing on abstract cultural knowledge [54]. Therefore, by deleting or merging these abstract topics, the study ensures that cultural content is both comprehensible and relevant to university students.

Under the main indicator Local Cognition and Understanding, the three secondary indicators - Daily Life Experiences, Local Cultural Practices, Art, and Literature, and National Virtues and Qualities - exhibited perfect evaluative consensus (CV=0.00). This consensus confirms the critical role of culturally-embedded assessment tasks in measuring EFL learners' ability to mediate local knowledge through English, as exemplified by "a day in the life" documentation (II-4) and local figure narration (II-5) tasks in S5 Table. Aligned with Alakrash's [2] cognitive authenticity principle, these evaluation criteria demonstrate how contextually-grounded content enhances linguistic encoding and identity positioning in assessment outcomes. The transformation task for local cultural artifacts (II-2 in S5 Table), for instance, evaluates students' symbolic mediation competence through parameters including: (1) cultural authenticity of adapted content, (2) linguistic accuracy in performance scripts, and (3) effective conveyance of embodied cultural meanings. Such multidimensional assessment operationalizes Kramsch's [49] "symbolic competence" framework, where successful performance necessitates transcending lexical equivalence to achieve semiotic resonance between form and cultural substance.

The substantial weighting of Local Development Achievements (global weight=0.0335) underscores the evaluation framework's recognition of contemporary cultural production as equally significant as traditional manifestations. This sub-indicator measures learners' techno-cultural literacy through their ability to mediate between abstract cultural values and concrete development metrics. In university assessment contexts, business students' cultural awareness is measured through tasks like creating "local development data visualization" reports in English (e.g., analyzing cultural industry GDP contributions) . Successful performance requires operationalizing Porto's [3] knowledge model by contextualizing technological and cultural narratives within specific development metrics. The assessment thus validates learners' capacity to concretize socio-cultural abstractions through quantitative discourse. This evaluative approach bridges the epistemic divide between cultural studies and development economics in EFL contexts.

Notably, the indicator Local and Global Issues in Everyday Contexts (M = 3.93, global weight=0.0200), though scoring relatively low, was retained by experts for its role in cultivating critical global citizenship. In university EFL teaching, this is operationalized through graded tasks: advanced learners' critical analysis capabilities are measured through English debates on "local issues with global implications" (e.g., "ancient town tourism vs. cultural authenticity preservation"), while intermediate learners demonstrate their understanding through structured pro-con reports on topics like "dialect protection and tourism development." This differentiated assessment approach validates Gu's [22] theoretical position that internationalized curricula can sustain cultural heterogeneity through critical engagement. The evaluation criteria operationalize Gu's conceptual integration of practical language skills and cultural awareness—specifically measuring learners' capacity to articulate local identities while navigating global discourse frameworks.

**Integration with existing standard frameworks in university context**

The proposed local cultural awareness assessment model provides a comprehensive approach to evaluating EFL learners' engagement with local culture. Furthermore, the practical application of this model can be aligned with widely recognized standard frameworks, such as the Common European Framework of Reference for Languages (CEFR) and the American Council on the Teaching of Foreign Languages (ACTFL) proficiency guidelines. Although this study highlights the blind spots of current frameworks (such as CEFR and ACTFL) in assessing local cultural expression, the new model is not intended to replace these standardized systems but to achieve synergistic enhancement through a hierarchical complementary strategy.

The CEFR offers a comprehensive set of language proficiency descriptors spanning diverse proficiency levels [56]. We propose integrating the local cultural awareness assessment model with the CEFR starting from the intermediate level, employing a descriptor transplantation approach. Specifically, this integration can be achieved by embedding local cultural indicators into the CEFR's mediational and plurilingual descriptors, as underscored in the document regarding "action-oriented scenarios" and "plurilingual repertoires" [56]. As outlined in S6 Table, this strategic embedding aligns secondary indicators with CEFR levels B1 to C2, illustrating how descriptors like "relaying specific information" (B2) and "facilitating pluricultural space" (C1) can be adapted to assess local cultural competence.

B1–B2 Intermediate Level Integration: At these levels, foundational local cultural indicators (e.g., Ⅱ-1 Local History, Ⅱ-4 Daily Life Experiences) can be integrated into CEFR's "Mediating Concepts" and "Reception of Information" descriptors. For instance, Ⅱ-1's requirement for learners to "discuss local history events in English" aligns with CEFR B2's descriptor for "relaying specific information" (Section 3.1.4.2)[56], where learners are expected to "summarize historical contexts with cultural significance". Similarly, Ⅱ-6 (Local Ethical and Legal Systems) can be grafted onto CEFR's B2 "Explaining Data" scale, as the document highlights the importance of mediating social norms through language.

C1–C2 Advanced Level Integration: At higher proficiency tiers, sophisticated cultural indicators (e.g., Ⅱ-15 Local Image Promotion, Ⅱ-18 Cultural Comparison) map to CEFR's "Plurilingual Mediation" and "Facilitating Pluricultural Space" domains. For example, Ⅱ-18's requirement to "compare local and target cultures" directly aligns with CEFR C1's descriptor for "mediating cross-cultural dialogue" (Section 7.2.2), where learners are expected to "negotiate cultural differences in complex scenarios". Additionally, Ⅱ-15's focus on "promoting local identity in English" resonates with the emphasis of CEFR on "activating plurilingual repertoires for social agency" , which can be embedded in CEFR C2's "Strategic Mediation" scale.

Cross-Level Task Design for Cultural Embedding: The document's advocacy for "scenario-based assessment" (Section 7.2.1) provides a framework for designing tasks that bridge proficiency levels [56]. For instance, in B1–B2 tasks, learners could complete a "local food culture interview" (Ⅱ-4) using CEFR B2's "Oral Interaction" descriptors, which emphasize "describing daily practices with cultural relevance". And in terms of C1–C2 tasks, A "local heritage preservation campaign" (Ⅱ-15) could be evaluated against CEFR C1's "Mediation of Public Discourse" criteria, requiring learners to "craft persuasive messages that integrate local values" .

Similarly, ACTFL emphasizes "intercultural communicative competence," focusing on the appropriate use of language in real social and cultural contexts [33]. We propose using the evaluation indicators established in this study to form a dual-track assessment mechanism (Fig 4). For example, a cultural awareness dimension can be added to the ACTFL Oral Proficiency Interview (OPI), allowing independent scoring of language fluency and cultural mediation while sharing weights (suggested ratio: 70% language + 30% culture). This integrated solution, which balances the emphasis on language and cultural competence, retains the reliability and validity of standardized tests and addresses the missing identity expression through the cultural competence matrix (S7 Table). It responds to Deardorff's [35] call for a "mixed assessment method" (quantitative + qualitative) to measure cultural depth. Meanwhile, the dual-track model proposed in this study requires minimal modification to the existing ACTFL protocols but significantly enhances ecological validity in multilingual contexts.

**Limitations and future directions**

This study represents an initial exploration of evaluating local cultural awareness among EFL learners through a Delphi-based framework, specifically contextualized within the Chinese educational landscape. The current expert panel, consisting solely of 15 professionals from China, offers a perspective on integrating local content into EFL education. However, this composition may limit the panel's ability to fully assess the adaptability of our framework across different cultural and educational contexts. To mitigate this limitation, future research should consider expanding the panel to include experts from a variety of EFL settings, such as Latin America, Africa, and Southeast Asia, ensuring a more comprehensive validation of the framework's universal applicability.

Building on this foundation, subsequent studies could pursue two complementary paths. First, cross-cultural validation in non-Chinese settings would shed light on whether the evaluation index system requires contextual adjustments or demonstrates transferable utility. Such efforts would enrich the framework by incorporating varied perspectives on balancing local and global cultural elements in EFL curricula. Second, longitudinal investigations tracking how local cultural awareness influences students' language development and intercultural competence over time would deepen our understanding of its educational impact. Mixed-method designs combining quantitative assessments with qualitative case studies could offer nuanced insights into how learners navigate cultural hybridity in different linguistic environments.

Notably, this research establishes a critical starting point for discussing local cultural integration in EFL education, particularly in contexts where similar cultural priorities may exist. By acknowledging the potential for broader application and inviting interdisciplinary dialogue, future studies can refine the framework to accommodate the diverse needs of EFL learners worldwide, while retaining sensitivity to regional cultural identities. This approach ensures that educational interventions can both honor local voices and foster global communicative agility."

### Conclusion & Future Work-Suggestion 1

**- Suggest pilot testing in non-Chinese contexts.**

**Response**

We would like to express our sincere gratitude for the insightful comments and constructive feedback provided. Your suggestion to pilot test our evaluation framework in non-Chinese contexts is invaluable and aligns perfectly with our vision for the broader applicability of our research.

The Discussion now explicitly addresses this recommendation through both limitation acknowledgment and future research directions, as detailed below:

“....*However, this composition may limit the panel's ability to fully assess the adaptability of our framework across different cultural and educational contexts. To mitigate this limitation, future research should consider expanding the panel to include experts from a variety of EFL settings, such as Latin America, Africa, and Southeast Asia, ensuring a more comprehensive validation of the framework's universal applicability."* ( Page 44, Line [774-779])

*"....subsequent studies could pursue two complementary paths. First, cross-cultural validation in non-Chinese settings would shed light on whether the evaluation index system requires contextual adjustments or demonstrates transferable utility. Such efforts would enrich the framework by incorporating varied perspectives on balancing local and global cultural elements in EFL curricula. " ( Page 44, Line [780-784])*

".....future studies can refine the framework to accommodate the diverse needs of EFL learners worldwide, while retaining sensitivity to regional cultural identities. This approach ensures that educational interventions can both honor local voices and foster global communicative agility."( Page 45, Line [793-796])

This addition directly addresses your recommendation by integrating non-Chinese context validation as a core future direction, ensuring the framework's robustness beyond the current sample. We appreciate your insight, which enhances the study's generalizability and practical impact.

### Note

**Please add DOIs with all references.**

**.**

**Response**

Thank you so much for your meticulous review! We truly appreciate your attention to detail, as it has helped us strengthen the manuscript significantly. All references have now been updated to include DOIs and carefully formatted following the journal's Vancouver style requirements. Your guidance ensures our work meets the highest academic standards, and we're grateful for the opportunity to refine the manuscript based on your suggestions!
