# Supplementary material for: Assessing local cultural awareness in university EFL learners: A Delphi and AHP-based index framework
Source: PLoS One. 2025 Oct 8;20(10):e0332233. doi: 10.1371/journal.pone.0332233 (PMC12507305; doi:10.1371/journal.pone.0332233)
Supplement: Attachment — Submitted filename: Response to Reviewers.docx [file pone.0332233.s013.docx]

# **Response Letter for Manuscript ID:PONE-D-25-18530R1**

Dear Dr. Muhammad Zammad Aslam and Reviewers,

We wish to express our sincere gratitude to you and the reviewers for the valuable comments and constructive suggestions on our manuscript entitled "Assessing local cultural awareness in university EFL learners: a Delphi and AHP-based index framework" (Manuscript ID: [PONE-D-25-18530R1]). These comments have been immensely helpful in improving the quality of our paper.

We have carefully considered all the points raised and have made extensive revisions to the manuscript accordingly. Below, we provide a point-by-point response to the comments. All changes in the manuscript have been highlighted in blue for the reviewers' convenience in the "Revised Manuscript with Track Changes" file.

Contents

[Response Letter for Manuscript ID:PONE-D-25-18530R1	1](#_Toc20026)

[Responses to Comments from Reviewer #1	3](#_Toc4337)

[Comment 1	3](#_Toc9557)

[Comment 2	5](#_Toc3264)

[Comment 3	6](#_Toc31278)

[Comment 4	8](#_Toc21125)

[Comment 5	10](#_Toc1160)

[Comment 6	11](#_Toc9878)

[Comment 7	12](#_Toc7322)

[Comment 8	13](#_Toc22710)

[Responses to Reviewer #2	15](#_Toc29144)

[Comment 1	15](#_Toc1604)

[Comment 2	17](#_Toc4342)

[Comment 3	22](#_Toc8877)

[Comment 4	24](#_Toc31508)

[Comment 5	28](#_Toc8299)

# Responses to Comments from Reviewer #1

Comment 1
****Implicit Suggestion on Title Wording****

**Response**

Dear Reviewer,

We sincerely appreciate your careful review of our manuscript, and we noted your highlighting (in yellow) of the preposition "in" in the title, reflecting your insightful observation on the choice of preposition. We understand that the alternative phrasing "local cultural awareness of university EFL learners" (with "of" instead of "in") could also be a valid and perhaps more conventional way to express this concept, and we value your thoughtful feedback on this linguistic choice.

After careful consideration, we have decided to retain the original formulation ("awareness in learners"), as it aligns with established conventions in applied linguistics research. For instance, Vandergrift (2006) refers to "metacognition in learners". Such structures—"competence/awareness in [population]"—explicitly denote an internal cognitive capacity rather than an external attribute. Furthermore, this phrasing is consistent with the terminology used in our abstract, where we refer to "awareness in EFL education."

Should you prefer the "of" construction, considering it may better suit the journal's style or conventions, we are fully prepared to adopt it in the final version. We highly appreciate your expertise regarding this nuanced use of prepositions, and we remain committed to ensuring the precision of our manuscript in line with your guidance.

Comment 2
**"Title says learners. Here, it says curricula, which one is the aim?"**

**Response**

Dear Reviewer,

Thank you sincerely for your insightful observation regarding the inconsistency between the title (which focuses on "learners") and the mention of "curricula" in the text. This highlights a potential ambiguity regarding the primary focus of our study, and we truly appreciate your careful review, which is invaluable in helping us enhance the clarity of our manuscript.

To clarify, our core aim remains centered on assessing university EFL learners' cultural awareness, as stated in the title. The mention of "curricula" refers to the contextual motivation for this study—specifically, addressing the neglect of cultural awareness development in current curricula, which underscores the need for such learner-focused research. To address this potential ambiguity, we have revised the first sentence of the abstract to clarify our focus. The updated sentence (Page 1, Lines 14–17) now reads:

*"To address the neglect of learners' local cultural identity in their educational development, this study develops and validates an evaluation framework for assessing their cultural awareness, with direct implications for curriculum reform."*

Thank you again for your valuable feedback, which has helped us refine the clarity and precision of our work. We hope these revisions fully address your concern.

Comment 3
****citation****

**Response**

Dear Reviewer,

We sincerely appreciate your critical feedback on the phrasing and citation of the specific term in our manuscript. Your insights have enabled us to identify critical issues requiring resolution, and we deeply value your meticulous attention to such details.

Yes, the original phrasing contained two notable shortcomings. First, it included an informal and potentially misleading assertion about the term's origin, which was not presented in the standardized citation format. Second, it involved a redundant and non-compliant citation practice: by naming the term's originator in the main text while simultaneously using a numerical citation [1], this conflicted with the Vancouver reference style guidelines.

To resolve these issues, we have revised the relevant sentences to ensure strict compliance with the Vancouver reference style. The updated sentences (Page 1, Lines 31–36) now read:

*"In today's globalized world, English proficiency functions as an essential skill for cross-border communication. Consequently, English as a Foreign Language (EFL) programs increasingly aim not only to cultivate linguistic competence but also to foster global engagement capabilities. However, learners frequently encounter 'Cultural Aphasia' [1]—defined as the persistent difficulty in conveying native cultural nuances despite advanced foreign language proficiency"*

Once again, thank you for your invaluable guidance in enhancing the precision and formality of our work. We trust these revisions have sufficiently addressed your concerns.

Comment 4

"****What does this mean? What does CEFR address and what is not addressed? "Does this mean "lack tools to evaluate how they explain their cultural identities in English?"****

**Response**

Dear Reviewer,

Thank you for your critical questions—they have been invaluable in sharpening the precision and clarity of our argument. Upon reflection, we acknowledge our original description of the CEFR's limitations in assessing local cultural identity was overly vague, and we appreciate your guidance in addressing this.

We have comprehensively revised the paragraph to deliver a more nuanced, accurate, and evidence-based analysis. Key improvements include:

1.Enhanced specificity: We replaced abstract critiques with a concrete example, citing the CEFR B1 reception descriptor to ground our argument in a tangible standard.

2.Balanced framing of "mediation": We incorporated an authoritative definition [35] to clarify the CEFR's view of mediation as a socio-cultural practice for collaboration and conflict resolution.

3.Precise gap identification: By grounding the research gap in concrete descriptors and contrasting it with the framework's robust mediation descriptors, we clarify that the gap extends beyond mere omissions to a structural limitation in evaluating this important dimension of local awareness.

We hope these revisions significantly improve the rigor of our analysis. Thank you again for your insightful feedback. Your guidance has been instrumental in refining our work. We hope these changes fully address your concerns.

The updated paragraph (Page 10, Lines 199–210) now reads:

*"For example, the CEFR's 'can do' descriptors primarily emphasize learners' ability to function in target-language contexts—for instance, 'understanding main points of clear standard input on familiar matters (work, school, leisure)' (CEFR B1) [35]. While the framework includes mediation as a key communicative activity, defined as a socio-cultural practice that 'promotes communication and collaboration' and 'resolves sensitive issues' across languages and cultures [35], this concept is largely framed as a gap-bridging process. It positions learners as neutral facilitators or interpreters, yet crucially omits explicit descriptors to assess their ability to proactively express, explain, or defend the unique elements of their cultural identity in English. This omission undermines the evaluation of cultural self-representation—a critical component of balanced intercultural communication [35]."*

Comment 5

****Citation problem****

**Response**

Dear Reviewer,

Thank you for raising this important question about citation formatting. We appreciate your attention to detail, as precise referencing is critical to academic rigor.

We confirm that all in-text citations have been revised to fully comply with the Vancouver reference style.

Please let us know if you require further clarification or additional adjustments—we are happy to refine this further to meet the journal's requirements.

Comment 6

"****Are these the Delphi panels?"****

**Response**

Dear Reviewer,

Thank you for this crucial question. It helps us clarify an important methodological point.

To answer directly: No, the focus group discussions and the formal Delphi panel were not the same. The focus group discussions were done by our research team as a first step. We used them to develop and improve the initial list of indicators.

The formal Delphi panel was a separate group. It included 15 external experts who did not work on the research design. These experts were not part of the early discussions.

We have changed the manuscript to make this clear. The updated sentences (Page 14, Lines 285–290) now read:

*"Concurrently, the research team (all front-line university EFL teachers) held two rounds of internal discussions to refine the initial indicator set by clarifying concepts, checking for redundancy, ensuring domain coverage, and verifying operational feasibility. Following this, a separate panel of 15 external EFL education experts was invited to participate in the formal Delphi consultation process."*

Thank you again for your question. It helped us make our methods clearer.

Comment 7

"****Scholar?"****

**Response**

Dear Reviewer,

Thank you for asking this important question. It helped us realize that our previous description was not clear enough about the theoretical foundation of our framework.

To answer directly: The core model we built upon is Byram's model of Intercultural Communicative Competence (ICC) , which clearly outlines the cognitive, affective, and behavioral dimensions. Our study then integrates Kramsch's perspective on symbolic competence to adapt and enrich Byram's model for the specific context of local cultural awareness.

We have revised the manuscript to state this much more clearly. The updated sentences (Page 14, Lines 294–297) now read:

"*The assessment framework was grounded in a Kramsch-informed adaptation of Byram's tripartite ICC model. The Kramsch-Byram integration achieves theoretical synergy: Byram's measurable dimensions are conceptually enriched through Kramsch's historicity-positioning-praxis triad [11,37].* "

Thank you again for your question, which has significantly improved the precision of our theoretical explanation.

Comment 8

"**To be clear, these primary and secondary indicators are all developed from literature review, following the tripartite model, right?"**

Response

Dear Reviewer,

Thank you for your question, which correctly identifies the source of our initial indicators.

Yes, your understanding is precise. The preliminary set of 3 primary and 24 secondary indicators was indeed derived from the synthesis of existing literature, structured explicitly according to the cognitive, affective, and behavioral dimensions of the Kramsch-Byram tripartite model.

To improve conciseness and avoid redundancy in the manuscript, we have replaced the detailed list with a reference to the supplementary table. The revised sentence now reads:

*"This framework generated 3 primary indicators with 24 secondary sub-indicators for initial Delphi validation (S1Table)."*

Finally, we would like to express our deepest gratitude once again for the tremendous time, energy, and care you have devoted to reviewing our manuscript. It is precisely your profound academic insights, meticulous attention to detail, and rigorous scholarly attitude that have guided us to identify gaps in our work we might otherwise have overlooked—and in turn, elevate the overall quality, clarity, and rigor of the study. Your thoughtful feedback has not only refined this manuscript but also provided invaluable inspiration for our future research. We are truly honored to have the opportunity to polish our work under your guidance, and we sincerely hope the revised version meets your expectations. Thank you again for your unwavering support and constructive guidance—your contributions have been instrumental to the improvement of our study.

# Responses to Reviewer #2

### Comment 1

**The manuscript is excessively long, with numerous repetitive examples, citations, and table explanations (e.g., extensive elaboration on indicators that repeat the same construct wording). The Results section, while data-rich, suffers from redundancy between narrative and table content. Condense repeated discussions on Delphi/AHP procedures. Consider moving detailed indicator breakdowns to appendices or supplementary files.**

**Response**

Dear Reviewer,

Thank you very much for your valuable comments on our manuscript. We sincerely appreciate your insightful suggestions and fully agree with your point that there is redundancy between the narrative and table content.

To address this issue, we have made the following revisions:

In the revised Results section, the narrative content focuses on summarizing key findings and trends, avoiding a point-by-point restatement of the table contents. Tables are used to present detailed data, ensuring that the text and tables complement each other without unnecessary repetition.

For the Delphi method results (Tables 6-10, Pages 21-25, lines 417-477), we have retained the key narrative describing the changes in consensus. We have significantly streamlined the detailed descriptions in the main text of each indicator's specific scores (mean, standard deviation, coefficient of variation) and their changes as these details are clearly presented in the tables. The main text now only highlights the most important changes or the indicators with the highest/lowest scores as examples.

Regarding the Analytic Hierarchy Process (AHP) results (Tables 11, 12, Pages 26-28, lines 485-518), we have optimized the expression. Table 12 originally included not only the weights but also detailed operational definitions of all 18 secondary indicators, which caused redundancy, especially considering that these indicators might be referenced in the discussion section. To solve this problem, we have removed the descriptions of each indicator from Table 12 in the main text. The detailed indicator definitions have been moved to Supplementary Table S5, titled "Operationalization Framework for Local Cultural Awareness Assessment".

Additionally, we have conducted a comprehensive check of redundancy throughout the entire manuscript to ensure consistency in conciseness. All sections have been reviewed to eliminate overlapping content and improve overall clarity.

Thank you again for your thoughtful guidance and careful review. We hope these adjustments bring the manuscript even closer to your vision for precision and conciseness. And we're eager to contribute to your positive experience with our work.

### Comment 2

**"Kramsch and Byram are central, but no conceptual model or diagram links the three dimensions (cognition, affect, behavior) to symbolic competence. While symbolic competence is mentioned, symbolism as a construct is underdeveloped. Add a conceptual model figure showing how symbolic competence maps onto your three dimensions. Also, better define how your index extends vs. operationalizes Kramsch's theory."**

**Response**

Dear Reviewer,

Thank you from the bottom of our hearts for this invaluable comment, which reflects your profound academic attainments and solid theoretical foundations. Your insight has pinpointed a profoundly critical issue—one that cuts to the core of our theoretical framework and had, regrettably, escaped our full attention. The questions you've raised are not only incisive but also reveal a depth of understanding that speaks to your extraordinary expertise in the field. Our entire research team has expressed sincere admiration for your ability to identify such a pivotal theoretical gap with such clarity and precision. We fully recognize that this observation addresses a key weakness in our theoretical elaboration, and we are deeply grateful for your guidance in illuminating this area.

To address your suggestions, we have implemented the following key revisions:

**1.Strengthened theoretical elaboration on symbolic competence**: We have added a dedicated subsection in the literature review titled "Theoretical Foundation: Symbolic Competence in Local Cultural Expression" to deepen our engagement with Kramsch's theory. This new section offers a comprehensive analysis of symbolic competence, with a dual focus on its core components and its specific relevance to local cultural expression—an aspect we previously underdeveloped.

The revised content can be found in Page 5, Lines [88-108]:

*"Kramsch's theory of symbolic competence [11] establishes the epistemological framework for local cultural awareness in EFL contexts. This theory redefines language learning as a dynamic process of meaning negotiation through historically embedded symbolic systems, contrasting sharply with transactional communication models [1,3].*

*Central to this perspective is the treatment of linguistic forms as semiotic resources that construct cultural identities rather than transmit information [12] . When EFL learners engage with English, they occupy a hybrid "third space" [13] where global and local narratives intersect, necessitating active navigation of one's identity through symbolic mediation. This process requires historicity—an awareness of how language preserves cultural memory and socio-political legacies that shape modern expression [11,13].*

*This framework directly explains the "cultural aphasia" phenomenon introduced earlier. Kramsch's lens interprets such communicative failures not as lexical deficits but as breakdowns in wielding English for cultural self-representation [11]. Consequently, assessing local cultural awareness must evaluate learners' ability to deploy L2 resources for authentic cultural identity expression—a dimension overlooked in standardized EFL assessments. Empirical evidence confirms that structured feedback in EFL writing not only enhances cultural articulation, but also scaffolds the development of cultural awareness, enabling learners to express local identities more effectively in English [14]."*

2. **Added a conceptual model figure**: Following your valuable suggestion, we have carefully integrated a new Figure 1 titled "Conceptual Model of Symbolic Competence Operationalization" into the literature review section. This addition directly responds to your insightful observation that no prior diagram explicitly linked our three core dimensions (cognition, affect, behavior) to Kramsch's symbolic competence framework.

The figure visually illustrates how Kramsch's symbolic competence—particularly its emphasis on meaning-making and cultural negotiation—serves as the foundational underpinning for our three-dimensional structure. By mapping the theoretical interplay between each dimension (cognition, affect, behavior) and the core elements of symbolic competence, we aim to clarify the often implicit connections between abstract theory and our operationalized index. This adjustment not only strengthens the conceptual coherence of our work but also directly addresses your constructive feedback to enhance reader understanding.

**Fig 1. Conceptual model of symbolic competence operationalization**

**3.Revised methodological workflow figure:** We have thoughtfully revised and renamed the original Figure 2 to "Methodological Workflow Integrating Theoretical Frameworks with Delphi-AHP Procedures." This adjustment was made to directly address your valuable insight regarding the need to clarify how our theoretical constructs—particularly Kramsch's symbolic competence and Byram's framework—connect to our methodological processes, as well as how our index operationalizes and extends Kramsch's theory. By renaming and refining this figure, we aim to enhance clarity for readers and ensure stronger alignment with your constructive feedback.

**Fig 2. Methodological workflow integrating theoretical frameworks with Delphi-AHP procedures**

4. **Clarified theoretical contributions in the discussion:** We sincerely appreciate your valuable feedback prompting us to clarify how our index operationalizes versus extends Kramsch's theory. In response, we have thoughtfully added a dedicated subsection titled "Operationalizing and Extending the Kramsch-Byram Operationalizing and extending the Kramsch-Byram Synthesis" within the Discussion section (pp. 29–30, Lines 539-549).

*"The empirical findings and indicator weights operationalize and extend the integrated Kramsch-Byram framework (Fig 2-Fig 3). Kramsch's symbolic competence (see Literature Review) positions language as a semiotic resource for meaning negotiation, identity construction, and navigating the "third space" within cultural historicity, while Byram's ICC model supplies the structural assessment dimensions.*

*Crucially, the index system concretizes Kramsch's symbolic action tenets—particularly active negotiation and inventive meaning construction within the third space—into specific dimensions, indicators, and assessment tasks. The framework further advances Kramsch's conception by integrating affective drivers, emphasizing native culture application, linking local symbols to global discourse, and providing pedagogical specificity for implementation."*

We further advance his framework by integrating elements critical to our EFL context. These modifications are further reinforced in the revised subsections:

1).In the subsection of Local Expression and Application dimension, we now explicitly link "storytelling" (II-14) to Kramsch's "third space" by illustrating how learners' narrative practices mediate cultural meaning (pp. 30–33, lines 551-612).

2).The emotional dimension now clarifies how "cultural pride" and "cross-cultural openness" (II-10, II-11) extend Kramsch's focus on "identity construction" by balancing self-affirmation with global openness (pp. 33–34, lines 612-644).

3).In the subsection ofCognitive dimension, we detail how "local language use" (II-3) operationalizes Kramsch's "semiotic resource" theory through measurable tasks (e.g., "translating local proverbs into English")(pp. 34–37, lines 645-697).

These revisions are designed to enhance the theoretical coherence of our study, making the linkages between foundational theories and our research design more transparent. We truly believe that, thanks to your guidance, these changes have not only filled the theoretical gaps but also elevated the overall rigor and depth of our work. Please accept our heartfelt gratitude once again for your invaluable input—your wisdom has been a tremendous inspiration, and we are committed to ensuring the final manuscript lives up to the standards your feedback has helped us strive for.

### Comment 3

**All 15 experts are from mainland China, and most have similar academic backgrounds. This weakens the cross-cultural applicability of the index. No diversity in learner or stakeholder perspectives (e.g., learners, employers, teachers from different systems). Acknowledge this limitation explicitly in the Discussion. Consider a future validation phase in diverse contexts (e.g., South Asia, ASEAN) as a next step.**

**Response**:

Thank you sincerely for raising these critical points about the limitations of our expert panel and stakeholder representation—we deeply appreciate your insight, as it has helped us strengthen the transparency and forward-looking direction of our work.

We fully acknowledge your concern that the homogeneity of our current expert panel (15 Chinese professionals with similar academic backgrounds) may constrain the cross-cultural applicability of our index system. In response, we have explicitly acknowledged this limitation in the revised "Limitations and Future Directions" section (Page 40, Lines 762–764), stating: "*Although the panel of 15 Chinese experts provides valuable insights, its homogeneous composition is a significant limitation. Their shared background may constrain the cross-cultural applicability of the index system.* "

We also agree that the absence of key stakeholder perspectives—such as EFL learners, employers, and educators from diverse educational systems—limits our understanding of the framework's practical implementation. To address this, we have expanded the discussion of future research to emphasize the inclusion of these groups. Specifically, we now note: "...*the lack of input from key stakeholders—such as EFL learners, employers, and educators from diverse educational systems—restricts our understanding of the framework's practical implementation across user groups. (Page 40, Lines 765–767)*

Regarding your suggestion to expand validation to diverse contexts (e.g., South Asia, ASEAN), we have strengthened our future directions to include concrete steps for cross-cultural testing. For example, we now specify: "*cross-cultural validation in non-Chinese settings would involve not only assessing contextual adjustments but also conducting empirical pilot tests (e.g., pre/post-intervention surveys measuring learners' intercultural competence) to quantify the framework's effectiveness across diverse EFL contexts." (Page 40, Lines 769–772)*

Finally, we have emphasized the integration of mixed-method designs in longitudinal studies to capture nuanced stakeholder experiences, as you recommended: *"longitudinal investigations are needed to examine the long-term impact of local cultural awareness on students' language development and intercultural competence.,Such research must employ mixed-methods designs, triangulating quantitative data (e.g., from standardized tests) with qualitative evidence (e.g., from learner diaries or case studies) to capture nuanced insights into how learners navigate cultural hybridity across different linguistic environments." (Page 40, Lines 775–780)*

Your feedback has been invaluable in refining our discussion of limitations and future steps. We are committed to acting on your suggestions to enhance the framework's robustness and applicability, and we hope these revisions adequately address your concerns.

Thank you again for your thoughtful engagement with our manuscript. We deeply value your expertise and look forward to contributing to the field with a more inclusive and rigorously validated tool.

### Comment 4

**While AHP calculations are provided, rationale for certain weightings lacks theoretical grounding. For instance, why is storytelling (13.67%) weighted more heavily than historical understanding (3.35%)? The decision to equal-weight affective attitudes like "pride" and "critical openness" is sociopolitically sensitive—more explanation is needed.**

**Justify key weight assignments with either empirical pilot results or pedagogical priorities tied to established models (e.g., Bloom's, Vygotsky).**

**Response**

We are deeply grateful and feel lucky to have you as the reviewer for these insightful and critical comments. Your suggestions have allowed us to significantly strengthen the theoretical and pedagogical justification for our AHP weight assignments in the revised manuscript. We have addressed each point of concern by embedding our explanations within the core theoretical framework of our study (Kramsch & Byram) and by incorporating supporting empirical evidence where available.

Our key revisions are as follows:

**1. Theoretical Justification for "Storytelling > History" Weighting:**

We have expanded the Discussion in the "Local Expression and Application Dimension" section to explicitly contrast the higher-order demands of storytelling against the foundational nature of historical knowledge. The weighting disparity is now justified as a direct operationalization of Kramsch's Symbolic Competence and Byram's Action-Orientation because storytelling requires creating meaning and engaging in symbolic action whereas historical understanding focuses on comprehending cultural symbols. This rationale is further validated by strong expert consensus (Kendall's W = 0.86, p < 0.01) from our Delphi process, confirming the pedagogical priority of such agentive practices.

The revised content can be found in Pages 31-32, Lines [571-598]:

*"The secondary indicator of Use of English for Local Storytelling(II-14) holds one of the highest global weights. Learners are not merely translating stories; they are selecting culturally significant symbols (events, figures, legends), constructing a narrative framework in English, and infusing the narrative with cultural meaning. The higher weighting of Use of English for Local Storytelling(13.67%) compared to Local History(3.35%) reflects our adherence to Kramsch's symbolic competence framework, which prioritizes active meaning negotiation over passive knowledge acquisition. Empirical pilot studies [3,18,19,23] have shown that narrative-based tasks significantly enhance learners' ability to mediate cultural identity in English, as they require selecting, interpreting, and reconstructing cultural symbols—skills critical for intercultural communication. In contrast, Local History (a component of cognitive understanding) was assigned lower weight because, while experts (via Delphi rounds) acknowledged its fundamental importance to EFL learning (e.g., providing cultural context for language use), the AHP process prioritized behavioral dimensions due to their more direct and frequent activation in real-world intercultural interactions—where narrative practices often serve as the primary vehicle for mediating cultural identity. This corresponds to Byram's ICC model, which emphasizes practical cultural engagement over rote factual recall.*

*In university EFL courses, this indicator can be applied through collaborative projects: advanced learners may document entrepreneurial histories of local time-honored brands, producing podcasts that weave entrepreneurs' interviews with cultural analyses (Ⅱ-14 in S5 Table). The narrative-driven approach diverges from earlier target-culture immersion studies [5,11], and recent work framing narratives as grammatical tools [54], addressing critiques of task-based methods that neglect cultural agency [15]. In contrast to the focus on refining cultural expression via corrective feedback [14], the index system foregrounds proactive cultural production. It embodies Kramsch's position that narrative empowers learners to explore and demonstrate symbolic power in a foreign language."*

**2.Sociopolitical Sensitivity and Justification for Equal Weighting of "Pride" and "Openness"**

In the "Emotional Dimension" section, we now thoroughly justify the equal weighting (11.81% each) of cultural pride (II-10) and cross-cultural openness (II-11) as essential for fostering hybrid cultural identity, a cornerstone of Kramsch's warning against cultural essentialism which emphasizes avoiding rigid cultural boundaries, as well as Byram's critical cultural awareness which requires both confidence in one's own culture and openness to others.

We have added empirical literature [55] and [56] to support this. The empirical studies (Nelson & Baumgarte, 2004; Cao & Sarsenbayeva, 2023) demonstrated that imbalanced attitudes hinder intercultural dialogue, while this dual focus enhances empathetic communication.

The revised content can be found inPage 33, Lines [620-635]
"*In the emotional dimension, the study shows balanced emphasis on Cultural Pride(Ⅱ-10) and Openness in Cross-Cultural Engagement(Ⅱ-11) . These results are consistent with the view that language education should avoid cultural essentialism and cultivate students' hybrid identity capabilities [28]. This equal emphasis addresses the limitations of earlier models that either overemphasized cultural loyalty or prioritized global perspectives at the expense of local roots [55] . Empirically, surveys revealed that students with imbalanced attitudes (e.g., excessive pride without openness) struggled to engage in meaningful cross-cultural dialogues[56], while those with balanced attitudes demonstrated better cultural empathy and communication adaptability[56]. This aligns with the sociopolitical goal of fostering an inclusive cultural identity—one that values local roots while remaining open to global perspectives—rather than promoting cultural superiority or dilution. Intercultural competence does not require replacing one's existing cultural identity. Rather, it involves expanding the range of identities one can engage with [5]. This perspective justifies the equal emphasis placed on both cultural pride and openness in the current study."*

Throughout the revised Discussion, we tightly link all weight assignments to the Kramsch-Byram integrated framework, framing them as structurally mandated by this theoretical foundation. The weights are further validated by rigorous Delphi expert consensus (e.g., CV = 0.00 for key affective indicators), leveraging this established method to confirm pedagogical soundness from experienced EFL practitioners.

We are confident that these revisions have significantly elevated the quality of our manuscript, and this progress would not have been possible without your meticulous review and profound academic insights. Your rigorous scrutiny and scholarly expertise have been instrumental in driving these meaningful improvements, and we are truly grateful for the transformative impact of your guidance.

### Comment 5

**Numerous grammatical issues, unclear sentence structures, and mixed citation styles (APA, numeric). Some sections (e.g., "Second Delphi survey results") read more like reports than academic narrative. Strong language editing is necessary. Use consistent academic English, e.g., replace "improved indicators" with "refined indicators.".**

**Response**

Dear Reviewer,

We are deeply grateful for the time and care you've invested in reviewing our work, and we're eager to share the meaningful revisions we've made in response to your suggestions—with a focus on addressing concerns related to language precision, citation consistency, and academic narrative depth.

**1.Language Editing and Sentence Clarity:** We have performed a comprehensive language edit throughout the entire manuscript. This involved: 1). Correcting grammatical errors. 2).Restructuring unclear and overly complex sentences to improve readability and flow. 3). Replacing informal or imprecise terms with formal academic language (e.g., "improved indicators" has been replaced with "refined indicators" or "revised indicators" where appropriate across the text).

**2.Citation Style Consistency:** We have carefully standardized the citation format to align with both PLOS ONE's numerical citation style and Vancouver's numbered reference system. Throughout the manuscript and reference list, we now consistently use numeric superscript brackets (e.g., [1], [2, 3]) for in-text citations, ensuring clarity and compliance with the journals' specific guidelines. This adjustment resolves prior inconsistencies in citation formatting, ensuring every reference is presented uniformly—critical for maintaining academic rigor and adhering to the journals' expectations.

**3.Academic Narrative Enhancement:** We have significantly revised sections that were overly descriptive, particularly the "Second Delphi survey results" and other results subsections. We have transformed the narrative from a mere reporting of data to a more analytical discussion. For instance, after presenting statistical values (e.g., mean and standard deviation), we now include brief interpretations explaining the implications of those values for the study's findings.

We warmly hope you and the editor will find these changes satisfactory, and we remain deeply grateful for the opportunity to refine our work under your discerning eye. Thank you again for your support and encouragement.
